# A large-scale metagenomic survey dataset of the post-weaning piglet gut lumen

**DOI:** 10.1093/gigascience/giab039

**Published:** 2021-06-03

**Authors:** Daniela Gaio, Matthew Z DeMaere, Kay Anantanawat, Graeme J Eamens, Michael Liu, Tiziana Zingali, Linda Falconer, Toni A Chapman, Steven P Djordjevic, Aaron E Darling

**Affiliations:** The iThree Institute, University of Technology Sydney, Sydney, NSW 2007, Australia; The iThree Institute, University of Technology Sydney, Sydney, NSW 2007, Australia; The iThree Institute, University of Technology Sydney, Sydney, NSW 2007, Australia; NSW Department of Primary Industries, Elizabeth Macarthur Agricultural Institute, Woodbridge Rd, Menangle NSW 2568, Australia; The iThree Institute, University of Technology Sydney, Sydney, NSW 2007, Australia; The iThree Institute, University of Technology Sydney, Sydney, NSW 2007, Australia; NSW Department of Primary Industries, Elizabeth Macarthur Agricultural Institute, Woodbridge Rd, Menangle NSW 2568, Australia; NSW Department of Primary Industries, Elizabeth Macarthur Agricultural Institute, Woodbridge Rd, Menangle NSW 2568, Australia; The iThree Institute, University of Technology Sydney, Sydney, NSW 2007, Australia; The iThree Institute, University of Technology Sydney, Sydney, NSW 2007, Australia

## Abstract

**Background:**

Early weaning and intensive farming practices predispose piglets to the development of infectious and often lethal diseases, against which antibiotics are used. Besides contributing to the build-up of antimicrobial resistance, antibiotics are known to modulate the gut microbial composition. As an alternative to antibiotic treatment, studies have previously investigated the potential of probiotics for the prevention of postweaning diarrhea. In order to describe the post-weaning gut microbiota, and to study the effects of two probiotics formulations and of intramuscular antibiotic treatment on the gut microbiota, we sampled and processed over 800 faecal time-series samples from 126 piglets and 42 sows.

**Results:**

Here we report on the largest shotgun metagenomic dataset of the pig gut lumen microbiome to date, consisting of >8 Tbp of shotgun metagenomic sequencing data. The animal trial, the workflow from sample collection to sample processing, and the preparation of libraries for sequencing, are described in detail. We provide a preliminary analysis of the dataset, centered on a taxonomic profiling of the samples, and a 16S-based beta diversity analysis of the mothers and the piglets in the first 5 weeks after weaning.

**Conclusions:**

This study was conducted to generate a publicly available databank of the faecal metagenome of weaner piglets aged between 3 and 9 weeks old, treated with different probiotic formulations and intramuscular antibiotic treatment. Besides investigating the effects of the probiotic and intramuscular antibiotic treatment, the dataset can be explored to assess a wide range of ecological questions with regards to antimicrobial resistance, host-associated microbial and phage communities, and their dynamics during the aging of the host.

## Data Description

**Figure 1:**
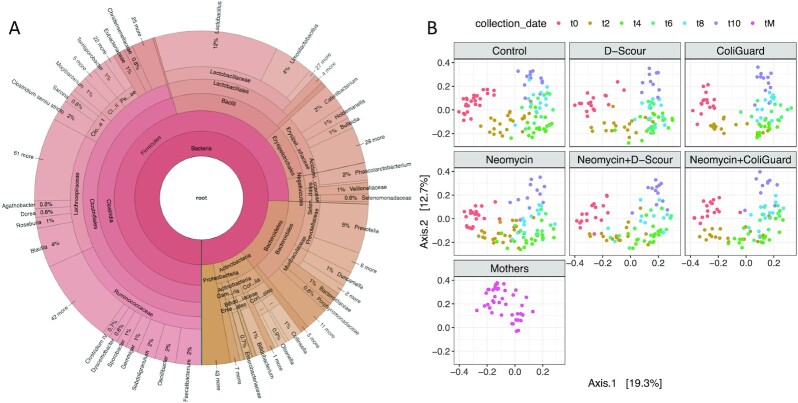
Microbial composition and diversity of the porcine microbiome. Taxonomic profiling based on the analysis of reads containing bacterial 16S rRNA genes extracted from shotgun metagenomic data. (A) The chart was generated using Krona [3], which displays hierarchically organized nodes of the taxonomic tree based on their relative abundance. Distinct colours represent separate domains of life. (B) Principal coordinate analysis plots of the pig faecal microbiomes explaining 19.3% of variance (Axis.1) and 12.5% of variance (Axis.2). Samples are coloured by day of collection from the first week post-weaning (t0) to the fifth week post-weaning (t10). Panels are split by cohort: Mothers (tM; bright pink; *n* = 42); Control (*n* = 30); D-Scour (*n* = 18); ColiGuard (*n* = 18); Neomycin (*n* = 24); Neomycin+D-Scour (*n* = 18); Neomycin+ColiGuard (*n* = 18).

The dataset includes 911 samples, comprising a total of 27 billion raw sequence reads. Preliminary analysis of the dataset consisted in the extraction of 16S ribosomal RNA (rRNA) gene-containing reads with SortMeRNA [1] and their classification with the RDP classifier [2]. In terms of taxonomic diversity, most operational taxonomic units (75.71%) were assigned to the Firmicutes phylum. The next most abundant bacterial phyla were Bacteroidetes (13.21%), Actinobacteria (5.10%), Proteobacteria (3.36%), and *Spirochaetes* (0.69%). A visualization of the microbial composition, obtained with Krona [3], is shown of the post-weaning piglets (Fig 1A) along with the β-diversity of the mothers and the piglets during the first 5 weeks after weaning (Fig. 1B). Interactive Krona maps are available as html files in our Github repository [4].

### Pig trial and sample collection

Animal studies were conducted at the Elizabeth Macarthur Agricultural Institute (EMAI) NSW, Australia, and were approved by the EMAI Ethics Committee (Approval M16/04). The trial animals comprised 4-week old male weaner pigs (*n* = 126) derived from a commercial swine farm and transferred to the study facility in January 2017. These were cross-bred animals of “Landrace,” “Duroc,” and “Large White” breeds and had been weaned at ∼3 weeks of age (Supplementary Table 1).

The pig facility consisted of 4 environmentally controlled rooms (Rooms 1–4) with air conditioning, concrete slatted block flooring with underground drainage, and open rung steel pens (Supplementary Fig. 1). Each room had 9 pens, consisting of a set of 6 and a set of 3 pens, designated a–f and g–i, respectively, with the 2 sets of pens being physically separate; *i.e*., animals could come in contact with each other through the pen's bars within each set of pens but not between sets. The rooms were physically separated by concrete walls and contamination between rooms was minimized by using separate equipment (boots, gloves, coveralls) for each room. In addition, under-floor drainage was flushed twice weekly and the flushed faeces/urine was retained in under-floor channels that ran the length of the facility, so that Rooms 1, 2 were separate from Rooms 3, 4 and flushing was in the direction 1 to 2 and 3 to 4.

The pigs were fed *ad libitum* a commercial pig grower mix of 17.95% protein free of antibiotics, via self-feeders. On the day of arrival (Day 1) 30, 18, 18, and 60 pigs were allocated randomly to Rooms 1, 2, 3, and 4, respectively, in groups of 6, 6, 6, and 6–7 pigs per pen, respectively (Supplementary Fig. 1A). Pigs were initially weighed on Day 2, and some pigs were moved between pens to achieve an initial mean pig weight per treatment of ∼6.5 kg (range: 6.48–6.70 kg; mean ± SD: 6.53 ± 0.08 kg). Pigs were weighed weekly throughout the trial, and behaviour and faecal consistency scores were taken daily over the 6-week period of the trial (Supplementary Table 1). Developmental and commercial probiotic paste preparations ColiGuard® and D-Scour™ from International Animal Health were used in some treatment groups.

The animals were acclimatized for 2 days before the following treatments were administered: Room 1: oral 1 g/pig of placebo paste daily for 14 d; Room 2: oral 1 g/pig of D-Scour™ paste daily for 14 d; Room 3: oral 1 g/pig of ColiGuard® paste daily for 14 d; Room 4: intramuscular injection of antibiotic administered at 0.1 mL per pig daily from a 200 mg/mL solution for a total treatment duration of 5 d.

On the day following the final neomycin treatment (Day 8), 36 pigs were moved from Room 4 to Room 2 (*n* = 18, 6 in each pen, Pens g–i), and to Room 3 (*n* = 18, 6 in each pen, Pens g–i) (Supplementary Fig. 1B). The following day (Day 9), oral administration of D-Scour™ (1 g/pig) and of ColiGuard® (1 g/pig) commenced for pigs in Room 2 Pens g–i and in Room 3 Pens g–i, respectively, and continued for a period of 14 days. Assignment of the 36 neomycin-treated pigs to the treatment groups neomycin+D-Scour™ (*n* = 18; Room 2 Pens g–i) and neomycin+ColiGuard® (*n* = 18; Room 3 Pens g–i) was carried out by distributing them so that the mean weight of the animals distributed across pens and rooms was similar. By this time point, each occupied pen in the trial housed 6 pigs (Supplementary Fig. 1B). From that time, 12 piglets from the original 126 were no longer present because they had been killed as pre-treatment controls at the start of the trial.

**Figure 2:**
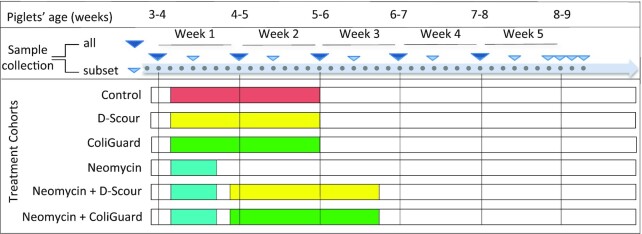
Timeline. Timeline of the animal trial indicating the start and the length of the treatment for each cohort, the sample collection points, and the piglets’ age during the trial. Piglets were allowed 2 days of acclimatization after arrival at the site of the trial and before the start of the treatments (pink: placebo paste; yellow: probiotic D-Scour™ formulation; green: probiotic ColiGuard® formulation; aqua: antibiotic neomycin intramuscular injection). Large triangles (dark blue) indicate main days of sampling where all piglets were sampled (*n* = 126). Small triangles (light blue) indicate sampling points from a subset of the piglets (8 per cohort; *n* = 48).

Faecal samples were collected from all piglets once per week and from a subset (*n* = 48 pigs; 8 from each of the 6 cohorts) twice per week over the 6-week study period (Fig. 2). From each piglet, faeces were collected per rectum with new disposable gloves; where minimal or no faeces could be collected on a collection day, sampling was performed the following morning. Samples were placed in 50 mL Falcon tubes and stored at 4°C within 30 mins of sample collection for a minimum of 30 mins and a maximum period of 6 h.

### Faecal sample processing

**Figure 3:**
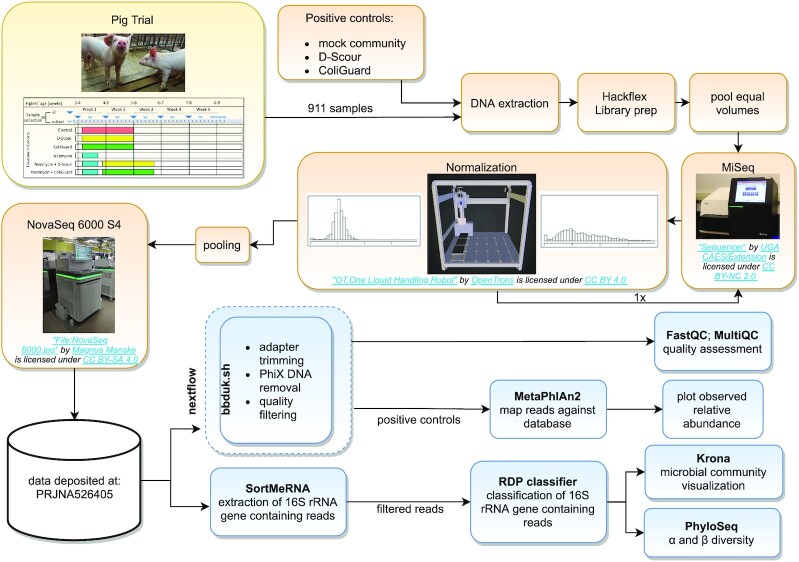
Workflow. A schematic representation of the experimental workflow from sample collection (yellow), through sample processing and sequencing (orange), to the preliminary data analysis (blue).

Samples (3 g/pig) were mixed with 15 mL phosphate-buffered saline (PBS) (200 mg/mL), in sterile stomacher bags and homogenized with a Bio-Rad stomacher. The homogenized samples were divided in replicates: 1 replicate was stored directly at −80°C and 1 replicate was supplemented with glycerol (20% v/v) (Sigma-Aldrich, Castle Hill, NSW, Australia) then stored at −80^o^ C. In addition, single time-point faecal samples from the dams of the trial pigs (*n* = 42) were obtained from the commercial facility of origin and were pre-processed at EMAI as described above. Thus, a total of 911 unique samples, between 1 and 10 samples per subject (mean: 4.8; median: 3) (Supplementary Table 1), were obtained throughout this study. At the end of the trial period, all samples were transported from EMAI to the University of Technology Sydney for further processing. The experimental workflow is schematically represented in Fig. 3.

### Positive controls

As a positive control “mock community” for this study, 4 gram-positive (*Bacillus subtilis* strain 168, *Enterococcus faecium, Staphylococcus aureus* ATCC25923, *Staphylococcus epidermidis* ATCC35983) and 3 gram-negative (*Enterobacter hormaechei* CP_032842, *Escherichia coli* K-12 MG1655, *Pseudomonas aeruginosa* PAO1) bacterial strains from −80°C stocks were cultured at 37°C for 16 h in LB (Luria-Bertani) then centrifuged at 14,000 rpm for 10 mins. From the resulting pellets, 1 g was transferred to 1 mL of LB and homogenized and a 1:10 dilution of this was made for each bacterial culture. A volume of 10 μL of bacterial suspension from each of the cultures was used to determine the number of colony-forming units (CFUs) in the original suspension in the following manner: by further diluting 10-fold in LB and by plating onto 1.6% LB agar plates and incubating overnight. The remaining suspensions (990 μL from each bacterial culture) were pooled into a sterile tube, then aliquoted into Eppendorf tubes in 500 μL volumes/tube. As a washing step, Eppendorf tubes were centrifuged at 14,000 rpm for 10 mins, 500 μL PBS was added to the pellet and subsequently resuspended. These tubes constituted the mock community samples and were stored at −80°C. Expected proportions of the mock community members were determined from the estimated CFUs multiplied by the genome size and were as follows: 8.7:13.0:7.7:16.7:38.9:14.5:0.4 for *S. aureus, B. subtilis, E. faecium, S. epidermidis, P. aeruginosa, Enterobacter cloacae*,and*E. coli*, respectively.

The 2 probiotic formulations used in this study were used as 2 additional positive controls. D-Scour™ is a commercially available probiotic formulation for livestock, with each 1 g containing 180 million CFUs of the following: *Lactobacillus acidophilus, Lactobacillus delbrueckii* subspecies *bulgaricus, Lactobacillus plantarum, Lactobacillus rhamnosus, Bifidobacterium bifidum, E. faecium*, and*Streptococcus salivarius* subspecies *thermophilus*, with an additional 20 mg of garlic extract (*Allium sativum*). The probiotic ColiGuard is a probiotic formulation developed for the treatment of entero-toxigenic *E. coli* in weaner pigs, developed in collaboration between the NSW DPI and International Animal Health Products, containing undefined concentrations of *L. plantarum* and *Lactobacillus salivarius*.

### DNA extraction

Piglet and sow faecal samples, mock community samples, negative controls, and probiotic samples (D-Scour™ and ColiGuard® paste) were allocated to a randomized block design to control for batch effects in DNA extraction and library preparation. The faecal samples were thawed on ice first, followed by the probiotics and mock community samples. MetaPolyzyme (Sigma-Aldrich, Castle Hill, NSW, Australia) treatment was performed according to the manufacturer's instructions except for the dilution factor, which we allowed to be 4.6 times higher. Immediately after incubation, DNA extraction was performed with the MagAttract PowerMicrobiome DNA/RNA EP kit (Qiagen, Chadstone Centre, Victoria, Australia) according to the manufacturer's instructions. Quantification of DNA was performed using PicoGreen (Thermofisher, Australia) and measurements were performed with a plate reader (Tecan, Life Sciences) using 50 and 80 gain settings. All samples were diluted to 10 ng/µL.

### Library preparation

Sample index barcode design using a previously introduced method [5] yielded a set of 96 × 8nt sequences with a 0.5 mean GC content and none of the barcodes containing 3 or more identical bases in a row. A total of 960 different combinations of i5 and i7 primers were used to create a uniquely barcoded library for each sample. The detailed sample-to-barcode assignment is given in Supplementary Table 2. Library preparation was carried out using a modification of the Nextera Flex protocol to produce low bias, called Hackflex, that allows the production of low-cost shotgun libraries [5]. For each sample, 10 ng of input genomic DNA in 10 μL ultrapure water (Invitrogen, Thermofisher Australia) was mixed with 10 μL of 1:50 diluted BLT beads, 25 μL of 2× laboratory-made tagmentation buffer 20 mM Tris (pH 7.6) (Chem-Supply), 20 mM MgCl (Sigma-Aldrich, Castle Hill, NSW, Australia), and 50% (v/v) dimethylformamide (Sigma-Aldrich, Castle Hill, NSW, Australia); the final volume for each tagmentation reaction was 45 μL. Following, 10 μL of 0.2% of sodium dodecyl sulphate (Sigma-Aldrich, Castle Hill, NSW, Australia) was added to each sample to stop tagmentation. Beads were then washed 3 times using 100 μL of washing solution, which was filtered prior to use (0.22 μm MF-Millipore™ membrane). The washing solution consisted of 10% polyethylene glycol 8000 (SigmaSigma-Aldrich, Castle Hill, NSW, Australia), 0.25M NaCl (Chem-Supply) in Tris-EDTA buffer (TE) (Sigma-Aldrich, Castle Hill, NSW, Australia). Library amplification was carried out using the PrimeSTAR GXL DNA Polymerase kit (Takara), according to the manufacturer protocol. Each PCR reaction contained 10 μL of 5× GXL buffer, 4 μL of 25 mM deoxynucleotide triphosphates, 2 μL of PrimeStar GXL polymerase, and 19 μL of nuclease-free water. The PCR mix was added into washed BLT beads. Then, 5 μL of each custom-synthesized 96-well plate Illumina Adapter Oligos i5 and i7 (i7: IDT plate No.: 11680765; i5: IDT plate No.: 11680754) was added to a final concentration of 0.555 μM to each reaction. Each sample's PCR reaction had a final volume of 45 μL. The following conditions were used: 3 min at 68°C; 3 min at 98°C; 12 cycles of the following 3 steps: 45 sec at 98°C, 30 sec at 62°C, 2 min at 68°C; 1 min at 68°C; and hold at 10°C. Following the amplification step, samples were centrifuged at 280*g* for 1 min and stored for 1–5 days at 4°C.

### Size selection and purification

Samples from the same 96-well plates were pooled into 1 tube by taking 5 μL from each library. This generated 10 pooled samples, 1 for each plate. A master pool was created by pooling 5 μL from the pool of each plate into a single pool. Forty microliters from each of the 10 plate pools and 40 μL from the master pool underwent library size selection and purification using equal volumes of SPRIselect beads (Beckman Coulter, USA) and ultrapure water (Invitrogen, Thermofisher Australia). Sample cleaning with SPRI-beads was performed as described previously [5]. A purified master pool comprising samples from all plates, and purified pools of individual plates to check for plate-specific anomalies, were diluted to 4 nM and fragment size distribution was assessed using the High Sensitivity DNA kit on the Bioanalyzer (Agilent Technologies, USA).

### Normalization and sequencing

The master pool was sequenced on an Illumina MiSeq v2 300 cycle nano flow cell (Illumina, USA). Read counts were obtained and used to normalize libraries. The liquid-handling robot OT-One (Opentrons) was programmed to re-pool libraries on the basis of read counts obtained from the previous MiSeq run. The code used to achieve the normalization is available through our Github repository.

The read count distribution after normalization is displayed in Supplementary Fig. 2. The normalized and purified pooled library was sequenced on an Illumina NovaSeq 6000 S4 flow cell at the Ramaciotti Centre for Genomics (Sydney, NSW, Australia), generating a total of 27 billion read pairs from 911 samples.

### Sequence data processing

Adapter trimming (parameters: k = 23 hdist = 1 tpe tbo mink = 11), PhiX DNA removal (parameters: k = 31 hdist = 1), and quality filtering (parameters: ftm = 0 qtrim = r trimq = 20) were performed using bbduk.sh [6] (bbmap version 38.22). Piglet samples (*n* = 825) had a median count of 32,949,208 clean paired reads (mean = 35,557,149) (script: readcounts.R). Quality assessment of raw reads was carried out using FastQC [7] and a combined report of all samples was obtained with MultiQC [8]. The presence of PCR duplicates was assessed by feeding read pairs to dedupe.sh [6] (parameters: ac = f). Nextflow [9] (version 18.10.1) was used to manage processing of the data on the HPC.

### Comparison of the expected and the observed taxonomic profile of the positive controls

All the mock community members, in 7 of the 8 technical replicates, were detected by MetaPhlAn2 (version 2.7.7) (Supplementary Fig. 3). One sample failed to sequence, reporting zero counts for any species. The observed mean ± SD relative abundances were as follows: *B. subtilis* (2.92 ± 0.994), *E. cloacae* (38.0 ± 6.404), *E. faecium* (0.97 ± 0.081), *E. coli* (10.12 ± 1.480), *E. coli* unclassified (7.83 ± 1.755), *P. aeruginosa* (26.72 ± 3.026), *S. aureus* (9.90 ± 3.613), and *S. epidermidis* (3.54 ± 1.435). Isolate *E. cloacae* C15117, used in this study for the make-up of the mock community, was recently found to be most closely related to the *E. hormaechei* phylogenomic group C type strain DSM 16687 and therefore re-identified as *E. hormaechei* subsp*. oharae* [10]. For this reason, taxonomic assignment by MetaPhlAn2 attributed the reads to *E. cloacae* instead. The expected proportions of the mock community members were derived from the CFUs by the genome size. On the basis of the expected (exp) and the observed (obs) relative abundance, we found, with the exception of *S. aureus* (exp: 8.7%, obs: 9.9%), 3 gram-positive members to be underrepresented (*B. subtilis*: exp: 13.0%, obs: 2.9%; *E. faecium:* exp: 7.7%, obs: 1.0%; *S. epidermidis*: exp: 16.7%, obs: 3.5%) and, with the exception of *P. aeruginosa* (exp: 38.9%, obs: 26.8%), 2 gram-negative members to be overrepresented (*E. cloacae:* exp: 14.5%, obs: 38.0%; *E. coli*: exp: 0.4%, obs: 7.8–10.1%) (Supplementary Fig. 4). Taxonomic assignment of the mock community samples reported 1 contaminating species in 1 of the 8 replicates: *L. salivarius* (mean: 0.008) (Supplementary Fig. 5).

The probiotic D-Scour™ is expected to contain, per 1 g, a total of 180 million CFU of *L. acidophilus, L. delbrueckii* subspecies *bulgaricus, L. plantarum, L. rhamnosus, B. bifidum, E. faecium*, and *S. salivarius* subspecies *thermophilus* in unknown proportions. From taxonomic analysis with MetaPhlAn2, we can conclude that 6 of the 7 expected species were determined to be present in the replicates in the following mean ± SD relative abundances: *B. bifidum*: 40.01 ± 12.558;  *E. faecium*: 30.98 ± 13.472;  *L. delbrueckii*: 11.56 ± 7.148;  *L. plantarum*: 6.23 ± 7.863;  *L. rhamnosus*: 2.08 ± 1.226;  *Streptococcus thermophilus*: 4.28 ± 1.523. *Lactobacillus acidophilus* was not detected and *Lactobacillus helveticus* was detected instead (*L. helveticus*: 4.75 ± 2.431) (Supplementary Fig. 3). An additional 25 taxa were detected, of which 18 and 7 were identified at the species and at the genus level, respectively. Contaminants were present at a higher concentration in 3 technical replicates (R3, R7, R8), with the most frequent contaminant (*Methanobrevibacter* spp.) being present in 5 of the 8 replicates (Supplementary Fig. 5).

Taxonomic analysis of the technical replicates of the probiotic ColiGuard® also showed a species profile consistent with the expected profile, with *L. salivarius* and *L. plantarum* in a 9:1 ratio (*L. salivarius*: mean ± SD: 93.52 ± 1.617;  *L. plantarum*: mean ± SD: 6.10 ± 1.134) across the replicates (Supplementary Fig. 3). ColiGuard® contained a total of 20 contaminants, of which 16 and 4 were identified at the species and the genus level, respectively. Contaminants were present at a higher level in 2 technical replicates (R5, R7), with R7 displaying the most diverse and highest contamination rate (R7: 14 taxa; total contaminating reads: 2.67%; R5: 9 taxa; total contaminating reads: 0.30%) (Supplementary Fig. 5).

### Technical controls in metagenomic studies and methodological limitations

Taxonomic assignment of the raw reads from the positive controls was performed with MetaPhlAn2 [11], which relies on ∼1M unique clade-specific markers derived from 17,000 reference genomes. Such a database to map against the positive controls suffices because these organisms are cultivable, and for this reason they are widely studied hence the sequences are known. This is not the case for real-world samples, where mapping against a database (the completeness of which relies on studied and often cultivable organisms) would narrow the view on the true diversity within the sample.

Positive controls with well-studied members and known ratios within the samples have proven to be a valuable approach to assess consistency among technical replicates across batches and to detect possible biases derived from the DNA extraction method. Systematic taxonomic bias in microbiome studies, resulting from differences in cell wall structures between gram-positive and gram-negative bacteria, have previously been reported; bead beating and sample treatment with enzymatic cocktails can modestly reduce this bias [12–15]. Although we implemented such steps in our workflow, it seems that, from the read abundance of our mock community, which contained 3 gram-negative and 4 gram-positive strains, a bias towards gram-negative taxa may still be present. However, this cannot be conclusively determined by our study because the expected amount of cells was derived from the CFUs method, which does not account for dead cells. Those cells would nonetheless contribute genomic DNA to the sample [16–19]. Knudsen *et al*. [15] compared various DNA isolation methods with distinct sample types and reported a reduced bias when using an adapted version of the QIAamp Fast DNA Stool Mini Kit (Qiagen, Chadstone Centre, Victoria, Australia) [15].

In terms of contamination we concluded that (i) contamination in our study was not batch specific and (ii) a problem of sample cross-contamination may have occurred at the DNA extraction step between neighbouring wells. During the bead-beating step of DNA extraction, the deep-well plate is sealed with a rubber sealing mat, rotated, and placed in a plate shaker for the bead beating to take place. Because leakage was observed around the wells despite the presence of the sealing mat, we consider that sample cross-contamination is most likely to occur during this step.

### Taxonomic profiling of samples

All raw reads were analysed with SortMeRNA [1] (version 4.0.0) to extract reads containing 16S rRNA genes. Extraction was performed by mapping reads against the silva-bac-16s-id90.fasta database with –fastx –blast 1 –num_alignments 1 parameters settings (script: sortmerna.sh). More than 60 million reads (*n* = 60,584,650) contained 16S rRNA genes, passing the E-value threshold for filtering (E-value ≤ 0.0001). These reads occupy between 36.4% and 37.1% of each sample (script: sortmerna_counts.R). Reads were further filtered based on E-value cut-off (E-value ≤ 1 × 10^−30^), sequence identity (identity ≥ 80%), and alignment length (length ≥ 100 bp). More than half of the reads (*n* = 32,419,310) passed the filtering (script: sortmerna_filter.sh) and were classified using the RDP classifier [2] (version 2.13), a naive Bayesian classifier that classifies 16S rRNA sequences into the new higher-order taxonomy proposed by Garrity *et al*. [20]) (script: RDP_Krona.sh). The most abundant phyla in the piglet population (*n* = 126) were *Firmicutes* (75.14%), *Bacteroidetes* (13.70%), *Actinobacteria* (5.31%), *Proteobacteria* (3.17%), *Spirochaetes* (0.72%), and*Synergistetes* (0.40%). The most abundant phyla in the mothers (*n* = 42) were *Firmicutes* (84.75%), *Proteobacteria* (6.30%), *Bacteroidetes* (5.44%), *Actinobacteria* (1.85%), *Verrucomicrobia* (0.36%), and *Synergistetes* (0.23%). The RDP classifier estimates the confidence of an assignment using the number of times a genus is selected out of 100 bootstrap trials. Assignments at the phylum level had a mean confidence of 0.93 (scale 0–1; median = 1.00) (script: RDP_analyze.R). Classifications were displayed using Krona [3] (version 2.7.1).

### α- and β-diversity

The abundance profile of all samples, based on the 16S rRNA reads that passed filtering (E-value ≤ 1 × 10^−30^; identity ≥ 80%; length ≥ 100 bp; *n* = 32,419,310; script: sortmerna_filter.sh), was used to estimate α- and β-diversity with phyloseq [21] (version 1.28.0) (script: sortmerna_diversity_giga.R). Samples with <10,000 read counts were excluded.

For α-diversity, library normalization was obtained by rarefaction and the Chao1, Shannon, and Simpson diversity indices were obtained. Confidence intervals (CI) for differences between time points were obtained using the R package pairwiseCI (v0.1–27). Sample diversity estimates were compared between time points using the *t*-test and adjusting significance with the Bonferroni method. The α-diversity decreased in terms of species richness and evenness between the first week (t0) and the fifth week after weaning (t10) (Shannon diversity estimate = −0.18, se = 0.10, *P* = 0.0078). The mothers had a higher species richness compared to the piglets in the first week after weaning (t0; Chao1 estimate = 162.28, se = 64.20, *P* < 0.0001) and in the fifth week after weaning (t10; Chao1 estimate = 146.20, se = 59.64, *P* = 0.0001).

For β-diversity, library normalization was obtained by rarefaction. A principal coordinate analysis was performed. Multivariate analysis of variance (ANOVA) was used to compare groups, and *t*-tests were used for pairwise comparisons, adjusting the significance with the Bonferroni method. Time points (piglet age) were found to be predictive of variance in microbial gut communities (F = 294.6, *P* < 0.001). Samples from the piglets (all time points) and the mothers significantly separated in β-diversity (F = 7.692; *P* = 0.00572).

### Potential uses

This dataset can be utilized to assess a broad range of ecological questions pertaining to host-associated microbial communities of the post-weaning piglet. These include the assessment of (i) the compositional and functional core faecal microbiome of the post-weaning piglet, (ii) the microbial changes that piglets undergo between the first and the fifth week after weaning, (iii) the degree of strain-host specificity, (iv) the variability of microbiomes within or between host species, (v) the variability of microbiomes between different cross-breeds and small age differences of the hosts, (vi) the degree of strain transfer from mothers to piglets, (vii) the effects of 2 probiotic treatments and of intramuscular antibiotic treatment on the post-weaning pig faecal microbiome, (viii) species co-occurrence and co-exclusion, (ix) the repertoire of antimicrobial resistance genes and how it is affected by antibiotic and probiotic treatment, and (x) the extent of within-host and population evolution of microbes over a 5-week period.

## Data Availability

The sequencing reads from each sequencing library have been deposited at NCBI Short Read Archive under project PRJNA526405. All supplementary figures and tables are provided as additional files. The scripts for the automated robot pooling (robot_pooling.py), for the sequence data processing (initial.nf), and for the data analysis can be found in our Github repository ([4]; tag: GigaScience). For the data analysis scripts, the R language (version 3.6.3) and the following packages were used: readr (v1.4), readxl (v1.3.1), tidyr (v1.1.2), tidyverse (v1.3.0), ggplot2 (v3.3.3), dplyr (v1.0.3), gridExtra (v2.3), pheatmap (v1.0.12), cowplot (v1.1.1), splitstackshape (v1.4.8), pairwiseCI (v0.1–27).

The full dataset, snapshots of our code, and other data further supporting this work are openly available in the *GigaScience* repository, GigaDB http://doi.org/10.5524/100890 [22].

## Additional Files

**Supplementary Figure 1**: Piglets' placements across rooms and pens.

**Supplementary Figure 2**: Read count distribution.

**Supplementary Figure 3**: Taxonomic assignment of reads from positive control samples.

**Supplementary Figure 4**: Expected and observed relative abundance of mock community members.

**Supplementary Figure 5**: Heat map reporting the contaminating species found within the technical replicates of the positive controls.

**Supplementary Table 1**: Metadata.

**Supplementary Table 2**: Barcodes.

## Abbreviations

bp: base pairs; CFU: colony-forming unit; EMAI: Elizabeth Macarthur Agricultural Institute; LB: Luria-Bertani; NCBI: National Center for Biotechnology Information; PBS: phosphate-buffered saline; rRNA: ribosomal RNA.

## Funding

This work was supported by the Australian Research Council, linkage grant LP150100912. This project was funded by the Australian Centre for Genomic Epidemiological Microbiology (Ausgem), a collaborative partnership between the NSW Department of Primary Industries and the University of Technology Sydney. T.Z. and D.G. are recipients of University of Technology Sydney (UTS) International Research and UTS President's Scholarships. NSW DPI approved the manuscript before submission for publication.

## Competing Interests

D-Scour™ was sourced from International Animal Health Products (IAHP). ColiGuard® was developed in a research project with NSW DPI, IAHP and AusIndustry Commonwealth government funding.

## Authors' Contributions

Pig trial: T.A.C., L.F., D.G., T.Z., G.J.E., A.E.D., S.P.D.

DNA extraction: D.G., M.L.

Library preparation, robot pooling: D.G., M.L., K.A., A.E.D.

Sequencing data processing: M.Z.D., D.G., A.E.D.

Data analysis: D.G., A.E.D.

Manuscript writing: D.G.

Manuscript editing: D.G., A.E.D., G.J.E.

## Supplementary Material

giab039_GIGA-D-20-00347_Original_Submission

giab039_GIGA-D-20-00347_Revision_1

giab039_GIGA-D-20-00347_Revision_2

giab039_GIGA-D-20-00347_Revision_3

giab039_Response_to_Reviewer_Comments_Original_Submission

giab039_Response_to_Reviewer_Comments_Revision_1

giab039_Response_to_Reviewer_Comments_Revision_2

giab039_Reviewer_1_Report_Original_Submission Weilan Wang -- 12/11/2020 Reviewed

giab039_Reviewer_1_Report_Revision_1 Weilan Wang -- 2/25/2021 Reviewed

giab039_Reviewer_2_Report_Original_Submission Alex Bossers -- 1/6/2021 Reviewed

giab039_Reviewer_2_Report_Revision_1 Alex Bossers -- 3/22/2021 Reviewed

giab039_Supplemental_Files
