# Supplementary material for: A large-scale metagenomic survey dataset of the post-weaning piglet gut lumen
Source: Gigascience. 2021 Jun 3;10(6):giab039. doi: 10.1093/gigascience/giab039 (PMC8173662; doi:10.1093/gigascience/giab039)
Supplement: giab039_GIGA-D-20-00347_Original_Submission [file giab039_giga-d-20-00347_original_submission.pdf]

GigaScience

Community composition and development of the post-weaning piglet gut microbiome

--Manuscript Draft--

**Manuscript Number:**	GIGA-D-20-00347
**Full Title:**	Community composition and development of the post-weaning piglet gut microbiome
**Article Type:**	Research
**Funding Information:**	
**Abstract:**	We report on the largest metagenomic analysis of the pig gut microbiome to date. By processing over 800 faecal time-series samples from 126 piglets and 42 sows, we generated over 8Tbp of metagenomic shotgun sequence data. Here we describe the animal trial procedures, the generation of our metagenomic dataset and the analysis of the microbial community composition using a phylogenetic framework. We assess the effects of intramuscular antibiotic treatment and probiotic oral treatment on the diversity of gut microbial communities. We found differences between individual hosts such as breed, litter, and age, to be important contributors to variation in the community composition. Treatment effects of the antibiotic and probiotic treatments were found but were subtle, while host age was the dominant factor in shaping the gut microbiota of piglets after weaning. The post-weaning pig gut microbiome appears to follow a highly structured developmental program with characteristic post-weaning changes that can distinguish hosts that were born as little as two days apart in the second month of life.
**Corresponding Author:**	Aaron Darling<br><br>AUSTRALIA
**Corresponding Author Secondary Information:**	
**Corresponding Author's Institution:**	
**Corresponding Author's Secondary Institution:**	
**First Author:**	Daniela Gaio
**First Author Secondary Information:**	
**Order of Authors:**	Daniela Gaio
	Matthew Z DeMaere
	Kay Anantanawat
	Graeme J Eamens
	Michael Liu
	Tiziana Zingali
	Linda Falconer
	Toni A Chapman
	Steven P Djordjevic
	Aaron E Darling
**Order of Authors Secondary Information:**	
**Additional Information:**	
**Question**	**Response**
Are you submitting this manuscript to a special series or article collection?	No

Powered by Editorial Manager® and ProduXion Manager® from Aries Systems Corporation

**Experimental design and statistics**<br><br>Full details of the experimental design and statistical methods used should be given in the Methods section, as detailed in our Minimum Standards Reporting Checklist. Information essential to interpreting the data presented should be made available in the figure legends.<br><br>Have you included all the information requested in your manuscript?	Yes
**Resources**<br><br>A description of all resources used, including antibodies, cell lines, animals and software tools, with enough information to allow them to be uniquely identified, should be included in the Methods section. Authors are strongly encouraged to cite Research Resource Identifiers (RRIDs) for antibodies, model organisms and tools, where possible.<br><br>Have you included the information requested as detailed in our Minimum Standards Reporting Checklist?	Yes
**Availability of data and materials**<br><br>All datasets and code on which the conclusions of the paper rely must be either included in your submission or deposited in publicly available repositories (where available and ethically appropriate), referencing such data using a unique identifier in the references and in the "Availability of Data and Materials" section of your manuscript.<br><br>Have you have met the above requirement as detailed in our Minimum Standards Reporting Checklist?	Yes

Powered by Editorial Manager® and ProduXion Manager® from Aries Systems Corporation

Click here to access/download;Manuscript;msfile-1.pdf

Community composition and development of the post-weaning piglet gut microbiome

D. Gaio[1], Matthew Z. DeMaere[1], Kay Anantanawat[1], Graeme J. Eamens[2], Michael Liu[1], Tiziana Zingali[1], Linda Falconer[2], Toni A. Chapman[2], Steven P. Djordjevic[1], Aaron E. Darling[1]*

[1]The ithree institute, University of Technology Sydney, Sydney, NSW, Australia

[2]NSW Department of Primary Industries, Elizabeth Macarthur Agricultural Institute, Menangle, NSW, Australia

*Corresponding author: Aaron E. Darling, aaron.darling@uts.edu.au

Keywords: shotgun metagenomics; pig; post-weaning; gut microbiome; phylogenetic diversity

ABSTRACT

We report on the largest metagenomic analysis of the pig gut microbiome to date. By processing over 800 faecal time-series samples from 126 piglets and 42 sows, we generated over 8Tbp of metagenomic shotgun sequence data. Here we describe the animal trial procedures, the generation of our metagenomic dataset and the analysis of the microbial community composition using a phylogenetic framework. We assess the effects of intramuscular antibiotic treatment and probiotic oral treatment on the diversity of gut microbial communities. We found differences between individual hosts such as breed, litter, and age, to be important contributors to variation in the community composition. Treatment effects of the antibiotic and probiotic treatments were found but were subtle, while host age was the dominant factor in shaping the gut microbiota of piglets after weaning. The post-weaning pig gut microbiome appears to follow a highly structured developmental program with characteristic post-weaning changes that can distinguish hosts that were born as little as two days apart in the second month of life.

INTRODUCTION

As the world population grows, there is an accompanying demand for animal derived products. In a semi-natural environment pig weaning occurs between the 12th and the 17th weeks from birth, whereas in the farm this typically occurs at 4 weeks of age[1]. Intensive animal husbandry and early weaning practices are commonly used to maximise production rates while minimizing costs. Both practices increase the risk of infections with pathogenic organisms, and thereby the need for antimicrobial strategies, which has included the common use of antibiotics. [2–12] Although antibiotics may kill some pathogens, the surviving bacteria can develop antimicrobial resistance (AMR) against the class of antibiotic used [13–20], as well as against other antibiotic classes [21–31]. A retrospective U.S. livestock study found evidence of multidrug resistance (resistance to >3 antimicrobial drug classes) in *Escherichia coli* that increased from 7.2% to 63.6% between the 1950s and the 2000s [32]. As the incidence of multidrug resistant (MDR) pathogens expands, the World Health Organization (WHO) has recognized AMR as one of the top health challenges of the current century [33,34].

Besides leading to AMR development, antibiotic usage is known to cause dysbiosis [34–37], the disruption of a balanced state within a gut microbial community. A balanced state decreases the chance of pathogens gaining a foothold, as there is niche and nutrient competition in play [39,40]. In livestock production, intramuscular (IM) antibiotic treatment is preferred over oral antibiotic treatment, as it is thought to contribute less to AMR than oral antibiotic treatment [41–43]. Extensive evidence exists on dysbiosis as a consequence of oral antibiotic treatment on the gut microbiome [22,35–38,44,45], whereas only a handful of studies report the effects of intramuscular (IM) antibiotic treatment on the gut microbial community [46–48], and none, to our knowledge have studied the use of IM neomycin treatment.

A number of non-antibiotic strategies to increase resistance to disease have been studied [49]. Strategies for which beneficial effects have been reported consist of hydrolases [50], fiber intake [51], β-lactamase enzymes [52], non-resistance inducing antimicrobial drugs [53,54], vaccination [55–58], phage therapy [59], in-feed organic acids [60], starches [61], and liquid feed [62–64]. Extensive evidence exists on the use of probiotics. Probiotics are reported to promote intestinal health in multiple ways: improvement of mucosal integrity [65–68], competitive exclusion against pathogenic species [50,69–73], reduction of intestinal inflammation [65,74,75] and of pathogen translocation [65,76,77]. Their efficacy has been determined, among others, in cell cultures [70,74,75,78], mice [50,67,68,73], and swine [65,66,74,76,79–83]. However, while most evidence of beneficial effects from probiotic treatment is derived from host immunity responses [65–68,74–

[76,80], host physiology [74,80–82], and pathogen relocation [50,65,69,70,72,77–79,84], fewer studies exist on the effects of probiotics on the whole gut microbial community [83,85].

This study was conducted to: 1) generate a public metagenomic databank of the gut microbiome of weaner piglets aged between 3 and 9 weeks old; 2) assess the effects of IM neomycin antibiotic use on the gut community; and 3) assess the effects of two probiotic formulations on the gut community. The data we present here is analysed from the perspective of the phylogenetic diversity of the microbial communities.

In the first results section we describe the phylogenetic diversity of the intestinal microbiome of the piglets and how it compares to the known composition of positive controls. Then, we describe evidence that highlights the importance of age, breed and litter as strongly associated factors with changes in community composition. Lastly, we describe the strong effect of time on community composition and the milder effect of antibiotic and probiotic treatments on community composition.

METHODS

Pig trial

Animal studies were conducted at the Elizabeth Macarthur Agricultural Institute (EMAI) NSW, Australia and were approved by the EMAI Ethics Committee (Approval M16/04). The trial animals comprised 4-week old male weaner pigs (*n*=126) derived from a commercial swine farm and transferred to the study facility in January 2017. These were cross-bred animals of Landrace, Duroc and Large White breeds and had been weaned at approximately 3 weeks of age.

The pig facility consisted of four environmentally controlled rooms (Rooms 1-4) with air conditioning, concrete slatted block flooring with underground drainage and open rung steel pens (**S1**). Each room had nine pens, consisting of a set of six and a set of three pens, designated a-f and g-i respectively, with the two sets of pens being physically separate, *i.e.* animals could come in contact with each other through the pen's bars within each set of pens, but not between sets. The rooms were physically separated by concrete walls and contamination between rooms was minimized by using separate equipment (boots, gloves, coveralls) for each room. In addition, under-floor drainage was flushed twice weekly and the

flushed faeces/urine was retained in under-floor channels that ran the length of the facility, so that Rooms 1, 2 were separate from Rooms 3, 4 and flushing was in the direction 1 to 2 and 3 to 4.

The pigs were fed *ad libitum* a commercial pig grower mix of 17.95% protein free of antibiotics, via self-feeders. On the day of arrival (day 1) 30, 18, 18, and 60 pigs were allocated randomly to Rooms 1, 2, 3 and 4 respectively in groups of 6, 6, 6 and 6-7 pigs per pen respectively (**S1A**). Pigs were initially weighed on day 2, and some pigs were moved between pens to achieve an initial mean pig weight per treatment of approximately 6.5 kg (range: 6.48-6.70; mean±SD: 6.53±0.08). Pigs were weighed weekly throughout the trial (**Supplementary file 1**). Behaviour and faecal consistency scores were taken daily over the 6-week period of the trial (**Supplementary file 2**). Developmental and commercial probiotic paste preparations ColiGuard® and D-Scour™ from International Animal Health, Australasia, were used in some treatment groups.

The animals were acclimatised for 2 days before the following treatments were administered: Room 1 - oral 1 g/pig of placebo paste daily for 14 d; Room 2 - oral 1 g/pig of D-Scour™ paste daily for 14 d; Room 3 - oral 1 g/pig of ColiGuard® paste daily for 14 d; Room 4 - intramuscular (IM) injection of antibiotic administered at 0.1 mL per pig daily from a 200 mg/mL solution for a total treatment duration of 5 d.

On the day following the final neomycin treatment (day 8), 36 pigs were moved from Room 4 to Room 2 (*n*=18, 6 in each pen, pens g-i), and to Room 3 (*n*=18, 6 in each pen, pens g-i) (**S1**). The following day (day 9), oral administration of D-Scour™ (1 g/pig) and of ColiGuard® (1 g/pig) commenced for pigs in Room 2 pens g-i and in Room 3 pens g-i, respectively, and continued for a period of 14 days. Assignment of the 36 neomycin-treated pigs to the treatment groups neomycin+D-Scour™ (*n*=18; Room 2 pens g-i) and neomycin+ColiGuard® (*n*=18; Room 3 pens g-i), was carried out by distributing them so that the mean weight of the animals distributed across pens and rooms was similar (**Supplementary file 1**). By this time point, each occupied pen in the trial housed six pigs. (**S1B**) From that time, twelve piglets from the original 126 were no longer present, as they had been euthanised as pre-treatment controls at the start of the trial.

Faecal samples were collected from all piglets once per week and from a subset (*n*=48 pigs; 8 from each of the six cohorts) twice per week over the 6-week study period (**Figure**

1). From each piglet, faeces were collected per rectum with new disposable gloves; where minimal or no faeces could be collected on a collection day, sampling was performed the following morning. Samples were placed in 50 mL Falcon tubes and stored at 4ºC within 30 mins of sample collection for a minimum of 30 mins and a maximum period of 6 h.

Faecal sample processing

Samples (3g/pig) were mixed with 15 mL PBS (200 mg/mL), in sterile stomacher bags and homogenized with a Bio-Rad stomacher. The homogenised samples were divided in replicates: one replicate was stored directly at -80ºC and one replicate was supplemented with glycerol (20% v/v) (Sigma-Aldrich) then stored at -80º C. In addition, single time-point faecal samples from the dams of the trial pigs (*n*=42) were obtained from the commercial facility of origin and were pre-processed at EMAI as described above. Thus, a total of 911 unique samples, between one and ten samples per subject (mean: 4.8; median: 3) (**S2**), were obtained throughout this study. At the end of the trial period, all samples were transported from EMAI to the University of Technology Sydney (UTS) for further processing.

Positive controls

As a positive control "mock community" for this study, four Gram positive (*Bacillus subtilis* strain 168, *Enterococcus faecium*, *Staphylococcus aureus* ATCC25923, *Staphylococcus epidermidis* ATCC35983) and three Gram negative (*Enterobacter hormaechei* CP_032842, *Escherchia coli* K-12 MG1655, *Pseudomonas aeruginosa* PAO1) bacterial strains from -80°C stocks were cultured at 37ºC for 16 h in LB (Luria-Bertani) then centrifuged at 14,000 rpm for 10 mins. From the resulting pellets, 1 g was transferred to 1 mL of LB and homogenised and a 1:10 dilution of this was made for each bacterial culture. Ten microliters of bacterial suspension from each of the cultures was used to determine the number of colony forming units (CFU) in the original suspension in the following manner: by further diluting tenfold in LB and by plating onto 1.6% LB agar plates and incubacted overnight. The remaining suspensions (990 μL from each bacterial culture) were pooled into a sterile tube, then aliquoted into Eppendorf tubes in 500 μL volumes/tube. As a washing step, Eppendorf tubes were centrifuged at 14,000 rpm for 10 mins, 500 μL PBS was added to the pellet and subsequently resuspended. These tubes constituted the mock community samples and were stored at -80ºC. Expected proportions of the mock community members were determined from the estimated colony forming units (CFU) multiplied by the genome

size and were as follows: 8.7:13.0:7.7:16.7:38.9:14.5:0.4 for *S. aureus*, *B. subtilis*, *E. faecium, S. epidermidis*, *P. aeruginosa*, *E. cloacae, and E.coli* respectively.

The two probiotic formulations used in this study were used as two additional positive controls. D-Scour™ is a commercially available probiotic formulation for livestock, with each gram containing 180 million CFU of the following: *Lactobacillus acidophilus*, *Lactobacillus delbrueckii* subspecies *bulgaricus*, *Lactobacillus plantarum*, *Lactobacillus rhamnosus*, *Bifidobacterium bifidum*, *Enterococcus faecium*, *Streptococcus salivarius* subspecies *thermophilus*, with an additional 20 mg of garlic extract (*Allium sativum*). The probiotic ColiGuard is a probiotic formulation developed for the treatment of entero-toxigenic *Escherichia coli* (ETEC) in weaner pigs, developed in collaboration between the NSW DPI and International Animal Health Products, containing undefined concentrations of *Lactobacillus plantarum* and *Lactobacillus salivarius*.

DNA extraction

Piglet and sow faecal samples, mock community samples, negative controls and probiotic samples (D-Scour™ and ColiGuard® paste) were allocated to a randomized block design to control for batch effects in DNA extraction and library preparation. The faecal samples were thawed on ice first, followed by the probiotics and mock community samples. MetaPolyzyme (Sigma-Aldrich) treatment was performed according to the manufacturer's instructions except for the dilution factor, which we allowed to be 4.6 times higher. Immediately after incubation, DNA extraction was performed with the MagAttract PowerMicrobiome DNA/RNA EP kit (Qiagen) according to the manufacturer's instructions. Quantification of DNA was performed using PicoGreen (Thermofisher) and measurements were performed with a plate reader (Tecan, Life Sciences) using 50 and 80 gain settings. All samples were diluted to 10 ng/μL.

Library preparation

Sample index barcode design using a previously introduced method [86] yielded a set of 96 x 8nt sequences with a 0.5 mean GC content and none of the barcodes containing 3 or more identical bases in a row. Nine hundred-sixty different combinations of i5 and i7 primers were used to create a uniquely barcoded library for each sample. The detailed sample-to-barcode assignment is given in **Supplementary file 3**. Library preparation was carried out using a modification of the Nextera Flex protocol to produce low bias, low cost shotgun libraries, as

described in a previous manuscript [86]. Following the amplification step, samples were centrifuged at 280 x *g* for 1 min and stored between 1 and 5 days at 4°C.

Size selection and purification

Samples from the same 96-well plates were pooled into one tube by taking 5 μL from each library. This generated 10 pooled samples, one for each plate. A master pool was created by pooling 5 μL from the pool of each plate into a single pool. Forty microliters from each of the 10 plate pools and 40 μL from the master pool underwent library size selection and purification using equal volumes of SPRIselect beads (Beckman Coulter) and ultrapure water (Invitrogen). Sample cleaning with SPRI-beads was performed as described previously [86]. A purified master pool comprising samples from all plates, and purified pools of individual plates to check for plate-specific anomalies, were diluted to 4 nM and fragment size distribution was assessed using the High Sensitivity DNA kit on the Bioanalyzer (Agilent Technologies, USA).

Normalisation and sequencing

The master pool was sequenced on an Illumina MiSeq v2 300 cycle nano flow cell (Illumina, USA). Read counts were obtained and used to normalise libraries. The liquid handling robot OT-One (Opentrons) was programmed to re-pool libraries based on read counts obtained from the previous MiSeq run. The script used to achieve the normalization is available through our Github repository.

The read count distribution after normalisation is displayed in supplementary figure (**S3**). The normalized and purified pooled library was sequenced on an Illumina NovaSeq S4 flow cell at the Ramaciotti Centre for Genomics (Sydney, NSW, Australia), generating a total of 27 billion read pairs from 911 samples.

Sequence data processing

Adapter trimming (parameters: k=23 hdist=1 tpe tbo mink=11), PhiX DNA removal (parameters: k=31 hdist=1), and quality filtering (parameters: ftm=0 qtrim=r trimq=20), were performed using bbduk.sh (http://jgi.doe.gov/data-and-tools/bbtools). Quality assessment of raw reads was carried out using FASTQC (http://www.bioinformatics.babraham.ac.uk/projects/fastqc/) and a combined report of all samples was obtained with MULTIQC [87] (**Supplementary File 4**). The presence of PCR

duplicates was assessed by feeding read pairs to dedupe.sh (http://jgi.doe.gov/data-and-tools/bbtools) (parameters: ac=f). Nextflow [88] was used to manage processing of the data on the HPC.

Determination of microbial diversity among samples

Phylogenetic diversity of all samples was assessed with PhyloSift [89] using the first 100k read pairs of each sample (parameters: --chunk-size 100000 --paired). In order to test for associations of phylogenetic diversity with treatment, time of sampling, and differences among hosts at the start of the trial, analysis of the unrooted phylogenetic diversity (PD) [90], the balance weighted phylogenetic diversity (BWPD) [91] and principal component analysis (PCA) of the Kantorovich-Rubinstein distances [92] (beta diversity analysis) were performed. Alpha-diversity and beta-diversity were analyzed and the results were visualized with R [93] and R packages [91–107]. The data analysis is schematically represented in **Supplementary Figure 4** (**S4**).

Additionally, SortmeRNA [111] (version 2.1) was applied to extract 16S ribosomal RNA genes from raw reads. The rRNA reference database silva-bac-16s-id90 was used. Hits were filtered (e-value <= 1e-30) and PCA was computed with R [93]. Sample counts were normalized for library size by proportions and were tested with the Spearman's Rank correlation coefficient method to find taxa correlating with the weight of the piglets across the trial.

*Batch effect*s

A randomized block design was adopted to mitigate batch effects. Because samples were distributed across ten 96-well plates during DNA extraction and library preparation, plate effects were expected. Although samples did not visibly cluster by DNA extraction plate across the first five principal components (**S5**), a batch effect was found by multiple comparison analysis with ANOVA and by applying Tukey post-hoc correction to pairwise comparisons. Batch effects were detected (ANOVA, alpha diversity: p range=4.8e-12-0.00011; beta diversity: p value<0.0023) (**S6**; **Supplementary file 5**) and removed with COMBAT[7] (ANOVA p value=1) (**S7**; **Supplementary file 5**).

Technical analysis of positive controls

As a quality control step, taxonomic analysis of the positive control samples and their technical replicates was performed with MetaPhlAn2 [112]. The positive controls included the mock community (*n*=8), D-Scour™ (*n*=8), and ColiGuard® (*n*=8) samples.

Data availability

The raw sequencing data has been submitted to the NCBI Short Read Archive under project PRJNA526405. Metadata and scripts are available in this article, its Supplementary material and in our Github repository https://github.com/GaioDany/metapigs_base

RESULTS

PhyloSift [89] was employed as a means to study microbial community diversity among the samples, and to test for associations with treatment, time of sampling, and differences among hosts at the start of the trial. To this end, analysis of the unrooted phylogenetic diversity (PD) [90], the balance weighted phylogenetic diversity (BWPD) [91] and principal component analysis (PCA) of the Kantorovich-Rubinstein distances [92] (beta diversity analysis) were performed.

Comparison of the expected and the observed taxonomic profile of the positive controls

All the mock community members, in seven of the eight technical replicates, were detected by MetaPhlAn2 (**S8**). One sample failed to sequence, reporting zero counts for any species. The observed mean relative abundances were as follows: *B. subtilis* (mean±SD: 2.92±0.994), *E. cloacae* (mean±SD: 38.0±6.404), *E. faecium* (mean±SD: 0.97±0.081), *E.coli* (mean±SD: 10.12±1.480), *E.coli* unclassified (mean±SD: 7.83± 1.755), *P. aeruginosa* (mean±SD: 26.72±3.026), *S. aureus* (mean±SD: 9.90±3.613), *S. epidermidis* (mean±SD: 3.54±1.435). Isolate *E. cloacae* C15117, used in this study for the make-up of the mock community, was recently found to be most closely related to the *Enterobacter hormaechei* phylogenomic group C type strain DSM 16687 and therefore re-identified as *Enterobacter hormaechei* subsp. *oharae*[113] . For this reason, taxonomic assignment by MetaPhlAn2 attributed the reads to *E. cloacae* instead. The expected proportions of the mock community members were derived from the CFU by the genome size. Based on the expected and the observed relative abundance, we found, with the exception of *S. aureus* (exp: 8.7% obs: 9.9%), three Gram positive members to be under-represented (*B. subtilis*: exp: 13.0% obs:

2.9%; *E.faecium:* exp: 7.7% obs: 1.0%; *S. epidermidis*: exp: 16.7% obs: 3.5%) and, with the exception of *P. aeruginosa* (exp: 38.9% obs: 26.8%), two Gram negative members to be over-represented (*E. cloacae:* exp: 14.5% obs: 38.0%; *E.coli*: exp: 0.4% obs: 7.8-10.1%) (**S9**). Taxonomic assignment of the mock community samples reported one contaminating species in one of the eight replicates: *Lactobacillus salivarius* (mean: 0.008) (**S10**).

The probiotic D-Scour™ is expected to contain, per gram, a total of 180 million CFU of *Lactobacillus acidophilus*, *Lactobacillus delbrueckii* subspecies *bulgaricus*, *Lactobacillus plantarum*, *Lactobacillus rhamnosus*, *Bifidobacterium bifidum*, *Enterococcus faecium*, *Streptococcus salivarius* subspecies *thermophilus* in unknown proportions. From taxonomic analysis with MetaPhlAn2, we can conclude that 6 of the 7 expected species were determined to be present in the replicates in the following mean relative abundances: *Bifidobacterium bifidum*: mean±SD: 40.01±12.558; *Enterococcus faecium*: mean±SD: 30.98±13.472; *Lactobacillus delbrueckii*: mean±SD: 11.56±7.148; *Lactobacillus plantarum*: mean±SD: 6.23±7.863; *Lactobacillus rhamnosus*: mean±SD: 2.08±1.226; *Streptococcus thermophilus*: mean±SD: 4.28±1.523. *Lactobacillus acidophilus* was not detected and *Lactobacillus helveticus* was detected instead (*Lactobacillus helveticus*: mean±SD: 4.75±2.431) (**S8**). An additional 25 taxa were detected, of which 18 and 7 were identified at the species and at the genus level, respectively. Contaminants were present at a higher concentration in three technical replicates (R3, R7, R8) with the most frequent contaminant (*Methanobrevibacter* spp.) being present in 5 of the 8 replicates (**S11**).

Taxonomic analysis of the technical replicates of the probiotic ColiGuard® also showed a species profile consistent with the expected profile, with *Lactobacillus salivarius* and *Lactobacillus plantarum* in a 9:1 ratio (*Lactobacillus salivarius*: mean±SD: 93.52±1.617; *Lactobacillus plantarum*: mean±SD: 6.10±1.134) across the replicates (**S8**). ColiGuard® contained a total of 20 contaminants, of which 16 and 4 were identified at the species and the genus level, respectively. Contaminants were present at a higher level in two technical replicates (R5, R7), with R7 displaying the most diverse and highest contamination rate (R7: 14 taxa; total contaminating reads: 2.67%; R5: 9 taxa; total contaminating reads: 0.30%). (**S12**).

Phylogenetic diversity of positive controls and how it compares to the taxonomic profile

Alpha diversity of the positive controls reflects the expected alpha diversity, with the mock community, ColiGuard® and D-Scour™ positive controls, displaying a progressively higher unrooted PD (mean±SD: Mock community: 31.53±29.50; ColiGuard®: 58.52±21.70; D-Scour™: 64.84±21.30). BWPD for the positive control ColiGuard® appears on the far left of the plot (mean±SD: 0.82±0.15), distant from the mock community (mean±SD: 1.58±0.12) and the positive control D-Scour™ (mean±SD: 1.89±0.26), which contains 2, 7 and 8 main species, respectively (**S13**; **Supplementary file 1**). The unrooted PD of the positive control ColiGuard® (mean±SD: 58.52±21.70) is closer to the unrooted PD of the Mock Community (mean±SD: 31.53±29.50) and to that of the positive control D-Scour™ (mean±SD: 64.84±21.30), than the BWPD.

The contribution to total diversity of phylogenetic tree edges with uneven quantities of reads placed on either side is down-weighted in BWPD, which is reflected in the low reported BWPD of the positive control ColiGuard®. ColiGuard® is mainly composed of two species in an uneven ratio (1:9) as by taxonomic analysis with MetaPhlAn2 (**S8**), in contrast with the mock community (0.01:1:3:4:8:10:10:27:38) and the positive control D-Scour™ (2.08:4.28:4.75:6.23:11.56:30.98:40.01) (**S8**). On the other hand, the unrooted PD reflects the absolute diversity, independently of the relative abundance of each species, within a sample. In fact, low-level contamination (<0.1%) detected in each of the positive controls (Mock Community: 1 taxon; ColiGuard®: 20 taxa; D-Scour™: 25 taxa) (**S11-13**) contributes toward the absolute diversity in the unrooted PD, erroneously inflating this value (mean±SD: Mock Community: 31.53±29.50; ColiGuard®: 58.52±21.70; D-Scour™: 64.84±21.30) (**Figure 2**).

Phylogenetic diversity of piglet gut communities immediately after weaning

The 126 piglets used in this study were derived from 4 main breed cross types "Duroc × Landrace" (n=46), "Duroc × Large White" (n=59), "Landrace × cross bred (LWxD)" (n=9), "Large White × Duroc" (n=12), and three pig lines (line 319: n=9; line 316: n=46; line 326: n=71). The piglets also differed slightly by age, being born between 1 and 6 days apart.

Based on Kruskal-Wallis one-way analysis of variance (Hommel adjusted p values to correct for multiple testing), phylogenetic diversity of the piglet samples did not cluster significantly by breed cross type ($p > 0.05$) or by pig line ($p > 0.05$) at the start of the trial, but a significant difference was found with breed and line in the fourth week of the trial

(breed: $p=0.04$; pig line: $p=0.02$) (**Figure 2**). Additionally a correlation of breed cross type was found with beta diversity principal components (PC) in the second week of the trial (PC3: $p=0.0357$) and in the fifth week of the trial (PC1: $p=0.027$). Alpha diversity significantly correlated with pig line in the fourth week of the trial (BWPD: $p=0.017$). (**Figure 2**; **Supplementary file 5**)

Notably, we found a significant correlation between alpha diversity and the age of the piglets at the first sampling time point (unrooted PD: $p=0.0016$; BWPD: $p=0.0355$) (**Figure 2**; **Supplementary file 5**). While a correlation with age was found for alpha diversity only at the start of the trial, age of the piglet was observed to be weakly associated with differences in community composition in week two (PC3: $p=0.0507$) (**S14**; **Supplementary file 5**).

As age groups were confounded with cross-breed types (*i.e.* not all age groups are represented by each of the four cross-breed types), we compared the phylogenetic diversity of age groups within each breed. As cross-breed types "Landrace × cross bred (LW × D)" and "Large White × Duroc" had only a small number of piglets in each age group, we tested for an association between phylogenetic diversity and age in cross-breeds "Duroc × Landrace" and "Duroc × Large White". In these cross-breeds, alpha and beta diversity are correlated with age in the "Duroc × Landrace" piglets (unrooted PD: $p=0.0059$; BWPD: $p=0.0226$; PC2: $p=0.0263$; PC5: $p=0.0063$) and to a lesser extent in the "Duroc × Large White" piglets (unrooted PD: $p=0.0529$; PC5: $p=0.0310$) during the first week of the trial. In the "Duroc × Landrace" piglets, a correlation between age and beta diversity was detected in week two (PC2: $p=0.0347$) (**Figure 2**; **S14**; **Supplementary file 5**). Differences in beta diversity by date of birth, within the same cross-breed (Duroc × Large white), were found to be significant (TukeyHSD adjusted p value: 1 day difference: 0.000782; 2 days difference: 0.018603) (**Supplementary file 5**).

Maternal effect on phylogenetic diversity

As piglets were derived from 42 distinct sows (maternal sows), and nursed by either the same or a different sow (a nurse sow) (**Supplementary File 5**), a litter effect was expected and determined. Based on Hommel adjusted p values, a similarity of alpha and beta phylogenetic diversity can be seen among piglets from the same maternal sow (unrooted PD: $p=0.0096$; BWPD: $p=0.0467$) and in piglets from the same nurse sow (unrooted PD: $p=0.0320$; PC1: $p=0.0391$) (**Figure 2**; **S16-19**; **Supplementary file 5**). The litter effect

described was found in samples at the start of the trial. Significance of the correlations did not persist thereafter (**Figure 2**; **Supplementary file 5**).

A strong effect of aging on phylogenetic diversity

Beta diversity analysis revealed a distinct and consistent change of the microbial community over time in all piglets, regardless of the treatment. Samples clustered in PC2 (accounting for 21.68% of the variation), showing a higher representation of *Bacteroidetes*, *Gammaproteobacteria* and *Prevotellaceae* from day 0-20, and a higher representation of *Firmicutes*, *Mollicutes* and *Ruminococcaceae* from day 20-40 (**Figure 3**; **S20**). In PC1 (accounting for 46.99% of the variation) samples shifted towards a higher representation of *Lactobacilli* from day 0 to day 20, and towards a higher representation of *Actinobacteria*, *Clostridiales* and *Mollicutes* from day 20 to day 40 (**Figure 3**; **Supplementary file 5**). A temporary shift towards a higher representation of *Bifidobacteriaceae* (*Bifidobacterium* being a component of the probiotic D-Scour™) is seen in PC5 (3.04% of the variation explained) one and two weeks from the start of the trial (**S21**).

Beta diversity analysis was performed separately for samples within each time point in order to find taxa associated with variation within each time point. Extent of variation was derived from the product of branch width by the variation explained by the principal component (**S22**). The following taxa were responsible for variation only at the start of the trial: *Gammaproteobacteria* (t0=0.12), *Enterobacteriaceae* (t0=0.08) and *Archaea* (t0=0.03). The following taxa were responsible for variation throughout the trial: *Clostridiales* (min=0.11; max=0.25), *Bacteroidetes* (min=0.06; max=0.11), *Bacteroidetes chlorobi* group (min=0.05; max=0.10). *Lactobacillus* became variable after the first week of the trial and remained highly variable throughout the trial (min=0.13; max=0.22). Some taxa contributed less to the variability among the piglets' faecal population, but they nonetheless consistently contributed to variability: *Bacteroidales* (min=0.01; max=0.04); *Firmicutes* (min=0.05; max=0.08); *Bacilli* (min=0.06; max=0.09); *Lactobacillales* (min=0.04; max=0.08); *Actinobacteria* (min=0.02; max=0.09); *Proteobacteria* (min=0.02; max=0.07). The variability of *Prevotella* (min=0.01; max=0.04) increased from the second week of the trial. (**S22**)

Taxonomic representation in terms of abundance was derived from analysis of samples with guppy fat, where abundance was derived from the branch width (**Figure 4**) and

combined with PhyloSift's taxonomic annotation of the marker gene phylogeny. Overall, *Clostridiales* were the most abundant throughout the trial (t0=0.18; t2=0.20; t4=0.11; t6=0.09; t8=0.10; t10=0.08). *Lactobacillus acidophilus* increased at the start (t0=0.00; t2=0.05; t4=0.13) then decreased (t6=0.09; t8=0.05; t10=0.07). *Subdoligranulum variabile* decreased after the first week (t0=0.05; t2=0.02) then gradually increased (t4=0.04; t6=0.05; t8=0.06; t10=0.07). Among other prevalent taxa at the start of the trial up to the second week and dropping at later time points we found *Metahobrevibacter smithii* (t0=0.06; t2=0.03) and *Bacteroidales* (t0=0.02; t2=0.01). Following an opposite trend we found *Ruminococcus* sp. JC304 (t0=0.00; t2=0.00; t4=0.01; t6=0.02; t8=0.02; t10=0.03), *Solobacterium moorei* (t0=0.00; t2=0.01; t4=0.02; t6=0.02; t8=0.02; t10=0.02) and *Prevotella copri* (t0=0.00; t2=0.00; t4=0.04; t6=0.03; t8=0.05; t10=0.02). In modest and stable abundance across the trial were *Mogibacterium* sp. CM50 (t0=0.02; t2=0.03; t4=0.02; t6=0.03; t8=0.02; t10=0.04) and *Oscillibacter* (t0=0.06; t2=0.04; t4=0.03; t6=0.03; t8=0.03; t10=0.03). In transient abundance we found *Bifidobacterium thermophilum* RBL67 (t2=0.01; t4=0.02). Gradually increasing from the second week we found *Eubacterium biforme* DSM3989 (t2=0.01; t4=0.02; t6=0.02; t8=0.02; t10=0.02), *Eubacterium rectale* (t4=0.02; t6=0.02; t8=0.03; t10=0.01) and, after the third week, *Faecalibacterium prausnitsii* (t6=0.01; t8=0.02; t10=0.02).

The effect of time was also clear in alpha diversity, where all the piglet cohorts followed a similar trend over time, independent of the treatment (**S23**; **Supplementary file 5**). Upon arrival on the trial site, the piglets' unrooted PD was lower than the sows (sows: 125.39; piglets range: 104.6-112.7) and reached a similar unrooted PD to the sows' in the second week (range: 123.4-130.7) (**Supplementary file 5**).

In comparing four timepoints at one week intervals from the start of the trial (intervals labeled as A, B, C, D), changes in alpha diversity among all the piglets were tested for and significance was determined using the Bonferroni correction. Unrooted phylogenetic diversity increased after the start of the trial (A mean: 107.86; B mean: 127.21; $p<0.001$), decreased in the following week (C mean: 122.92; p=ns) and the fourth week (D mean: 118.74; p=ns) amongst the piglets cohorts. In contrast, BWPD decreased after the start of the trial (A mean: 2.17; B mean: 2.05; $p<0.0001$), to increase in the following week (C mean: 2.14; $p<0.0001$) and decrease in the fourth week (D mean: 2.07; $p<0.01$). (**S23**; **Supplementary file 5**). The increase in unrooted PD in the first week was significant for all

treatment cohorts (*p* range: 0.0015-0.03) except ColiGuard® and neomycin (*p*=ns) cohorts. The decrease in BWPD in the first week was only significant for the control (*p*=0.04), ColiGuard® (*p*=0.0015) and neomycin+ColiGuard® (*p*=0.036) cohorts. In week two only the increase of BWPD of the control cohort reached statistical significance (*p*=0.0121). (**S24**; **Supplementary file 5**)

Effect of antibiotic and probiotic treatment on alpha diversity

We hypothesized that the probiotic treatments, whether alone (D-Scour™ and ColiGuard®) or administered after neomycin (neomycin+D-Scour™ and neomycin+ColiGuard®) would cause a change in the microbial community composition that would be measurable via phylogenetic diversity. We tested whether the treatments correlated with a change in phylogenetic diversity independently of the changes occurring with time. Given the differences in alpha and beta diversity detected among the subjects at the start of the trial, we analyzed the deltas of phylogenetic diversity instead of relying on the absolute means, similar to the procedure applied by Kembel *et al* (2012) [114]. Time-point measurements of alpha diversity were taken and deltas were computed for each piglet. Delta means were compared between cohorts, where the control cohort would serve as a control group for neomycin, D-Scour™ and ColiGuard® cohorts, whereas the neomycin cohort would serve as a control group for the neomycin+D-Scour™ and neomycin+ColiGuard® cohorts.

After the first week of the trial, the majority of the piglets displayed an increase in unrooted PD (85%) and a decrease of BWPD (75%). The following week the trend was opposite: an increase of BWPD (78%) and a decrease of unrooted PD (60%) (**Supplementary file 5**). However, the neomycin cohort displayed the smallest BWPD drop in the week following the start of the trial and the overall trend of neomycin in unrooted PD was the most different from the other cohorts (**S25**). Due to the delayed rise in unrooted PD of neomycin, significance was found in unrooted PD between neomycin and neomycin+D-Scour™ during the first week of D-Scour™ treatment (neomycin mean: 1.59; neomycin+D-Scour™ mean: -11.79; *p*= 0.012188) (**S25 B-C**) and in unrooted PD between neomycin and neomycin+ColiGuard® (neomycin mean: -5.53; neomycin+ColiGuard®: -6.70; *p*=0.043138) (**S25 C-D**). The change was opposite in BWPD (*p*=0.049263) (**S26 B-C**) and persisted during the following week, up to 3 days post- D-Scour™ treatment (neomycin mean: -6.34; neomycin+D-Scour™ mean: 4.52; BH adjusted *p*=0.024092) (**S26 B-D**).

The only significant difference between the neomycin and the control cohort was detected in BWPD in the second week of the trial (control mean: 7.10; neomycin mean: 0.28) p=0.033751) (**S26 B-C**).

No significant differences were detected between the control and the D-Scour™ cohort (p<0.05) (**Supplementary file 5**). Instead, a significant decrease in BWPD was detected in the ColiGuard® cohort between the start of the trial and the end of the ColiGuard® treatment, where nearly all piglets (88%) in the ColiGuard® cohort displayed a decrease in BWPD, whereas the control cohort was split between piglets increasing (53%) and decreasing (47%) in BWPD (control mean: 0.85; ColiGuard® mean: -5.49; p=0.040041) (**S26 A-C**).

Effects of antibiotic and probiotic treatment on beta diversity

To investigate the treatment effect on beta diversity, principal component analysis (PCA) of the Kantorovich-Rubenstein distances (beta diversity analysis) was performed on all samples and, additionally, on samples within individual time points. This analysis is conceptually similar to the weighted Unifrac approach for beta diversity analysis, but is designed to work with phylogenetic placement data [92]. When examining all samples together, there was no clear separation of cohorts on any of the five principal component axes. When individual time points were analysed, some clustering by cohort was observed (**S27**). D-Scour™ separated from the control cohort in PC2 (17.95%) and in PC3 (8.06%) one week and two weeks after probiotic treatment, respectively (**S27**). ColiGuard® separated from control two weeks post probiotic treatment in PC3 (7.88%). Two weeks after the end of neomycin treatment, neomycin separated from control in PC4 (5.32%), with a smaller clustering and a higher representation of *Mollicutes*. Two and four weeks after probiotic treatment, neomycin+D-Scour™ separated from neomycin in PC2 (17.74%) and PC5 (3.81%), respectively. In both timepoints and principal components, neomycin+D-Scour™ showed a higher representation of *Lactobacillales*. (**S27**)

Association between weight and community composition

Weight correlated with the abundance of certain species (n=83) at each time point as it resulted from principal component analysis of 16S rRNA reads. Positively correlating with weight we found among others: *Blautia* (t0), *Cetobacterium* (t0), *Ruminibacter* (t0), *Rickettsia* (t2), *Lactobacillus* (t6), *Mycoplasma* (t6), *Anaerostipes* (t8), *Ruminococcus* (t8-

t10), *Cerasibacillus* (t10). Negatively correlating with weight we found among others: *Pyramidobacter* (t0), *Odoribacter* (t2), *Schwartzia* (t6), *Streptococcus* (t6), *Dokdonella* (t8). (**S28-S33**) Significance values and correlation estimates are given in **Supplementary file 5**.

Effect of treatments on weight gain

Overall weight gain from initial to final weight (**S34 A-E**; **Supplementary file 5**) was not significantly affected by any treatment. However, the probiotic ColiGuard® was found to have a partial effect on piglet weight gain (**S34**; **Supplementary file 5**). Weight was measured weekly for a total of six measurements. Based on Tukey adjusted *p* values, a lower weight gain was detected in the ColiGuard® cohort in week 3 compared to the control cohort (p=0.0008) (**S34 C-D**; **Supplementary file 5**). Similarly, a lower weight gain was detected in the neomycin+ColiGuard® cohort in C-E compared to the neomycin cohort (p=0.0393) (**S34 C-E**; **Supplementary file 5**). Breed and age differences among piglets were not associated with weight gain (**Supplementary file 5**).

DISCUSSION

The consistent trend in community composition over time, across all the cohorts, indicates that an age-related process of ecological succession is the largest factor shaping the microbial community of post-weaning piglets, as found in this study where animals aged 20-63 days and were fed the same diet. A peak in unrooted phylogenetic diversity and drop in balance weighted phylogenetic diversity (BWPD) reflects the acquisition of new species with the loss of dominating species. This change, detected in the week following the piglets' arrival at the trial site irrespective of the cohort, could be linked to the piglets being subjected to microbial interchange (e.g.: new pen mates [115,116]) and/or to diet transition (peri-weaning transition to solid food [115,116]) leading to the reshaping of the gut microbial community. The week following the drop of BWPD, a significant increase of BWPD was recorded, reflecting the acquisition of a larger proportion of the community by the newly introduced species. The strong changes in phylogenetic diversity detected in the first and the second week could as well be attributable to other post-weaning related physiological changes, as previous studies report [44,115–117].

The highest inter-individual differences among piglets are seen in the first week of life, irrespective of maternal or environmental effects. The microbiota of 3 week old piglets is

still very dynamic, but environmental factors become evident [116]. At 6 weeks of age, CD8+ T cells infiltrate the intestinal tissue and the mucosa and intestinal lining resemble that of an adult pig [115] . In this study, piglets reached a comparable alpha diversity to the sows after the first week of the trial, at which time the piglets were aged between 3.8 and 4.6 weeks. Unrooted PD did not reach higher levels at later sampling time points. The highest BWPD accompanied by a high unrooted PD was reached after the second week of the trial when piglets were aged between 4.9 and 5.6 weeks. Age-dependent physiological changes could explain i) the major shifts we detected in alpha diversity during the first two weeks of the trial and, ii) the distinct differences in community composition with age, even with a narrow age difference between piglets (1-6 days). We were able to appreciate a significant trend of increasing unrooted PD and decreasing BWPD with age in piglets that separated a 6 days maximum by day of birth from each other. Since age groups were confounded with breeds in our study, we attempted to determine the correlation within single breeds. Unfortunately, although the correlations with age could still be detected, we could not determine the association at later time points due to the introduction of treatment effects.

Animal trials are often conducted in controlled environments so as to minimize environmental effects. However, individual variations such as breed and age are often unavoidable in large animal trials. Previously reported confounding factors include: individual variation [44], cohabitation [115,116], age [44,115,116], maternal effects [115,116,118], hormones [44], behavioural differences between breeds (e.g. coprophagy, mouth to mouth contact) and extent of long-term behavioural adaptation, which can differ between breeds for reasons not attributable to genetics [44,115,116]. A litter effect was found in piglets at the start of the trial and was lost at later time points during the trial. This could be due to either of the aforementioned factors. Co-housing, aging and the splitting of the piglets in separate rooms to receive a different treatment, are possible causes for loss of the litter effect. In this study we confirm the importance of these factors in the contribution to inter-individual variability of gut microbial composition. Motta *et al* (2019) report a correlation of beta diversity with age and no correlation of genotype and litter effect with either alpha or beta diversity [9] . On the contrary, we found the piglet samples to significantly cluster by litter, breed and by age up to the second and the fourth week post weaning, in alpha diversity and beta diversity, respectively. We conclude that even small age differences among post- weaning piglets, down to the day, must be accounted for in an experimental set up.

Three groups of piglets (cohorts neomycin, neomycin+D-Scour™ and neomycin+ColiGuard®) underwent 5 days of treatment with the broad-spectrum antibiotic neomycin, via intramuscular administration. Intramuscular neomycin poorly diffuses (<10%) into a healthy gastrointestinal tract [119] , therefore a direct effect of neomycin on the gut microbiome may not be expected. However, neomycin showed a different trend in unrooted PD between the second and the third week of the trial, corresponding to the week following the neomycin treatment period for the neomycin cohort. Taking this time frame into consideration, the neomycin cohort did not increase in BWPD to the extent of the Control cohort. Although statistically significant differences between neomycin and Control in alpha diversity were not reached, the BWPD of the neomycin cohort appears to follow a different trend to the Control from the first week (during neomycin treatment) where neomycin treated piglets show the lowest decrease of BWPD compared to the control cohort and all other cohorts. While all cohorts show an increase in absolute phylogenetic diversity accompanied by a decrease of diversity evenness during this time frame, the neomycin cohort piglets show a lower drop in BWPD, suggesting an increase of species richness, without a corresponding loss of species evenness. Furthermore the neomycin cohort significantly separated from the control cohort in beta diversity in the third week of the trial, showing a higher representation of *Mollicutes*. Numerous studies report the link of oral antibiotic use with dysbiosis [22,35–38,44,45], as well as with host physiology changes [37]. On the contrary, the effect of intramuscular antibiotic administration on the microbiome is less well investigated. Correlation between intramuscular antibiotic use and dysbiosis has been reported in fish [46], gorillas [47], humans [120], and pigs [48,121]. In 1 day old piglets, a single IM injection of amoxicillin (penicillin class) is reported to have an effect on the intestinal microbiota, detectable 40 days post treatment [48]. Zeineldin *et al* (2018) tested the effects of IM administration of several antibiotics of various classes (penicillin, macrolide, cephalosporin and tetracycline), in 8-week old piglets, reporting shifts of the *Firmicutes*/*Bacteroidetes* ratio following treatment (length of the treatment not reported)[121]. The effects of intramuscular administration of neomycin (aminoglycoside class) on the gut microbiota have to our knowledge not been investigated. Based on our results we conclude that a mild effect on phylogenetic diversity is appreciable post IM neomycin treatment, up to two weeks after termination of the treatment. Additional compositional and functional analysis is necessary to determine the source of this mild variation. Differences were not detected at later time points, based on our phylogenetic

diversity analyses, suggesting a full recovery of the microbial communities after two week from the end of the treatment.

It is possible that the large shifts in phylogenetic diversity taking place in the first two weeks irrespective of the treatment (an increase, then decrease of unrooted PD, and an opposite trend of BWPD) have masked the milder effects of the treatment, despite our efforts to control for the effects of aging. This could be the reason why a significantly distinct alpha diversity trend was found in the neomycin+D-Scour™ cohort compared to the neomycin cohort, but not in the D-Scour™ cohort compared to the Control cohort. The neomycin+D-Scour™ cohort underwent 5 days of neomycin treatment followed by 2 weeks of D-Scour™ treatment. A significant increase of BWPD was detected in the two-week period of D-Scour™ treatment, indicating a possible enhancement of microbiome evenness following neomycin treatment. To our knowledge there are no studies reporting an increased evenness in piglet gut community composition following a specific probiotic treatment. There are instead multiple studies reporting beneficial effects of probiotic treatment in sucker and weaner piglets in terms of improved gut mucosal integrity [66,80], growth rate [80–83], digestibility of proteins and water absorption [80,83], reduction of pathogen invasion efficiency [76,79,80], and decreased mortality [80,82]. Although the assessment of physiologic changes from probiotic treatments was outside the scope of this study, we found significant separation of neomycin+D-Scour™ cohort samples to neomycin cohort samples in beta diversity 3 and 10 days after D-Scour™ treatment, where neomycin+D-Scour™ samples showed a higher representation of *Lactobacillales* compared to neomycin samples, suggesting a transient establishment of the probiotic strains in the piglet guts.

The second probiotic in this study, ColiGuard®, did not have an effect on alpha diversity, but clustering was detected in beta diversity, where ColiGuard® samples separated from Control cohort samples in the third principal component (explaining 7.88% of the variation) two weeks post probiotic treatment. Additionally, the ColiGuard® treatment correlated with a lower weight gain, whether or not it was preceded by the antibiotic treatment. However, when comparing the overall weight gain (from the start to the end of the trial) the weight gain in the cohorts receiving ColiGuard® did not differ from the other cohorts.

We extracted the 16S rRNA gene hypervariable regions from our dataset, obtained the counts, and ran a correlation analysis to discover taxa that correlated with the weight of the

piglets. As a consequence of the library size normalization step, the use of correlation with compositional data can inflate the false discovery rate [122,123]. For this reason it can be expected that some of the taxa we found to correlate with the weight of the piglets (eighty-three distinct species) could be spurious while other correlations may have been missed.

Technical controls in metagenomic studies and methodological limitations

Taxonomic assignment of the raw reads from the positive controls was performed with MetaPhlAn2[112] which relies on a ca. 1M unique clade-specific markers derived from 17,000 reference genomes. Such a database to map against the positive controls suffices as these organisms are cultivable, and for this reason they are widely studied hence the sequences are known. This is not the case for real-world samples where mapping against a database (which completeness relies on studied and often cultivable organisms) would narrow the view on the true diversity within the sample.

Positive controls with well-studied members and known ratios within the samples, has proven to be a valuable tool to assess consistency among technical replicates across batches and to detect possible biases derived from the DNA extraction method.

Systematic taxonomic bias in microbiome studies, resulting from differences in cell wall structures between Gram positive and Gram negative bacteria, have previously been reported; sample treatment with enzymatic cocktails can modestly reduce this bias [124–126]. Although we implemented this step in our workflow, it seems that, from the read abundance of our mock community, which contained three Gram negative and four Gram positive strains, a bias towards Gram negative taxa may still be present.

In terms of contamination we concluded that: a) contamination in our study was not batch specific; b) a problem of sample cross-contamination existed at the DNA extraction step between neighbouring wells. During the bead beating step of DNA extraction, the deep-well plate is sealed with a sealing mat, rotated and placed in a plate shaker for the bead beating to take place. We consider that sample cross-contamination is most likely to occur during this step.

Conclusion

Our study provides a publicly-available databank of the pig gut metagenome. Our findings further stress the importance of confounding factors such as breed, age and maternal effects when assessing the effect of treatment on the gut microbiome. We found that age, even within a narrow age span (1-6 days) can have an impact on microbial shifts and should be accounted for in microbiome studies. Intramuscular neomycin treatment correlated with a clustering in alpha diversity and a higher representation of *Mollicutes* compared to control. D-Scour™ treated piglets displayed a mild shift in alpha diversity compared to control, and a transient establishment of *Lactobacillales*. ColiGuard® treated piglets displayed a clustering in beta diversity and a transient lower weight gain compared to control. Weight correlated with the abundance of a number of taxa. Age was the strongest factor shaping phylogenetic diversity of the piglets.

As previously mentioned, phylogenetic diversity is based on distinct taxa (richness) and their collective structure (proportions reflected by BWPD) and not on a direct assessment of composition and function. These types of analyses will be necessary to further describe the effects of the treatments.

Declarations

Competing Interests

D-Scour™ was sourced from International Animal Health Products (IAHP). ColiGuard® was developed in a research project with NSW DPI, IAHP and AusIndustry Commonwealth government funding.

Authors Contributions

Pig Trial: TC, LF, DG, TZ, GJE, AED, SPD

DNA extraction: DG, ML

Library prep, robot pooling: DG, ML, KA, AED

Sequencing data processing: MZD, DG, AED

Data analysis: DG, AED

Manuscript: DG

Manuscript editing: DG, AED, GJE, John Webster

Funding

This work was supported by the Australian Research Council, linkage grant LP150100912. This project was funded by the Australian Centre for Genomic Epidemiological Microbiology (Ausgem), a collaborative partnership between the NSW Department of Primary Industries and the University of Technology Sydney. TZ and DG are recipients of UTS International Research and UTS President's Scholarships. NSW DPI approved the paper before submission for publication.

Acknowledgements

We would like to thank the on site team of the pig trial: Graeme Eamens, Linda Falconer, Shayne Fell, Tiziana Zingali, Toni Chapman. Thank you Amy Bottomley, Giulia Ballerin, Rosy Cavaliere, for providing the mock community strains. Thank you Akane Tanaka, Leigh Monahan and Joyce To for the technical support. Thank you John Webster for providing DPI internal review of this manuscript and for the helpful suggestion about its content. International Animal Health Products for providing access to the probiotic supplements and the Australian Centre for Genomic Epidemiological Microbiology (Ausgem) for financially supporting this study.

Supplementary Files

Supplementary File 1. (animal details)

Supplementary File 2. (metadata)

Supplementary File 3. (barcodes)

Supplementary File 4. (multiqc report)

Supplementary file 5. (stats output)

REFERENCES

1. Jensen, P. & Recén, B. When to wean — Observations from free-ranging domestic pigs. *Appl. Anim. Behav. Sci.* **23**, 49–60 (1989).
2. Van Heugten, E., Coffey, M. T. & Spears, J. W. Effects of immune challenge, dietary energy density, and source of energy on performance and immunity in weanling pigs. *J. Anim. Sci.* **74**, 2431–2440 (1996).
3. Blecha, F. Immune system response to stress. *Biol. Anim. Stress Basic Princ. Implic. Anim. Welf. Moberg GP Mench JA Eds* 111–123 (2000).
4. Fairbrother, J. M., Nadeau, É. & Gyles, C. L. Escherichia coli in postweaning diarrhea in pigs: an update on bacterial types, pathogenesis, and prevention strategies. *Anim. Health Res. Rev.* **6**, 17–39 (2005).
5. Faubert, C. & Drolet, R. Hemorrhagic gastroenteritis caused by Escherichia coli in piglets: clinical, pathological and microbiological findings. *Can. Vet. J.* **33**, 251 (1992).
6. Nabuurs, M. J. A. Weaning piglets as a model for studying pathophysiology of diarrhea. *Vet. Q.* **20**, 42–45 (1998).
7. Lallès, J.-P., Bosi, P., Smidt, H. & Stokes, C. R. Weaning — A challenge to gut physiologists. *Livest. Sci.* **108**, 82–93 (2007).
8. Heo, J. M. *et al*. Gastrointestinal health and function in weaned pigs: a review of feeding strategies to control post-weaning diarrhoea without using in-feed antimicrobial compounds. *J. Anim. Physiol. Anim. Nutr.* **97**, 207–237 (2013).
9. Motta, V., Luise, D., Bosi, P. & Trevisi, P. Faecal microbiota shift during weaning transition in piglets and evaluation of AO blood types as shaping factor for the bacterial community profile. *PloS One* **14**, e0217001 (2019).

10. Alain B. Pajarillo, E., Chae, J.-P., P. Balolong, M., Bum Kim, H. & Kang, D.-K. Assessment of fecal bacterial diversity among healthy piglets during the weaning transition. *J. Gen. Appl. Microbiol.* **60**, 140–146 (2014).
11. Kaper, J. B., Nataro, J. P. & Mobley, H. L. T. Pathogenic Escherichia coli. *Nat. Rev. Microbiol.* **2**, 123–140 (2004).
12. Jordan, D. *et al.* Antimicrobial use in the Australian pig industry: results of a national survey. *Aust. Vet. J.* **87**, 222–229 (2009).
13. Zhu, Y.-G. *et al.* Diverse and abundant antibiotic resistance genes in Chinese swine farms. *Proc. Natl. Acad. Sci.* **110**, 3435–3440 (2013).
14. Erik, K. & Knudsen, B. Development of antibiotic resistance and options to replace antimicrobials in animal diets. *Proc. Nutr. Soc.* **60**, 291–299 (2001).
15. Levy, S. B. & Marshall, B. Antibacterial resistance worldwide: causes, challenges and responses. *Nat. Med.* **10**, S122 (2004).
16. White, D. G. Antimicrobial resistance in pathogenic Escherichia coli from animals. in *Antimicrobial resistance in bacteria of animal origin* 145–166 (American Society of Microbiology, 2006).
17. Broens, E. M., Graat, E. A. M., Van der Wolf, P. J., Van de Giessen, A. W. & De Jong, M. C. M. Prevalence and risk factor analysis of livestock associated MRSA-positive pig herds in The Netherlands. *Prev. Vet. Med.* **102**, 41–49 (2011).
18. Modi, S. R., Lee, H. H., Spina, C. S. & Collins, J. J. Antibiotic treatment expands the resistance reservoir and ecological network of the phage metagenome. *Nature* **499**, 219–222 (2013).
19. Prescott, J. F. History of antimicrobial usage in agriculture: an overview. in *Antimicrobial resistance in bacteria of animal origin* 19–28 (American Society of Microbiology, 2006).

20. Marshall, B. M. & Levy, S. B. Food animals and antimicrobials: impacts on human health. *Clin. Microbiol. Rev.* **24**, 718–733 (2011).

21. Callens, B. *et al.* Presence of antimicrobial resistance and antimicrobial use in sows are risk factors for antimicrobial resistance in their offspring. *Microb. Drug Resist.* **21**, 50–58 (2015).

22. Looft, T. P. *The swine intestinal microbiota: localized adaptations and responses to in-feed antibiotics.* (Iowa State University, 2012).

23. Salyers, A. A. & Shoemaker, N. B. Resistance gene transfer in anaerobes: new insights, new problems. *Clin. Infect. Dis.* **23**, S36–S43 (1996).

24. Salyers, A. A. & Amábile-Cuevas, C. F. Why are antibiotic resistance genes so resistant to elimination? *Antimicrob. Agents Chemother.* **41**, 2321–2325 (1997).

25. Dorado-García, A. *et al.* Dose-Response Relationship between Antimicrobial Drugs and Livestock-Associated MRSA in Pig Farming1. *Emerg. Infect. Dis.* **21**, 950–959 (2015).

26. Didelot, X. & Maiden, M. C. J. Impact of recombination on bacterial evolution. *Trends Microbiol.* **18**, 315–322 (2010).

27. Canchaya, C., Fournous, G., Chibani-Chennoufi, S., Dillmann, M.-L. & Brüssow, H. Phage as agents of lateral gene transfer. *Curr. Opin. Microbiol.* **6**, 417–424 (2003).

28. Claverys, J.-P., Martin, B. & Polard, P. The genetic transformation machinery: composition, localization, and mechanism. *FEMS Microbiol. Rev.* **33**, 643–656 (2009).

29. Ravi, A., Avershina, E., Ludvigsen, J., L'Abée-Lund, T. & Rudi, K. Integrons in the Intestinal Microbiota as Reservoirs for Transmission of Antibiotic Resistance Genes. *Pathogens* **3**, 238–248 (2014).

30. Porse, A., Schønning, K., Munck, C. & Sommer, M. O. A. Survival and Evolution of a Large Multidrug Resistance Plasmid in New Clinical Bacterial Hosts. *Mol. Biol. Evol.* **33**, 2860–2873 (2016).

31. Bednorz, C. *et al.* The broader context of antibiotic resistance: Zinc feed supplementation of piglets increases the proportion of multi-resistant Escherichia coli in vivo. *Int. J. Med. Microbiol.* **303**, 396–403 (2013).

32. Tadesse, D. A. *et al.* Antimicrobial drug resistance in Escherichia coli from humans and food animals, United States, 1950–2002. *Emerg. Infect. Dis.* **18**, 741 (2012).

33. U. S. Food & Drug, A. FDA Task Force on Antimicrobial Resistance: Key Recommendations and Report. (2000).

34. Centers for Disease Control & Prevention. Get smart: know when antibiotics work. Centers for Disease Control and Prevention, Atlanta, GA. (2010).

35. Dethlefsen, L. & Relman, D. A. Incomplete recovery and individualized responses of the human distal gut microbiota to repeated antibiotic perturbation. *Proc. Natl. Acad. Sci.* **108**, 4554–4561 (2011).

36. Cho, I. *et al.* Antibiotics in early life alter the murine colonic microbiome and adiposity. *Nature* **488**, 621–626 (2012).

37. Antunes, L. C. M. *et al.* Effect of Antibiotic Treatment on the Intestinal Metabolome. *Antimicrob. Agents Chemother.* **55**, 1494–1503 (2011).

38. Gonzalez-Perez, G. *et al.* Maternal Antibiotic Treatment Impacts Development of the Neonatal Intestinal Microbiome and Antiviral Immunity. *J. Immunol.* **196**, 3768–3779 (2016).

39. Sachs, J. L., Mueller, U. G., Wilcox, T. P. & Bull, J. J. The evolution of cooperation. *Q. Rev. Biol.* **79**, 135–160 (2004).

40. Foster, K. R. & Wenseleers, T. A general model for the evolution of mutualisms. *J. Evol. Biol.* **19**, 1283–1293 (2006).

41. Birck, M. M. *et al.* Enteral but not parenteral antibiotics enhance gut function and prevent necrotizing enterocolitis in formula-fed newborn preterm pigs. *Am. J. Physiol.-Gastrointest. Liver Physiol.* **310**, G323–G333 (2016).

42. Wiuff, C., Lykkesfeldt, J., Svendsen, O. & Aarestrup, F. M. The effects of oral and intramuscular administration and dose escalation of enrofloxacin on the selection of quinolone resistance among Salmonella and coliforms in pigs. *Res. Vet. Sci.* **75**, 185–193 (2003).

43. Chantziaras, I., Smet, A., Haesebrouck, F., Boyen, F. & Dewulf, J. Studying the effect of administration route and treatment dose on the selection of enrofloxacin resistance in commensal Escherichia coli in broilers. *J. Antimicrob. Chemother.* **72**, 1991–2001 (2017).

44. Xiao, L. *et al.* A reference gene catalogue of the pig gut microbiome. *Nat. Microbiol.* **1**, 16161 (2016).

45. Deshmukh, H. S. *et al.* The microbiota regulates neutrophil homeostasis and host resistance to Escherichia coli K1 sepsis in neonatal mice. *Nat. Med.* **20**, 524–530 (2014).

46. Kim, A. *et al.* Administration of antibiotics can cause dysbiosis in fish gut. *Aquaculture* **512**, 734330 (2019).

47. Vlčková, K. *et al.* Effect of Antibiotic Treatment on the Gastrointestinal Microbiome of Free-Ranging Western Lowland Gorillas (Gorilla g. gorilla). *Microb. Ecol.* **72**, 943–954 (2016).

48. Janczyk, P. *et al*. Parenteral long-acting amoxicillin reduces intestinal bacterial community diversity in piglets even 5 weeks after the administration. *ISME J.* **1**, 180–183 (2007).

49. Allen, H. K., Levine, U. Y., Looft, T., Bandrick, M. & Casey, T. A. Treatment, promotion, commotion: antibiotic alternatives in food-producing animals. *Trends Microbiol.* **21**, 114–119 (2013).

50. Rangan, K. J. *et al*. A secreted bacterial peptidoglycan hydrolase enhances tolerance to enteric pathogens. *science* **353**, 1434–1437 (2016).

51. Tap, J. *et al*. Gut microbiota richness promotes its stability upon increased dietary fibre intake in healthy adults. *Environ. Microbiol.* **17**, 4954–4964 (2015).

52. Kaleko, M. *et al*. Development of SYN-004, an oral beta-lactamase treatment to protect the gut microbiome from antibiotic-mediated damage and prevent Clostridium difficile infection. *Anaerobe* (2016) doi:10.1016/j.anaerobe.2016.05.015.

53. Song, J., Li, Y. li & Hu, C. hong. Effects of copper-exchanged montmorillonite, as alternative to antibiotic, on diarrhea, intestinal permeability and proinflammatory cytokine of weanling pigs. *Appl. Clay Sci.* **77–78**, 52–55 (2013).

54. Holler, E. *et al*. Metagenomic Analysis of the Stool Microbiome in Patients Receiving Allogeneic Stem Cell Transplantation: Loss of Diversity Is Associated with Use of Systemic Antibiotics and More Pronounced in Gastrointestinal Graft-versus-Host Disease. *Biol. Blood Marrow Transplant.* **20**, 640–645 (2014).

55. Nogueira, M., Collins, A., Dunlop, R. & Emery, D. Effect of the route of administration on the mucosal and systemic immune responses to *Lawsonia intracellularis* vaccine in pigs. *Aust. Vet. J.* **93**, 124–126 (2015).

56. Bak, H. & Rathkjen, P. H. Reduced use of antimicrobials after vaccination of pigs against porcine proliferative enteropathy in a Danish SPF herd. *Acta Vet. Scand.* **51**, 1 (2009).

57. Francis, D. H. & Willgohs, J. A. Evaluation of a live avirulent Escherichia coli vaccine for K88+, LT+ enterotoxigenic colibacillosis in weaned pigs. *Am. J. Vet. Res.* **52**, 1051–1055 (1991).

58. Morgan, R. L., Isaacson, R. E., Moon, H. W., Brinton, C. C. & To, C. C. Immunization of suckling pigs against enterotoxigenic Escherichia coli-induced diarrheal disease by vaccinating dams with purified 987 or K99 pili: protection correlates with pilus homology of vaccine and challenge. *Infect. Immun.* **22**, 771–777 (1978).

59. Callaway, T. R. *et al.* Evaluation of Phage Treatment as a Strategy to Reduce *Salmonella* Populations in Growing Swine. *Foodborne Pathog. Dis.* **8**, 261–266 (2011).

60. Willamil, J., Creus, E., Francisco Pérez, J., Mateu, E. & Martín-Orúe, S. M. Effect of a microencapsulated feed additive of lactic and formic acid on the prevalence of Salmonella in pigs arriving at the abattoir. *Arch. Anim. Nutr.* **65**, 431–444 (2011).

61. Brouns, F., Kettlitz, B. & Arrigoni, E. Resistant starch and "the butyrate revolution". *Trends Food Sci. Technol.* **13**, 251–261 (2002).

62. Missotten, J. A. M., Michiels, J., Ovyn, A., De Smet, S. & Dierick, N. A. Fermented liquid feed for pigs. *Arch. Anim. Nutr.* **64**, 437–466 (2010).

63. Canibe, N. & Jensen, B. B. Fermented and nonfermented liquid feed to growing pigs: effect on aspects of gastrointestinal ecology and growth performance. *J. Anim. Sci.* **81**, 2019–2031 (2003).

64. Moran, C. A. Development and benefits of liquid diets for newly weaned pigs. *PhD Univ. Plymouth* (2001).

65. Lessard, M. *et al*. Administration of Pediococcus acidilactici or Saccharomyces cerevisiae boulardii modulates development of porcine mucosal immunity and reduces intestinal bacterial translocation after Escherichia coli challenge1, 2. *J. Anim. Sci.* **87**, 922 (2009).
66. Deng, J., Li, Y., Zhang, J. & Yang, Q. Co-administration of Bacillus subtilis RJGP16 and Lactobacillus salivarius B1 strongly enhances the intestinal mucosal immunity of piglets. *Res. Vet. Sci.* **94**, 62–68 (2013).
67. Garrido-Mesa, N. *et al*. The association of minocycline and the probiotic Escherichia coli Nissle 1917 results in an additive beneficial effect in a DSS model of reactivated colitis in mice. *Biochem. Pharmacol.* **82**, 1891–1900 (2011).
68. Ukena, S. N. *et al*. Probiotic Escherichia coli Nissle 1917 Inhibits Leaky Gut by Enhancing Mucosal Integrity. *PloS One* **2**, e1308 (2007).
69. Deriu, E. *et al*. Probiotic bacteria reduce salmonella typhimurium intestinal colonization by competing for iron. *Cell Host Microbe* **14**, 26–37 (2013).
70. Hancock, V., Dahl, M. & Klemm, P. Probiotic Escherichia coli strain Nissle 1917 outcompetes intestinal pathogens during biofilm formation. *J. Med. Microbiol.* **59**, 392–399 (2010).
71. Heyman, M. Effect of lactic acid bacteria on diarrheal diseases. *J. Am. Coll. Nutr.* **19**, 137S-146S (2000).
72. Jin, L.-Z., Marquardt, R. R. & Baidoo, S. K. Inhibition of enterotoxigenic Escherichia coli K88, K99 and 987P by the Lactobacillus isolates from porcine intestine. *J Sci Food Agric* 6 (2000).
73. Maltby, R., Leatham-Jensen, M. P., Gibson, T., Cohen, P. S. & Conway, T. Nutritional basis for colonization resistance by human commensal Escherichia coli strains HS and

Nissle 1917 against E. coli O157: H7 in the mouse intestine. *PloS One* **8**, e53957 (2013).

74. Suda, Y. *et al*. Immunobiotic Lactobacillus jensenii as immune-health promoting factor to improve growth performance and productivity in post-weaning pigs. *BMC Immunol.* **15**, 24 (2014).

75. Shimazu, T. *et al*. Immunobiotic Lactobacillus jensenii Elicits Anti-Inflammatory Activity in Porcine Intestinal Epithelial Cells by Modulating Negative Regulators of the Toll-Like Receptor Signaling Pathway. *Infect. Immun.* **80**, 276–288 (2012).

76. Splichalova, A. *et al*. Interference of Bifidobacterium choerinum or Escherichia coli Nissle 1917 with Salmonella Typhimurium in gnotobiotic piglets correlates with cytokine patterns in blood and intestine. *Clin. Exp. Immunol.* **163**, 242–249 (2011).

77. Roselli, M. *et al*. The novel porcine Lactobacillus sobrius strain protects intestinal cells from enterotoxigenic Escherichia coli K88 infection and prevents membrane barrier damage. *J. Nutr.* **137**, 2709–2716 (2007).

78. Altenhoefer, A. *et al*. The probiotic Escherichia coli strain Nissle 1917 interferes with invasion of human intestinal epithelial cells by different enteroinvasive bacterial pathogens. *FEMS Immunol. Med. Microbiol.* **40**, 223–229 (2004).

79. Šmajs, D. *et al*. Experimental Administration of the Probiotic Escherichia coli Strain Nissle 1917 Results in Decreased Diversity of E. coli Strains in Pigs. *Curr. Microbiol.* **64**, 205–210 (2012).

80. Valeriano, V. D. V., Balolong, M. P. & Kang, D. K. Probiotic roles of Lactobacillus sp. in swine: insights from gut microbiota. *J. Appl. Microbiol.* **122**, 554–567 (2017).

81. Abe, F., Ishibashi, N. & Shimamura, S. Effect of administration of bifidobacteria and lactic acid bacteria to newborn calves and piglets. *J. Dairy Sci.* **78**, 2838–2846 (1995).

82. Davis, M. E. *et al.* Effect of a Bacillus-based direct-fed microbial feed supplement on growth performance and pen cleaning characteristics of growing-finishing pigs. *J. Anim. Sci.* **86**, 1459–1467 (2008).
83. Chu, G. M., Lee, S. J., Jeong, H. S. & Lee, S. S. Efficacy of probiotics from anaerobic microflora with prebiotics on growth performance and noxious gas emission in growing pigs: SYNBIOTICS ON NOXIOUS GAS EMISSION IN PIGS. *Anim. Sci. J.* **82**, 282–290 (2011).
84. Kim, P. I. *et al.* Probiotic properties of Lactobacillus and Bifidobacterium strains isolated from porcine gastrointestinal tract. *Appl. Microbiol. Biotechnol.* **74**, 1103–1111 (2007).
85. Takahashi, S., Egawa, Y., Simojo, N., Tsukahara, T. & Ushida, K. Oral administration of Lactobacillus plantarum strain Lq80 to weaning piglets stimulates the growth of indigenous lactobacilli to modify the lactobacillal population. *J. Gen. Appl. Microbiol.* **53**, 325–332 (2007).
86. Gaio, D. *et al.* Hackflex: low cost Illumina sequencing library construction for high sample counts. *bioRxiv* 779215 (2019).
87. Ewels, P., Magnusson, M., Lundin, S. & Käller, M. MultiQC: summarize analysis results for multiple tools and samples in a single report. *Bioinformatics* **32**, 3047–3048 (2016).
88. Ewels, P. A. *et al.* nf-core *: Community curated bioinformatics pipelines*. (2019).
89. Darling, A. E. *et al.* PhyloSift: phylogenetic analysis of genomes and metagenomes. *PeerJ* **2**, e243 (2014).
90. Faith, D. P. Conservation evaluation and phylogenetic diversity. *Biol. Conserv.* **61**, 1–10 (1992).

91. McCoy, C. O. & Matsen, F. A. Abundance-weighted phylogenetic diversity measures distinguish microbial community states and are robust to sampling depth. *PeerJ* **1**, e157 (2013).
92. Evans, S. N. & Matsen, F. A. The phylogenetic Kantorovich-Rubinstein metric for environmental sequence samples. *J. R. Stat. Soc. Ser. B Stat. Methodol.* **74**, 569–592 (2012).
93. R Core Team, R. C. T. R: A language and environment for statistical computing. (2013).
94. Wickham, H., Hester, J. & Francois, R. *readr: Read Rectangular Text Data*. (2018).
95. Wickham, H. & Bryan, J. *readxl: Read Excel Files*. (2019).
96. Wickham, H., François, R., Henry, L. & Müller, K. *dplyr: A Grammar of Data Manipulation*. (2019).
97. Wickham, H. *et al.* Welcome to the tidyverse. *J. Open Source Softw.* **4**, 1686 (2019).
98. Robinson, D. & Hayes, A. *broom: Convert Statistical Analysis Objects into Tidy Tibbles*. (2019).
99. Vu, V. Q. *ggbiplot: A ggplot2 based biplot*. (2011).
100. Pinheiro, J., Bates, D., DebRoy, S., Sarkar, D. & R. Core Team. *nlme: Linear and Nonlinear Mixed Effects Models*. (2019).
101. Wilke, C. O. *cowplot: Streamlined Plot Theme and Plot Annotations for 'ggplot2'*. (2019).
102. Kassambara, A. *ggpubr: 'ggplot2' Based Publication Ready Plots*. (2019).
103. Schauberger, P. & Walker, A. *openxlsx: Read, Write and Edit xlsx Files*. (2019).
104. Mahto, A. *splitstackshape: Stack and Reshape Datasets After Splitting Concatenated Values*. (2019).
105. Kassambara, A. *rstatix: Pipe-Friendly Framework for Basic Statistical Tests*. (2019).

106. Henry, L. & Wickham, H. *purrr: Functional Programming Tools*. (2019).

107. Dinno, A. *dunn.test: Dunn's Test of Multiple Comparisons Using Rank Sums*. (2017).

108. Auguie, B. *gridExtra: Miscellaneous Functions for 'Grid' Graphics*. (2017).

109. Bache, S. M. & Wickham, H. *magrittr: A Forward-Pipe Operator for R*. (2014).

110. Bates, D. & Maechler, M. *Matrix: Sparse and Dense Matrix Classes and Methods*. (2019).

111. Kopylova, E., Noé, L. & Touzet, H. SortMeRNA: fast and accurate filtering of ribosomal RNAs in metatranscriptomic data. *Bioinformatics* **28**, 3211–3217 (2012).

112. Truong, D. T. *et al*. MetaPhlAn2 for enhanced metagenomic taxonomic profiling. *Nat. Methods* **12**, 902–903 (2015).

113. Monahan, L. G. *et al*. High contiguity genome sequence of a multidrug-resistant hospital isolate of Enterobacter hormaechei. *Gut Pathog*. **11**, 3 (2019).

114. Kembel, S. W. *et al*. Architectural design influences the diversity and structure of the built environment microbiome. *ISME J*. **6**, 1469–1479 (2012).

115. Pajarillo, E. A. B., Chae, J. P., Kim, H. B., Kim, I. H. & Kang, D.-K. Barcoded pyrosequencing-based metagenomic analysis of the faecal microbiome of three purebred pig lines after cohabitation. *Appl. Microbiol. Biotechnol*. **99**, 5647–5656 (2015).

116. Thompson, C. L., Wang, B. & Holmes, A. J. The immediate environment during postnatal development has long-term impact on gut community structure in pigs. *ISME J*. **2**, 739 (2008).

117. Bailey, M. *et al*. Regulation of mucosal immune responses in effector sites. *Proc. Nutr. Soc*. **60**, 427–435 (2001).

118. Funkhouser, L. J. & Bordenstein, S. R. Mom knows best: the universality of maternal microbial transmission. *PLoS Biol* **11**, e1001631 (2013).

119. Ludlow, K. Australian Pesticides and Veterinary Medicines Authority. in *Encyclopedia of Nanoscience and Society* (SAGE Publications, Inc., 2010).

120. Fouhy, F. *et al*. High-Throughput Sequencing Reveals the Incomplete, Short-Term Recovery of Infant Gut Microbiota following Parenteral Antibiotic Treatment with Ampicillin and Gentamicin. *Antimicrob. Agents Chemother.* **56**, 5811–5820 (2012).

121. Zeineldin, M., Aldridge, B., Blair, B., Kancer, K. & Lowe, J. Impact of parenteral antimicrobial administration on the structure and diversity of the fecal microbiota of growing pigs. *Microb. Pathog.* **118**, 220–229 (2018).

122. Friedman, J. & Alm, E. J. Inferring Correlation Networks from Genomic Survey Data. *PLoS Comput. Biol.* **8**, e1002687 (2012).

123. Lovell, D., Pawlowsky-Glahn, V., Egozcue, J. J., Marguerat, S. & Bähler, J. Proportionality: A Valid Alternative to Correlation for Relative Data. *PLOS Comput. Biol.* **11**, e1004075 (2015).

124. von Wintzingerode, F., Göbel, U. B. & Stackebrandt, E. Determination of microbial diversity in environmental samples: pitfalls of PCR-based rRNA analysis. *FEMS Microbiol. Rev.* **21**, 213–229 (1997).

125. Han, Z., Sun, J., Lv, A. & Wang, A. Biases from different DNA extraction methods in intestine microbiome research based on 16S rDNA sequencing: a case in the koi carp, Cyprinus carpio var. Koi. *MicrobiologyOpen* **8**, e00626 (2019).

126. Moss, E. L., Maghini, D. G. & Bhatt, A. S. Complete, closed bacterial genomes from microbiomes using nanopore sequencing. *Nat. Biotechnol.* (2020) doi:10.1038/s41587-020-0422-6.

Click here to access/download;Manuscript;giga-211326.pdf

Community composition and development of the post-weaning piglet gut microbiome

D. Gaio[1], Matthew Z. DeMaere[1], Kay Anantanawat[1], Graeme J. Eamens[2], Michael Liu[1], Tiziana Zingali[1], Linda Falconer[2], Toni A. Chapman[2], Steven P. Djordjevic[1], Aaron E. Darling[1]*

[1]The ithree institute, University of Technology Sydney, Sydney, NSW, Australia

[2]NSW Department of Primary Industries, Elizabeth Macarthur Agricultural Institute, Menangle, NSW, Australia

*Corresponding author: Aaron E. Darling, aaron.darling@uts.edu.au

Keywords: shotgun metagenomics; pig; post-weaning; gut microbiome; phylogenetic diversity

ABSTRACT

We report on the largest metagenomic analysis of the pig gut microbiome to date. By processing over 800 faecal time-series samples from 126 piglets and 42 sows, we generated over 8Tbp of metagenomic shotgun sequence data. Here we describe the animal trial procedures, the generation of our metagenomic dataset and the analysis of the microbial community composition using a phylogenetic framework. We assess the effects of intramuscular antibiotic treatment and probiotic oral treatment on the diversity of gut microbial communities. We found differences between individual hosts such as breed, litter, and age, to be important contributors to variation in the community composition. Treatment effects of the antibiotic and probiotic treatments were found but were subtle, while host age was the dominant factor in shaping the gut microbiota of piglets after weaning. The post-weaning pig gut microbiome appears to follow a highly structured developmental program with characteristic post-weaning changes that can distinguish hosts that were born as little as two days apart in the second month of life.

INTRODUCTION

As the world population grows, there is an accompanying demand for animal derived products. In a semi-natural environment pig weaning occurs between the 12th and the 17th weeks from birth, whereas in the farm this typically occurs at 4 weeks of age[1]. Intensive animal husbandry and early weaning practices are commonly used to maximise production rates while minimizing costs. Both practices increase the risk of infections with pathogenic organisms, and thereby the need for antimicrobial strategies, which has included the common use of antibiotics. [2–12] Although antibiotics may kill some pathogens, the surviving bacteria can develop antimicrobial resistance (AMR) against the class of antibiotic used [13–20], as well as against other antibiotic classes [21–31]. A retrospective U.S. livestock study found evidence of multidrug resistance (resistance to >3 antimicrobial drug classes) in *Escherichia coli* that increased from 7.2% to 63.6% between the 1950s and the 2000s [32]. As the incidence of multidrug resistant (MDR) pathogens expands, the World Health Organization (WHO) has recognized AMR as one of the top health challenges of the current century [33,34].

Besides leading to AMR development, antibiotic usage is known to cause dysbiosis [34–37], the disruption of a balanced state within a gut microbial community. A balanced state decreases the chance of pathogens gaining a foothold, as there is niche and nutrient competition in play [39,40]. In livestock production, intramuscular (IM) antibiotic treatment is preferred over oral antibiotic treatment, as it is thought to contribute less to AMR than oral antibiotic treatment [41–43]. Extensive evidence exists on dysbiosis as a consequence of oral antibiotic treatment on the gut microbiome [22,35–38,44,45], whereas only a handful of studies report the effects of intramuscular (IM) antibiotic treatment on the gut microbial community [46–48], and none, to our knowledge have studied the use of IM neomycin treatment.

A number of non-antibiotic strategies to increase resistance to disease have been studied [49]. Strategies for which beneficial effects have been reported consist of hydrolases [50], fiber intake [51], β-lactamase enzymes [52], non-resistance inducing antimicrobial drugs [53,54], vaccination [55–58], phage therapy [59], in-feed organic acids [60], starches [61], and liquid feed [62–64]. Extensive evidence exists on the use of probiotics. Probiotics are reported to promote intestinal health in multiple ways: improvement of mucosal integrity [65–68], competitive exclusion against pathogenic species [50,69–73], reduction of intestinal inflammation [65,74,75] and of pathogen translocation [65,76,77]. Their efficacy has been determined, among others, in cell cultures [70,74,75,78], mice [50,67,68,73], and swine [65,66,74,76,79–83]. However, while most evidence of beneficial effects from probiotic treatment is derived from host immunity responses [65–68,74–

[76,80], host physiology [74,80–82], and pathogen relocation [50,65,69,70,72,77–79,84], fewer studies exist on the effects of probiotics on the whole gut microbial community [83,85].

This study was conducted to: 1) generate a public metagenomic databank of the gut microbiome of weaner piglets aged between 3 and 9 weeks old; 2) assess the effects of IM neomycin antibiotic use on the gut community; and 3) assess the effects of two probiotic formulations on the gut community. The data we present here is analysed from the perspective of the phylogenetic diversity of the microbial communities.

In the first results section we describe the phylogenetic diversity of the intestinal microbiome of the piglets and how it compares to the known composition of positive controls. Then, we describe evidence that highlights the importance of age, breed and litter as strongly associated factors with changes in community composition. Lastly, we describe the strong effect of time on community composition and the milder effect of antibiotic and probiotic treatments on community composition.

METHODS

Pig trial

Animal studies were conducted at the Elizabeth Macarthur Agricultural Institute (EMAI) NSW, Australia and were approved by the EMAI Ethics Committee (Approval M16/04). The trial animals comprised 4-week old male weaner pigs (*n*=126) derived from a commercial swine farm and transferred to the study facility in January 2017. These were cross-bred animals of Landrace, Duroc and Large White breeds and had been weaned at approximately 3 weeks of age.

The pig facility consisted of four environmentally controlled rooms (Rooms 1-4) with air conditioning, concrete slatted block flooring with underground drainage and open rung steel pens (**S1**). Each room had nine pens, consisting of a set of six and a set of three pens, designated a-f and g-i respectively, with the two sets of pens being physically separate, *i.e.* animals could come in contact with each other through the pen's bars within each set of pens, but not between sets. The rooms were physically separated by concrete walls and contamination between rooms was minimized by using separate equipment (boots, gloves, coveralls) for each room. In addition, under-floor drainage was flushed twice weekly and the

flushed faeces/urine was retained in under-floor channels that ran the length of the facility, so that Rooms 1, 2 were separate from Rooms 3, 4 and flushing was in the direction 1 to 2 and 3 to 4.

The pigs were fed *ad libitum* a commercial pig grower mix of 17.95% protein free of antibiotics, via self-feeders. On the day of arrival (day 1) 30, 18, 18, and 60 pigs were allocated randomly to Rooms 1, 2, 3 and 4 respectively in groups of 6, 6, 6 and 6-7 pigs per pen respectively (**S1A**). Pigs were initially weighed on day 2, and some pigs were moved between pens to achieve an initial mean pig weight per treatment of approximately 6.5 kg (range: 6.48-6.70; mean±SD: 6.53±0.08). Pigs were weighed weekly throughout the trial (**Supplementary file 1**). Behaviour and faecal consistency scores were taken daily over the 6-week period of the trial (**Supplementary file 2**). Developmental and commercial probiotic paste preparations ColiGuard® and D-Scour™ from International Animal Health, Australasia, were used in some treatment groups.

The animals were acclimatised for 2 days before the following treatments were administered: Room 1 - oral 1 g/pig of placebo paste daily for 14 d; Room 2 - oral 1 g/pig of D-Scour™ paste daily for 14 d; Room 3 - oral 1 g/pig of ColiGuard® paste daily for 14 d; Room 4 - intramuscular (IM) injection of antibiotic administered at 0.1 mL per pig daily from a 200 mg/mL solution for a total treatment duration of 5 d.

On the day following the final neomycin treatment (day 8), 36 pigs were moved from Room 4 to Room 2 (*n*=18, 6 in each pen, pens g-i), and to Room 3 (*n*=18, 6 in each pen, pens g-i) (**S1**). The following day (day 9), oral administration of D-Scour™ (1 g/pig) and of ColiGuard® (1 g/pig) commenced for pigs in Room 2 pens g-i and in Room 3 pens g-i, respectively, and continued for a period of 14 days. Assignment of the 36 neomycin-treated pigs to the treatment groups neomycin+D-Scour™ (*n*=18; Room 2 pens g-i) and neomycin+ColiGuard® (*n*=18; Room 3 pens g-i), was carried out by distributing them so that the mean weight of the animals distributed across pens and rooms was similar (**Supplementary file 1**). By this time point, each occupied pen in the trial housed six pigs. (**S1B**) From that time, twelve piglets from the original 126 were no longer present, as they had been euthanised as pre-treatment controls at the start of the trial.

Faecal samples were collected from all piglets once per week and from a subset (*n*=48 pigs; 8 from each of the six cohorts) twice per week over the 6-week study period (**Figure**

1). From each piglet, faeces were collected per rectum with new disposable gloves; where minimal or no faeces could be collected on a collection day, sampling was performed the following morning. Samples were placed in 50 mL Falcon tubes and stored at 4ºC within 30 mins of sample collection for a minimum of 30 mins and a maximum period of 6 h.

Faecal sample processing

Samples (3g/pig) were mixed with 15 mL PBS (200 mg/mL), in sterile stomacher bags and homogenized with a Bio-Rad stomacher. The homogenised samples were divided in replicates: one replicate was stored directly at -80ºC and one replicate was supplemented with glycerol (20% v/v) (Sigma-Aldrich) then stored at -80º C. In addition, single time-point faecal samples from the dams of the trial pigs (*n*=42) were obtained from the commercial facility of origin and were pre-processed at EMAI as described above. Thus, a total of 911 unique samples, between one and ten samples per subject (mean: 4.8; median: 3) (**S2**), were obtained throughout this study. At the end of the trial period, all samples were transported from EMAI to the University of Technology Sydney (UTS) for further processing.

Positive controls

As a positive control "mock community" for this study, four Gram positive (*Bacillus subtilis* strain 168, *Enterococcus faecium*, *Staphylococcus aureus* ATCC25923, *Staphylococcus epidermidis* ATCC35983) and three Gram negative (*Enterobacter hormaechei* CP_032842, *Escherchia coli* K-12 MG1655, *Pseudomonas aeruginosa* PAO1) bacterial strains from -80°C stocks were cultured at 37ºC for 16 h in LB (Luria-Bertani) then centrifuged at 14,000 rpm for 10 mins. From the resulting pellets, 1 g was transferred to 1 mL of LB and homogenised and a 1:10 dilution of this was made for each bacterial culture. Ten microliters of bacterial suspension from each of the cultures was used to determine the number of colony forming units (CFU) in the original suspension in the following manner: by further diluting tenfold in LB and by plating onto 1.6% LB agar plates and incubacted overnight. The remaining suspensions (990 μL from each bacterial culture) were pooled into a sterile tube, then aliquoted into Eppendorf tubes in 500 μL volumes/tube. As a washing step, Eppendorf tubes were centrifuged at 14,000 rpm for 10 mins, 500 μL PBS was added to the pellet and subsequently resuspended. These tubes constituted the mock community samples and were stored at -80ºC. Expected proportions of the mock community members were determined from the estimated colony forming units (CFU) multiplied by the genome

size and were as follows: 8.7:13.0:7.7:16.7:38.9:14.5:0.4 for *S. aureus*, *B. subtilis*, *E. faecium, S. epidermidis*, *P. aeruginosa*, *E. cloacae, and E.coli* respectively.

The two probiotic formulations used in this study were used as two additional positive controls. D-Scour™ is a commercially available probiotic formulation for livestock, with each gram containing 180 million CFU of the following: *Lactobacillus acidophilus*, *Lactobacillus delbrueckii* subspecies *bulgaricus*, *Lactobacillus plantarum*, *Lactobacillus rhamnosus*, *Bifidobacterium bifidum*, *Enterococcus faecium*, *Streptococcus salivarius* subspecies *thermophilus*, with an additional 20 mg of garlic extract (*Allium sativum*). The probiotic ColiGuard is a probiotic formulation developed for the treatment of entero-toxigenic *Escherichia coli* (ETEC) in weaner pigs, developed in collaboration between the NSW DPI and International Animal Health Products, containing undefined concentrations of *Lactobacillus plantarum* and *Lactobacillus salivarius*.

DNA extraction

Piglet and sow faecal samples, mock community samples, negative controls and probiotic samples (D-Scour™ and ColiGuard® paste) were allocated to a randomized block design to control for batch effects in DNA extraction and library preparation. The faecal samples were thawed on ice first, followed by the probiotics and mock community samples. MetaPolyzyme (Sigma-Aldrich) treatment was performed according to the manufacturer's instructions except for the dilution factor, which we allowed to be 4.6 times higher. Immediately after incubation, DNA extraction was performed with the MagAttract PowerMicrobiome DNA/RNA EP kit (Qiagen) according to the manufacturer's instructions. Quantification of DNA was performed using PicoGreen (Thermofisher) and measurements were performed with a plate reader (Tecan, Life Sciences) using 50 and 80 gain settings. All samples were diluted to 10 ng/μL.

Library preparation

Sample index barcode design using a previously introduced method [86] yielded a set of 96 x 8nt sequences with a 0.5 mean GC content and none of the barcodes containing 3 or more identical bases in a row. Nine hundred-sixty different combinations of i5 and i7 primers were used to create a uniquely barcoded library for each sample. The detailed sample-to-barcode assignment is given in **Supplementary file 3**. Library preparation was carried out using a modification of the Nextera Flex protocol to produce low bias, low cost shotgun libraries, as

described in a previous manuscript [86]. Following the amplification step, samples were centrifuged at 280 x *g* for 1 min and stored between 1 and 5 days at 4°C.

Size selection and purification

Samples from the same 96-well plates were pooled into one tube by taking 5 µL from each library. This generated 10 pooled samples, one for each plate. A master pool was created by pooling 5 µL from the pool of each plate into a single pool. Forty microliters from each of the 10 plate pools and 40 µL from the master pool underwent library size selection and purification using equal volumes of SPRIselect beads (Beckman Coulter) and ultrapure water (Invitrogen). Sample cleaning with SPRI-beads was performed as described previously [86]. A purified master pool comprising samples from all plates, and purified pools of individual plates to check for plate-specific anomalies, were diluted to 4 nM and fragment size distribution was assessed using the High Sensitivity DNA kit on the Bioanalyzer (Agilent Technologies, USA).

Normalisation and sequencing

The master pool was sequenced on an Illumina MiSeq v2 300 cycle nano flow cell (Illumina, USA). Read counts were obtained and used to normalise libraries. The liquid handling robot OT-One (Opentrons) was programmed to re-pool libraries based on read counts obtained from the previous MiSeq run. The script used to achieve the normalization is available through our Github repository.

The read count distribution after normalisation is displayed in supplementary figure (**S3**). The normalized and purified pooled library was sequenced on an Illumina NovaSeq S4 flow cell at the Ramaciotti Centre for Genomics (Sydney, NSW, Australia), generating a total of 27 billion read pairs from 911 samples.

Sequence data processing

Adapter trimming (parameters: k=23 hdist=1 tpe tbo mink=11), PhiX DNA removal (parameters: k=31 hdist=1), and quality filtering (parameters: ftm=0 qtrim=r trimq=20), were performed using bbduk.sh (http://jgi.doe.gov/data-and-tools/bbtools). Quality assessment of raw reads was carried out using FASTQC (http://www.bioinformatics.babraham.ac.uk/projects/fastqc/) and a combined report of all samples was obtained with MULTIQC [87] (**Supplementary File 4**). The presence of PCR

duplicates was assessed by feeding read pairs to dedupe.sh (http://jgi.doe.gov/data-and-tools/bbtools) (parameters: ac=f). Nextflow [88] was used to manage processing of the data on the HPC.

Determination of microbial diversity among samples

Phylogenetic diversity of all samples was assessed with PhyloSift [89] using the first 100k read pairs of each sample (parameters: --chunk-size 100000 --paired). In order to test for associations of phylogenetic diversity with treatment, time of sampling, and differences among hosts at the start of the trial, analysis of the unrooted phylogenetic diversity (PD) [90], the balance weighted phylogenetic diversity (BWPD) [91] and principal component analysis (PCA) of the Kantorovich-Rubinstein distances [92] (beta diversity analysis) were performed. Alpha-diversity and beta-diversity were analyzed and the results were visualized with R [93] and R packages [91–107]. The data analysis is schematically represented in **Supplementary Figure 4** (**S4**).

Additionally, SortmeRNA [111] (version 2.1) was applied to extract 16S ribosomal RNA genes from raw reads. The rRNA reference database silva-bac-16s-id90 was used. Hits were filtered (e-value <= 1e-30) and PCA was computed with R [93]. Sample counts were normalized for library size by proportions and were tested with the Spearman's Rank correlation coefficient method to find taxa correlating with the weight of the piglets across the trial.

*Batch effect*s

A randomized block design was adopted to mitigate batch effects. Because samples were distributed across ten 96-well plates during DNA extraction and library preparation, plate effects were expected. Although samples did not visibly cluster by DNA extraction plate across the first five principal components (**S5**), a batch effect was found by multiple comparison analysis with ANOVA and by applying Tukey post-hoc correction to pairwise comparisons. Batch effects were detected (ANOVA, alpha diversity: *p* range=4.8e-12-0.00011; beta diversity: *p* value<0.0023) (**S6**; **Supplementary file 5**) and removed with COMBAT[7] (ANOVA *p* value=1) (**S7**; **Supplementary file 5**).

Technical analysis of positive controls

As a quality control step, taxonomic analysis of the positive control samples and their technical replicates was performed with MetaPhlAn2 [112]. The positive controls included the mock community (*n*=8), D-Scour™ (*n*=8), and ColiGuard® (*n*=8) samples.

Data availability

The raw sequencing data has been submitted to the NCBI Short Read Archive under project PRJNA526405. Metadata and scripts are available in this article, its Supplementary material and in our Github repository https://github.com/GaioDany/metapigs_base

RESULTS

PhyloSift [89] was employed as a means to study microbial community diversity among the samples, and to test for associations with treatment, time of sampling, and differences among hosts at the start of the trial. To this end, analysis of the unrooted phylogenetic diversity (PD) [90], the balance weighted phylogenetic diversity (BWPD) [91] and principal component analysis (PCA) of the Kantorovich-Rubinstein distances [92] (beta diversity analysis) were performed.

Comparison of the expected and the observed taxonomic profile of the positive controls

All the mock community members, in seven of the eight technical replicates, were detected by MetaPhlAn2 (**S8**). One sample failed to sequence, reporting zero counts for any species. The observed mean relative abundances were as follows: *B. subtilis* (mean±SD: 2.92±0.994), *E. cloacae* (mean±SD: 38.0±6.404), *E. faecium* (mean±SD: 0.97±0.081), *E.coli* (mean±SD: 10.12±1.480), *E.coli* unclassified (mean±SD: 7.83± 1.755), *P. aeruginosa* (mean±SD: 26.72±3.026), *S. aureus* (mean±SD: 9.90±3.613), *S. epidermidis* (mean±SD: 3.54±1.435). Isolate *E. cloacae* C15117, used in this study for the make-up of the mock community, was recently found to be most closely related to the *Enterobacter hormaechei* phylogenomic group C type strain DSM 16687 and therefore re-identified as *Enterobacter hormaechei* subsp. *oharae*[113] . For this reason, taxonomic assignment by MetaPhlAn2 attributed the reads to *E. cloacae* instead. The expected proportions of the mock community members were derived from the CFU by the genome size. Based on the expected and the observed relative abundance, we found, with the exception of *S. aureus* (exp: 8.7% obs: 9.9%), three Gram positive members to be under-represented (*B. subtilis*: exp: 13.0% obs:

2.9%; *E.faecium:* exp: 7.7% obs: 1.0%; *S. epidermidis*: exp: 16.7% obs: 3.5%) and, with the exception of *P. aeruginosa* (exp: 38.9% obs: 26.8%), two Gram negative members to be over-represented (*E. cloacae:* exp: 14.5% obs: 38.0%; *E.coli*: exp: 0.4% obs: 7.8-10.1%) (**S9**). Taxonomic assignment of the mock community samples reported one contaminating species in one of the eight replicates: *Lactobacillus salivarius* (mean: 0.008) (**S10**).

The probiotic D-Scour™ is expected to contain, per gram, a total of 180 million CFU of *Lactobacillus acidophilus*, *Lactobacillus delbrueckii* subspecies *bulgaricus*, *Lactobacillus plantarum*, *Lactobacillus rhamnosus*, *Bifidobacterium bifidum*, *Enterococcus faecium*, *Streptococcus salivarius* subspecies *thermophilus* in unknown proportions. From taxonomic analysis with MetaPhlAn2, we can conclude that 6 of the 7 expected species were determined to be present in the replicates in the following mean relative abundances: *Bifidobacterium bifidum*: mean±SD: 40.01±12.558; *Enterococcus faecium*: mean±SD: 30.98±13.472; *Lactobacillus delbrueckii*: mean±SD: 11.56±7.148; *Lactobacillus plantarum*: mean±SD: 6.23±7.863; *Lactobacillus rhamnosus*: mean±SD: 2.08±1.226; *Streptococcus thermophilus*: mean±SD: 4.28±1.523. *Lactobacillus acidophilus* was not detected and *Lactobacillus helveticus* was detected instead (*Lactobacillus helveticus*: mean±SD: 4.75±2.431) (**S8**). An additional 25 taxa were detected, of which 18 and 7 were identified at the species and at the genus level, respectively. Contaminants were present at a higher concentration in three technical replicates (R3, R7, R8) with the most frequent contaminant (*Methanobrevibacter* spp.) being present in 5 of the 8 replicates (**S11**).

Taxonomic analysis of the technical replicates of the probiotic ColiGuard® also showed a species profile consistent with the expected profile, with *Lactobacillus salivarius* and *Lactobacillus plantarum* in a 9:1 ratio (*Lactobacillus salivarius*: mean±SD: 93.52±1.617; *Lactobacillus plantarum*: mean±SD: 6.10±1.134) across the replicates (**S8**). ColiGuard® contained a total of 20 contaminants, of which 16 and 4 were identified at the species and the genus level, respectively. Contaminants were present at a higher level in two technical replicates (R5, R7), with R7 displaying the most diverse and highest contamination rate (R7: 14 taxa; total contaminating reads: 2.67%; R5: 9 taxa; total contaminating reads: 0.30%). (**S12**).

Phylogenetic diversity of positive controls and how it compares to the taxonomic profile

Alpha diversity of the positive controls reflects the expected alpha diversity, with the mock community, ColiGuard® and D-Scour™ positive controls, displaying a progressively higher unrooted PD (mean±SD: Mock community: 31.53±29.50; ColiGuard®: 58.52±21.70; D-Scour™: 64.84±21.30). BWPD for the positive control ColiGuard® appears on the far left of the plot (mean±SD: 0.82±0.15), distant from the mock community (mean±SD: 1.58±0.12) and the positive control D-Scour™ (mean±SD: 1.89±0.26), which contains 2, 7 and 8 main species, respectively (**S13**; **Supplementary file 1**). The unrooted PD of the positive control ColiGuard® (mean±SD: 58.52±21.70) is closer to the unrooted PD of the Mock Community (mean±SD: 31.53±29.50) and to that of the positive control D-Scour™ (mean±SD: 64.84±21.30), than the BWPD.

The contribution to total diversity of phylogenetic tree edges with uneven quantities of reads placed on either side is down-weighted in BWPD, which is reflected in the low reported BWPD of the positive control ColiGuard®. ColiGuard® is mainly composed of two species in an uneven ratio (1:9) as by taxonomic analysis with MetaPhlAn2 (**S8**), in contrast with the mock community (0.01:1:3:4:8:10:10:27:38) and the positive control D-Scour™ (2.08:4.28:4.75:6.23:11.56:30.98:40.01) (**S8**). On the other hand, the unrooted PD reflects the absolute diversity, independently of the relative abundance of each species, within a sample. In fact, low-level contamination (<0.1%) detected in each of the positive controls (Mock Community: 1 taxon; ColiGuard®: 20 taxa; D-Scour™: 25 taxa) (**S11-13**) contributes toward the absolute diversity in the unrooted PD, erroneously inflating this value (mean±SD: Mock Community: 31.53±29.50; ColiGuard®: 58.52±21.70; D-Scour™: 64.84±21.30) (**Figure 2**).

Phylogenetic diversity of piglet gut communities immediately after weaning

The 126 piglets used in this study were derived from 4 main breed cross types "Duroc × Landrace" (n=46), "Duroc × Large White" (n=59), "Landrace × cross bred (LWxD)" (n=9), "Large White × Duroc" (n=12), and three pig lines (line 319: n=9; line 316: n=46; line 326: n=71). The piglets also differed slightly by age, being born between 1 and 6 days apart.

Based on Kruskal-Wallis one-way analysis of variance (Hommel adjusted p values to correct for multiple testing), phylogenetic diversity of the piglet samples did not cluster significantly by breed cross type ($p > 0.05$) or by pig line ($p > 0.05$) at the start of the trial, but a significant difference was found with breed and line in the fourth week of the trial

(breed: *p*=0.04; pig line: *p*=0.02) (**Figure 2**). Additionally a correlation of breed cross type was found with beta diversity principal components (PC) in the second week of the trial (PC3: *p*=0.0357) and in the fifth week of the trial (PC1: *p*=0.027). Alpha diversity significantly correlated with pig line in the fourth week of the trial (BWPD: *p*=0.017). (**Figure 2**; **Supplementary file 5**)

Notably, we found a significant correlation between alpha diversity and the age of the piglets at the first sampling time point (unrooted PD: *p*=0.0016; BWPD: *p*=0.0355) (**Figure 2**; **Supplementary file 5**). While a correlation with age was found for alpha diversity only at the start of the trial, age of the piglet was observed to be weakly associated with differences in community composition in week two (PC3: *p*=0.0507) (**S14**; **Supplementary file 5**).

As age groups were confounded with cross-breed types (*i.e.* not all age groups are represented by each of the four cross-breed types), we compared the phylogenetic diversity of age groups within each breed. As cross-breed types "Landrace × cross bred (LW × D)" and "Large White × Duroc" had only a small number of piglets in each age group, we tested for an association between phylogenetic diversity and age in cross-breeds "Duroc × Landrace" and "Duroc × Large White". In these cross-breeds, alpha and beta diversity are correlated with age in the "Duroc × Landrace" piglets (unrooted PD: *p*=0.0059; BWPD: *p*=0.0226; PC2: *p*=0.0263; PC5: *p*=0.0063) and to a lesser extent in the "Duroc × Large White" piglets (unrooted PD: *p*=0.0529; PC5: *p*=0.0310) during the first week of the trial. In the "Duroc × Landrace" piglets, a correlation between age and beta diversity was detected in week two (PC2: *p*=0.0347) (**Figure 2**; **S14**; **Supplementary file 5**). Differences in beta diversity by date of birth, within the same cross-breed (Duroc × Large white), were found to be significant (TukeyHSD adjusted *p* value: 1 day difference: 0.000782; 2 days difference: 0.018603) (**Supplementary file 5**).

Maternal effect on phylogenetic diversity

As piglets were derived from 42 distinct sows (maternal sows), and nursed by either the same or a different sow (a nurse sow) (**Supplementary File 5**), a litter effect was expected and determined. Based on Hommel adjusted *p* values, a similarity of alpha and beta phylogenetic diversity can be seen among piglets from the same maternal sow (unrooted PD: *p*=0.0096; BWPD: *p*=0.0467) and in piglets from the same nurse sow (unrooted PD: *p*=0.0320; PC1: *p*=0.0391) (**Figure 2**; **S16-19**; **Supplementary file 5**). The litter effect

described was found in samples at the start of the trial. Significance of the correlations did not persist thereafter (**Figure 2**; **Supplementary file 5**).

A strong effect of aging on phylogenetic diversity

Beta diversity analysis revealed a distinct and consistent change of the microbial community over time in all piglets, regardless of the treatment. Samples clustered in PC2 (accounting for 21.68% of the variation), showing a higher representation of *Bacteroidetes*, *Gammaproteobacteria* and *Prevotellaceae* from day 0-20, and a higher representation of *Firmicutes*, *Mollicutes* and *Ruminococcaceae* from day 20-40 (**Figure 3**; **S20**). In PC1 (accounting for 46.99% of the variation) samples shifted towards a higher representation of *Lactobacilli* from day 0 to day 20, and towards a higher representation of *Actinobacteria*, *Clostridiales* and *Mollicutes* from day 20 to day 40 (**Figure 3**; **Supplementary file 5**). A temporary shift towards a higher representation of *Bifidobacteriaceae* (*Bifidobacterium* being a component of the probiotic D-Scour™) is seen in PC5 (3.04% of the variation explained) one and two weeks from the start of the trial (**S21**).

Beta diversity analysis was performed separately for samples within each time point in order to find taxa associated with variation within each time point. Extent of variation was derived from the product of branch width by the variation explained by the principal component (**S22**). The following taxa were responsible for variation only at the start of the trial: *Gammaproteobacteria* (t0=0.12), *Enterobacteriaceae* (t0=0.08) and *Archaea* (t0=0.03). The following taxa were responsible for variation throughout the trial: *Clostridiales* (min=0.11; max=0.25), *Bacteroidetes* (min=0.06; max=0.11), *Bacteroidetes chlorobi* group (min=0.05; max=0.10). *Lactobacillus* became variable after the first week of the trial and remained highly variable throughout the trial (min=0.13; max=0.22). Some taxa contributed less to the variability among the piglets' faecal population, but they nonetheless consistently contributed to variability: *Bacteroidales* (min=0.01; max=0.04); *Firmicutes* (min=0.05; max=0.08); *Bacilli* (min=0.06; max=0.09); *Lactobacillales* (min=0.04; max=0.08); *Actinobacteria* (min=0.02; max=0.09); *Proteobacteria* (min=0.02; max=0.07). The variability of *Prevotella* (min=0.01; max=0.04) increased from the second week of the trial. (**S22**)

Taxonomic representation in terms of abundance was derived from analysis of samples with guppy fat, where abundance was derived from the branch width (**Figure 4**) and

combined with PhyloSift's taxonomic annotation of the marker gene phylogeny. Overall, *Clostridiales* were the most abundant throughout the trial (t0=0.18; t2=0.20; t4=0.11; t6=0.09; t8=0.10; t10=0.08). *Lactobacillus acidophilus* increased at the start (t0=0.00; t2=0.05; t4=0.13) then decreased (t6=0.09; t8=0.05; t10=0.07). *Subdoligranulum variabile* decreased after the first week (t0=0.05; t2=0.02) then gradually increased (t4=0.04; t6=0.05; t8=0.06; t10=0.07). Among other prevalent taxa at the start of the trial up to the second week and dropping at later time points we found *Metahobrevibacter smithii* (t0=0.06; t2=0.03) and *Bacteroidales* (t0=0.02; t2=0.01). Following an opposite trend we found *Ruminococcus* sp. JC304 (t0=0.00; t2=0.00; t4=0.01; t6=0.02; t8=0.02; t10=0.03), *Solobacterium moorei* (t0=0.00; t2=0.01; t4=0.02; t6=0.02; t8=0.02; t10=0.02) and *Prevotella copri* (t0=0.00; t2=0.00; t4=0.04; t6=0.03; t8=0.05; t10=0.02). In modest and stable abundance across the trial were *Mogibacterium* sp. CM50 (t0=0.02; t2=0.03; t4=0.02; t6=0.03; t8=0.02; t10=0.04) and *Oscillibacter* (t0=0.06; t2=0.04; t4=0.03; t6=0.03; t8=0.03; t10=0.03). In transient abundance we found *Bifidobacterium thermophilum* RBL67 (t2=0.01; t4=0.02). Gradually increasing from the second week we found *Eubacterium biforme* DSM3989 (t2=0.01; t4=0.02; t6=0.02; t8=0.02; t10=0.02), *Eubacterium rectale* (t4=0.02; t6=0.02; t8=0.03; t10=0.01) and, after the third week, *Faecalibacterium prausnitsii* (t6=0.01; t8=0.02; t10=0.02).

The effect of time was also clear in alpha diversity, where all the piglet cohorts followed a similar trend over time, independent of the treatment (**S23**; **Supplementary file 5**). Upon arrival on the trial site, the piglets' unrooted PD was lower than the sows (sows: 125.39; piglets range: 104.6-112.7) and reached a similar unrooted PD to the sows' in the second week (range: 123.4-130.7) (**Supplementary file 5**).

In comparing four timepoints at one week intervals from the start of the trial (intervals labeled as A, B, C, D), changes in alpha diversity among all the piglets were tested for and significance was determined using the Bonferroni correction. Unrooted phylogenetic diversity increased after the start of the trial (A mean: 107.86; B mean: 127.21; $p<0.001$), decreased in the following week (C mean: 122.92; p=ns) and the fourth week (D mean: 118.74; p=ns) amongst the piglets cohorts. In contrast, BWPD decreased after the start of the trial (A mean: 2.17; B mean: 2.05; $p<0.0001$), to increase in the following week (C mean: 2.14; $p<0.0001$) and decrease in the fourth week (D mean: 2.07; $p<0.01$). (**S23**; **Supplementary file 5**). The increase in unrooted PD in the first week was significant for all

treatment cohorts (*p* range: 0.0015-0.03) except ColiGuard® and neomycin (*p*=ns) cohorts. The decrease in BWPD in the first week was only significant for the control (*p*=0.04), ColiGuard® (*p*=0.0015) and neomycin+ColiGuard® (*p*=0.036) cohorts. In week two only the increase of BWPD of the control cohort reached statistical significance (*p*=0.0121). (**S24**; **Supplementary file 5**)

Effect of antibiotic and probiotic treatment on alpha diversity

We hypothesized that the probiotic treatments, whether alone (D-Scour™ and ColiGuard®) or administered after neomycin (neomycin+D-Scour™ and neomycin+ColiGuard®) would cause a change in the microbial community composition that would be measurable via phylogenetic diversity. We tested whether the treatments correlated with a change in phylogenetic diversity independently of the changes occurring with time. Given the differences in alpha and beta diversity detected among the subjects at the start of the trial, we analyzed the deltas of phylogenetic diversity instead of relying on the absolute means, similar to the procedure applied by Kembel *et al* (2012) [114]. Time-point measurements of alpha diversity were taken and deltas were computed for each piglet. Delta means were compared between cohorts, where the control cohort would serve as a control group for neomycin, D-Scour™ and ColiGuard® cohorts, whereas the neomycin cohort would serve as a control group for the neomycin+D-Scour™ and neomycin+ColiGuard® cohorts.

After the first week of the trial, the majority of the piglets displayed an increase in unrooted PD (85%) and a decrease of BWPD (75%). The following week the trend was opposite: an increase of BWPD (78%) and a decrease of unrooted PD (60%) (**Supplementary file 5**). However, the neomycin cohort displayed the smallest BWPD drop in the week following the start of the trial and the overall trend of neomycin in unrooted PD was the most different from the other cohorts (**S25**). Due to the delayed rise in unrooted PD of neomycin, significance was found in unrooted PD between neomycin and neomycin+D-Scour™ during the first week of D-Scour™ treatment (neomycin mean: 1.59; neomycin+D-Scour™ mean: -11.79; *p*= 0.012188) (**S25 B-C**) and in unrooted PD between neomycin and neomycin+ColiGuard® (neomycin mean: -5.53; neomycin+ColiGuard®: -6.70; *p*=0.043138) (**S25 C-D**). The change was opposite in BWPD (*p*=0.049263) (**S26 B-C**) and persisted during the following week, up to 3 days post- D-Scour™ treatment (neomycin mean: -6.34; neomycin+D-Scour™ mean: 4.52; BH adjusted *p*=0.024092) (**S26 B-D**).

The only significant difference between the neomycin and the control cohort was detected in BWPD in the second week of the trial (control mean: 7.10; neomycin mean: 0.28) $p=0.033751$ (**S26 B-C**).

No significant differences were detected between the control and the D-Scour™ cohort ($p<0.05$) (**Supplementary file 5**). Instead, a significant decrease in BWPD was detected in the ColiGuard® cohort between the start of the trial and the end of the ColiGuard® treatment, where nearly all piglets (88%) in the ColiGuard® cohort displayed a decrease in BWPD, whereas the control cohort was split between piglets increasing (53%) and decreasing (47%) in BWPD (control mean: 0.85; ColiGuard® mean: -5.49; $p=0.040041$) (**S26 A-C**).

Effects of antibiotic and probiotic treatment on beta diversity

To investigate the treatment effect on beta diversity, principal component analysis (PCA) of the Kantorovich-Rubenstein distances (beta diversity analysis) was performed on all samples and, additionally, on samples within individual time points. This analysis is conceptually similar to the weighted Unifrac approach for beta diversity analysis, but is designed to work with phylogenetic placement data [92]. When examining all samples together, there was no clear separation of cohorts on any of the five principal component axes. When individual time points were analysed, some clustering by cohort was observed (**S27**). D-Scour™ separated from the control cohort in PC2 (17.95%) and in PC3 (8.06%) one week and two weeks after probiotic treatment, respectively (**S27**). ColiGuard® separated from control two weeks post probiotic treatment in PC3 (7.88%). Two weeks after the end of neomycin treatment, neomycin separated from control in PC4 (5.32%), with a smaller clustering and a higher representation of *Mollicutes*. Two and four weeks after probiotic treatment, neomycin+D-Scour™ separated from neomycin in PC2 (17.74%) and PC5 (3.81%), respectively. In both timepoints and principal components, neomycin+D-Scour™ showed a higher representation of *Lactobacillales*. (**S27**)

Association between weight and community composition

Weight correlated with the abundance of certain species ($n=83$) at each time point as it resulted from principal component analysis of 16S rRNA reads. Positively correlating with weight we found among others: *Blautia* (t0), *Cetobacterium* (t0), *Ruminibacter* (t0), *Rickettsia* (t2), *Lactobacillus* (t6), *Mycoplasma* (t6), *Anaerostipes* (t8), *Ruminococcus* (t8-

t10), *Cerasibacillus* (t10). Negatively correlating with weight we found among others: *Pyramidobacter* (t0), *Odoribacter* (t2), *Schwartzia* (t6), *Streptococcus* (t6), *Dokdonella* (t8). (**S28-S33**) Significance values and correlation estimates are given in **Supplementary file 5**.

Effect of treatments on weight gain

Overall weight gain from initial to final weight (**S34 A-E**; **Supplementary file 5**) was not significantly affected by any treatment. However, the probiotic ColiGuard® was found to have a partial effect on piglet weight gain (**S34**; **Supplementary file 5**). Weight was measured weekly for a total of six measurements. Based on Tukey adjusted *p* values, a lower weight gain was detected in the ColiGuard® cohort in week 3 compared to the control cohort (p=0.0008) (**S34 C-D**; **Supplementary file 5**). Similarly, a lower weight gain was detected in the neomycin+ColiGuard® cohort in C-E compared to the neomycin cohort (p=0.0393) (**S34 C-E**; **Supplementary file 5**). Breed and age differences among piglets were not associated with weight gain (**Supplementary file 5**).

DISCUSSION

The consistent trend in community composition over time, across all the cohorts, indicates that an age-related process of ecological succession is the largest factor shaping the microbial community of post-weaning piglets, as found in this study where animals aged 20-63 days and were fed the same diet. A peak in unrooted phylogenetic diversity and drop in balance weighted phylogenetic diversity (BWPD) reflects the acquisition of new species with the loss of dominating species. This change, detected in the week following the piglets' arrival at the trial site irrespective of the cohort, could be linked to the piglets being subjected to microbial interchange (e.g.: new pen mates [115,116]) and/or to diet transition (peri-weaning transition to solid food [115,116]) leading to the reshaping of the gut microbial community. The week following the drop of BWPD, a significant increase of BWPD was recorded, reflecting the acquisition of a larger proportion of the community by the newly introduced species. The strong changes in phylogenetic diversity detected in the first and the second week could as well be attributable to other post-weaning related physiological changes, as previous studies report [44,115–117].

The highest inter-individual differences among piglets are seen in the first week of life, irrespective of maternal or environmental effects. The microbiota of 3 week old piglets is

still very dynamic, but environmental factors become evident [116]. At 6 weeks of age, CD8+ T cells infiltrate the intestinal tissue and the mucosa and intestinal lining resemble that of an adult pig [115] . In this study, piglets reached a comparable alpha diversity to the sows after the first week of the trial, at which time the piglets were aged between 3.8 and 4.6 weeks. Unrooted PD did not reach higher levels at later sampling time points. The highest BWPD accompanied by a high unrooted PD was reached after the second week of the trial when piglets were aged between 4.9 and 5.6 weeks. Age-dependent physiological changes could explain i) the major shifts we detected in alpha diversity during the first two weeks of the trial and, ii) the distinct differences in community composition with age, even with a narrow age difference between piglets (1-6 days). We were able to appreciate a significant trend of increasing unrooted PD and decreasing BWPD with age in piglets that separated a 6 days maximum by day of birth from each other. Since age groups were confounded with breeds in our study, we attempted to determine the correlation within single breeds. Unfortunately, although the correlations with age could still be detected, we could not determine the association at later time points due to the introduction of treatment effects.

Animal trials are often conducted in controlled environments so as to minimize environmental effects. However, individual variations such as breed and age are often unavoidable in large animal trials. Previously reported confounding factors include: individual variation [44], cohabitation [115,116], age [44,115,116], maternal effects [115,116,118], hormones [44], behavioural differences between breeds (e.g. coprophagy, mouth to mouth contact) and extent of long-term behavioural adaptation, which can differ between breeds for reasons not attributable to genetics [44,115,116]. A litter effect was found in piglets at the start of the trial and was lost at later time points during the trial. This could be due to either of the aforementioned factors. Co-housing, aging and the splitting of the piglets in separate rooms to receive a different treatment, are possible causes for loss of the litter effect. In this study we confirm the importance of these factors in the contribution to inter-individual variability of gut microbial composition. Motta *et al* (2019) report a correlation of beta diversity with age and no correlation of genotype and litter effect with either alpha or beta diversity [9] . On the contrary, we found the piglet samples to significantly cluster by litter, breed and by age up to the second and the fourth week post weaning, in alpha diversity and beta diversity, respectively. We conclude that even small age differences among post- weaning piglets, down to the day, must be accounted for in an experimental set up.

Three groups of piglets (cohorts neomycin, neomycin+D-Scour™ and neomycin+ColiGuard®) underwent 5 days of treatment with the broad-spectrum antibiotic neomycin, via intramuscular administration. Intramuscular neomycin poorly diffuses (<10%) into a healthy gastrointestinal tract [119] , therefore a direct effect of neomycin on the gut microbiome may not be expected. However, neomycin showed a different trend in unrooted PD between the second and the third week of the trial, corresponding to the week following the neomycin treatment period for the neomycin cohort. Taking this time frame into consideration, the neomycin cohort did not increase in BWPD to the extent of the Control cohort. Although statistically significant differences between neomycin and Control in alpha diversity were not reached, the BWPD of the neomycin cohort appears to follow a different trend to the Control from the first week (during neomycin treatment) where neomycin treated piglets show the lowest decrease of BWPD compared to the control cohort and all other cohorts. While all cohorts show an increase in absolute phylogenetic diversity accompanied by a decrease of diversity evenness during this time frame, the neomycin cohort piglets show a lower drop in BWPD, suggesting an increase of species richness, without a corresponding loss of species evenness. Furthermore the neomycin cohort significantly separated from the control cohort in beta diversity in the third week of the trial, showing a higher representation of *Mollicutes*. Numerous studies report the link of oral antibiotic use with dysbiosis [22,35–38,44,45], as well as with host physiology changes [37]. On the contrary, the effect of intramuscular antibiotic administration on the microbiome is less well investigated. Correlation between intramuscular antibiotic use and dysbiosis has been reported in fish [46], gorillas [47], humans [120], and pigs [48,121]. In 1 day old piglets, a single IM injection of amoxicillin (penicillin class) is reported to have an effect on the intestinal microbiota, detectable 40 days post treatment [48]. Zeineldin *et al* (2018) tested the effects of IM administration of several antibiotics of various classes (penicillin, macrolide, cephalosporin and tetracycline), in 8-week old piglets, reporting shifts of the *Firmicutes*/*Bacteroidetes* ratio following treatment (length of the treatment not reported)[121]. The effects of intramuscular administration of neomycin (aminoglycoside class) on the gut microbiota have to our knowledge not been investigated. Based on our results we conclude that a mild effect on phylogenetic diversity is appreciable post IM neomycin treatment, up to two weeks after termination of the treatment. Additional compositional and functional analysis is necessary to determine the source of this mild variation. Differences were not detected at later time points, based on our phylogenetic

diversity analyses, suggesting a full recovery of the microbial communities after two week from the end of the treatment.

It is possible that the large shifts in phylogenetic diversity taking place in the first two weeks irrespective of the treatment (an increase, then decrease of unrooted PD, and an opposite trend of BWPD) have masked the milder effects of the treatment, despite our efforts to control for the effects of aging. This could be the reason why a significantly distinct alpha diversity trend was found in the neomycin+D-Scour™ cohort compared to the neomycin cohort, but not in the D-Scour™ cohort compared to the Control cohort. The neomycin+D-Scour™ cohort underwent 5 days of neomycin treatment followed by 2 weeks of D-Scour™ treatment. A significant increase of BWPD was detected in the two-week period of D-Scour™ treatment, indicating a possible enhancement of microbiome evenness following neomycin treatment. To our knowledge there are no studies reporting an increased evenness in piglet gut community composition following a specific probiotic treatment. There are instead multiple studies reporting beneficial effects of probiotic treatment in sucker and weaner piglets in terms of improved gut mucosal integrity [66,80], growth rate [80–83], digestibility of proteins and water absorption [80,83], reduction of pathogen invasion efficiency [76,79,80], and decreased mortality [80,82]. Although the assessment of physiologic changes from probiotic treatments was outside the scope of this study, we found significant separation of neomycin+D-Scour™ cohort samples to neomycin cohort samples in beta diversity 3 and 10 days after D-Scour™ treatment, where neomycin+D-Scour™ samples showed a higher representation of *Lactobacillales* compared to neomycin samples, suggesting a transient establishment of the probiotic strains in the piglet guts.

The second probiotic in this study, ColiGuard®, did not have an effect on alpha diversity, but clustering was detected in beta diversity, where ColiGuard® samples separated from Control cohort samples in the third principal component (explaining 7.88% of the variation) two weeks post probiotic treatment. Additionally, the ColiGuard® treatment correlated with a lower weight gain, whether or not it was preceded by the antibiotic treatment. However, when comparing the overall weight gain (from the start to the end of the trial) the weight gain in the cohorts receiving ColiGuard® did not differ from the other cohorts.

We extracted the 16S rRNA gene hypervariable regions from our dataset, obtained the counts, and ran a correlation analysis to discover taxa that correlated with the weight of the

piglets. As a consequence of the library size normalization step, the use of correlation with compositional data can inflate the false discovery rate [122,123]. For this reason it can be expected that some of the taxa we found to correlate with the weight of the piglets (eighty-three distinct species) could be spurious while other correlations may have been missed.

Technical controls in metagenomic studies and methodological limitations

Taxonomic assignment of the raw reads from the positive controls was performed with MetaPhlAn2[112] which relies on a ca. 1M unique clade-specific markers derived from 17,000 reference genomes. Such a database to map against the positive controls suffices as these organisms are cultivable, and for this reason they are widely studied hence the sequences are known. This is not the case for real-world samples where mapping against a database (which completeness relies on studied and often cultivable organisms) would narrow the view on the true diversity within the sample.

Positive controls with well-studied members and known ratios within the samples, has proven to be a valuable tool to assess consistency among technical replicates across batches and to detect possible biases derived from the DNA extraction method.

Systematic taxonomic bias in microbiome studies, resulting from differences in cell wall structures between Gram positive and Gram negative bacteria, have previously been reported; sample treatment with enzymatic cocktails can modestly reduce this bias [124–126]. Although we implemented this step in our workflow, it seems that, from the read abundance of our mock community, which contained three Gram negative and four Gram positive strains, a bias towards Gram negative taxa may still be present.

In terms of contamination we concluded that: a) contamination in our study was not batch specific; b) a problem of sample cross-contamination existed at the DNA extraction step between neighbouring wells. During the bead beating step of DNA extraction, the deep-well plate is sealed with a sealing mat, rotated and placed in a plate shaker for the bead beating to take place. We consider that sample cross-contamination is most likely to occur during this step.

Conclusion

Our study provides a publicly-available databank of the pig gut metagenome. Our findings further stress the importance of confounding factors such as breed, age and maternal effects when assessing the effect of treatment on the gut microbiome. We found that age, even within a narrow age span (1-6 days) can have an impact on microbial shifts and should be accounted for in microbiome studies. Intramuscular neomycin treatment correlated with a clustering in alpha diversity and a higher representation of *Mollicutes* compared to control. D-Scour™ treated piglets displayed a mild shift in alpha diversity compared to control, and a transient establishment of *Lactobacillales*. ColiGuard® treated piglets displayed a clustering in beta diversity and a transient lower weight gain compared to control. Weight correlated with the abundance of a number of taxa. Age was the strongest factor shaping phylogenetic diversity of the piglets.

As previously mentioned, phylogenetic diversity is based on distinct taxa (richness) and their collective structure (proportions reflected by BWPD) and not on a direct assessment of composition and function. These types of analyses will be necessary to further describe the effects of the treatments.

Declarations

Competing Interests

D-Scour™ was sourced from International Animal Health Products (IAHP). ColiGuard® was developed in a research project with NSW DPI, IAHP and AusIndustry Commonwealth government funding.

Authors Contributions

Pig Trial: TC, LF, DG, TZ, GJE, AED, SPD

DNA extraction: DG, ML

Library prep, robot pooling: DG, ML, KA, AED

Sequencing data processing: MZD, DG, AED

Data analysis: DG, AED

Manuscript: DG

Manuscript editing: DG, AED, GJE, John Webster

Funding

This work was supported by the Australian Research Council, linkage grant LP150100912. This project was funded by the Australian Centre for Genomic Epidemiological Microbiology (Ausgem), a collaborative partnership between the NSW Department of Primary Industries and the University of Technology Sydney. TZ and DG are recipients of UTS International Research and UTS President's Scholarships. NSW DPI approved the paper before submission for publication.

Acknowledgements

We would like to thank the on site team of the pig trial: Graeme Eamens, Linda Falconer, Shayne Fell, Tiziana Zingali, Toni Chapman. Thank you Amy Bottomley, Giulia Ballerin, Rosy Cavaliere, for providing the mock community strains. Thank you Akane Tanaka, Leigh Monahan and Joyce To for the technical support. Thank you John Webster for providing DPI internal review of this manuscript and for the helpful suggestion about its content. International Animal Health Products for providing access to the probiotic supplements and the Australian Centre for Genomic Epidemiological Microbiology (Ausgem) for financially supporting this study.

Supplementary Files

Supplementary File 1. (animal details)

Supplementary File 2. (metadata)

Supplementary File 3. (barcodes)

Supplementary File 4. (multiqc report)

Supplementary file 5. (stats output)

REFERENCES

1. Jensen, P. & Recén, B. When to wean — Observations from free-ranging domestic pigs. *Appl. Anim. Behav. Sci.* **23**, 49–60 (1989).
2. Van Heugten, E., Coffey, M. T. & Spears, J. W. Effects of immune challenge, dietary energy density, and source of energy on performance and immunity in weanling pigs. *J. Anim. Sci.* **74**, 2431–2440 (1996).
3. Blecha, F. Immune system response to stress. *Biol. Anim. Stress Basic Princ. Implic. Anim. Welf. Moberg GP Mench JA Eds* 111–123 (2000).
4. Fairbrother, J. M., Nadeau, É. & Gyles, C. L. Escherichia coli in postweaning diarrhea in pigs: an update on bacterial types, pathogenesis, and prevention strategies. *Anim. Health Res. Rev.* **6**, 17–39 (2005).
5. Faubert, C. & Drolet, R. Hemorrhagic gastroenteritis caused by Escherichia coli in piglets: clinical, pathological and microbiological findings. *Can. Vet. J.* **33**, 251 (1992).
6. Nabuurs, M. J. A. Weaning piglets as a model for studying pathophysiology of diarrhea. *Vet. Q.* **20**, 42–45 (1998).
7. Lallès, J.-P., Bosi, P., Smidt, H. & Stokes, C. R. Weaning — A challenge to gut physiologists. *Livest. Sci.* **108**, 82–93 (2007).
8. Heo, J. M. *et al*. Gastrointestinal health and function in weaned pigs: a review of feeding strategies to control post-weaning diarrhoea without using in-feed antimicrobial compounds. *J. Anim. Physiol. Anim. Nutr.* **97**, 207–237 (2013).
9. Motta, V., Luise, D., Bosi, P. & Trevisi, P. Faecal microbiota shift during weaning transition in piglets and evaluation of AO blood types as shaping factor for the bacterial community profile. *PloS One* **14**, e0217001 (2019).

10. Alain B. Pajarillo, E., Chae, J.-P., P. Balolong, M., Bum Kim, H. & Kang, D.-K. Assessment of fecal bacterial diversity among healthy piglets during the weaning transition. *J. Gen. Appl. Microbiol.* **60**, 140–146 (2014).

11. Kaper, J. B., Nataro, J. P. & Mobley, H. L. T. Pathogenic Escherichia coli. *Nat. Rev. Microbiol.* **2**, 123–140 (2004).

12. Jordan, D. *et al.* Antimicrobial use in the Australian pig industry: results of a national survey. *Aust. Vet. J.* **87**, 222–229 (2009).

13. Zhu, Y.-G. *et al.* Diverse and abundant antibiotic resistance genes in Chinese swine farms. *Proc. Natl. Acad. Sci.* **110**, 3435–3440 (2013).

14. Erik, K. & Knudsen, B. Development of antibiotic resistance and options to replace antimicrobials in animal diets. *Proc. Nutr. Soc.* **60**, 291–299 (2001).

15. Levy, S. B. & Marshall, B. Antibacterial resistance worldwide: causes, challenges and responses. *Nat. Med.* **10**, S122 (2004).

16. White, D. G. Antimicrobial resistance in pathogenic Escherichia coli from animals. in *Antimicrobial resistance in bacteria of animal origin* 145–166 (American Society of Microbiology, 2006).

17. Broens, E. M., Graat, E. A. M., Van der Wolf, P. J., Van de Giessen, A. W. & De Jong, M. C. M. Prevalence and risk factor analysis of livestock associated MRSA-positive pig herds in The Netherlands. *Prev. Vet. Med.* **102**, 41–49 (2011).

18. Modi, S. R., Lee, H. H., Spina, C. S. & Collins, J. J. Antibiotic treatment expands the resistance reservoir and ecological network of the phage metagenome. *Nature* **499**, 219–222 (2013).

19. Prescott, J. F. History of antimicrobial usage in agriculture: an overview. in *Antimicrobial resistance in bacteria of animal origin* 19–28 (American Society of Microbiology, 2006).

20. Marshall, B. M. & Levy, S. B. Food animals and antimicrobials: impacts on human health. *Clin. Microbiol. Rev.* **24**, 718–733 (2011).

21. Callens, B. *et al.* Presence of antimicrobial resistance and antimicrobial use in sows are risk factors for antimicrobial resistance in their offspring. *Microb. Drug Resist.* **21**, 50–58 (2015).

22. Looft, T. P. *The swine intestinal microbiota: localized adaptations and responses to in-feed antibiotics*. (Iowa State University, 2012).

23. Salyers, A. A. & Shoemaker, N. B. Resistance gene transfer in anaerobes: new insights, new problems. *Clin. Infect. Dis.* **23**, S36–S43 (1996).

24. Salyers, A. A. & Amábile-Cuevas, C. F. Why are antibiotic resistance genes so resistant to elimination? *Antimicrob. Agents Chemother.* **41**, 2321–2325 (1997).

25. Dorado-García, A. *et al.* Dose-Response Relationship between Antimicrobial Drugs and Livestock-Associated MRSA in Pig Farming1. *Emerg. Infect. Dis.* **21**, 950–959 (2015).

26. Didelot, X. & Maiden, M. C. J. Impact of recombination on bacterial evolution. *Trends Microbiol.* **18**, 315–322 (2010).

27. Canchaya, C., Fournous, G., Chibani-Chennoufi, S., Dillmann, M.-L. & Brüssow, H. Phage as agents of lateral gene transfer. *Curr. Opin. Microbiol.* **6**, 417–424 (2003).

28. Claverys, J.-P., Martin, B. & Polard, P. The genetic transformation machinery: composition, localization, and mechanism. *FEMS Microbiol. Rev.* **33**, 643–656 (2009).

29. Ravi, A., Avershina, E., Ludvigsen, J., L'Abée-Lund, T. & Rudi, K. Integrons in the Intestinal Microbiota as Reservoirs for Transmission of Antibiotic Resistance Genes. *Pathogens* **3**, 238–248 (2014).

30. Porse, A., Schønning, K., Munck, C. & Sommer, M. O. A. Survival and Evolution of a Large Multidrug Resistance Plasmid in New Clinical Bacterial Hosts. *Mol. Biol. Evol.* **33**, 2860–2873 (2016).

31. Bednorz, C. *et al.* The broader context of antibiotic resistance: Zinc feed supplementation of piglets increases the proportion of multi-resistant Escherichia coli in vivo. *Int. J. Med. Microbiol.* **303**, 396–403 (2013).

32. Tadesse, D. A. *et al.* Antimicrobial drug resistance in Escherichia coli from humans and food animals, United States, 1950–2002. *Emerg. Infect. Dis.* **18**, 741 (2012).

33. U. S. Food & Drug, A. FDA Task Force on Antimicrobial Resistance: Key Recommendations and Report. (2000).

34. Centers for Disease Control & Prevention. Get smart: know when antibiotics work. Centers for Disease Control and Prevention, Atlanta, GA. (2010).

35. Dethlefsen, L. & Relman, D. A. Incomplete recovery and individualized responses of the human distal gut microbiota to repeated antibiotic perturbation. *Proc. Natl. Acad. Sci.* **108**, 4554–4561 (2011).

36. Cho, I. *et al.* Antibiotics in early life alter the murine colonic microbiome and adiposity. *Nature* **488**, 621–626 (2012).

37. Antunes, L. C. M. *et al.* Effect of Antibiotic Treatment on the Intestinal Metabolome. *Antimicrob. Agents Chemother.* **55**, 1494–1503 (2011).

38. Gonzalez-Perez, G. *et al.* Maternal Antibiotic Treatment Impacts Development of the Neonatal Intestinal Microbiome and Antiviral Immunity. *J. Immunol.* **196**, 3768–3779 (2016).

39. Sachs, J. L., Mueller, U. G., Wilcox, T. P. & Bull, J. J. The evolution of cooperation. *Q. Rev. Biol.* **79**, 135–160 (2004).

40. Foster, K. R. & Wenseleers, T. A general model for the evolution of mutualisms. *J. Evol. Biol.* **19**, 1283–1293 (2006).

41. Birck, M. M. *et al.* Enteral but not parenteral antibiotics enhance gut function and prevent necrotizing enterocolitis in formula-fed newborn preterm pigs. *Am. J. Physiol.-Gastrointest. Liver Physiol.* **310**, G323–G333 (2016).

42. Wiuff, C., Lykkesfeldt, J., Svendsen, O. & Aarestrup, F. M. The effects of oral and intramuscular administration and dose escalation of enrofloxacin on the selection of quinolone resistance among Salmonella and coliforms in pigs. *Res. Vet. Sci.* **75**, 185–193 (2003).

43. Chantziaras, I., Smet, A., Haesebrouck, F., Boyen, F. & Dewulf, J. Studying the effect of administration route and treatment dose on the selection of enrofloxacin resistance in commensal Escherichia coli in broilers. *J. Antimicrob. Chemother.* **72**, 1991–2001 (2017).

44. Xiao, L. *et al.* A reference gene catalogue of the pig gut microbiome. *Nat. Microbiol.* **1**, 16161 (2016).

45. Deshmukh, H. S. *et al.* The microbiota regulates neutrophil homeostasis and host resistance to Escherichia coli K1 sepsis in neonatal mice. *Nat. Med.* **20**, 524–530 (2014).

46. Kim, A. *et al.* Administration of antibiotics can cause dysbiosis in fish gut. *Aquaculture* **512**, 734330 (2019).

47. Vlčková, K. *et al.* Effect of Antibiotic Treatment on the Gastrointestinal Microbiome of Free-Ranging Western Lowland Gorillas (Gorilla g. gorilla). *Microb. Ecol.* **72**, 943–954 (2016).

48. Janczyk, P. *et al*. Parenteral long-acting amoxicillin reduces intestinal bacterial community diversity in piglets even 5 weeks after the administration. *ISME J.* **1**, 180–183 (2007).

49. Allen, H. K., Levine, U. Y., Looft, T., Bandrick, M. & Casey, T. A. Treatment, promotion, commotion: antibiotic alternatives in food-producing animals. *Trends Microbiol.* **21**, 114–119 (2013).

50. Rangan, K. J. *et al*. A secreted bacterial peptidoglycan hydrolase enhances tolerance to enteric pathogens. *science* **353**, 1434–1437 (2016).

51. Tap, J. *et al*. Gut microbiota richness promotes its stability upon increased dietary fibre intake in healthy adults. *Environ. Microbiol.* **17**, 4954–4964 (2015).

52. Kaleko, M. *et al*. Development of SYN-004, an oral beta-lactamase treatment to protect the gut microbiome from antibiotic-mediated damage and prevent Clostridium difficile infection. *Anaerobe* (2016) doi:10.1016/j.anaerobe.2016.05.015.

53. Song, J., Li, Y. li & Hu, C. hong. Effects of copper-exchanged montmorillonite, as alternative to antibiotic, on diarrhea, intestinal permeability and proinflammatory cytokine of weanling pigs. *Appl. Clay Sci.* **77–78**, 52–55 (2013).

54. Holler, E. *et al*. Metagenomic Analysis of the Stool Microbiome in Patients Receiving Allogeneic Stem Cell Transplantation: Loss of Diversity Is Associated with Use of Systemic Antibiotics and More Pronounced in Gastrointestinal Graft-versus-Host Disease. *Biol. Blood Marrow Transplant.* **20**, 640–645 (2014).

55. Nogueira, M., Collins, A., Dunlop, R. & Emery, D. Effect of the route of administration on the mucosal and systemic immune responses to *Lawsonia intracellularis* vaccine in pigs. *Aust. Vet. J.* **93**, 124–126 (2015).

56. Bak, H. & Rathkjen, P. H. Reduced use of antimicrobials after vaccination of pigs against porcine proliferative enteropathy in a Danish SPF herd. *Acta Vet. Scand.* **51**, 1 (2009).

57. Francis, D. H. & Willgohs, J. A. Evaluation of a live avirulent Escherichia coli vaccine for K88+, LT+ enterotoxigenic colibacillosis in weaned pigs. *Am. J. Vet. Res.* **52**, 1051–1055 (1991).

58. Morgan, R. L., Isaacson, R. E., Moon, H. W., Brinton, C. C. & To, C. C. Immunization of suckling pigs against enterotoxigenic Escherichia coli-induced diarrheal disease by vaccinating dams with purified 987 or K99 pili: protection correlates with pilus homology of vaccine and challenge. *Infect. Immun.* **22**, 771–777 (1978).

59. Callaway, T. R. *et al.* Evaluation of Phage Treatment as a Strategy to Reduce *Salmonella* Populations in Growing Swine. *Foodborne Pathog. Dis.* **8**, 261–266 (2011).

60. Willamil, J., Creus, E., Francisco Pérez, J., Mateu, E. & Martín-Orúe, S. M. Effect of a microencapsulated feed additive of lactic and formic acid on the prevalence of Salmonella in pigs arriving at the abattoir. *Arch. Anim. Nutr.* **65**, 431–444 (2011).

61. Brouns, F., Kettlitz, B. & Arrigoni, E. Resistant starch and "the butyrate revolution". *Trends Food Sci. Technol.* **13**, 251–261 (2002).

62. Missotten, J. A. M., Michiels, J., Ovyn, A., De Smet, S. & Dierick, N. A. Fermented liquid feed for pigs. *Arch. Anim. Nutr.* **64**, 437–466 (2010).

63. Canibe, N. & Jensen, B. B. Fermented and nonfermented liquid feed to growing pigs: effect on aspects of gastrointestinal ecology and growth performance. *J. Anim. Sci.* **81**, 2019–2031 (2003).

64. Moran, C. A. Development and benefits of liquid diets for newly weaned pigs. *PhD Univ. Plymouth* (2001).

65. Lessard, M. *et al*. Administration of Pediococcus acidilactici or Saccharomyces cerevisiae boulardii modulates development of porcine mucosal immunity and reduces intestinal bacterial translocation after Escherichia coli challenge1, 2. *J. Anim. Sci*. **87**, 922 (2009).

66. Deng, J., Li, Y., Zhang, J. & Yang, Q. Co-administration of Bacillus subtilis RJGP16 and Lactobacillus salivarius B1 strongly enhances the intestinal mucosal immunity of piglets. *Res. Vet. Sci*. **94**, 62–68 (2013).

67. Garrido-Mesa, N. *et al*. The association of minocycline and the probiotic Escherichia coli Nissle 1917 results in an additive beneficial effect in a DSS model of reactivated colitis in mice. *Biochem. Pharmacol*. **82**, 1891–1900 (2011).

68. Ukena, S. N. *et al*. Probiotic Escherichia coli Nissle 1917 Inhibits Leaky Gut by Enhancing Mucosal Integrity. *PloS One* **2**, e1308 (2007).

69. Deriu, E. *et al*. Probiotic bacteria reduce salmonella typhimurium intestinal colonization by competing for iron. *Cell Host Microbe* **14**, 26–37 (2013).

70. Hancock, V., Dahl, M. & Klemm, P. Probiotic Escherichia coli strain Nissle 1917 outcompetes intestinal pathogens during biofilm formation. *J. Med. Microbiol*. **59**, 392–399 (2010).

71. Heyman, M. Effect of lactic acid bacteria on diarrheal diseases. *J. Am. Coll. Nutr*. **19**, 137S-146S (2000).

72. Jin, L.-Z., Marquardt, R. R. & Baidoo, S. K. Inhibition of enterotoxigenic Escherichia coli K88, K99 and 987P by the Lactobacillus isolates from porcine intestine. *J Sci Food Agric* 6 (2000).

73. Maltby, R., Leatham-Jensen, M. P., Gibson, T., Cohen, P. S. & Conway, T. Nutritional basis for colonization resistance by human commensal Escherichia coli strains HS and

Nissle 1917 against E. coli O157: H7 in the mouse intestine. *PloS One* **8**, e53957 (2013).

74. Suda, Y. *et al*. Immunobiotic Lactobacillus jensenii as immune-health promoting factor to improve growth performance and productivity in post-weaning pigs. *BMC Immunol.* **15**, 24 (2014).

75. Shimazu, T. *et al*. Immunobiotic Lactobacillus jensenii Elicits Anti-Inflammatory Activity in Porcine Intestinal Epithelial Cells by Modulating Negative Regulators of the Toll-Like Receptor Signaling Pathway. *Infect. Immun.* **80**, 276–288 (2012).

76. Splichalova, A. *et al*. Interference of Bifidobacterium choerinum or Escherichia coli Nissle 1917 with Salmonella Typhimurium in gnotobiotic piglets correlates with cytokine patterns in blood and intestine. *Clin. Exp. Immunol.* **163**, 242–249 (2011).

77. Roselli, M. *et al*. The novel porcine Lactobacillus sobrius strain protects intestinal cells from enterotoxigenic Escherichia coli K88 infection and prevents membrane barrier damage. *J. Nutr.* **137**, 2709–2716 (2007).

78. Altenhoefer, A. *et al*. The probiotic Escherichia coli strain Nissle 1917 interferes with invasion of human intestinal epithelial cells by different enteroinvasive bacterial pathogens. *FEMS Immunol. Med. Microbiol.* **40**, 223–229 (2004).

79. Šmajs, D. *et al*. Experimental Administration of the Probiotic Escherichia coli Strain Nissle 1917 Results in Decreased Diversity of E. coli Strains in Pigs. *Curr. Microbiol.* **64**, 205–210 (2012).

80. Valeriano, V. D. V., Balolong, M. P. & Kang, D. K. Probiotic roles of Lactobacillus sp. in swine: insights from gut microbiota. *J. Appl. Microbiol.* **122**, 554–567 (2017).

81. Abe, F., Ishibashi, N. & Shimamura, S. Effect of administration of bifidobacteria and lactic acid bacteria to newborn calves and piglets. *J. Dairy Sci.* **78**, 2838–2846 (1995).

82. Davis, M. E. *et al.* Effect of a Bacillus-based direct-fed microbial feed supplement on growth performance and pen cleaning characteristics of growing-finishing pigs. *J. Anim. Sci.* **86**, 1459–1467 (2008).

83. Chu, G. M., Lee, S. J., Jeong, H. S. & Lee, S. S. Efficacy of probiotics from anaerobic microflora with prebiotics on growth performance and noxious gas emission in growing pigs: SYNBIOTICS ON NOXIOUS GAS EMISSION IN PIGS. *Anim. Sci. J.* **82**, 282–290 (2011).

84. Kim, P. I. *et al.* Probiotic properties of Lactobacillus and Bifidobacterium strains isolated from porcine gastrointestinal tract. *Appl. Microbiol. Biotechnol.* **74**, 1103–1111 (2007).

85. Takahashi, S., Egawa, Y., Simojo, N., Tsukahara, T. & Ushida, K. Oral administration of Lactobacillus plantarum strain Lq80 to weaning piglets stimulates the growth of indigenous lactobacilli to modify the lactobacillal population. *J. Gen. Appl. Microbiol.* **53**, 325–332 (2007).

86. Gaio, D. *et al.* Hackflex: low cost Illumina sequencing library construction for high sample counts. *bioRxiv* 779215 (2019).

87. Ewels, P., Magnusson, M., Lundin, S. & Käller, M. MultiQC: summarize analysis results for multiple tools and samples in a single report. *Bioinformatics* **32**, 3047–3048 (2016).

88. Ewels, P. A. *et al.* nf-core *: Community curated bioinformatics pipelines*. (2019).

89. Darling, A. E. *et al.* PhyloSift: phylogenetic analysis of genomes and metagenomes. *PeerJ* **2**, e243 (2014).

90. Faith, D. P. Conservation evaluation and phylogenetic diversity. *Biol. Conserv.* **61**, 1–10 (1992).

91. McCoy, C. O. & Matsen, F. A. Abundance-weighted phylogenetic diversity measures distinguish microbial community states and are robust to sampling depth. *PeerJ* **1**, e157 (2013).

92. Evans, S. N. & Matsen, F. A. The phylogenetic Kantorovich-Rubinstein metric for environmental sequence samples. *J. R. Stat. Soc. Ser. B Stat. Methodol.* **74**, 569–592 (2012).

93. R Core Team, R. C. T. R: A language and environment for statistical computing. (2013).

94. Wickham, H., Hester, J. & Francois, R. *readr: Read Rectangular Text Data*. (2018).

95. Wickham, H. & Bryan, J. *readxl: Read Excel Files*. (2019).

96. Wickham, H., François, R., Henry, L. & Müller, K. *dplyr: A Grammar of Data Manipulation*. (2019).

97. Wickham, H. *et al*. Welcome to the tidyverse. *J. Open Source Softw.* **4**, 1686 (2019).

98. Robinson, D. & Hayes, A. *broom: Convert Statistical Analysis Objects into Tidy Tibbles*. (2019).

99. Vu, V. Q. *ggbiplot: A ggplot2 based biplot*. (2011).

100. Pinheiro, J., Bates, D., DebRoy, S., Sarkar, D. & R. Core Team. *nlme: Linear and Nonlinear Mixed Effects Models*. (2019).

101. Wilke, C. O. *cowplot: Streamlined Plot Theme and Plot Annotations for 'ggplot2'*. (2019).

102. Kassambara, A. *ggpubr: 'ggplot2' Based Publication Ready Plots*. (2019).

103. Schauberger, P. & Walker, A. *openxlsx: Read, Write and Edit xlsx Files*. (2019).

104. Mahto, A. *splitstackshape: Stack and Reshape Datasets After Splitting Concatenated Values*. (2019).

105. Kassambara, A. *rstatix: Pipe-Friendly Framework for Basic Statistical Tests*. (2019).

106. Henry, L. & Wickham, H. *purrr: Functional Programming Tools*. (2019).
107. Dinno, A. *dunn.test: Dunn's Test of Multiple Comparisons Using Rank Sums*. (2017).
108. Auguie, B. *gridExtra: Miscellaneous Functions for 'Grid' Graphics*. (2017).
109. Bache, S. M. & Wickham, H. *magrittr: A Forward-Pipe Operator for R*. (2014).
110. Bates, D. & Maechler, M. *Matrix: Sparse and Dense Matrix Classes and Methods*. (2019).
111. Kopylova, E., Noé, L. & Touzet, H. SortMeRNA: fast and accurate filtering of ribosomal RNAs in metatranscriptomic data. *Bioinformatics* **28**, 3211–3217 (2012).
112. Truong, D. T. *et al*. MetaPhlAn2 for enhanced metagenomic taxonomic profiling. *Nat. Methods* **12**, 902–903 (2015).
113. Monahan, L. G. *et al*. High contiguity genome sequence of a multidrug-resistant hospital isolate of Enterobacter hormaechei. *Gut Pathog*. **11**, 3 (2019).
114. Kembel, S. W. *et al*. Architectural design influences the diversity and structure of the built environment microbiome. *ISME J*. **6**, 1469–1479 (2012).
115. Pajarillo, E. A. B., Chae, J. P., Kim, H. B., Kim, I. H. & Kang, D.-K. Barcoded pyrosequencing-based metagenomic analysis of the faecal microbiome of three purebred pig lines after cohabitation. *Appl. Microbiol. Biotechnol*. **99**, 5647–5656 (2015).
116. Thompson, C. L., Wang, B. & Holmes, A. J. The immediate environment during postnatal development has long-term impact on gut community structure in pigs. *ISME J*. **2**, 739 (2008).
117. Bailey, M. *et al*. Regulation of mucosal immune responses in effector sites. *Proc. Nutr. Soc*. **60**, 427–435 (2001).
118. Funkhouser, L. J. & Bordenstein, S. R. Mom knows best: the universality of maternal microbial transmission. *PLoS Biol* **11**, e1001631 (2013).

119. Ludlow, K. Australian Pesticides and Veterinary Medicines Authority. in *Encyclopedia of Nanoscience and Society* (SAGE Publications, Inc., 2010).

120. Fouhy, F. *et al*. High-Throughput Sequencing Reveals the Incomplete, Short-Term Recovery of Infant Gut Microbiota following Parenteral Antibiotic Treatment with Ampicillin and Gentamicin. *Antimicrob. Agents Chemother.* **56**, 5811–5820 (2012).

121. Zeineldin, M., Aldridge, B., Blair, B., Kancer, K. & Lowe, J. Impact of parenteral antimicrobial administration on the structure and diversity of the fecal microbiota of growing pigs. *Microb. Pathog.* **118**, 220–229 (2018).

122. Friedman, J. & Alm, E. J. Inferring Correlation Networks from Genomic Survey Data. *PLoS Comput. Biol.* **8**, e1002687 (2012).

123. Lovell, D., Pawlowsky-Glahn, V., Egozcue, J. J., Marguerat, S. & Bähler, J. Proportionality: A Valid Alternative to Correlation for Relative Data. *PLOS Comput. Biol.* **11**, e1004075 (2015).

124. von Wintzingerode, F., Göbel, U. B. & Stackebrandt, E. Determination of microbial diversity in environmental samples: pitfalls of PCR-based rRNA analysis. *FEMS Microbiol. Rev.* **21**, 213–229 (1997).

125. Han, Z., Sun, J., Lv, A. & Wang, A. Biases from different DNA extraction methods in intestine microbiome research based on 16S rDNA sequencing: a case in the koi carp, Cyprinus carpio var. Koi. *MicrobiologyOpen* **8**, e00626 (2019).

126. Moss, E. L., Maghini, D. G. & Bhatt, A. S. Complete, closed bacterial genomes from microbiomes using nanopore sequencing. *Nat. Biotechnol.* (2020) doi:10.1038/s41587-020-0422-6.

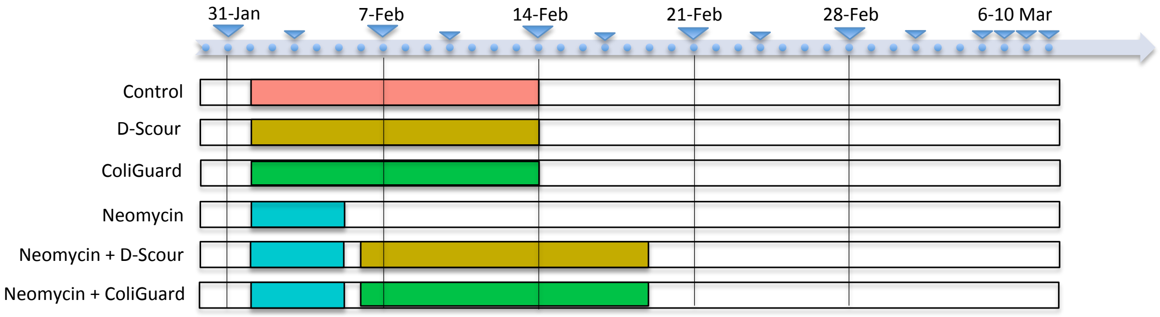

Figure 1. Timeline.

Timeline of the trial indicating the start and the length of the treatment for each cohort. Piglets arrived on the site of the trial on January 30th and were allowed 2 days of acclimatisation before the start of the treatments (pink: placebo paste; yellow: probiotic D-Scour™ formulation; green: probiotic ColiGuard® formulation; blue: antibiotic neomycin intramuscular injection). Vertical lines indicate main days of sampling where all piglets where sampled (*n*=126). Small arrows indicate days of sampling for a subset of the piglets (8 per cohort; *n*=48).

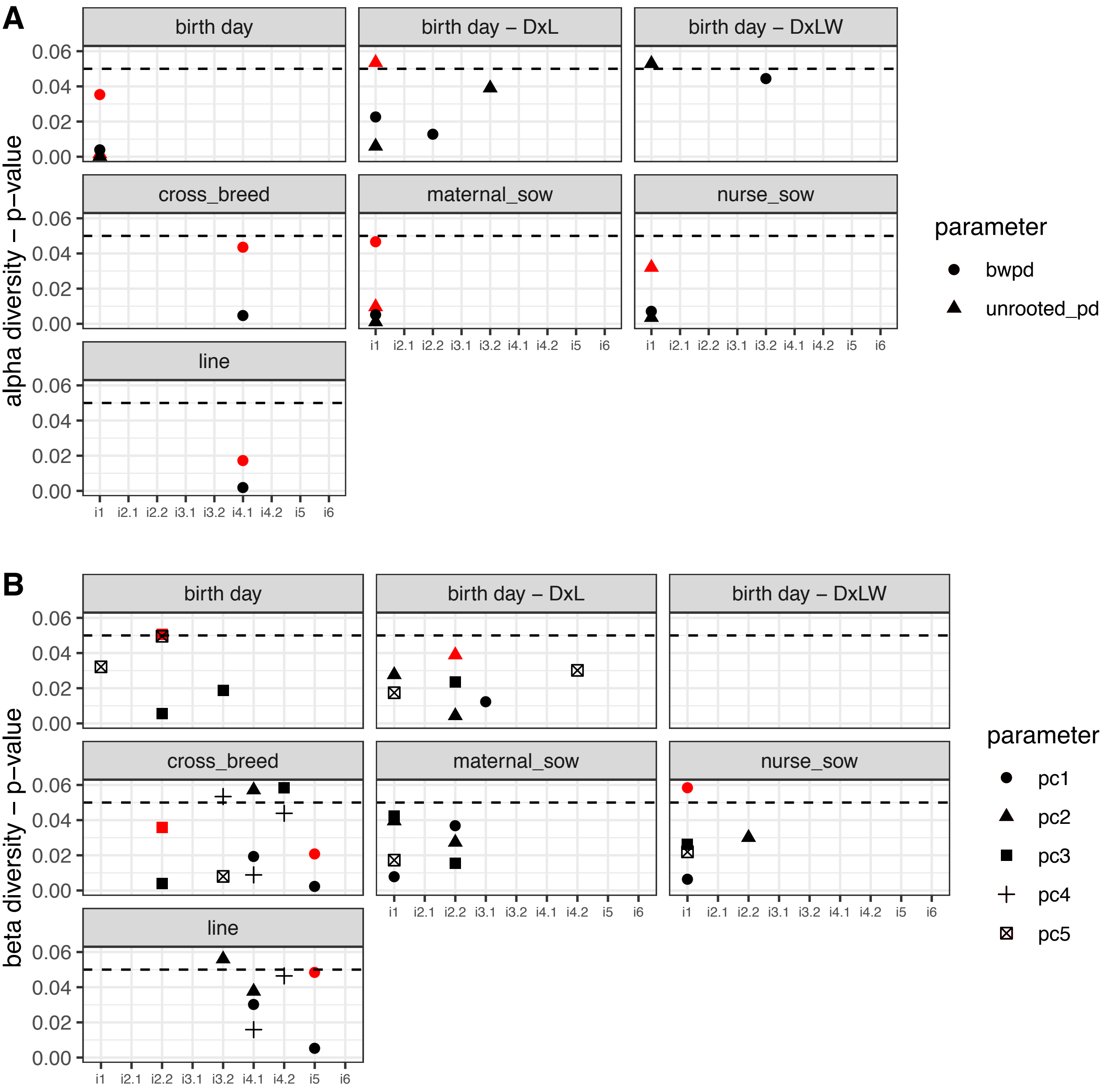

Figure 2. Significance of correlations between phylogenetic diversity and specified factors.

Plots of *p* values derived from Kruskal-Wallis analysis of variance (black) and adjusted *p* values by Benjamini-Hochberg method (red). x-axis displays distinct sampling time points in chronological order. Correlation of specified factors of samples are shown with (**A**) alpha diversity and (**B**) beta diversity. Abbreviations: DxL = "Duroc x Landrace" cross breed; DxLW = "Duroc x Large White" cross breed.

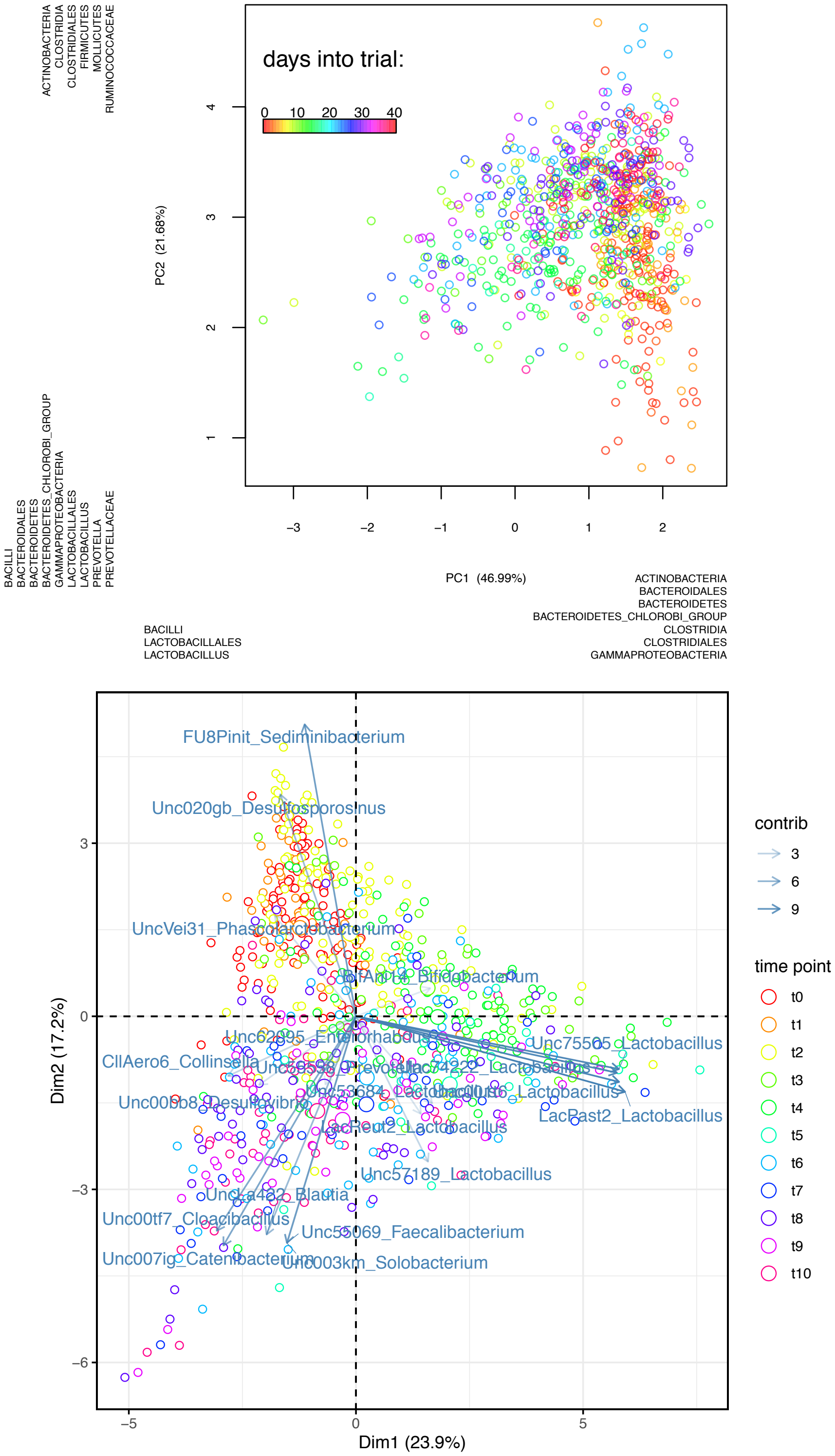

Figure 3. Effect of time on beta diversity.

Principal component analysis (PCA) of samples. PCA from edge component analysis with PhyloSift (top) and PCA from 20 most abundant 16S reads extracted with SortMeRNA (bottom). In the top, distribution of samples on either side of the plot (left *versus* right; top *versus* bottom) reflect the taxa that were found to explain the variation. Samples are colour coded by days into the trial. In the lower plot, arrows indicate which of the 20 taxa read contributed to the variation of samples, where arrows thickness represents a higher (thicker) or lower (thinner) contribution. Samples are colour coded by time point during the trial.

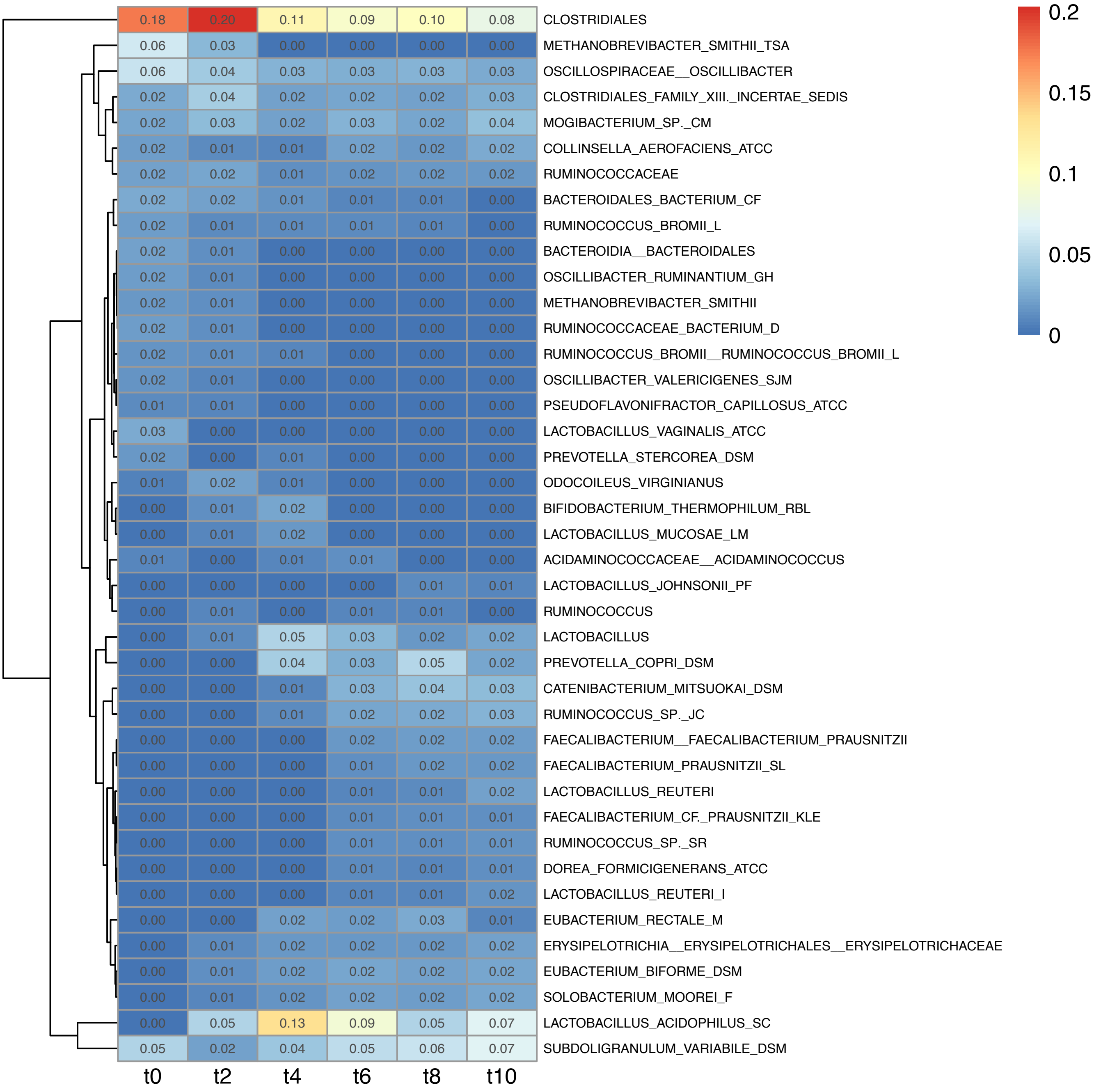

Taxon	t0	t2	t4	t6	t8	t10
CLOSTRIDIALES	0.18	0.20	0.11	0.09	0.10	0.08
METHANOBREVIBACTER_SMITHII_TSA	0.06	0.03	0.00	0.00	0.00	0.00
OSCILLOSPIRACEAE__OSCILLIBACTER	0.06	0.04	0.03	0.03	0.03	0.03
CLOSTRIDIALES_FAMILY_XIII._INCERTAE_SEDIS	0.02	0.04	0.02	0.02	0.02	0.03
MOGIBACTERIUM_SP._CM	0.02	0.03	0.02	0.03	0.02	0.04
COLLINSELLA_AEROFACIENS_ATCC	0.02	0.01	0.01	0.02	0.02	0.02
RUMINOCOCCACEAE	0.02	0.02	0.01	0.02	0.02	0.02
BACTEROIDALES_BACTERIUM_CF	0.02	0.02	0.01	0.01	0.01	0.00
RUMINOCOCCUS_BROMII_L	0.02	0.01	0.01	0.01	0.01	0.00
BACTEROIDIA__BACTEROIDALES	0.02	0.01	0.00	0.00	0.00	0.00
OSCILLIBACTER_RUMINANTIUM_GH	0.02	0.01	0.00	0.00	0.00	0.00
METHANOBREVIBACTER_SMITHII	0.02	0.01	0.00	0.00	0.00	0.00
RUMINOCOCCACEAE_BACTERIUM_D	0.02	0.01	0.00	0.00	0.00	0.00
RUMINOCOCCUS_BROMII__RUMINOCOCCUS_BROMII_L	0.02	0.01	0.01	0.00	0.00	0.00
OSCILLIBACTER_VALERICIGENES_SJM	0.02	0.01	0.00	0.00	0.00	0.00
PSEUDOFLAVONIFRACTOR_CAPILLOSUS_ATCC	0.01	0.01	0.00	0.00	0.00	0.00
LACTOBACILLUS_VAGINALIS_ATCC	0.03	0.00	0.00	0.00	0.00	0.00
PREVOTELLA_STERCOREA_DSM	0.02	0.00	0.01	0.00	0.00	0.00
ODOCOILEUS_VIRGINIANUS	0.01	0.02	0.01	0.00	0.00	0.00
BIFIDOBACTERIUM_THERMOPHILUM_RBL	0.00	0.01	0.02	0.00	0.00	0.00
LACTOBACILLUS_MUCOSAE_LM	0.00	0.01	0.02	0.00	0.00	0.00
ACIDAMINOCOCCACEAE__ACIDAMINOCOCCUS	0.01	0.00	0.01	0.01	0.00	0.00
LACTOBACILLUS_JOHNSONII_PF	0.00	0.00	0.00	0.00	0.01	0.01
RUMINOCOCCUS	0.00	0.01	0.00	0.01	0.01	0.00
LACTOBACILLUS	0.00	0.01	0.05	0.03	0.02	0.02
PREVOTELLA_COPRI_DSM	0.00	0.00	0.04	0.03	0.05	0.02
CATENIBACTERIUM_MITSUOKAI_DSM	0.00	0.00	0.01	0.03	0.04	0.03
RUMINOCOCCUS_SP._JC	0.00	0.00	0.01	0.02	0.02	0.03
FAECALIBACTERIUM__FAECALIBACTERIUM_PRAUSNITZII	0.00	0.00	0.00	0.02	0.02	0.02
FAECALIBACTERIUM_PRAUSNITZII_SL	0.00	0.00	0.00	0.01	0.02	0.02
LACTOBACILLUS_REUTERI	0.00	0.00	0.00	0.01	0.01	0.02
FAECALIBACTERIUM_CF._PRAUSNITZII_KLE	0.00	0.00	0.00	0.01	0.01	0.01
RUMINOCOCCUS_SP._SR	0.00	0.00	0.00	0.01	0.01	0.01
DOREA_FORMICIGENERANS_ATCC	0.00	0.00	0.00	0.01	0.01	0.01
LACTOBACILLUS_REUTERI_I	0.00	0.00	0.00	0.01	0.01	0.02
EUBACTERIUM_RECTALE_M	0.00	0.00	0.02	0.02	0.03	0.01
ERYSIPELOTRICHIA__ERYSIPELOTRICHALES__ERYSIPELOTRICHACEAE	0.00	0.01	0.02	0.02	0.02	0.02
EUBACTERIUM_BIFORME_DSM	0.00	0.01	0.02	0.02	0.02	0.02
SOLOBACTERIUM_MOOREI_F	0.00	0.01	0.02	0.02	0.02	0.02
LACTOBACILLUS_ACIDOPHILUS_SC	0.00	0.05	0.13	0.09	0.05	0.07
SUBDOLIGRANULUM_VARIABILE_DSM	0.05	0.02	0.04	0.05	0.06	0.07

Figure 4. Abundance heatmap.

Most abundant taxa within each time point are obtained from analysis with guppy fat. Abundance is derived from the branch width. The distance between each time point is of one week.

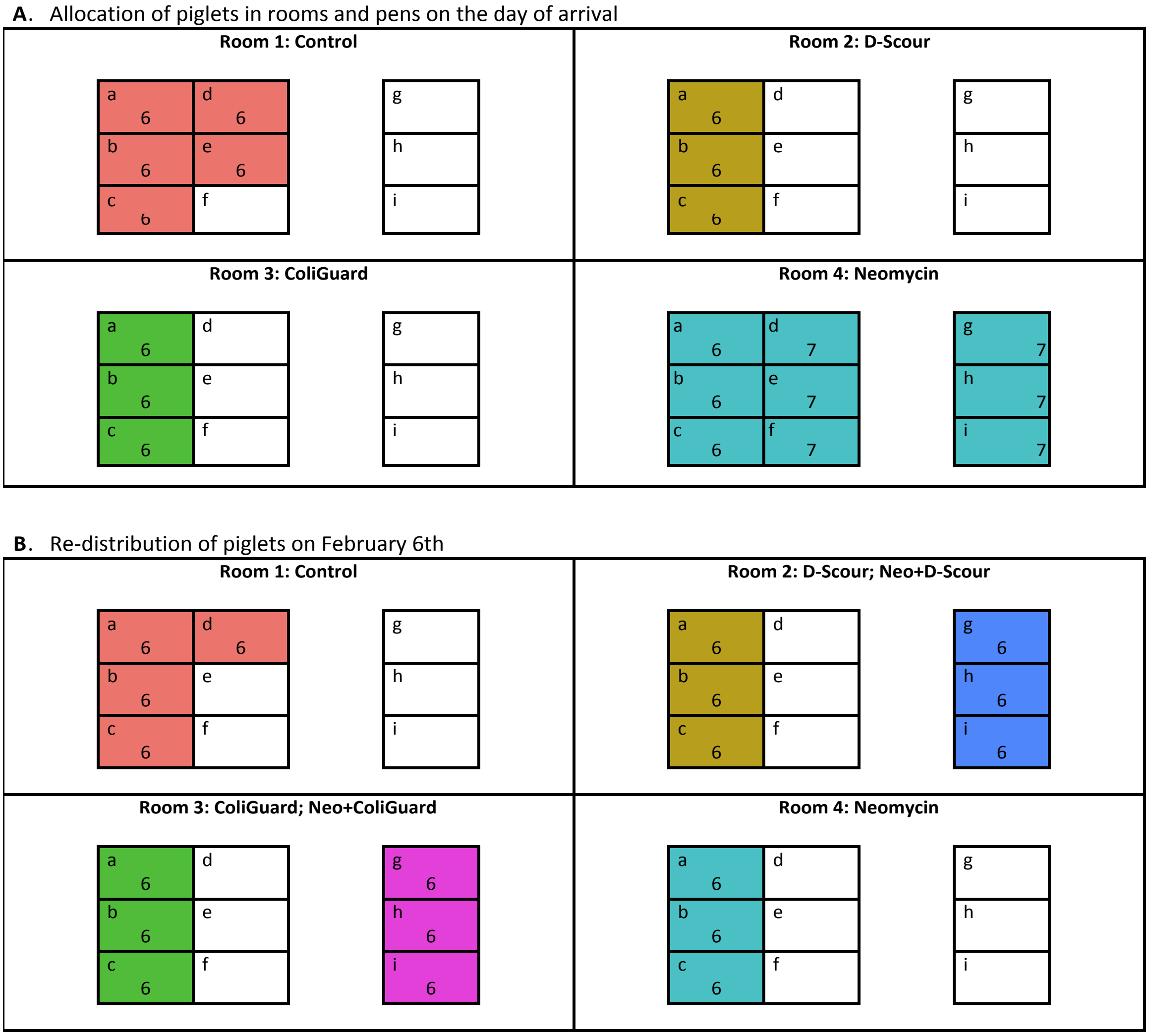

Supplementary Figure 1. Piglets placements across rooms and pens.

Disposition of piglets in rooms (1-4) and pens (a-i) at the start of the trial (**A**) and re-arrangement of a subset of neomycin treated piglets from room 4 to room 2 and room 3, for D-Scour™ and ColiGuard® treatment, respectively (**B**).

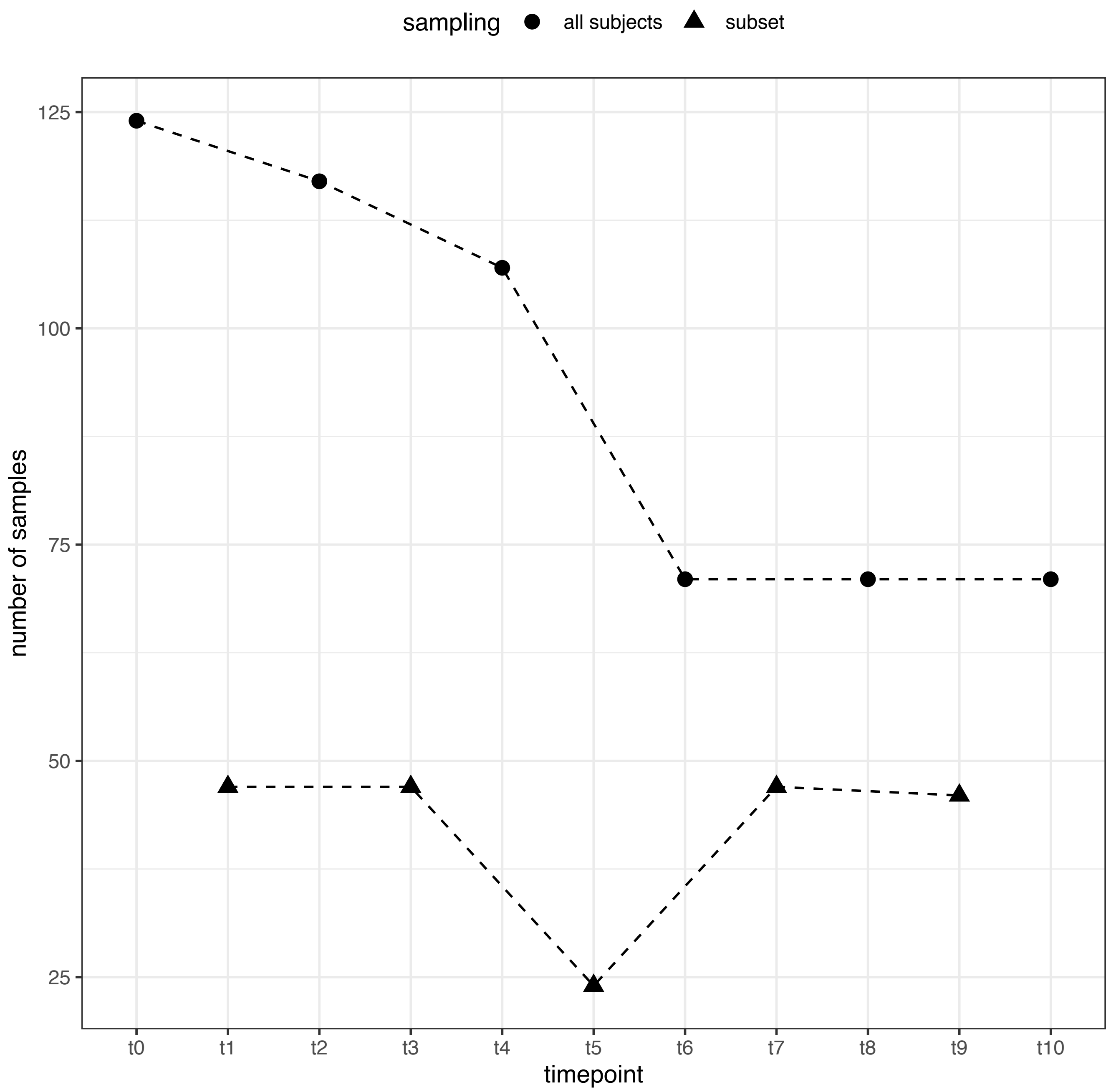

Supplementary Figure 2. Sample collection.

Samples were collected from all piglets once weekly, and from a subset group (8 per cohort; *n*=48) twice weekly. Overall, piglets were sampled between 1 and 10 times.

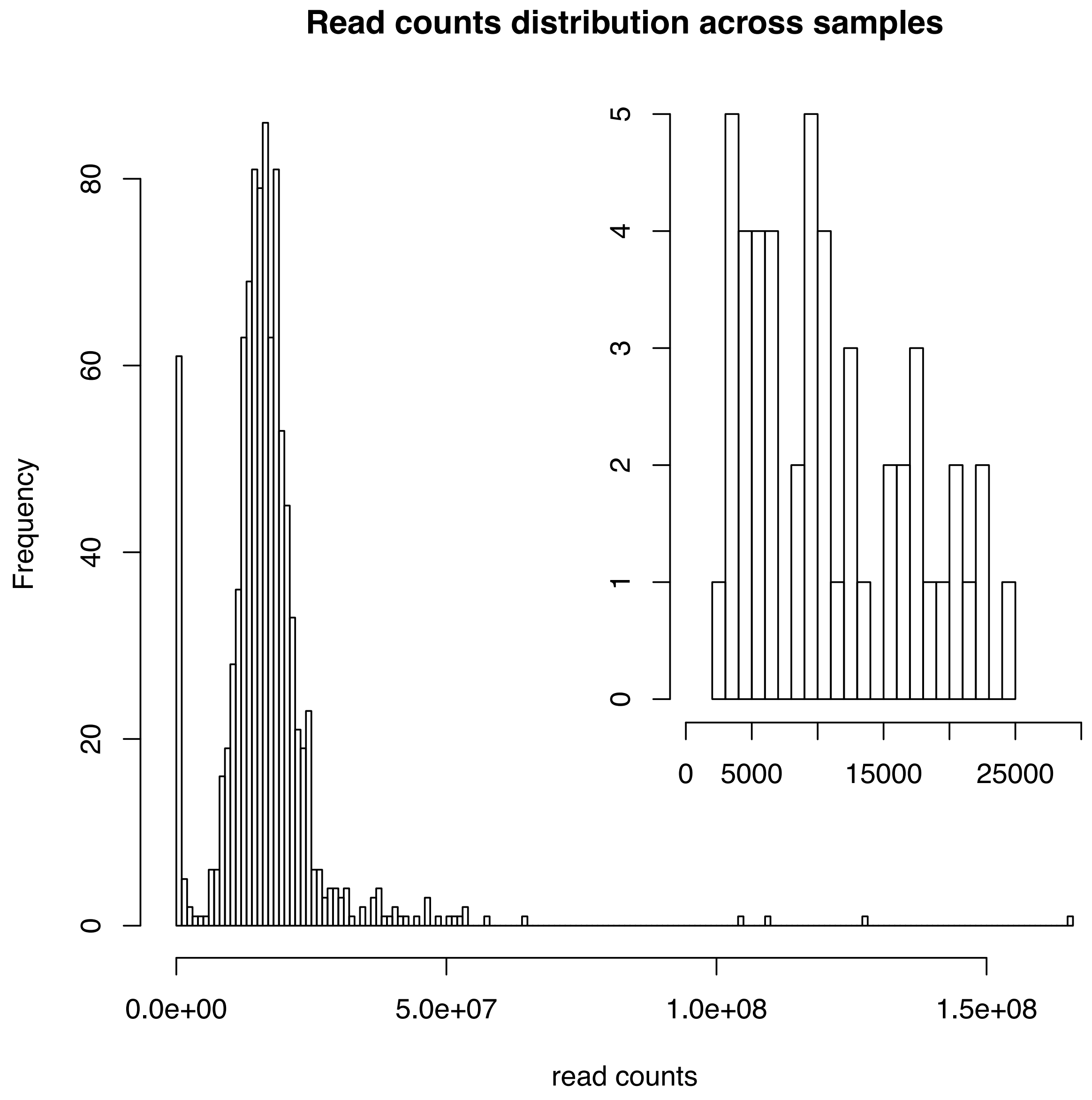

Supplementary Figure 3. Read counts distribution.

Read counts distribution of all samples (main histogram) and low read counts samples (top right histogram).

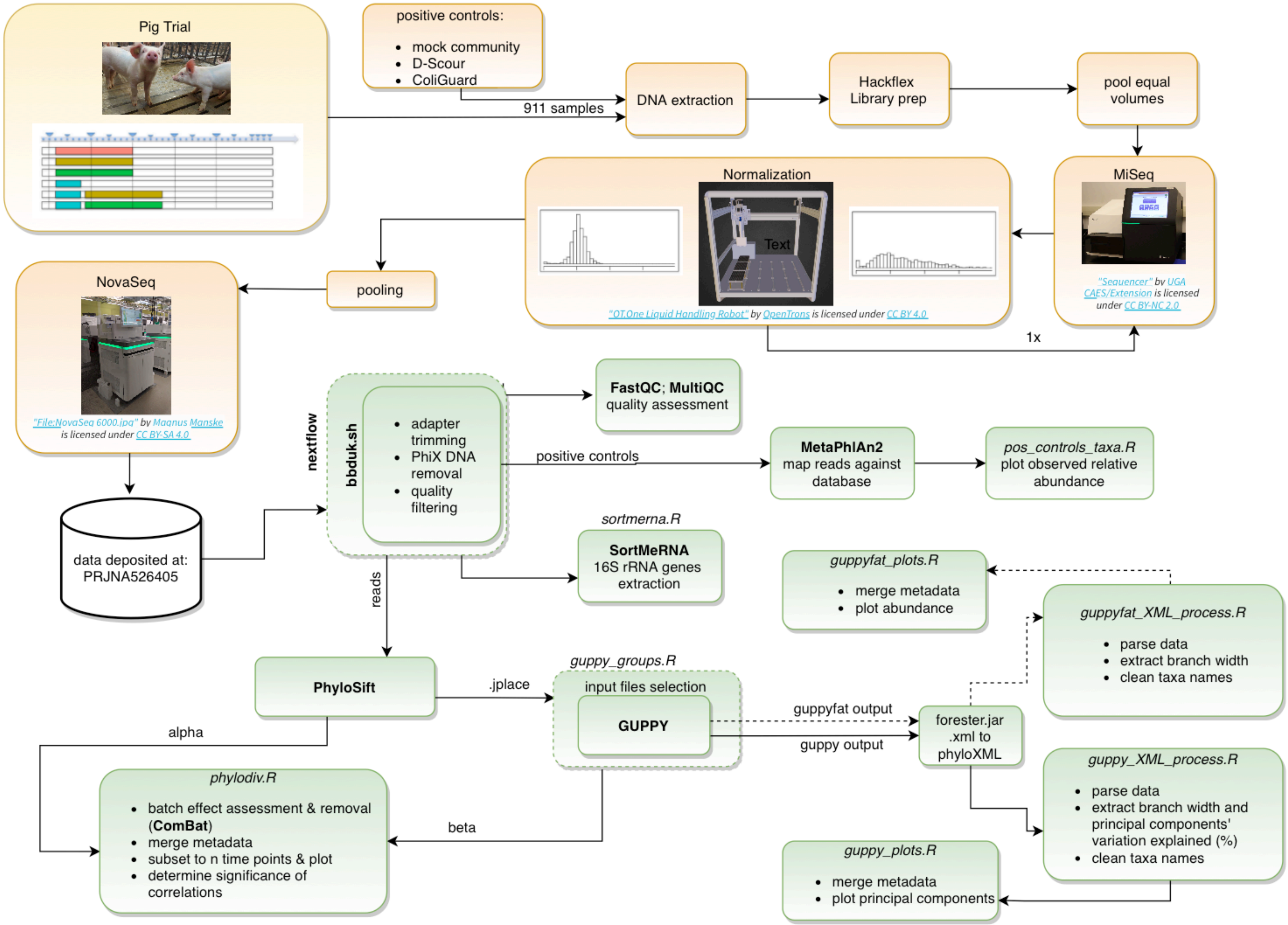

Supplementary Figure 4. Summarised workflow.

Schematic workflow from sample collection to sequencing (orange) and data analysis (green). R scripts (italic) are available in our Github repository.

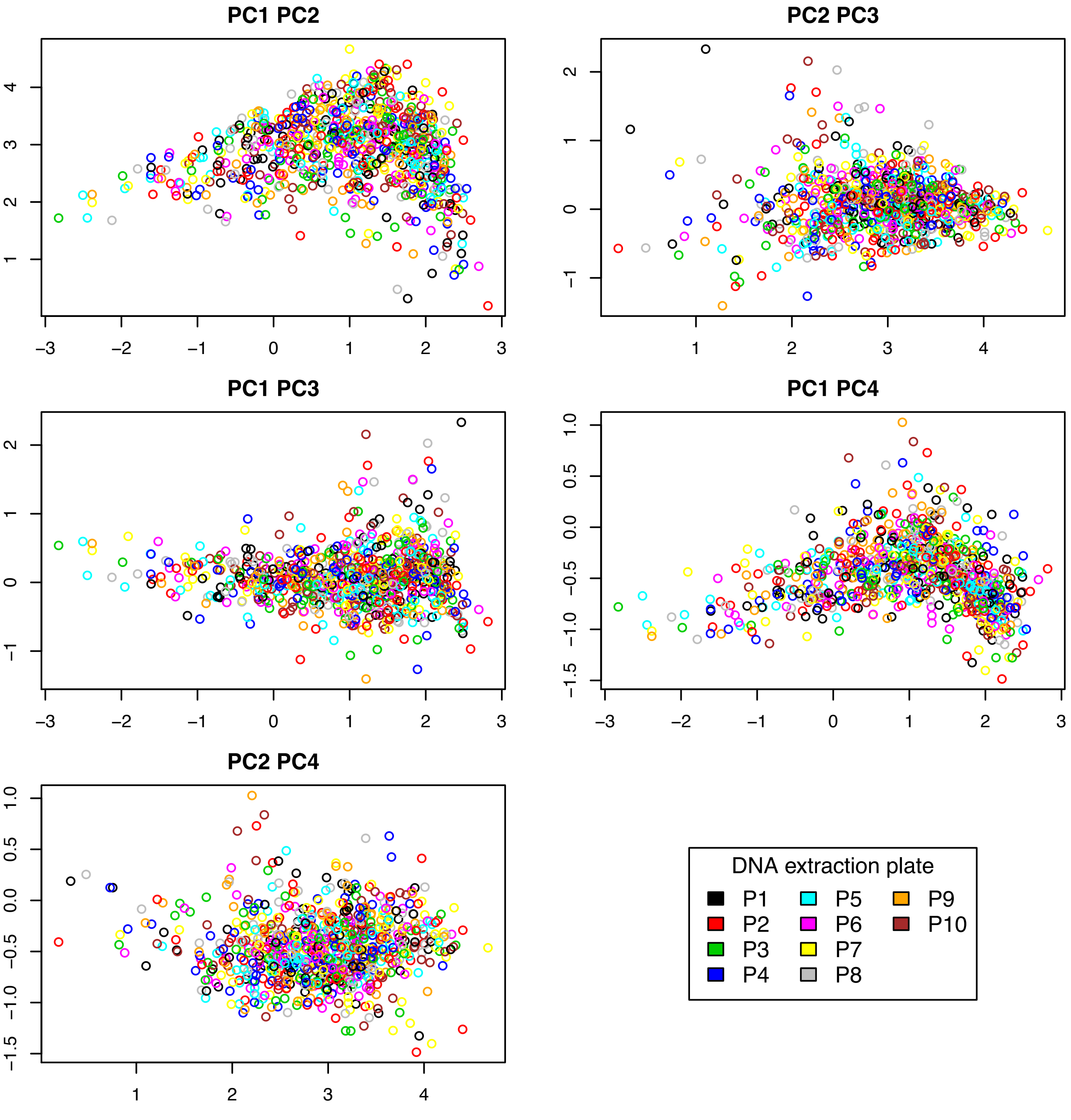

Supplementary Figure 5. Principal component analysis of batch effect before batch effect removal.

Principal components 1 to 4 from beta diversity analysis of all samples before batch effect removal. Samples are coloured by DNA extraction plate. No clustering of samples by DNA extraction plate is visible.

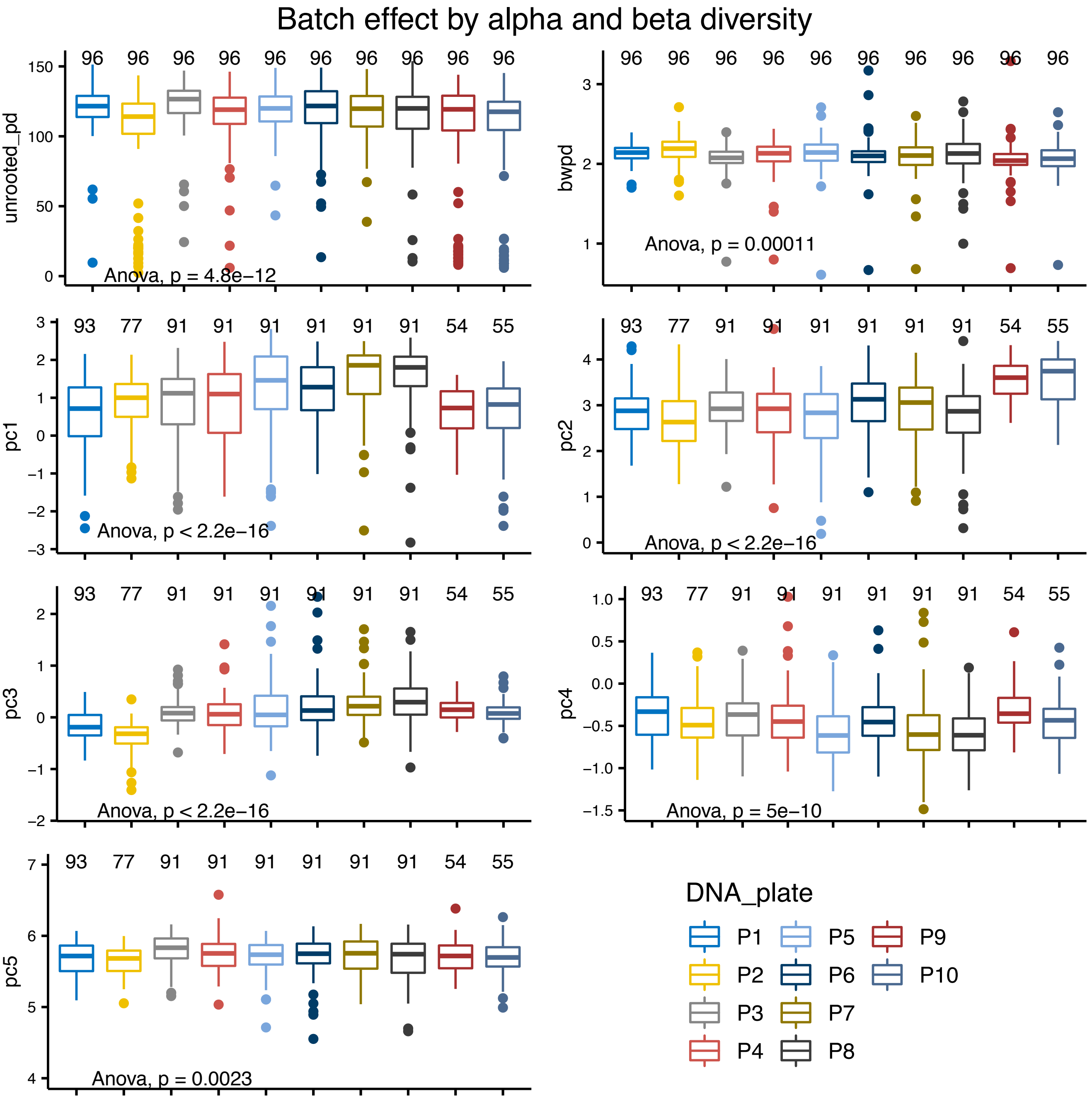

Supplementary Figure 6. Batch effect on alpha and beta diversity before batch effect removal.

Batch effect by alpha (top two plots) and beta diversity (bottom five plots) before batch effect removal. Samples are grouped by DNA extraction plate. The *p* values are derived from multiple comparison analysis with ANOVA, indicating equality of the means. *Post hoc* corrected *p* values for pairwise comparisons are provided in Supplementary File 5.

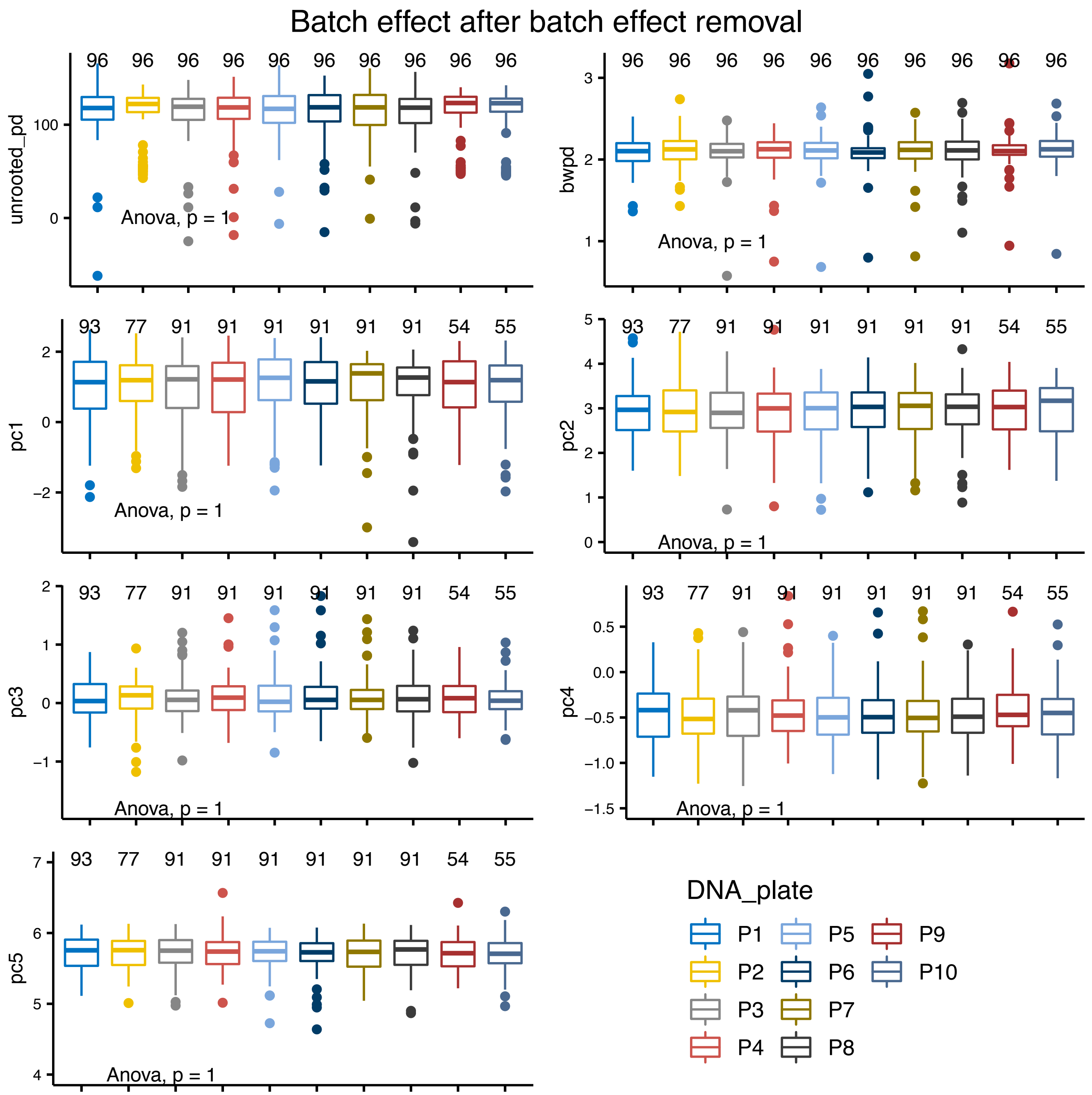

Supplementary Figure 7. Batch effect on alpha and beta diversity after batch effect removal.

Batch effect by alpha (top two plots) and beta diversity (bottom five plots) after batch effect removal. Samples are grouped by DNA extraction plate. The *p* values are derived from multiple comparison analysis with ANOVA, indicating equality of the means. *Post hoc* corrected *p* values for pairwise comparisons are provided in Supplementary File 5.

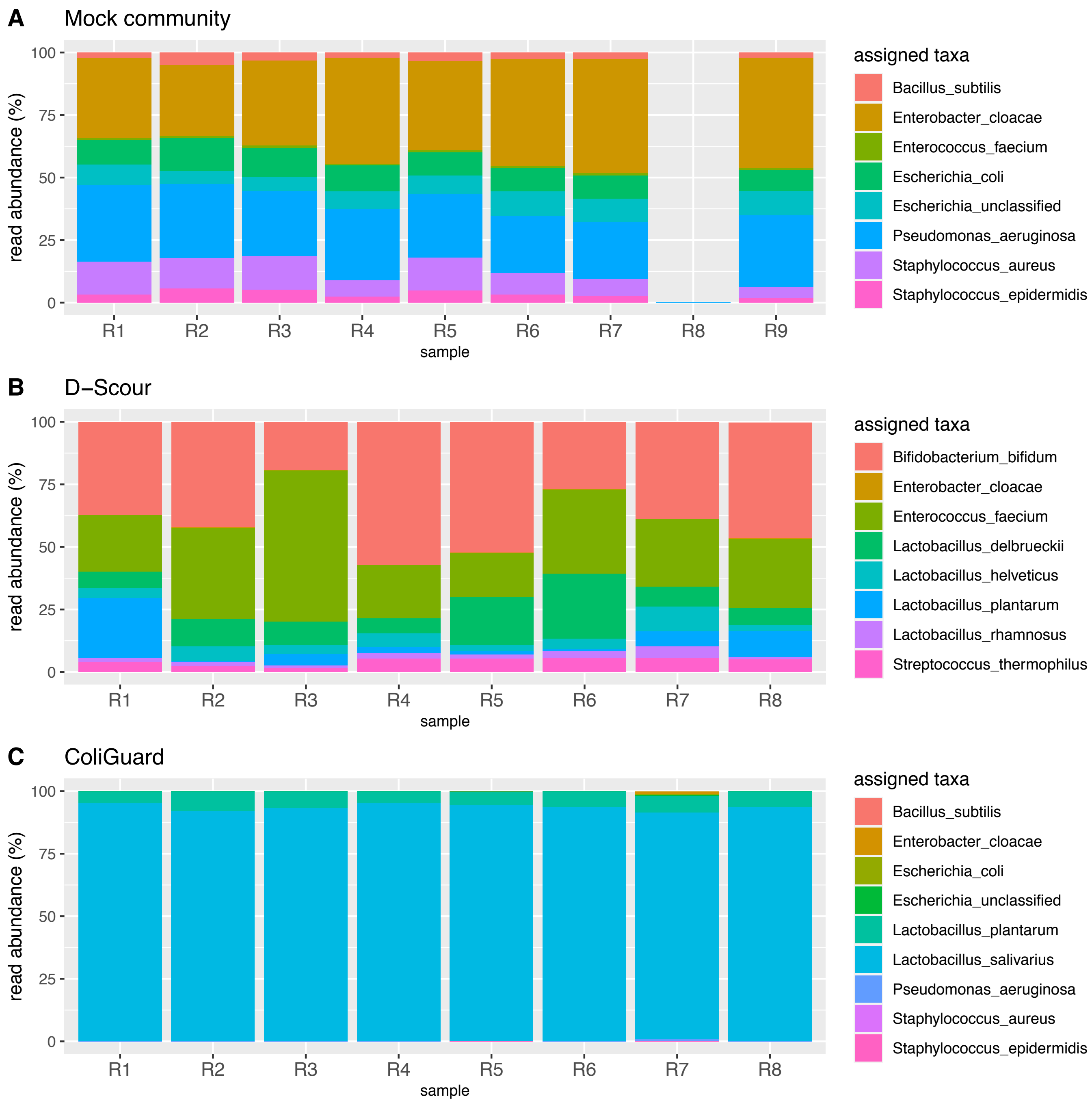

Supplementary Figure 8. Taxonomic assignment of reads from positive control samples.

Taxonomic profile of the positive controls used in this study obtained by mapping the reads against a ~1M bacterial genomes database with MetaPhlAn2. Each stacked barplot represents a technical replicate. Taxa of which reads are present in >0.1% are displayed. **A**. In-house made mock community; **B**. commercially available livestock probiotic D-Scour™ ; **C**. ColiGuard®.

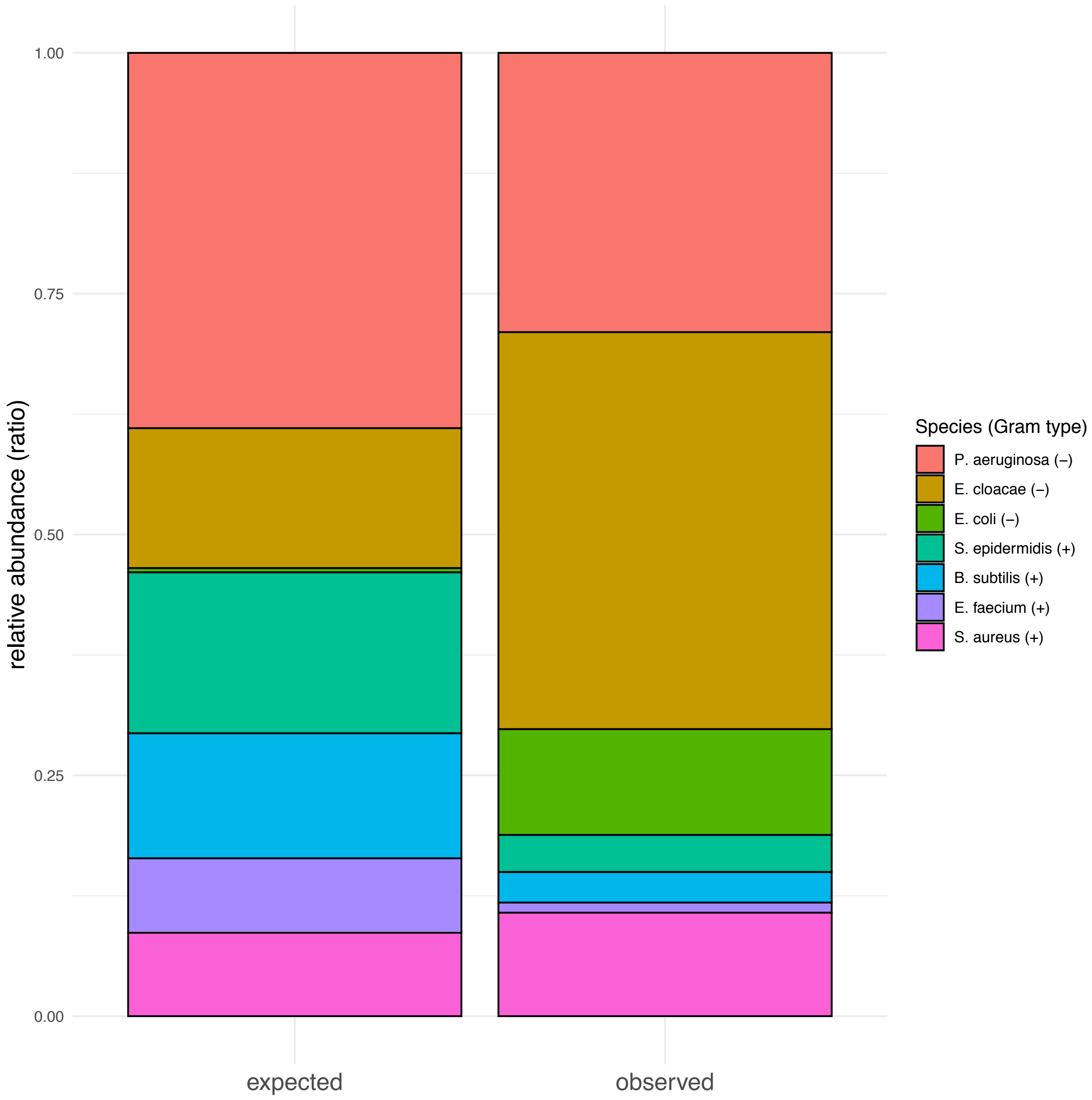

Supplementary Figure 9. Expected and observed relative abundance of mock community members.

Expected relative abundance is derived by multiplying CFU count by genome size. Observed relative abundance is derived by multiplying reads mapping with MetaPhlAn2. A higher observed/expected ratio is observed in two of the three Gram negative species and a lower observed/expected ratio is observed in three of the four Gram negative species.

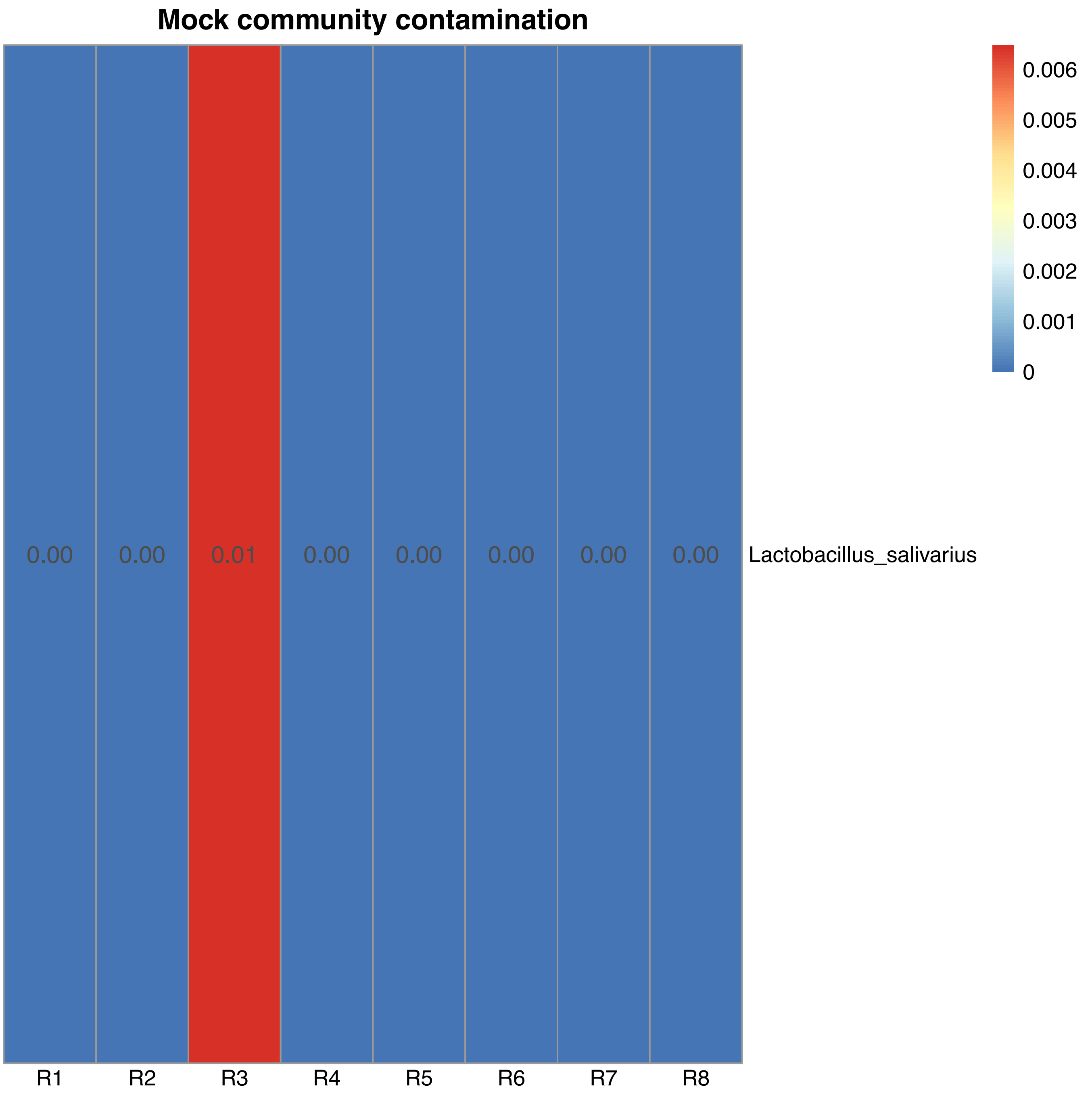

Supplementary Figure 10. Contamination rate in mock community samples.

Heatmap of contaminating species present in the positive control mock community technical replicates. *Lactobacillus salivarius* is found in one replicate at 0.01% of the total reads.

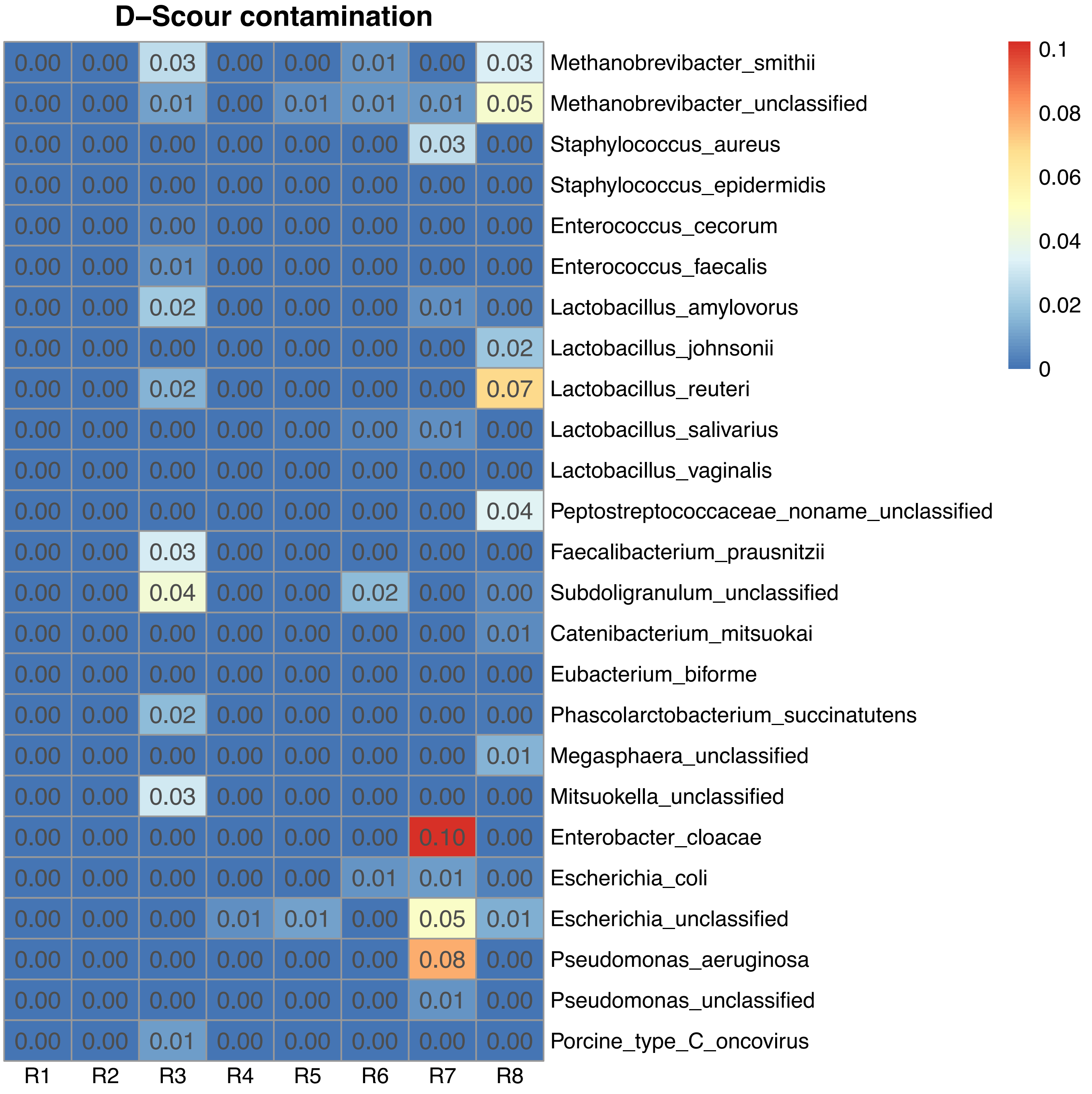

Supplementary Figure 11. Contamination rate in D-Scour™ samples.

Heatmap of contaminating species present in the positive control D-Scour™ technical replicates. The D-Scour™ technical replicates contained 25 contaminants, of which 18 and 7 were identified at the species and at genus level, respectively. Contaminants are majorly present in three technical replicates (R3, R7, R8) and the most frequent contaminant (*Metahobrevibacter*) was present in 5 of the 8 replicates.

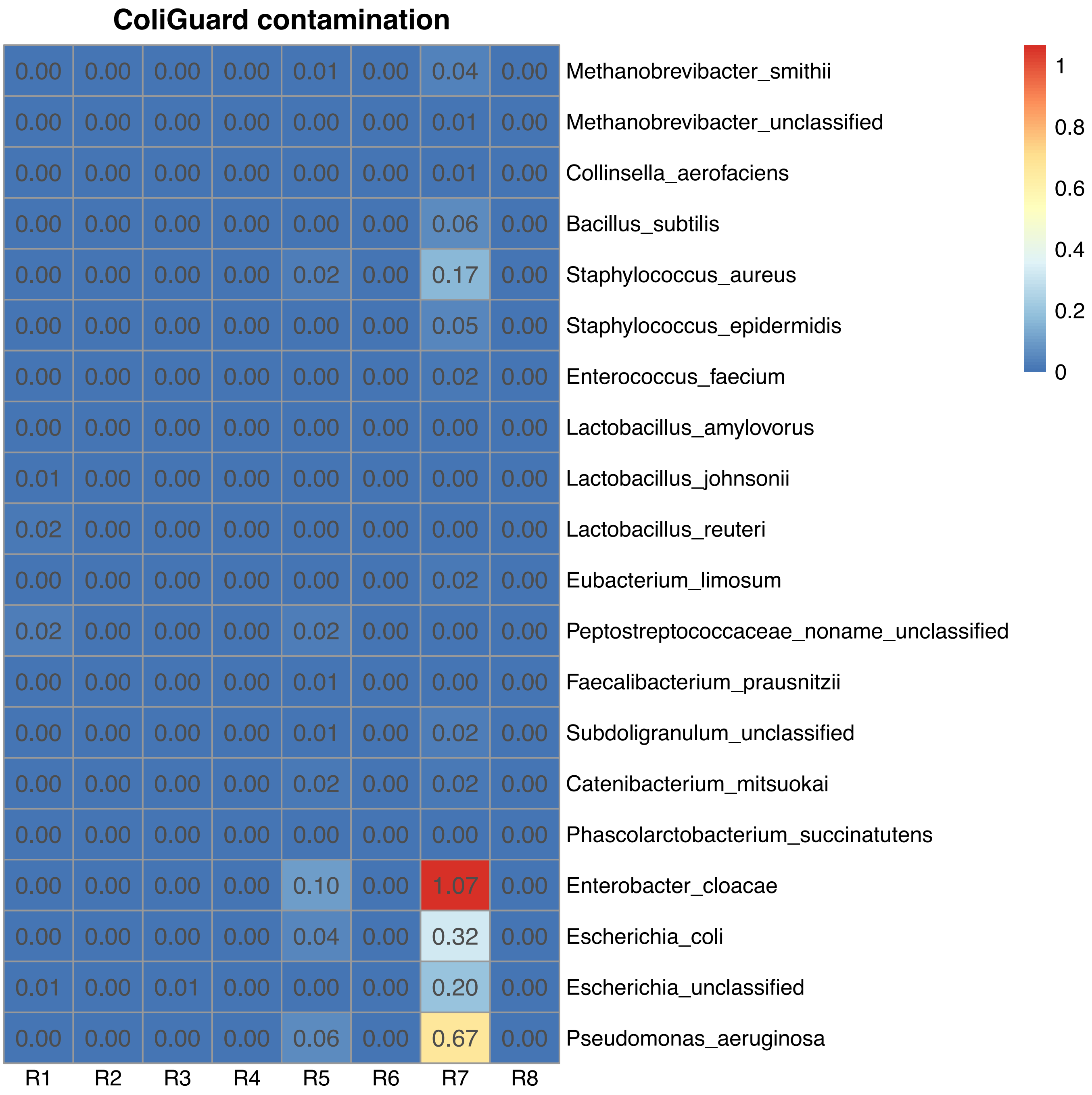

Supplementary Figure 12. Contamination rate in ColiGuard® samples.

Heatmap of contaminating species present in the positive control ColiGuard® technical replicates. ColiGuard® contained 20 contaminants, of which 16 and 4 were identified at the species and at genus level, respectively. Contaminants were present majorly in two technical replicates (R5, R7).

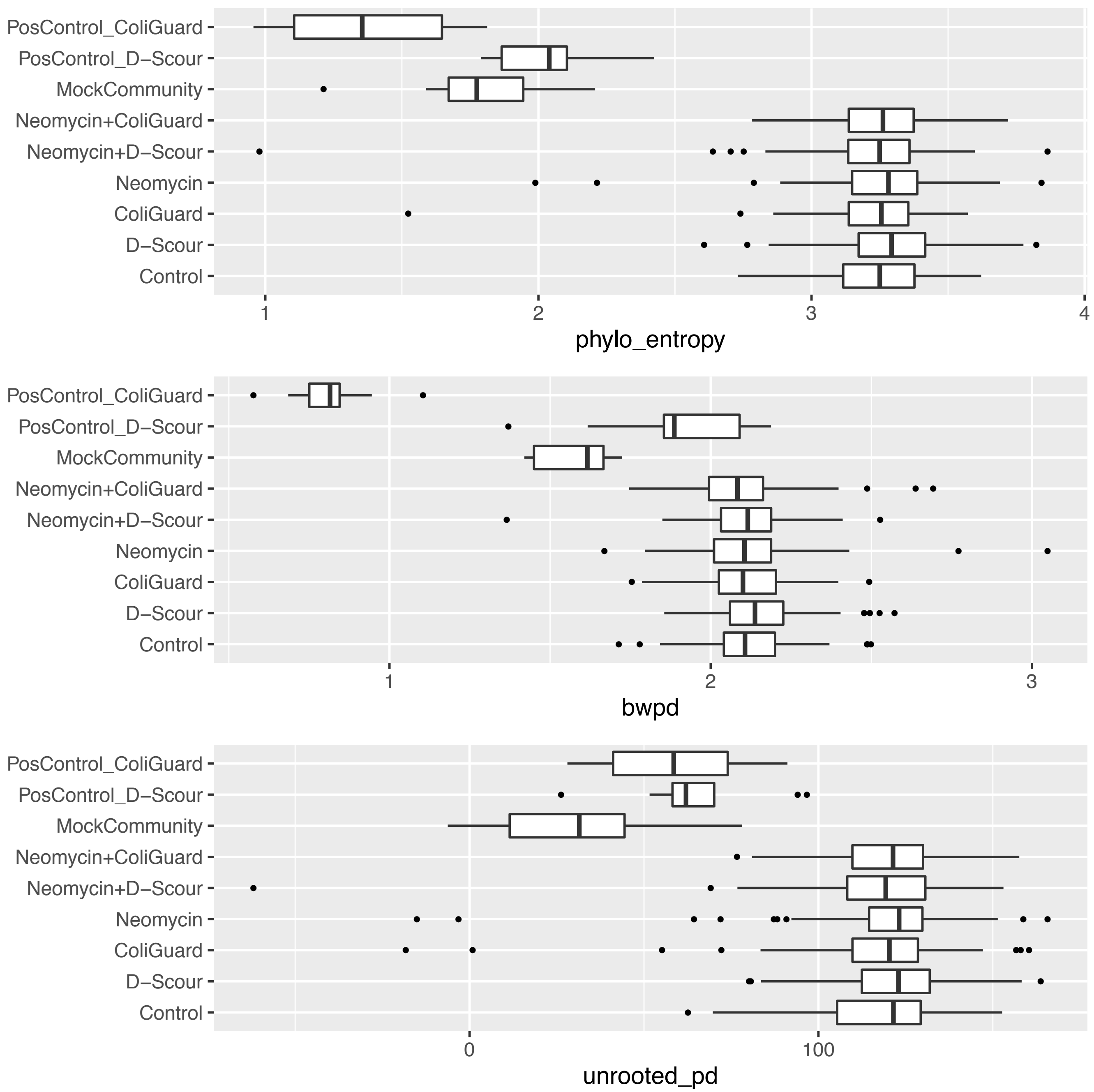

Supplementary Figure 13. Alpha phylogenetic diversity of cohorts.

Alpha phylogenetic diversity per cohort from samples across all time points. Phylogenetic entropy (top); Balance-weighted phylogenetic diversity (BWPD) (mean±SD: Positive control Mock community: 1.58±0.12; Positive control D-Scour™: 1.89±0.26; Positive control ColiGuard®: 0.82±0.15; Control: 2.12±0.14; D-Scour™: 2.14±0.14; ColiGuard®: 2.11±0.13; neomycin: 2.12±0.17; neomycin+D-Scour™: 2.12±0.14; neomycin+ColiGuard®: 2.10±0.15; sows: 2.13±0.14; all piglet cohorts: 2.12±0.14); (middle); Unrooted phylogenetic diversity (Positive control Mock community: 31.53±29.50; Positive control D-Scour™: 64.84±21.30; Positive control ColiGuard®: 58.52±21.70; Control: 117±16.8; D-Scour™: 121±14.7; ColiGuard®: 118±22.3; neomycin: 119±22.6; neomycin+D-Scour™: 116±23.4; neomycin+ColiGuard®: 121±15.8; sows: 125.39±7.56; all piglet cohorts: 118.54±19.70) (bottom).

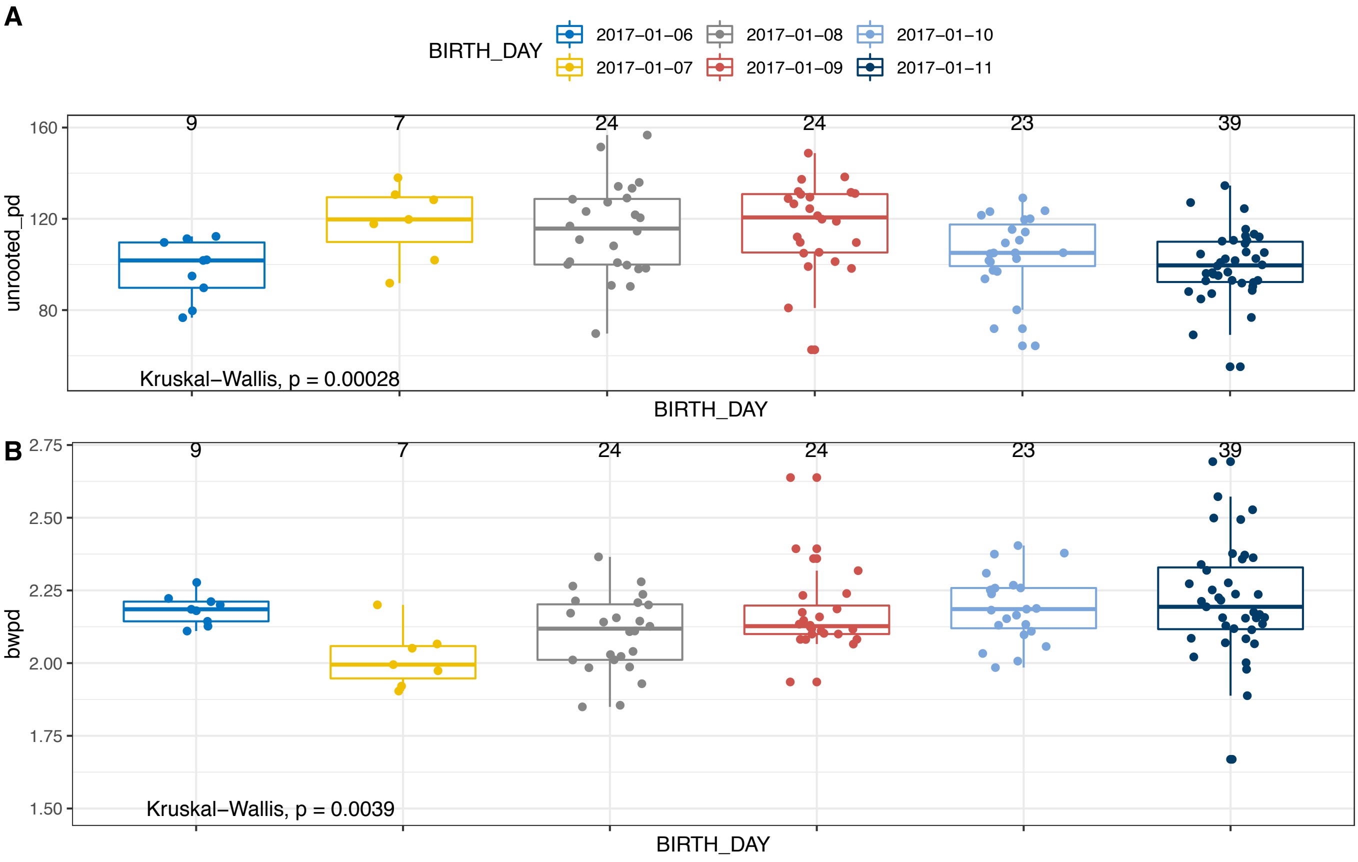

Supplementary Figure 14. Alpha phylogenetic diversity by age group.

Alpha diversity of samples from the start of the trial grouped by the date of birth of the piglet. Unrooted phylogenetic diversity (top) and balance weighted phylogenetic diversity (bottom). *P* values are derived from Kruskal-Wallis analysis of variance. Pairwise comparisons and *post hoc* corrected *p* values are provided in Supplementary File 5.

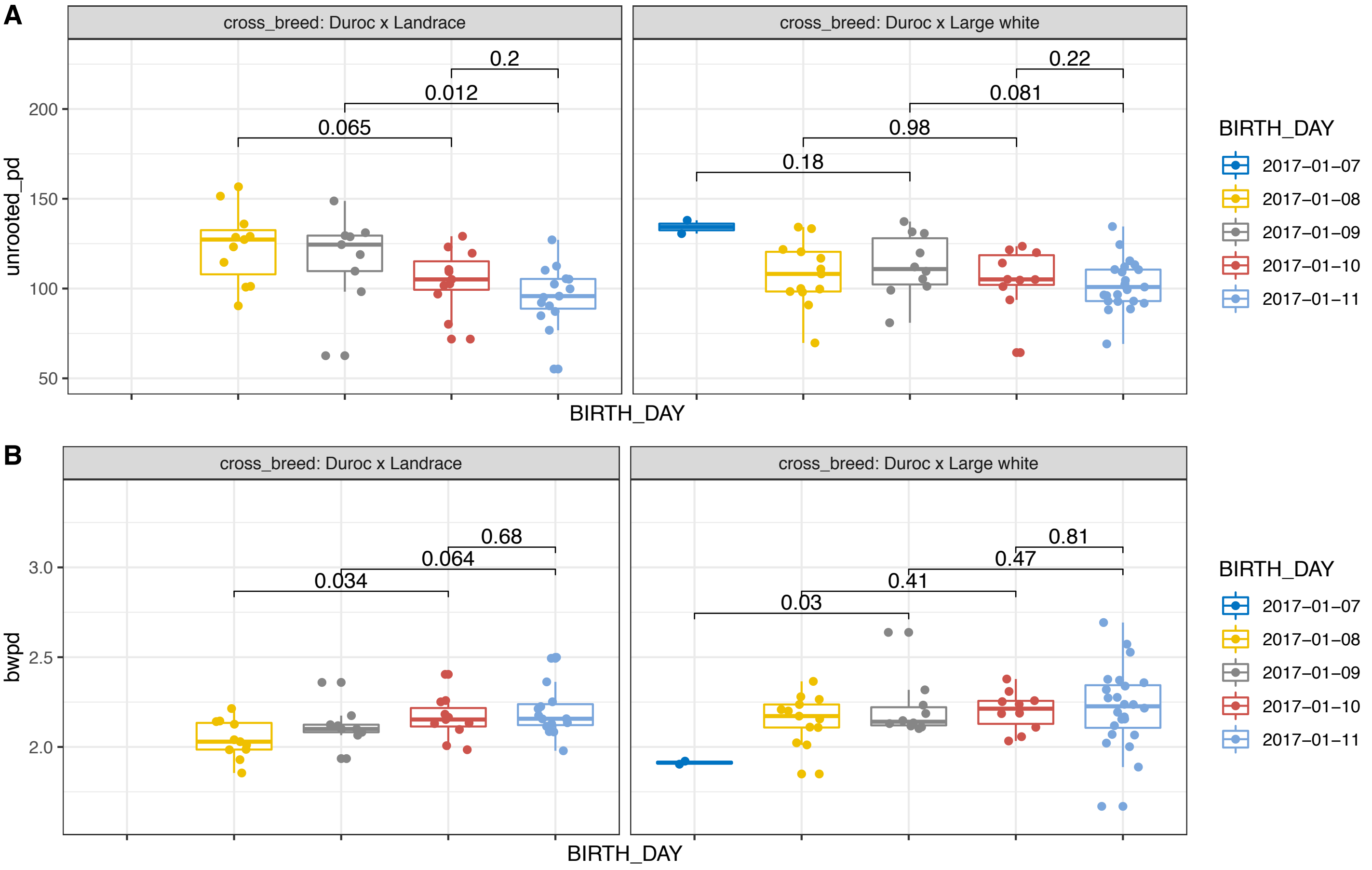

Supplementary Figure 15. Alpha phylogenetic diversity by age breed.

Alpha diversity of samples from the start of the trial grouped by breed and by date of birth. Unrooted phylogenetic diversity (top) and balance weighted phylogenetic diversity (bottom). *P* values are derived from Kruskal-Wallis analysis of variance. Pairwise comparisons and *post hoc* corrected *p* values are provided in Supplementary File 5.

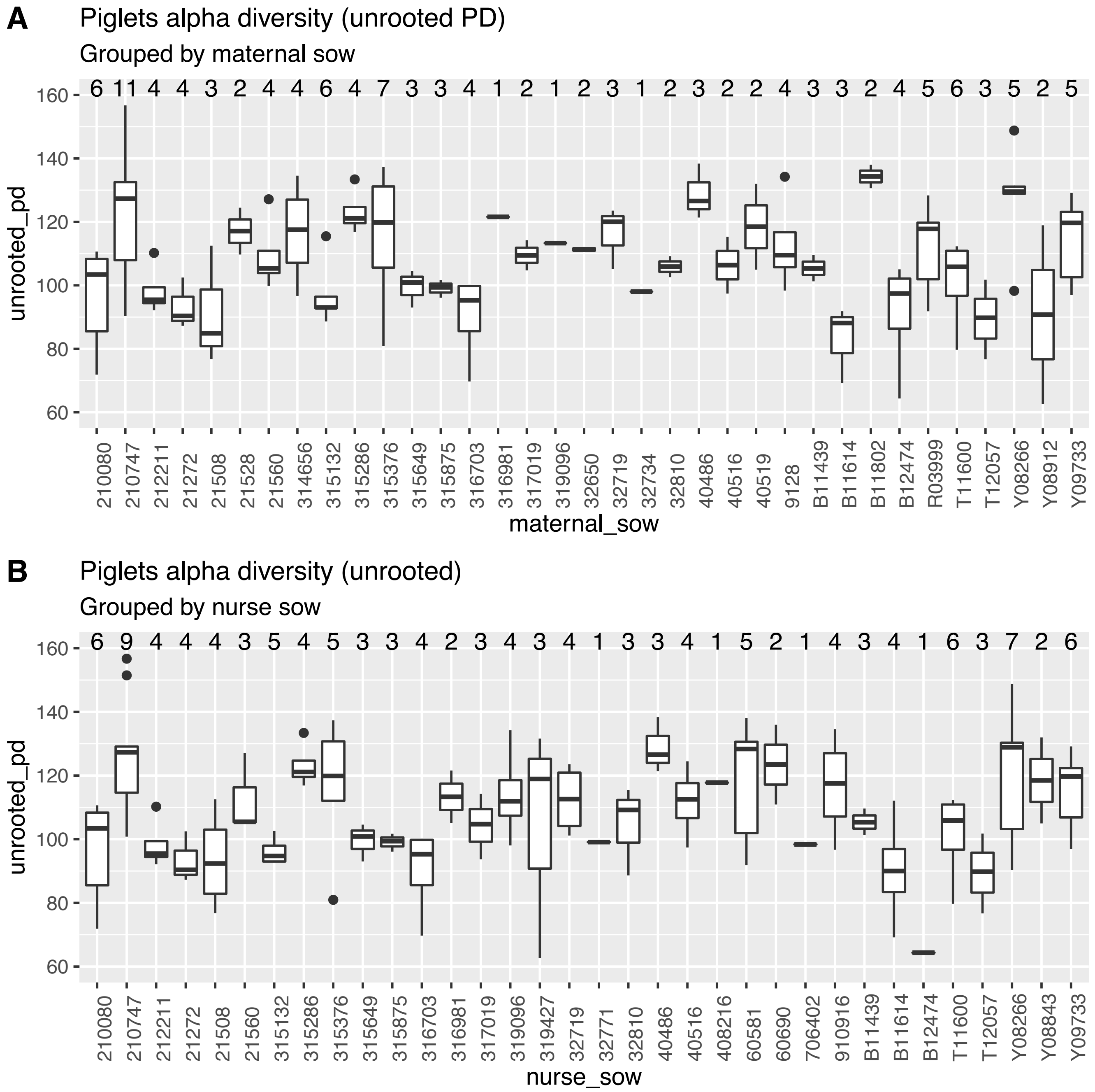

Supplementary Figure 16. Unrooted alpha phylogenetic diversity by maternal and nurse sow.

Alpha phylogenetic diversity (unrooted) of samples from piglets at the start of the trial (one time point -sample per piglet). The grouping is based on the maternal sow (**A**) or nurse sow (**B**). *P* values from multiple groups comparison by maternal sow: Kruskal-Wallis *p* values: 0.0011; BWPD: 0.0049. *P* values from multiple groups comparison by nurse sow: Kruskal-Wallis *p* values: 0.0036; BWPD: 0.0071. *Post-hoc* pairwise comparisons and adjusted *p* values are provided in Supplementary File 5.

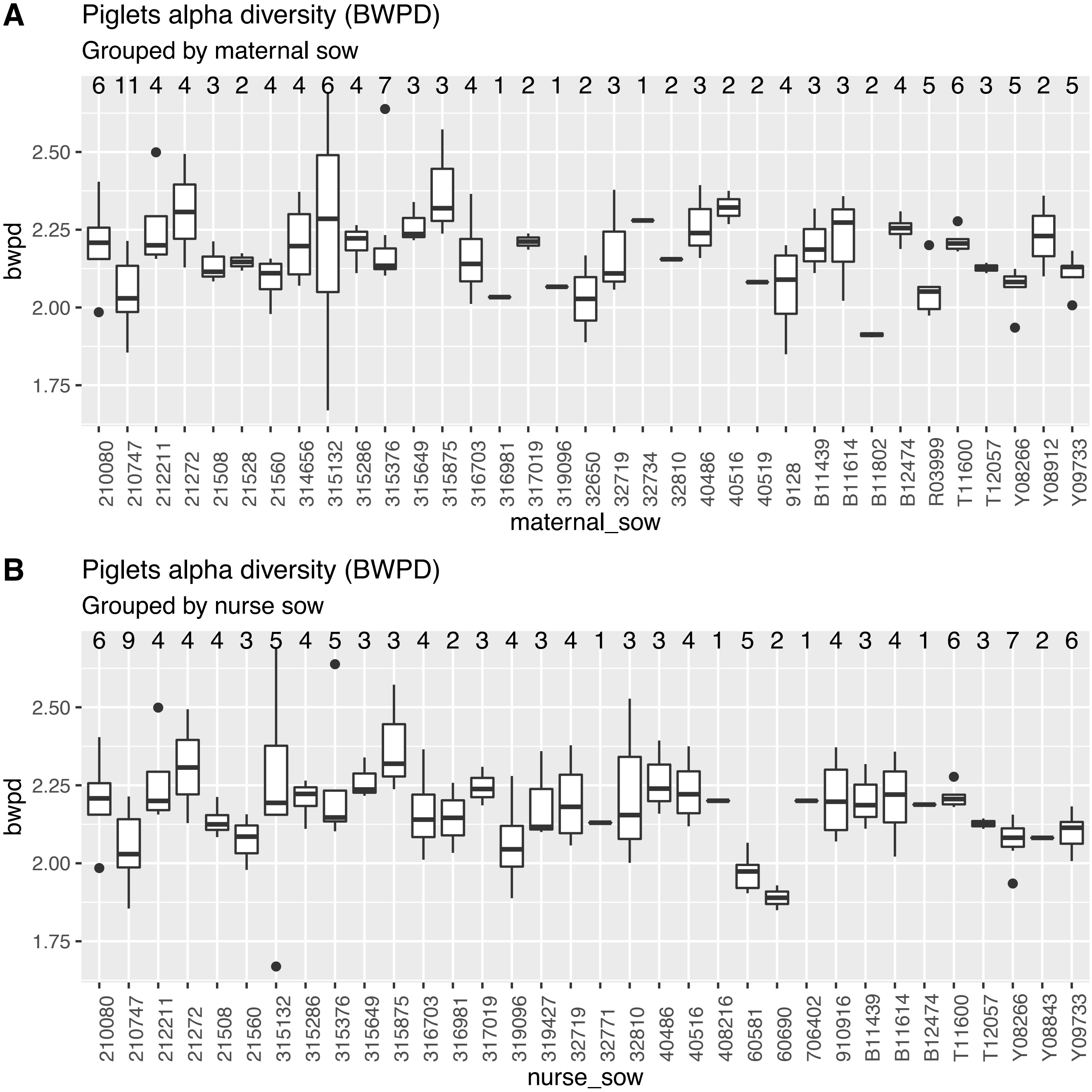

Supplementary Figure 17. Balance weighted alpha phylogenetic diversity by maternal and nurse sow.

Alpha phylogenetic diversity (unrooted) of samples from piglets at the start of the trial (one time point -sample per piglet). The grouping is based on the maternal sow (**A**) or nurse sow (**B**). *P* values from multiple groups comparison by maternal sow: Kruskal-Wallis *p*=0.0049. *P* values from multiple groups comparison by nurse sow: Kruskal-Wallis *p*=0.0071. *Post-hoc* pairwise comparisons and adjusted *p* values are provided in Supplementary File 5.

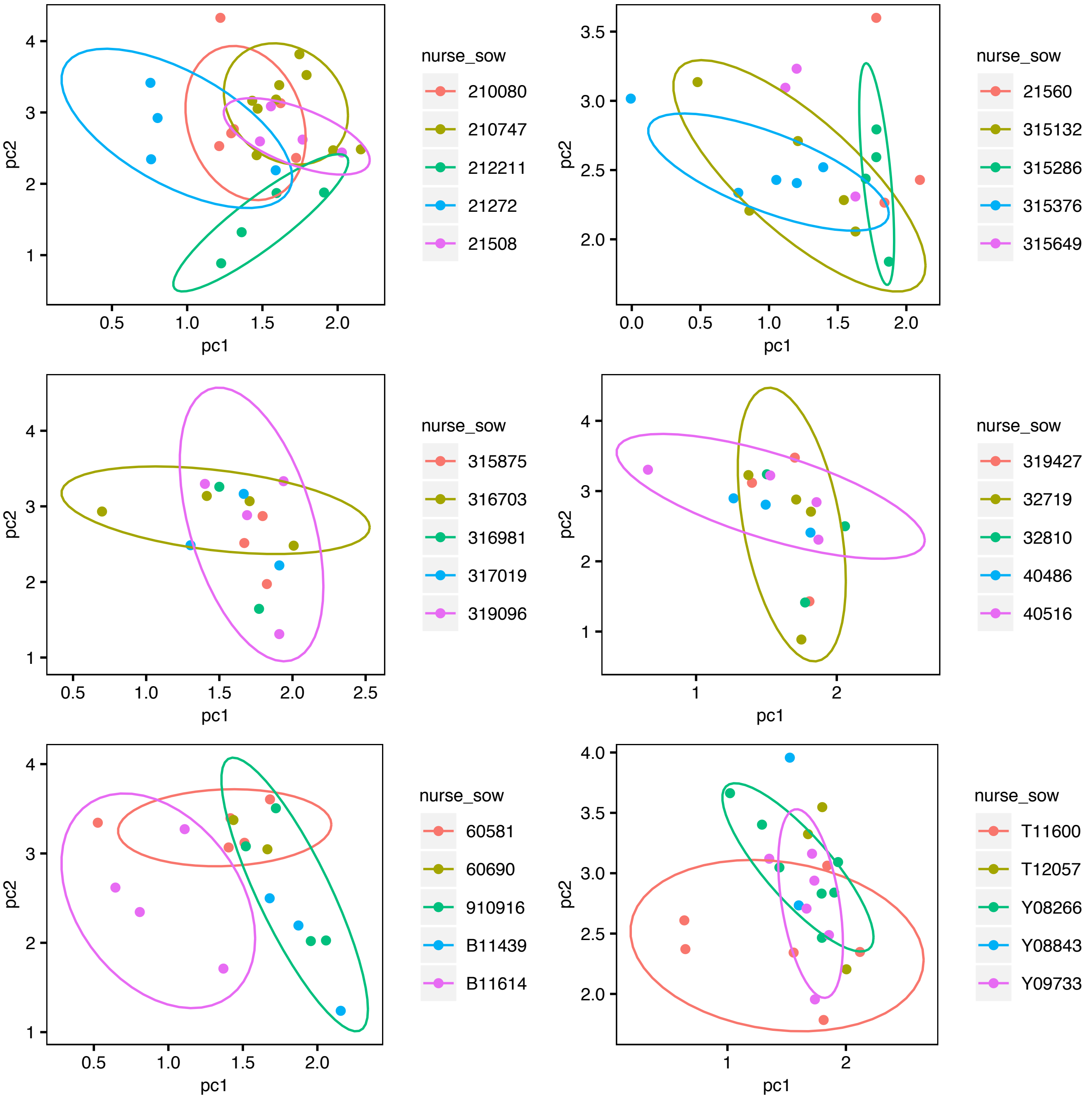

Supplementary Figure 18. Principal component analysis of piglet samples by nurse sow.

First two principal components of beta phylogenetic diversity. Principal component 1 (PC1) and principal component 2 (PC2) explaining 46.99% and 21.68% of the variation, respectively.
Samples from piglets at the start of the trial (one time point -sample per piglet) coloured by nurse sow (n=30). Ellipse is drawn at 0.80 confidence level.

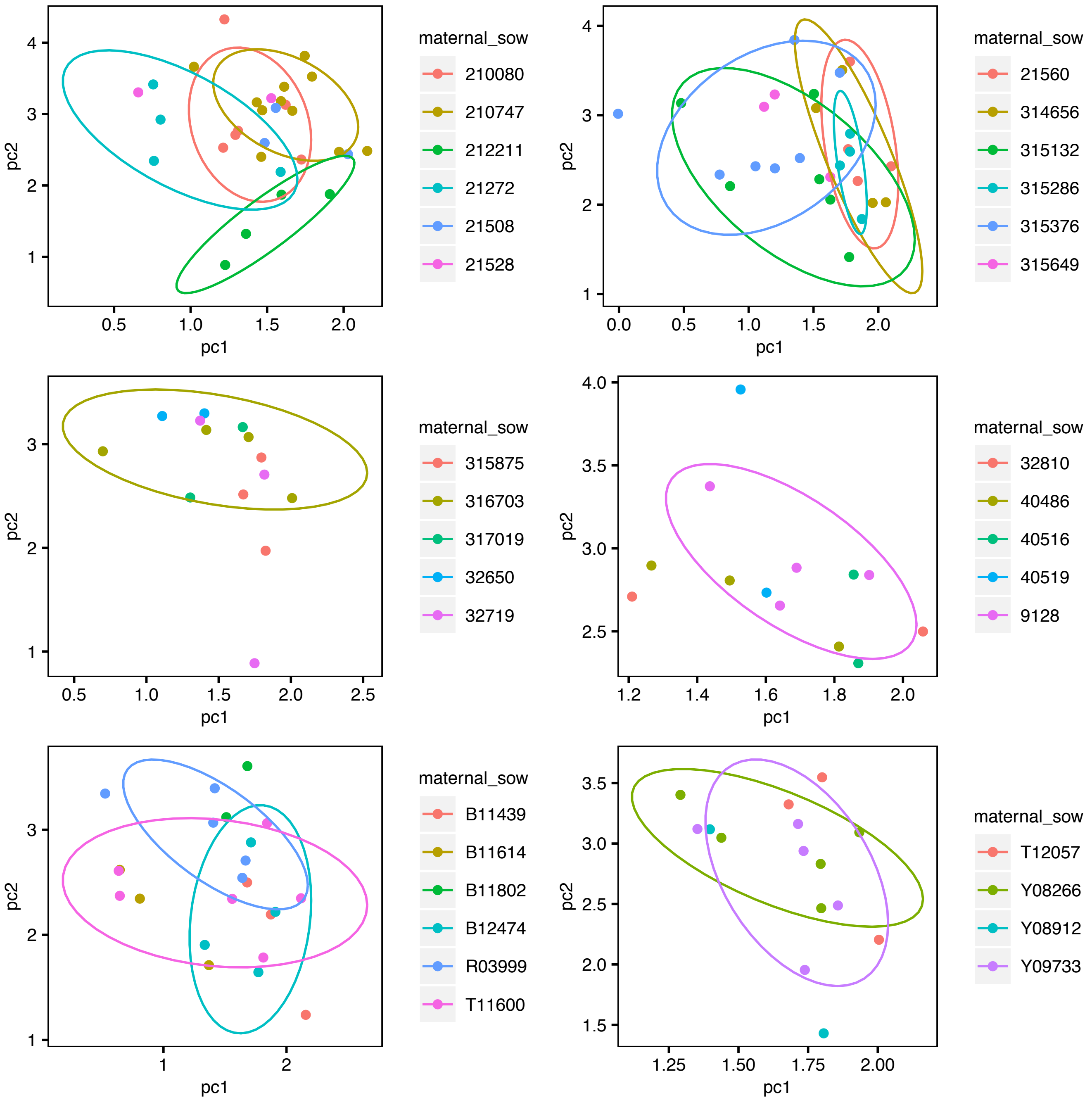

Supplementary Figure 19. Principal component analysis of piglet samples by maternal sow.

First two principal components of beta phylogenetic diversity. Principal component 1 (PC1) and principal component 2 (PC2) explaining 46.99% and 21.68% of the variation, respectively. Samples from piglets at the start of the trial (one time point -sample per piglet) colored by maternal sow (n=32). Ellipse is drawn at 0.80 confidence level.

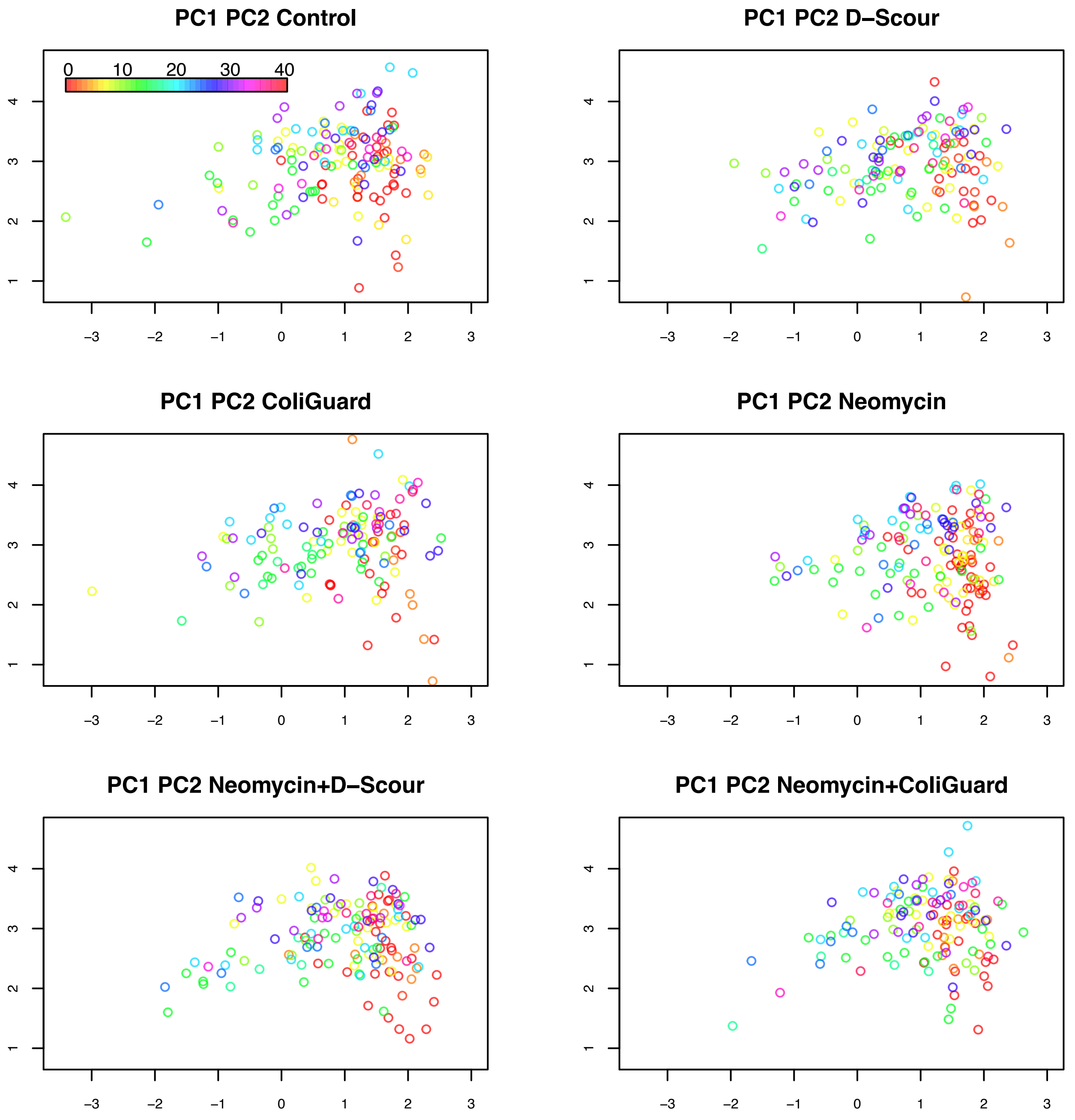

Supplementary Figure 20. Time effect on beta diversity per cohort.

Edge principal component analysis of all samples coloured by sample collection time across the trial (day 0: red; day 10: yellow; day 20: green; day 30: light blue; day 40: dark blue) where principal component 1 (PC1) explains 46.99% of the variation and principal component 2 (PC2) explains 21.68% of the variation. Each plot displays all samples within each cohort.

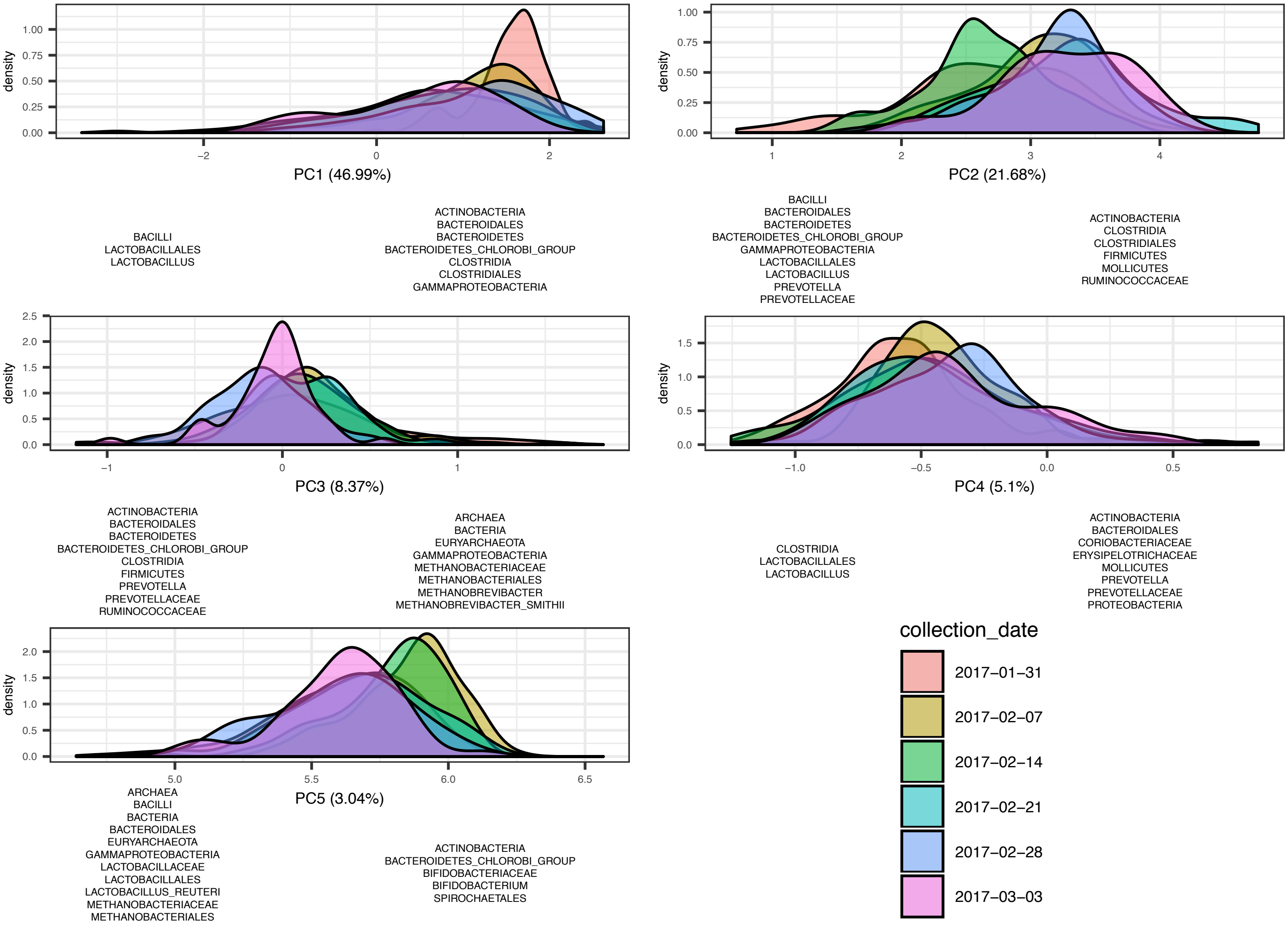

Supplementary Figure 21. Time effect on beta diversity.

Each plot represents the samples distribution along one of the five principal components across time. Most variation is explained by PC1 (46.99%), followed by PC2 (21.68%), PC3 (8.37%), PC4 (5.1%) and PC5 (3.04%). Distribution of the samples on either side of a plot (left *versus* right) reflects the taxa that were found to explain the variation. The distributions are colour coded by time point during the trial.

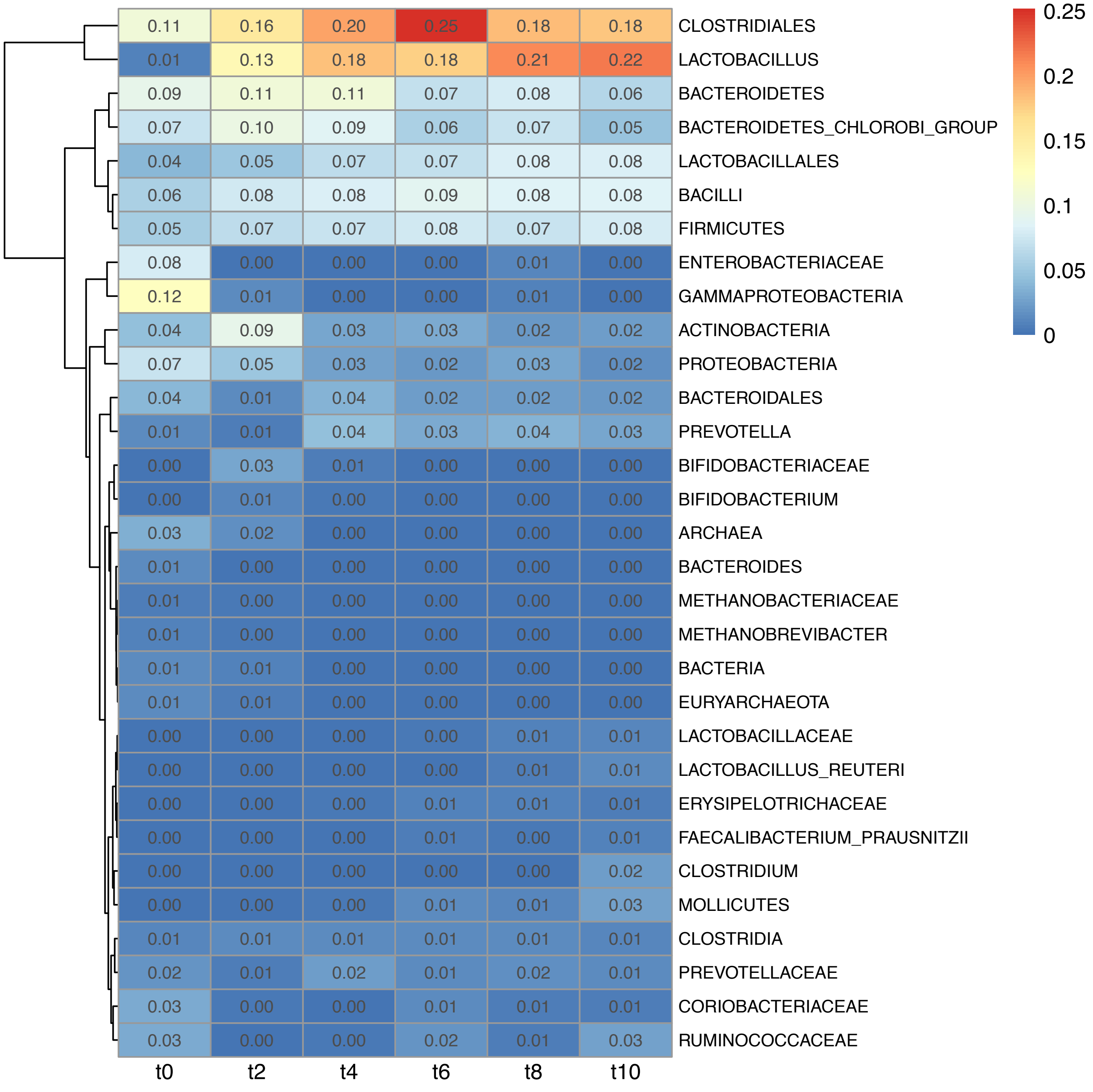

Supplementary Figure 22. Taxa displaying the highest variation in beta diversity across time.

Heatmap of taxa explaining the community composition of samples from separate time points of the trial (1 week interval between time points) derived from edge principal component analysis. Intensity is derived from branch width by the percentage of variability explained by the principal components.

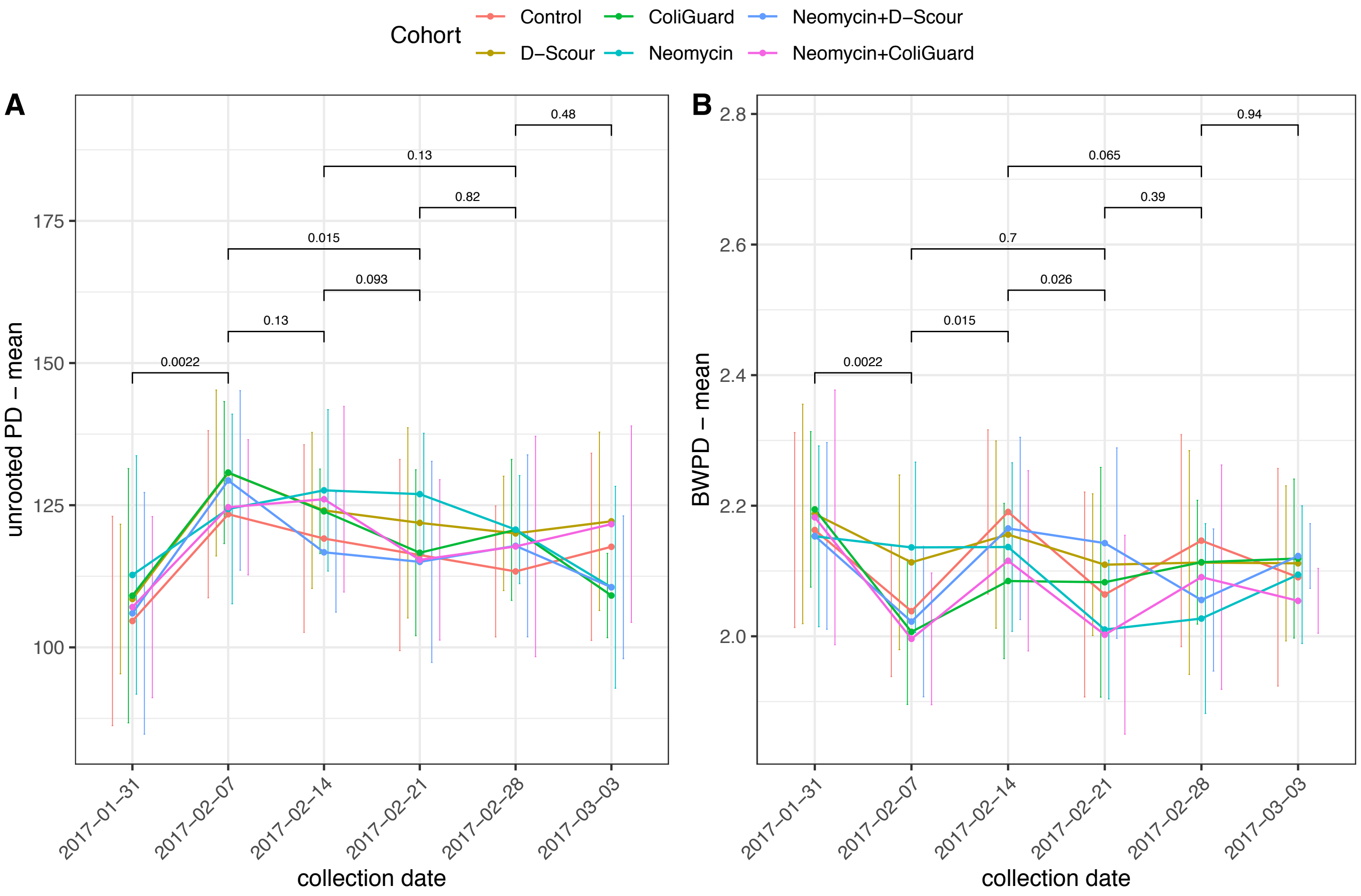

Supplementary Figure 23. Time trend of alpha diversity by cohort.

Unrooted PD (**A**) and BWPD (**B**) describe richness and evenness, respectively, of alpha phylogenetic diversity for all samples across time, grouped and colour coded by cohort. The *p* values derived from pairwise comparisons of time points within each of the cohorts. *P* values and *post-hoc* corrected *p* values of time points comparisons for each separate cohort are provided in Supplementary File 5.

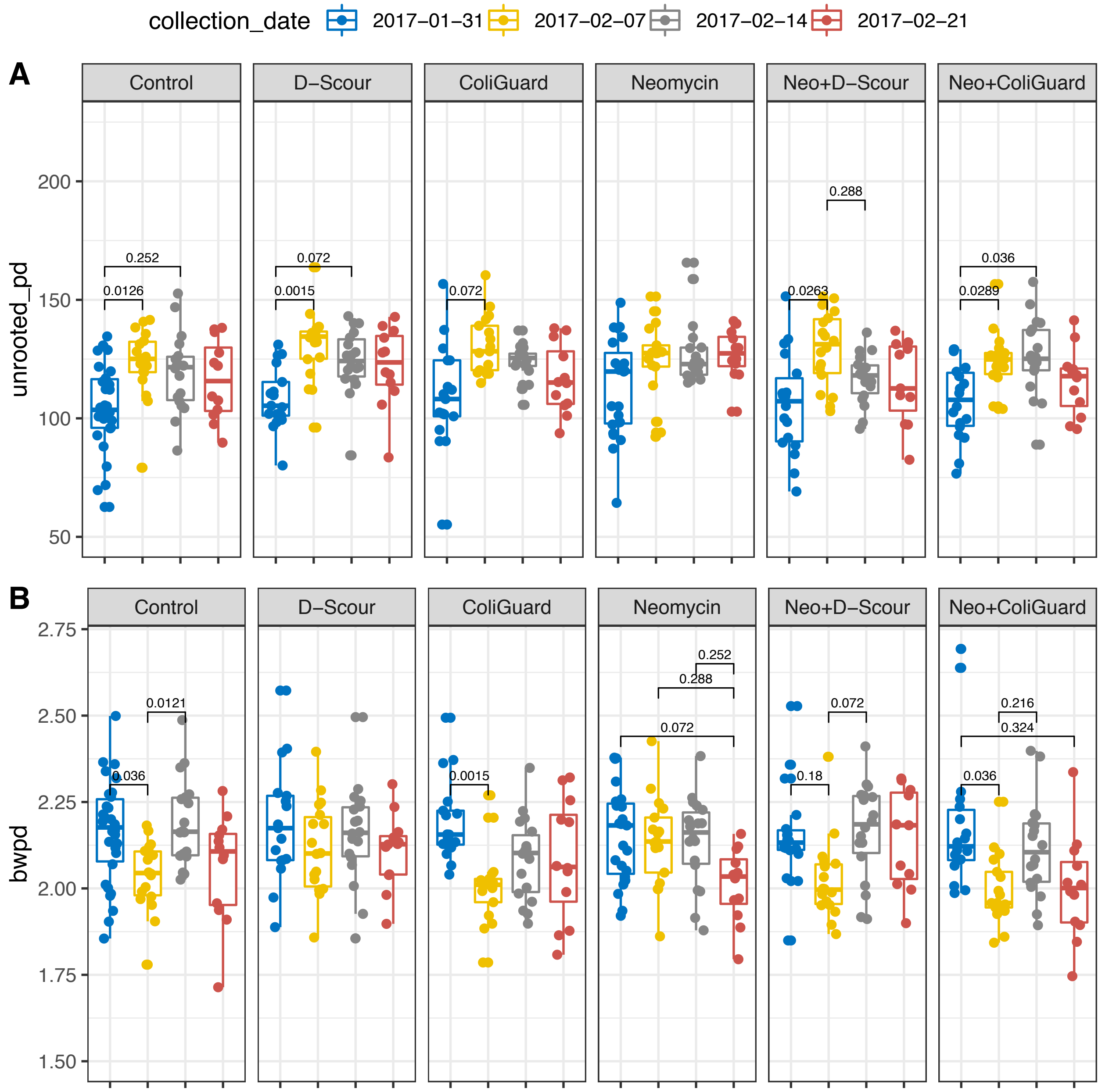

Supplementary Figure 24 Alpha diversity by cohort across four time points.

Alpha phylogenetic diversity for each cohort for a subset of 4 time points. *P* values are derived from pairwise comparisons of the means of each time point of (**A**) unrooted phylogenetic diversity and (**B**) balance weighted phylogenetic diversity. Bonferroni correction of *p* values is applied. More time points were tested and significance was determined; reported in Supplementary File 5.

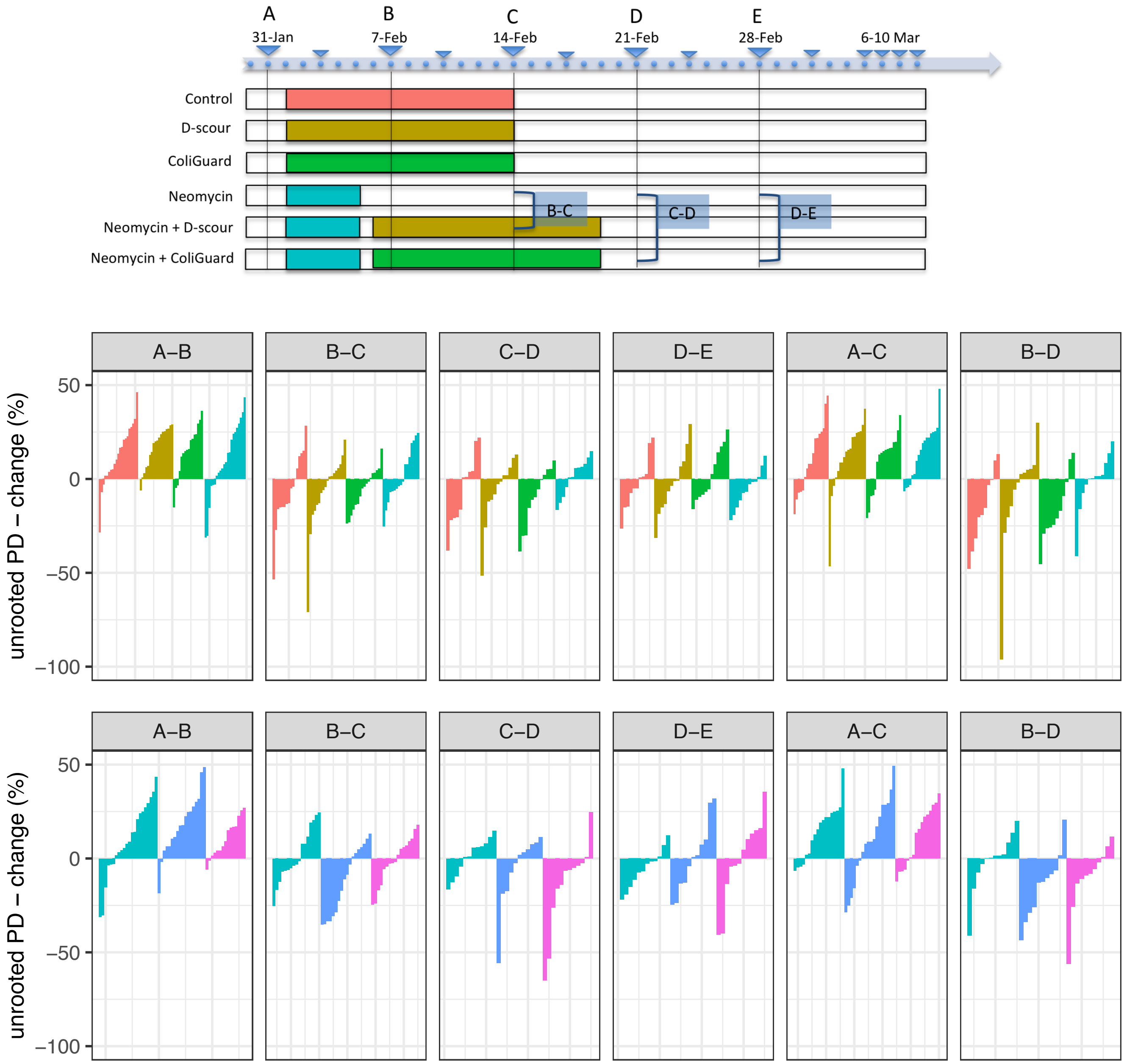

Supplementary Figure 25. Deltas of unrooted alpha phylogenetic diversity between time points.

Change of alpha unrooted phylogenetic diversity in samples between time points across the trial. On the y-axis of plots the percentage difference in unrooted PD between time points per cohort. Letters on the top left of each plot indicate the time points compared with one week interval (A-B, B-C, C-D, D-E) and two weeks interval (A-C, B-D). Pairwise t-test comparisons between cohorts were computed. Brackets in the timeline (top) indicate a significant *p* value ($p<0.05$) between cohorts (1-week interval); bold brackets indicate a significant *p* value ($p<0.05$) between cohorts (2-weeks interval). * indicate significance reached when applying Benjamini-Hochberg *post-hoc* correction.

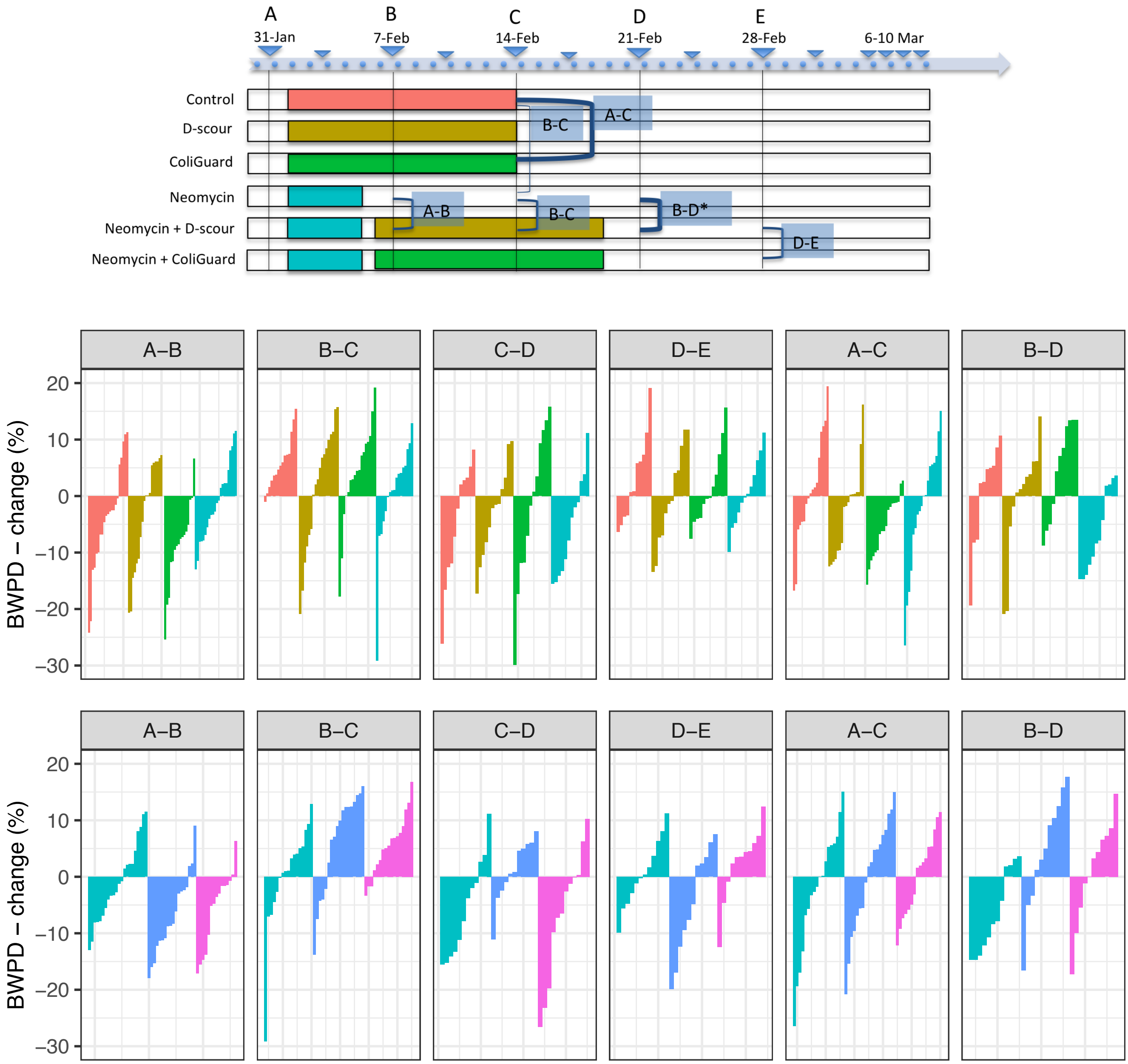

Supplementary Figure 26. Deltas of balance weighted alpha phylogenetic diversity between time points.

Change of alpha balance weighted phylogenetic diversity in samples between time points across the trial. On the y-axis of plots the percentage difference in balance weighted phylogenetic diversity between time points per cohort. Letters on the top left of each plot indicate the time points compared with one week interval (A-B, B-C, C-D, D-E) and two weeks interval (A-C, B-D). Pairwise t-test comparisons between cohorts were computed. Brackets in the timeline (top) indicate a significant *p* value ($p<0.05$) between cohorts (1-week interval); bold brackets indicate a significant *p* value ($p<0.05$) between cohorts (2-weeks interval). * indicate significance reached when applying Benjamini-Hochberg *post-hoc* correction.

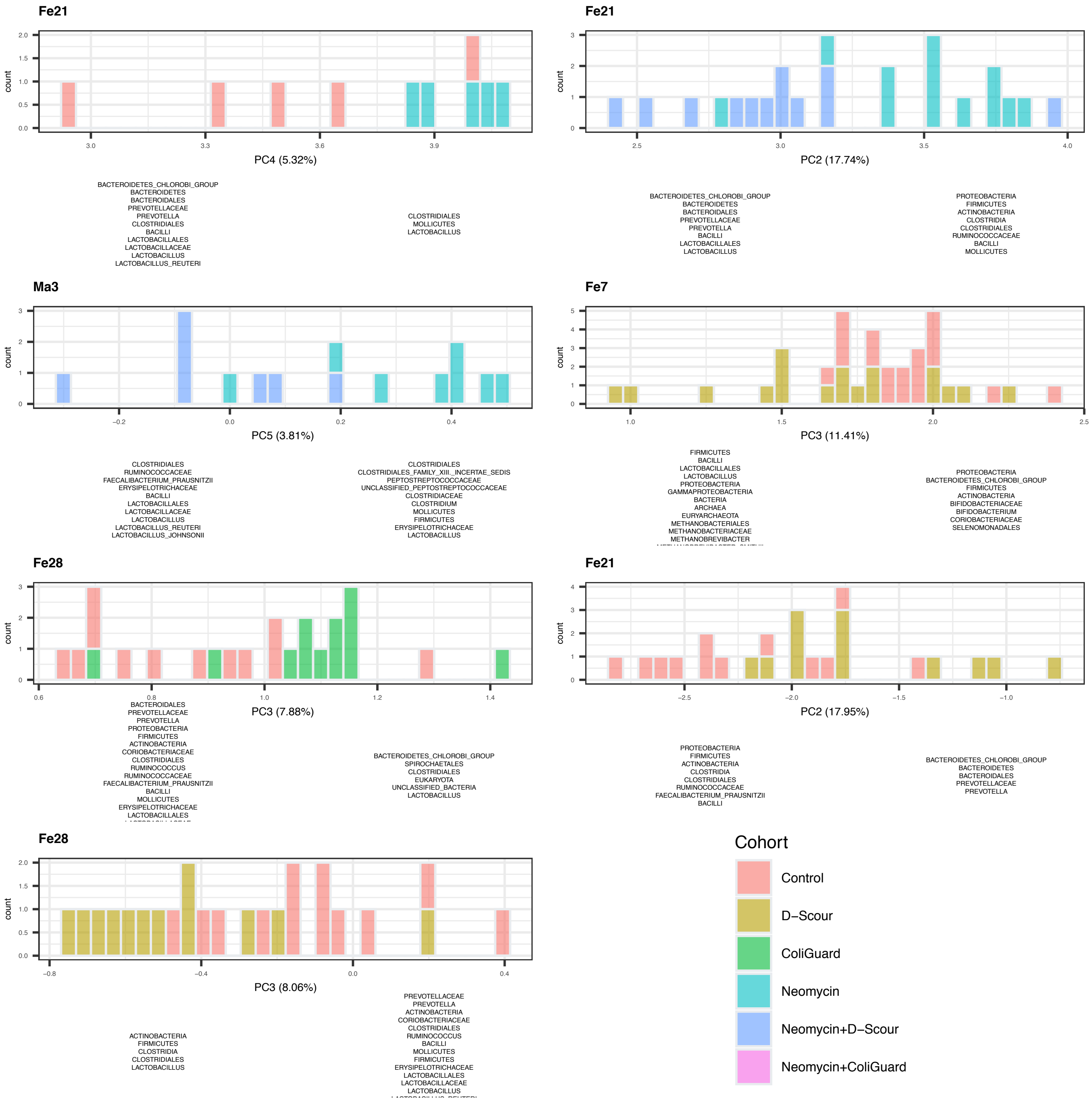

Supplementary Figure 27. Significant differences in beta diversity between cohorts at specific time points.

Significance was determined by comparing groups by pairwise t-test and the resulting *p* values where adjusted with the Bonferroni method. Significance values are provided in Supplementary File 5. The x-axes represent the principal component. As plots are derived from distinct guppy runs, each principal component explains variation to a different extent (percentage specified in parentheses). The number of samples is specified on the y-axis. Distribution of the samples on either side of a plot (left *versus* right) reflects the taxa that were found to explain the variation. Distributions are colour coded by cohort.

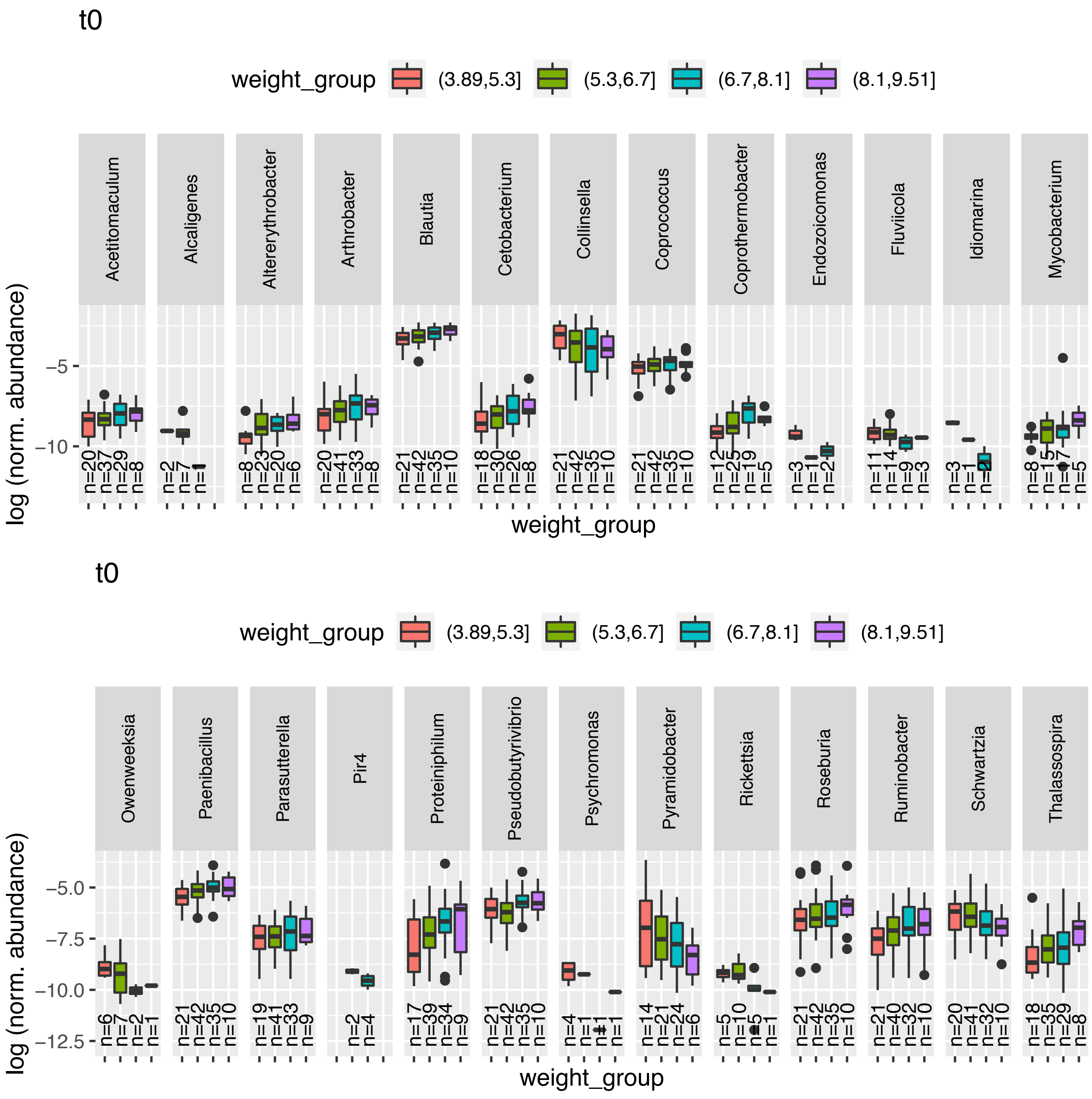

Supplementary Figure 28. Correlation of weight with taxonomy at time point 0.

16S rRNA reads were extracted with SortMeRNA. Abundance was estimated and correlation with weight was assessed with the Spearman method. Significance values are provided in Supplementary File 5. Plotted are the correlations with p value < 0.05. For visualization purposes, subjects are binned into weight groups (x-axis). Bins are formed with subjects whose weight falls below the first quartile (red), above the first quartile (green), below the third quartile (blue), above the third quartile (purple). At the bottom of each whisker plot, the samples size for each bin is provided.

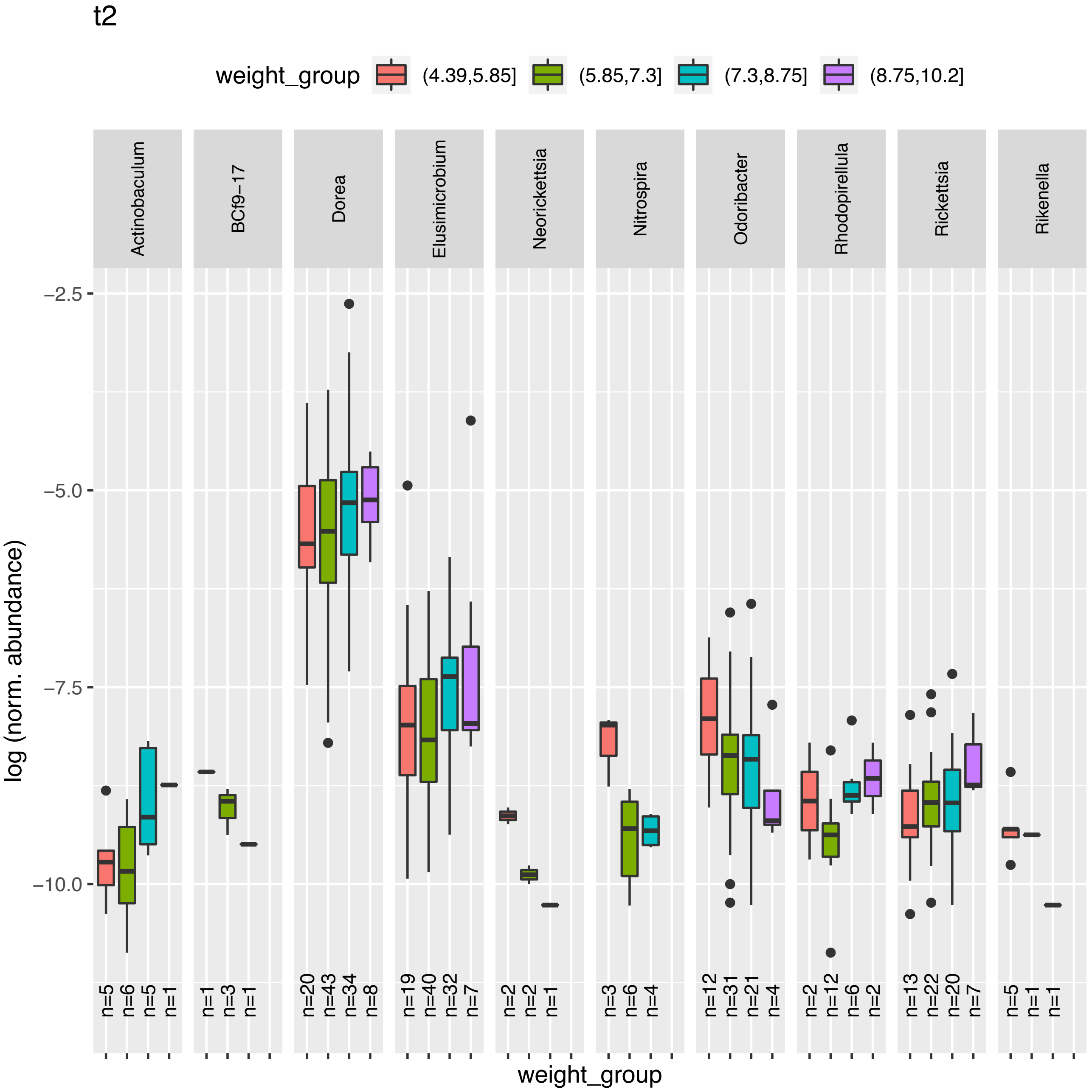

Supplementary Figure 29. Correlation of weight with taxonomy at time point 2.

16S rRNA reads were extracted with SortMeRNA. Abundance was estimated and correlation with weight was assessed with the Spearman method. Significance values are provided in Supplementary File 5. Plotted are the correlations with p value < 0.05. For visualization purposes, subjects are binned into weight groups (x-axis). Bins are formed with subjects whose weight falls below the first quartile (red), above the first quartile (green), below the third quartile (blue), above the third quartile (purple). At the bottom of each whisker plot, the samples size for each bin is provided.

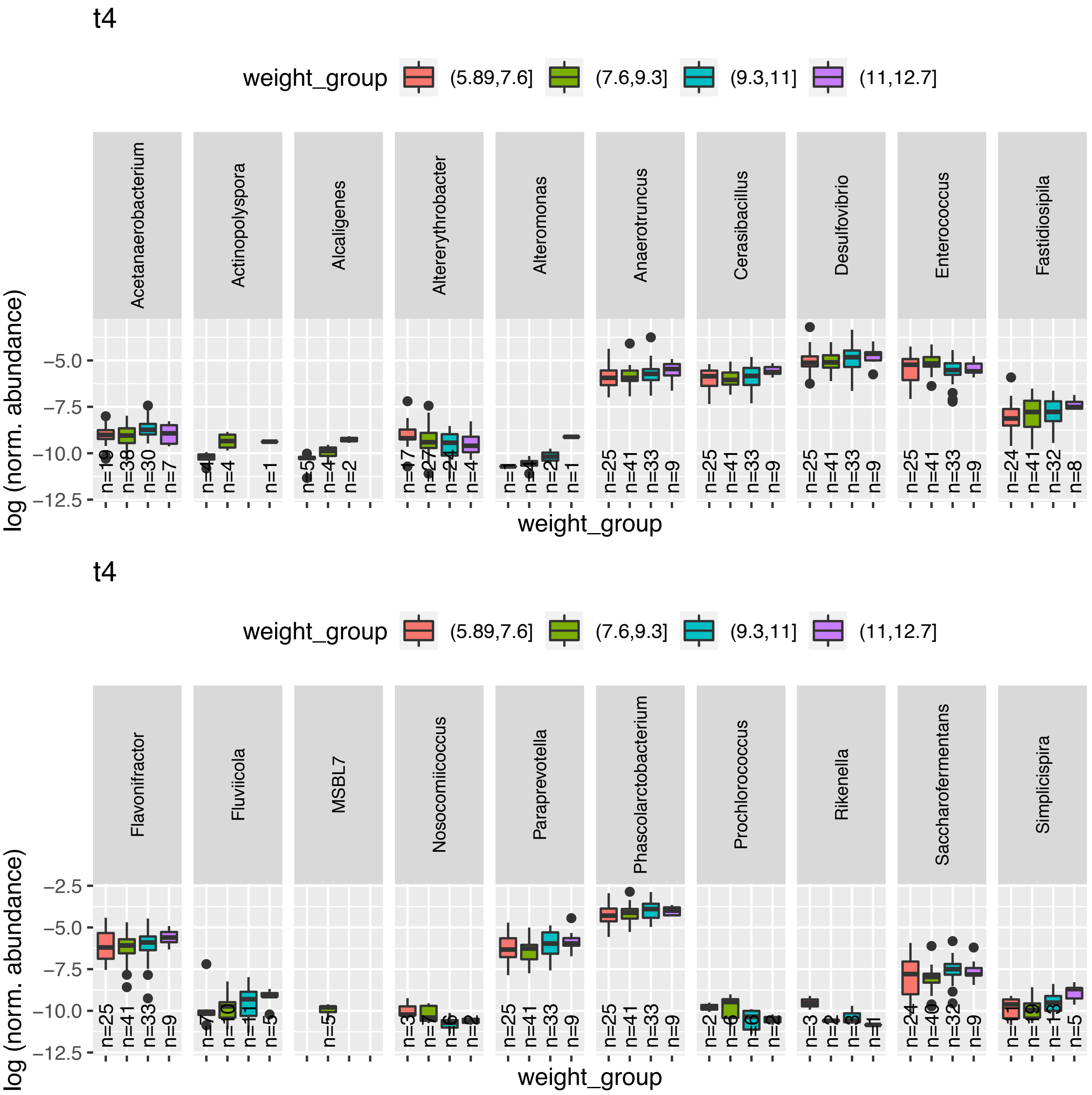

Supplementary Figure 30. Correlation of weight with taxonomy at time point 4.

16S rRNA reads were extracted with SortMeRNA. Abundance was estimated and correlation with weight was assessed with the Spearman method. Significance values are provided in Supplementary File 5. Plotted are the correlations with p value < 0.05. For visualization purposes, subjects are binned into weight groups (x-axis). Bins are formed with subjects whose weight falls below the first quartile (red), above the first quartile (green), below the third quartile (blue), above the third quartile (purple). At the bottom of each whisker plot, the samples size for each bin is provided.

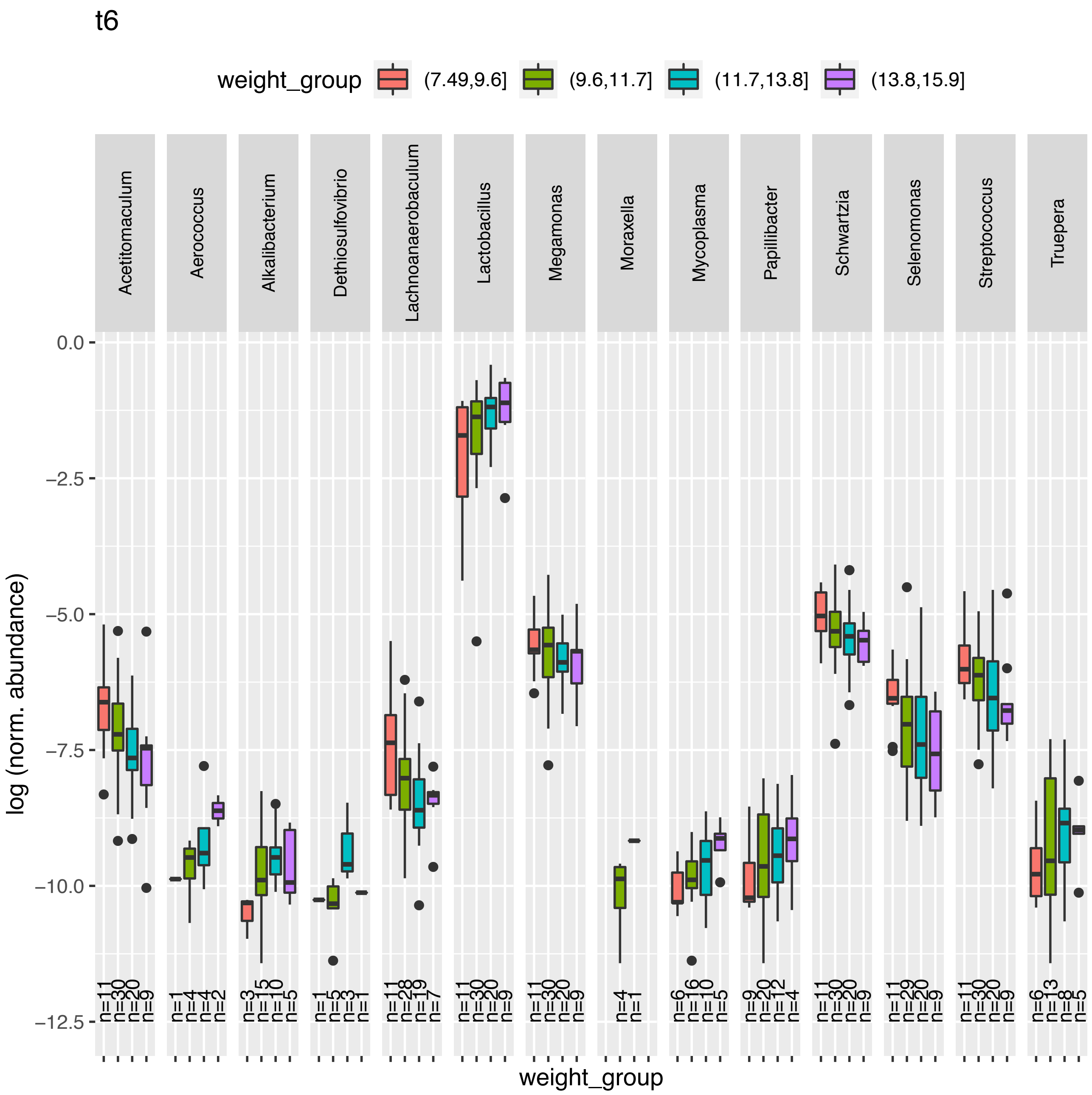

Supplementary Figure 31. Correlation of weight with taxonomy at time point 6.

16S rRNA reads were extracted with SortMeRNA. Abundance was estimated and correlation with weight was assessed with the Spearman method. Significance values are provided in Supplementary File 5. Plotted are the correlations with p value < 0.05. For visualization purposes, subjects are binned into weight groups (x-axis). Bins are formed with subjects whose weight falls below the first quartile (red), above the first quartile (green), below the third quartile (blue), above the third quartile (purple). At the bottom of each whisker plot, the samples size for each bin is provided.

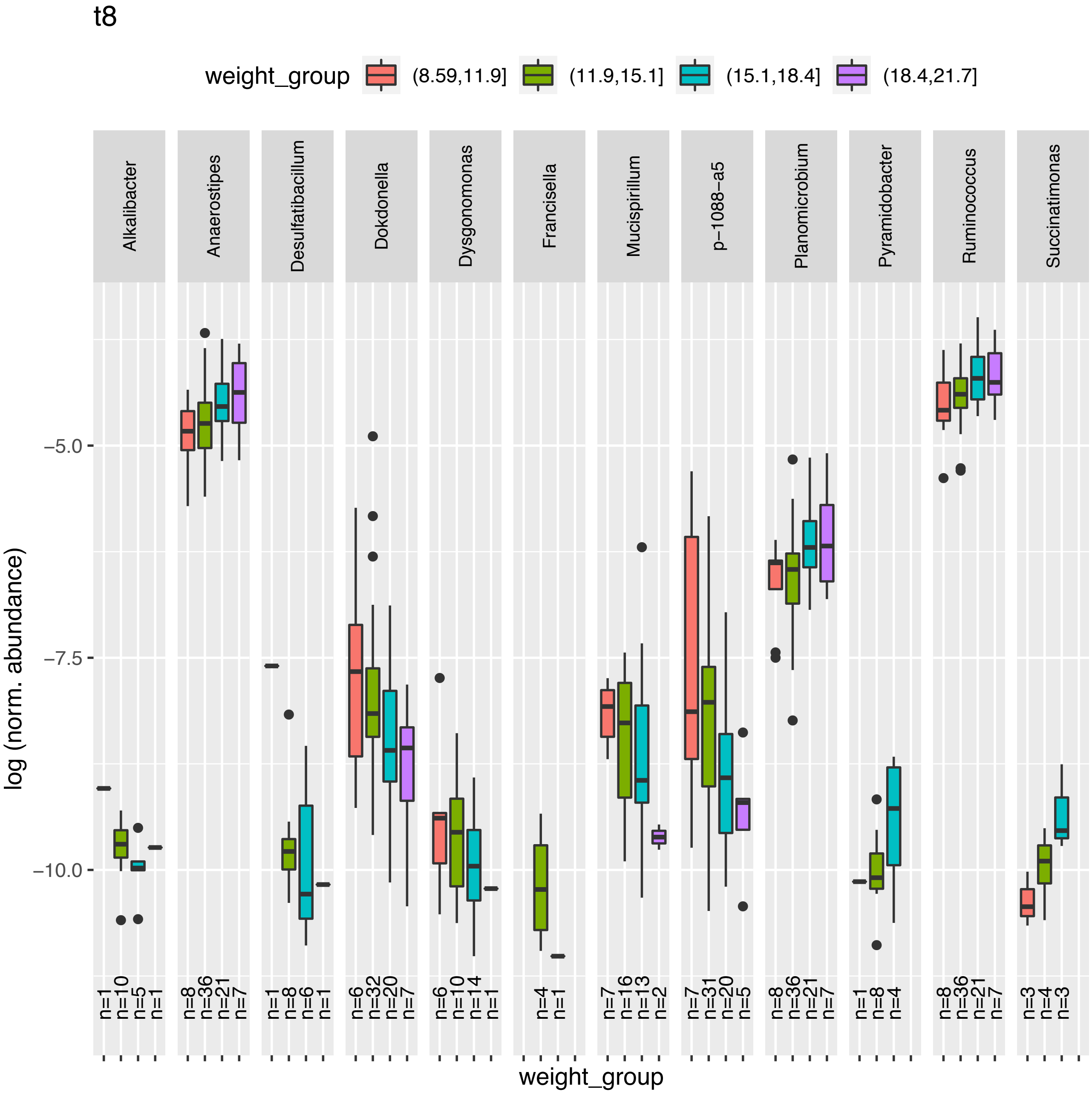

Supplementary Figure 32. Correlation of weight with taxonomy at time point 8.

16S rRNA reads were extracted with SortMeRNA. Abundance was estimated and correlation with weight was assessed with the Spearman method. Significance values are provided in Supplementary File 5. Plotted are the correlations with p value < 0.05. For visualization purposes, subjects are binned into weight groups (x-axis). Bins are formed with subjects whose weight falls below the first quartile (red), above the first quartile (green), below the third quartile (blue), above the third quartile (purple). At the bottom of each whisker plot, the samples size for each bin is provided.

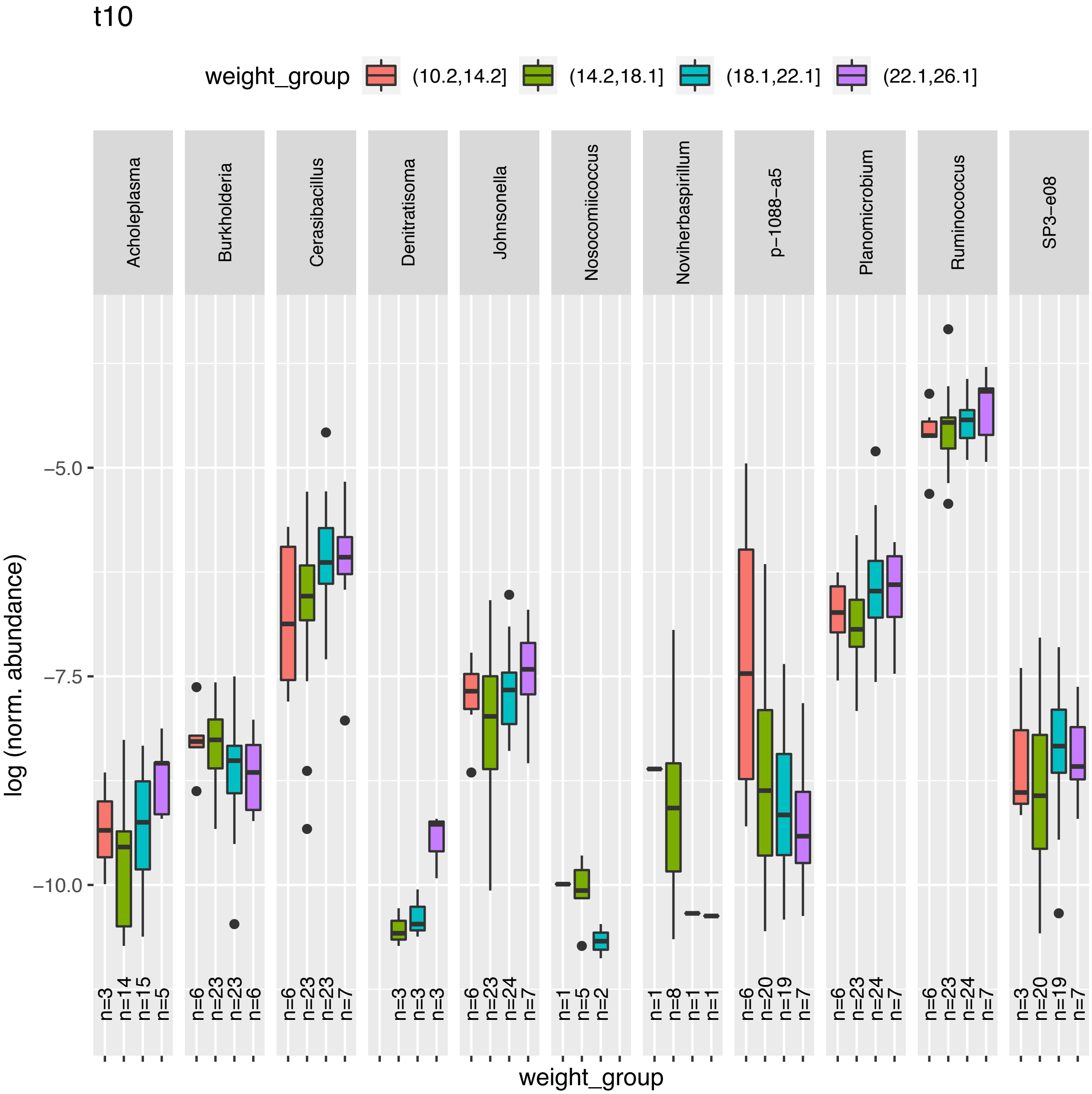

Supplementary Figure 33. Correlation of weight with taxonomy at time point 10.

16S rRNA reads were extracted with SortMeRNA. Abundance was estimated and correlation with weight was assessed with the Spearman method. Significance values are provided in Supplementary File 5. Plotted are the correlations with *p* value < 0.05. For visualization purposes, subjects are binned into weight groups (x-axis). Bins are formed with subjects whose weight falls below the first quartile (red), above the first quartile (green), below the third quartile (blue), above the third quartile (purple). At the bottom of each whisker plot, the samples size for each bin is provided.

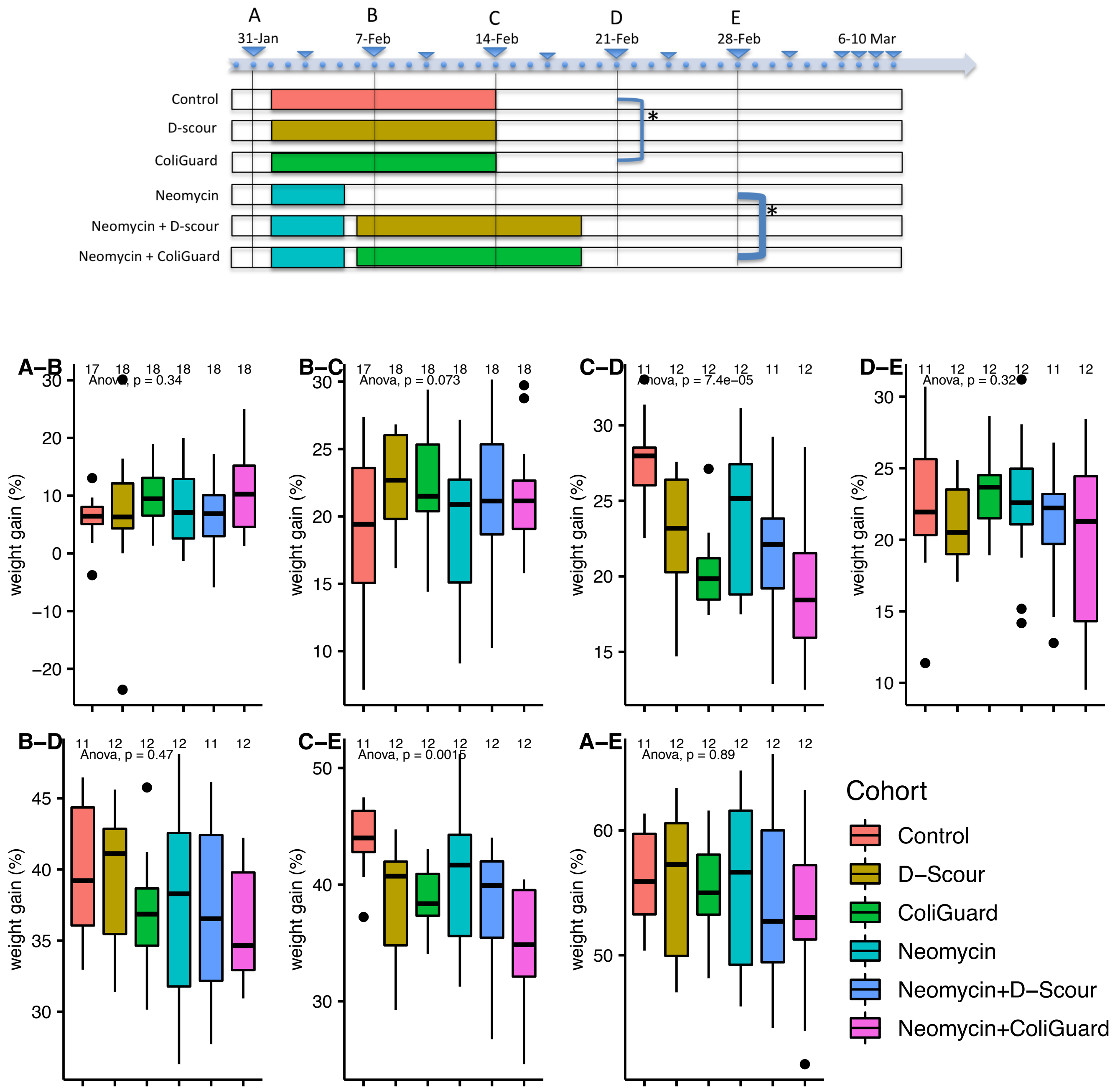

Supplementary Figure 34. Weight gain of piglets by cohort across the trial.

On the y-axis of plots the weight gain between time points is provided in percentage. Letters on the top left of each plot indicate the time points compared with one week interval (A-B, B-C, C-D, D-E) and with two weeks interval (B-D, C-E, A-E). Pairwise t-test comparisons between cohorts were computed. Brackets in the timeline (top) indicate a significant *p* value between cohorts (1-week interval); bold brackets indicate a significant *p* value between cohorts (2-weeks interval). A significant difference was found between Control and ColiGuard® (C-D, Tukey adjusted *p* value=0.000747) and between neomycin and neomycin+ColiGuard® (C-E, Tukey adjusted *p* value=0.039286). *in the timeline indicate that the significance was maintained after applying Tukey HSD *post-hoc* correction.

Click here to access/download;Figure;file-1.pdf

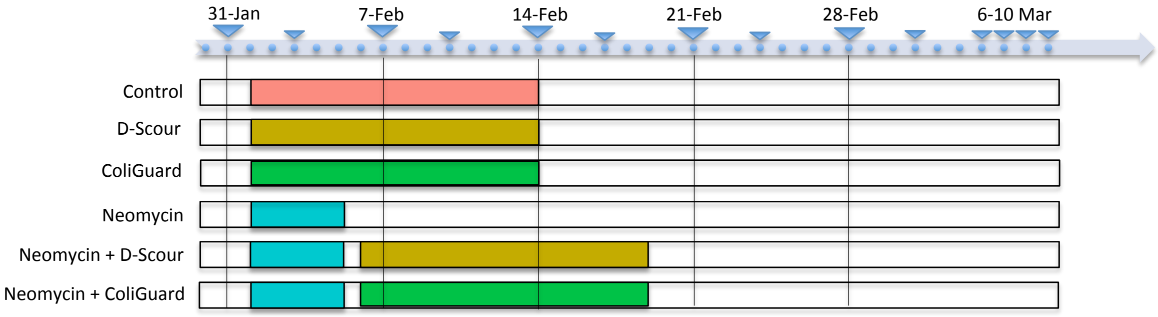

Figure 1. Timeline.

Timeline of the trial indicating the start and the length of the treatment for each cohort. Piglets arrived on the site of the trial on January 30th and were allowed 2 days of acclimatisation before the start of the treatments (pink: placebo paste; yellow: probiotic D-Scour™ formulation; green: probiotic ColiGuard® formulation; blue: antibiotic neomycin intramuscular injection). Vertical lines indicate main days of sampling where all piglets where sampled (n=126). Small arrows indicate days of sampling for a subset of the piglets (8 per cohort; n=48).

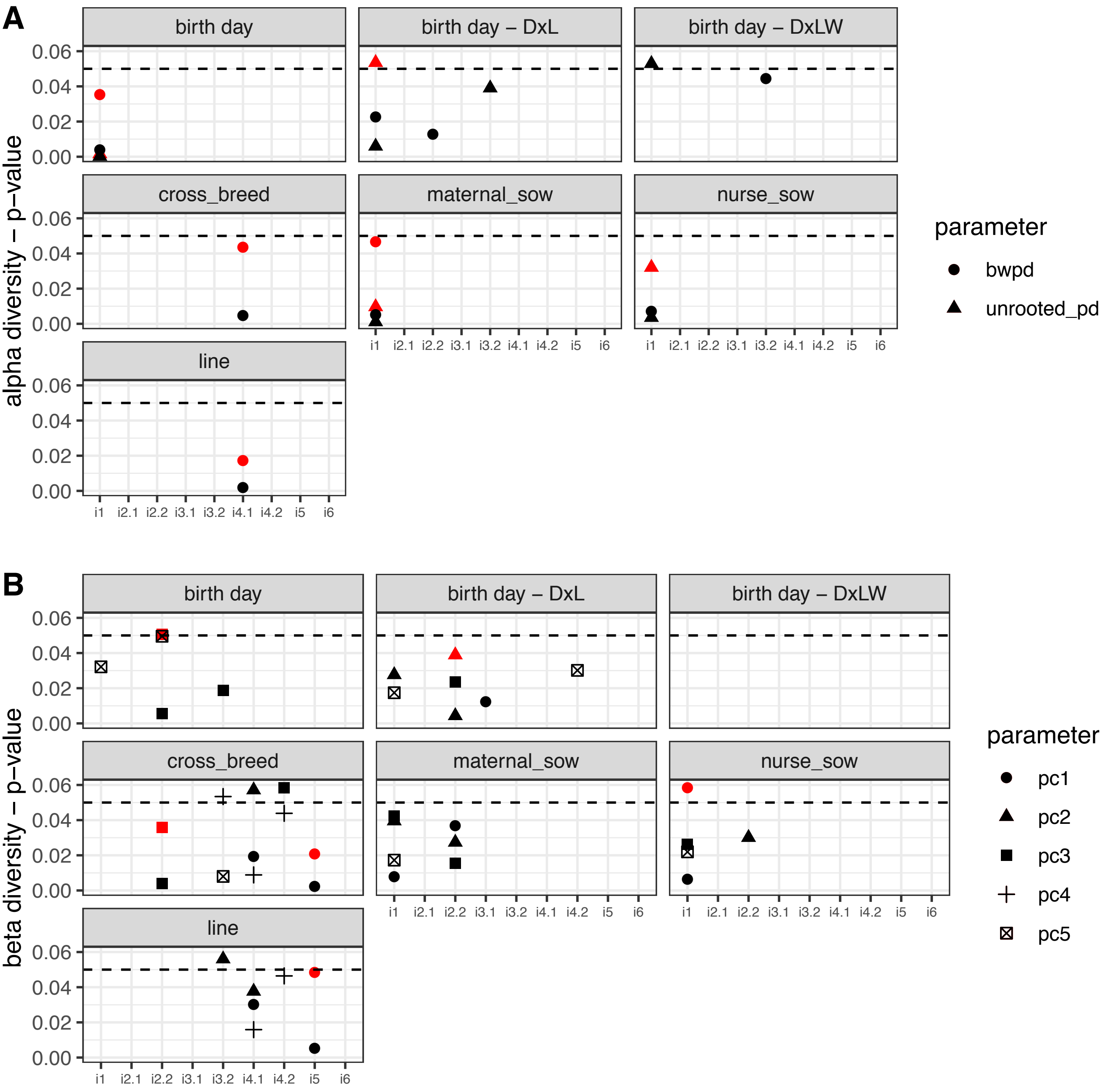

Figure 2. Significance of correlations between phylogenetic diversity and specified factors.

Plots of *p* values derived from Kruskal-Wallis analysis of variance (black) and adjusted *p* values by Benjamini-Hochberg method (red). x-axis displays distinct sampling time points in chronological order. Correlation of specified factors of samples are shown with (**A**) alpha diversity and (**B**) beta diversity. Abbreviations: DxL = "Duroc x Landrace" cross breed; DxLW = "Duroc x Large White" cross breed.

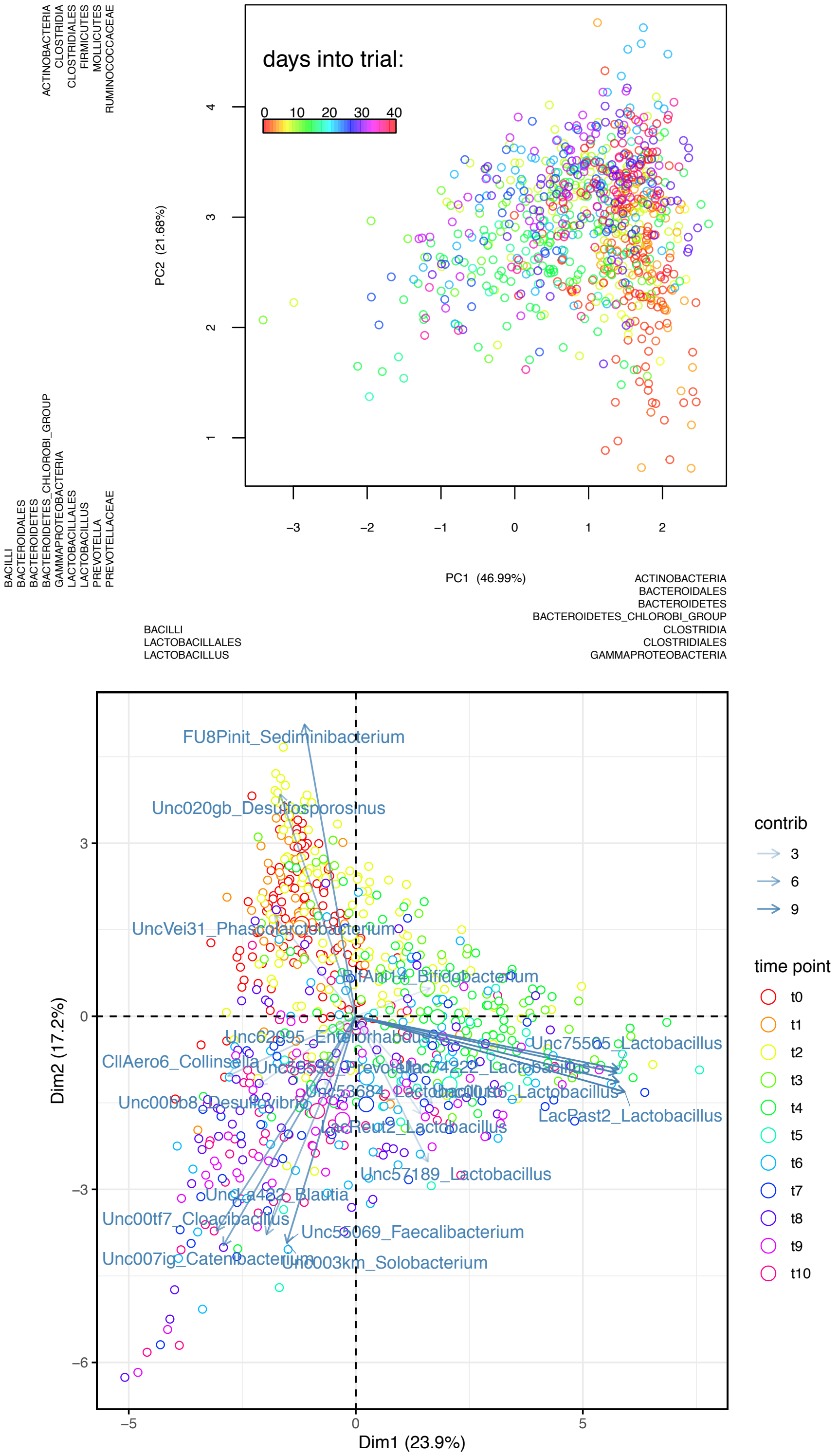

Figure 3. Effect of time on beta diversity.

Principal component analysis (PCA) of samples. PCA from edge component analysis with PhyloSift (top) and PCA from 20 most abundant 16S reads extracted with SortMeRNA (bottom). In the top, distribution of samples on either side of the plot (left *versus* right; top *versus* bottom) reflect the taxa that were found to explain the variation. Samples are colour coded by days into the trial. In the lower plot, arrows indicate which of the 20 taxa read contributed to the variation of samples, where arrows thickness represents a higher (thicker) or lower (thinner) contribution. Samples are colour coded by time point during the trial.

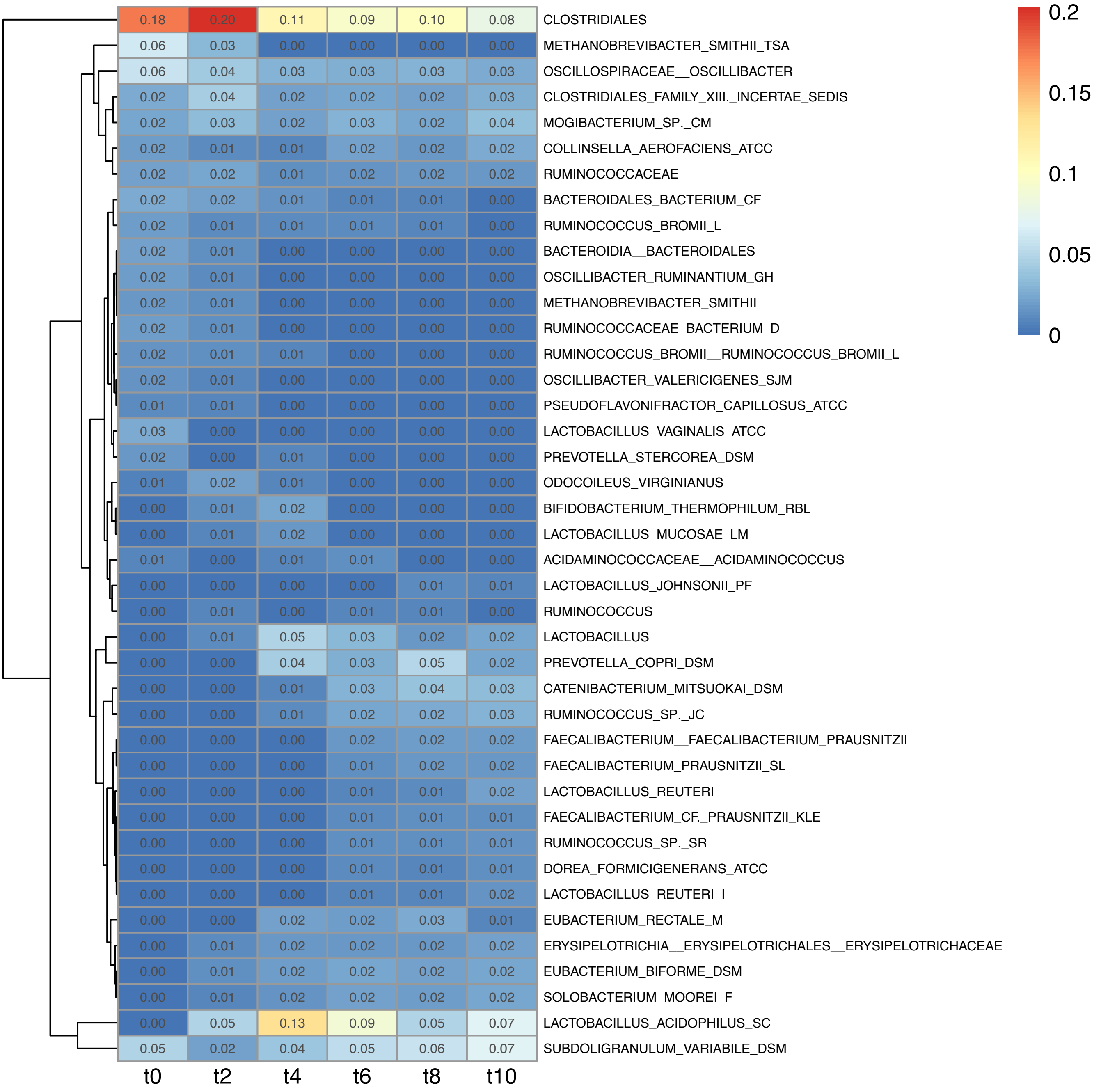

Figure 4. Abundance heatmap.

Most abundant taxa within each time point are obtained from analysis with guppy fat. Abundance is derived from the branch width. The distance between each time point is of one week.

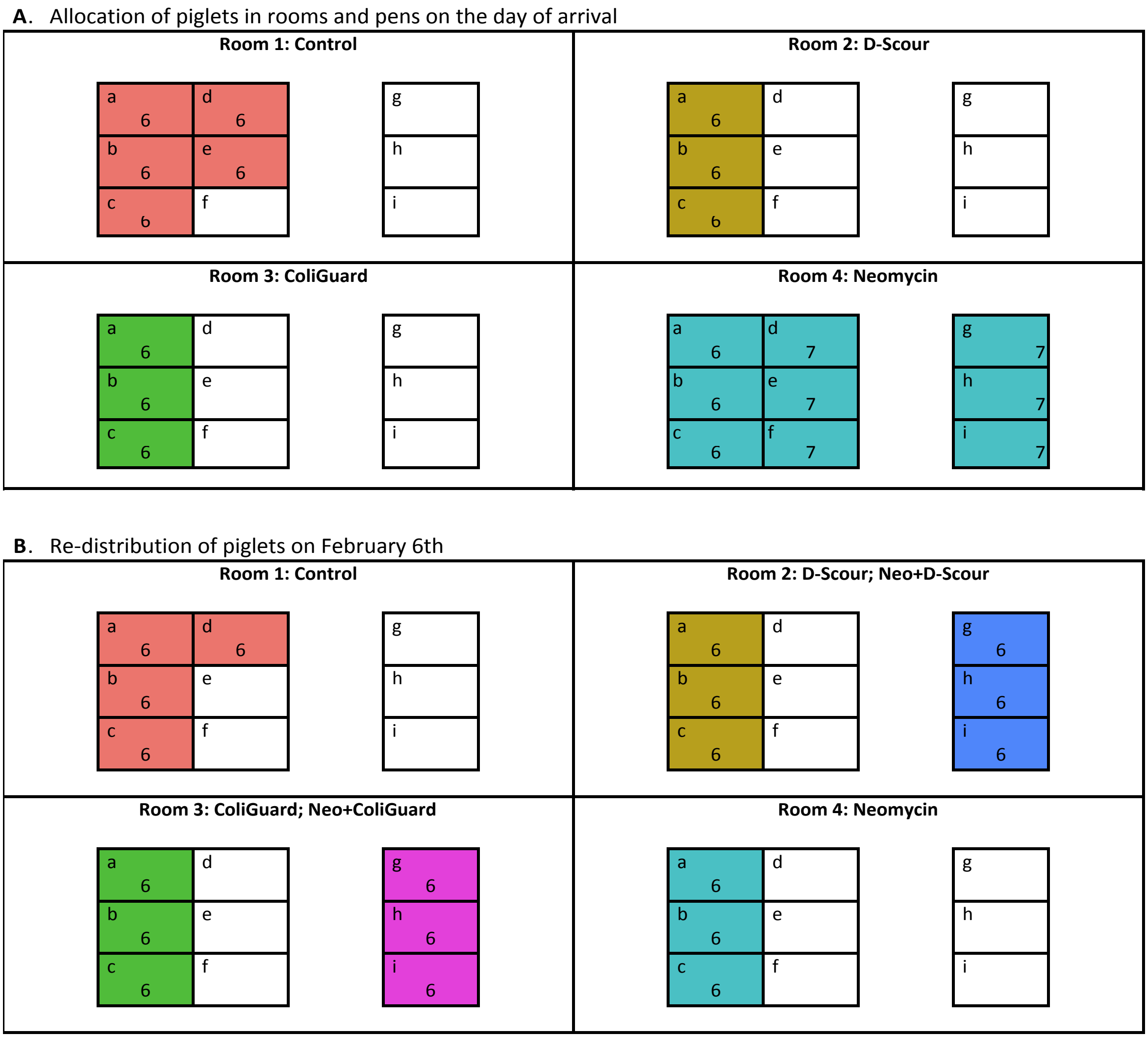

Supplementary Figure 1. Piglets placements across rooms and pens.

Disposition of piglets in rooms (1-4) and pens (a-i) at the start of the trial (**A**) and re-arrangement of a subset of neomycin treated piglets from room 4 to room 2 and room 3, for D-Scour™ and ColiGuard® treatment, respectively (**B**).

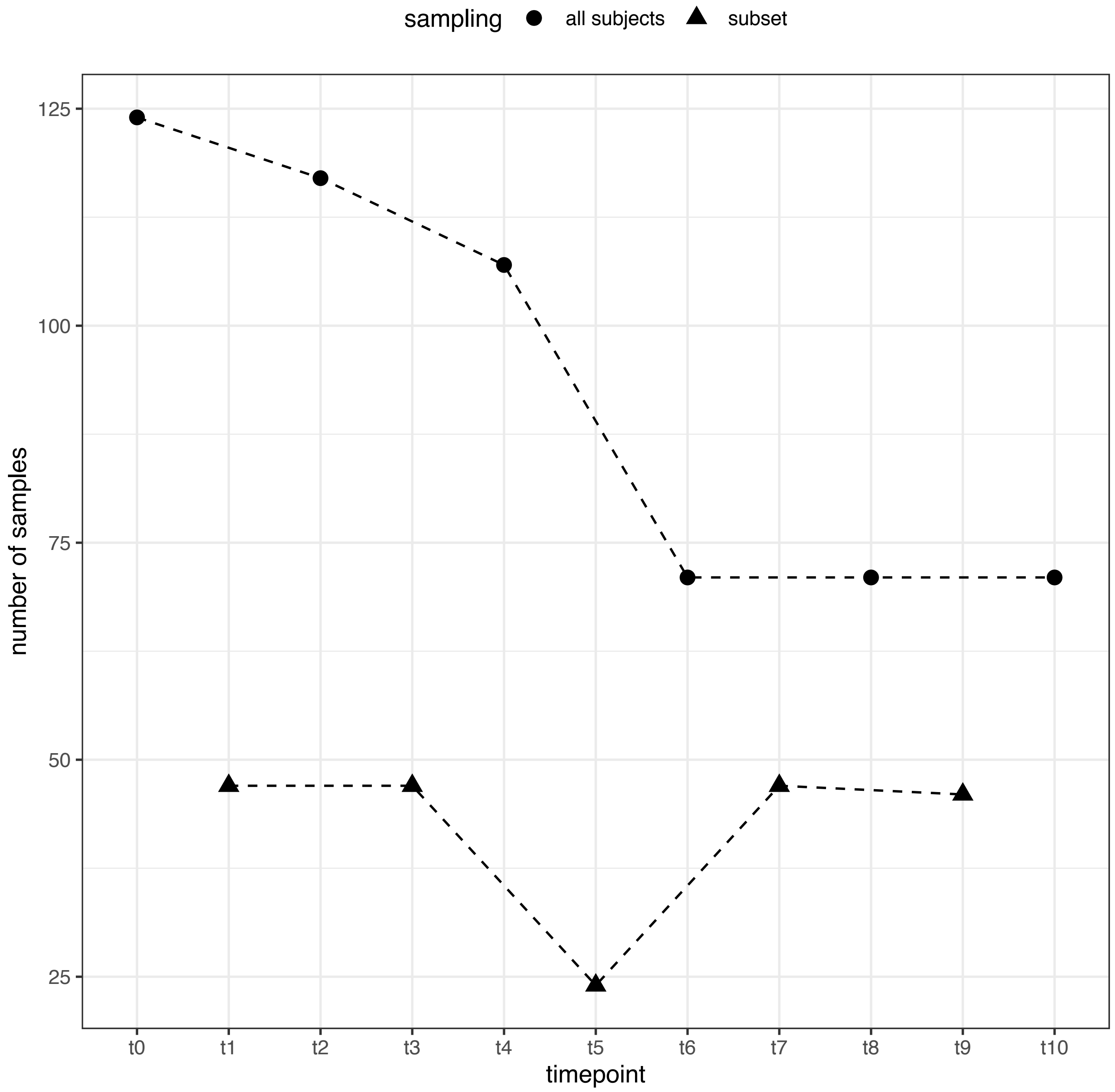

Supplementary Figure 2. Sample collection.

Samples were collected from all piglets once weekly, and from a subset group (8 per cohort; *n*=48) twice weekly. Overall, piglets were sampled between 1 and 10 times.

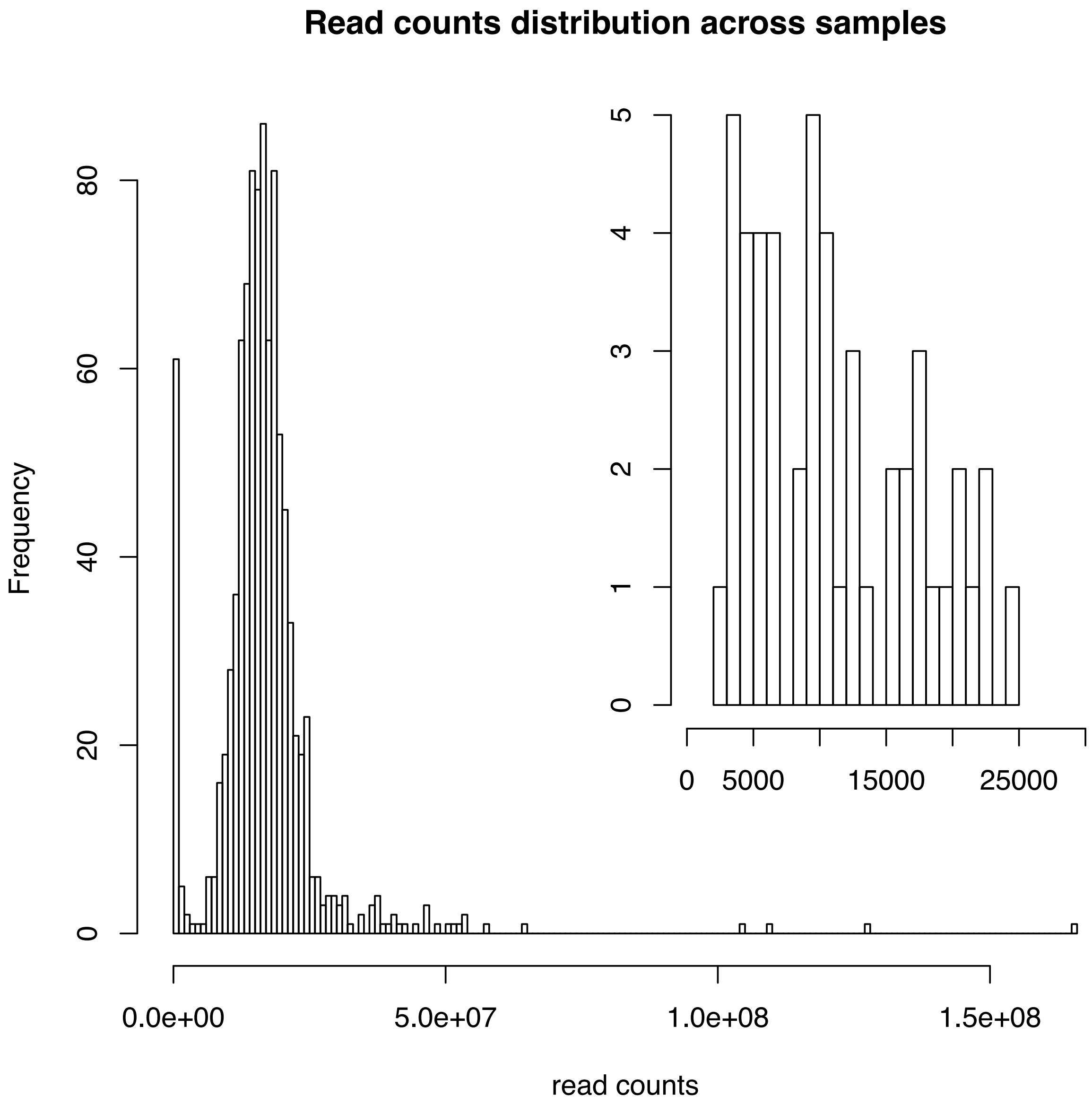

Supplementary Figure 3. Read counts distribution.

Read counts distribution of all samples (main histogram) and low read counts samples (top right histogram).

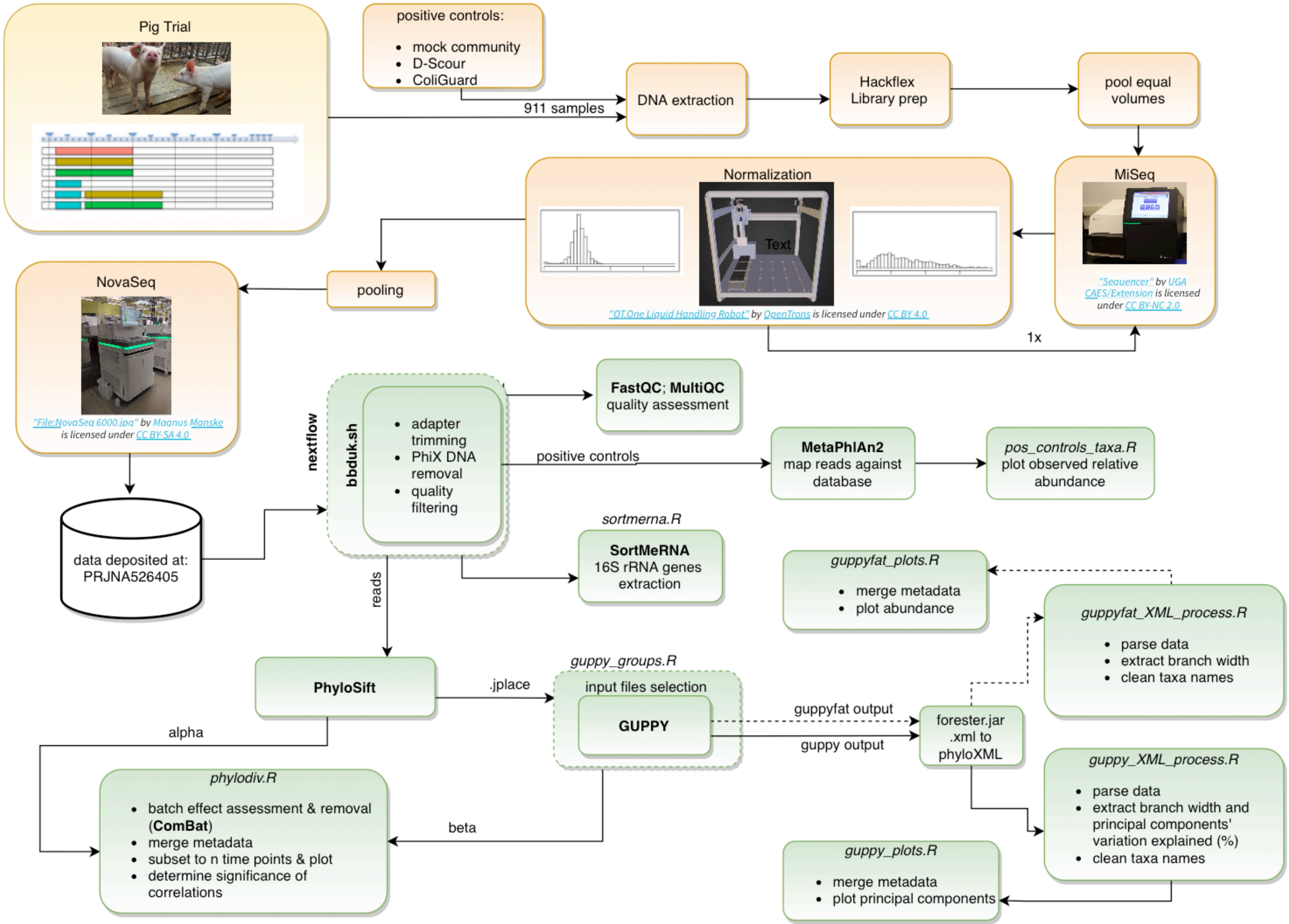

Supplementary Figure 4. Summarised workflow.

Schematic workflow from sample collection to sequencing (orange) and data analysis (green). R scripts (italic) are available in our Github repository.

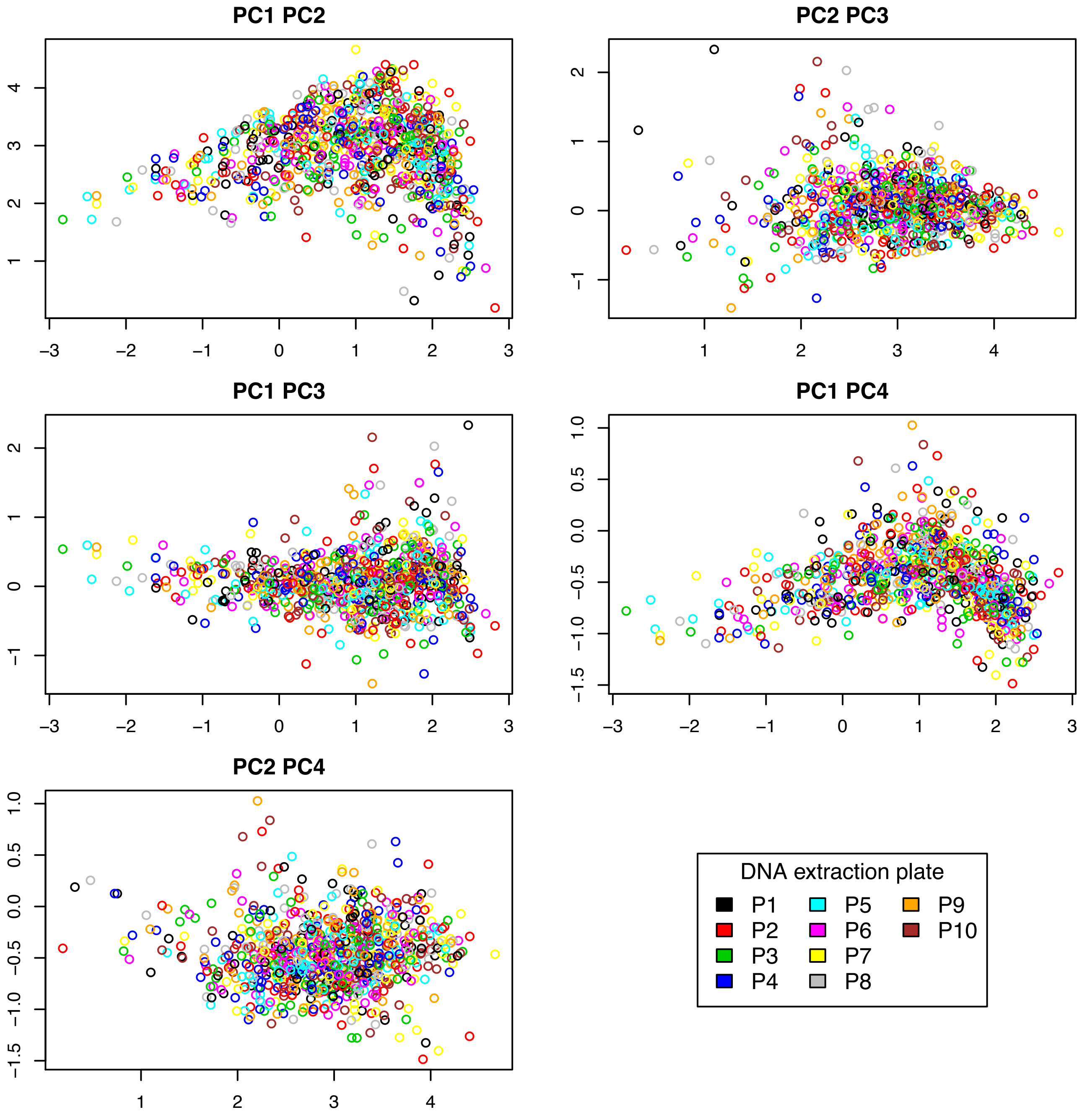

Supplementary Figure 5. Principal component analysis of batch effect before batch effect removal.

Principal components 1 to 4 from beta diversity analysis of all samples before batch effect removal. Samples are coloured by DNA extraction plate. No clustering of samples by DNA extraction plate is visible.

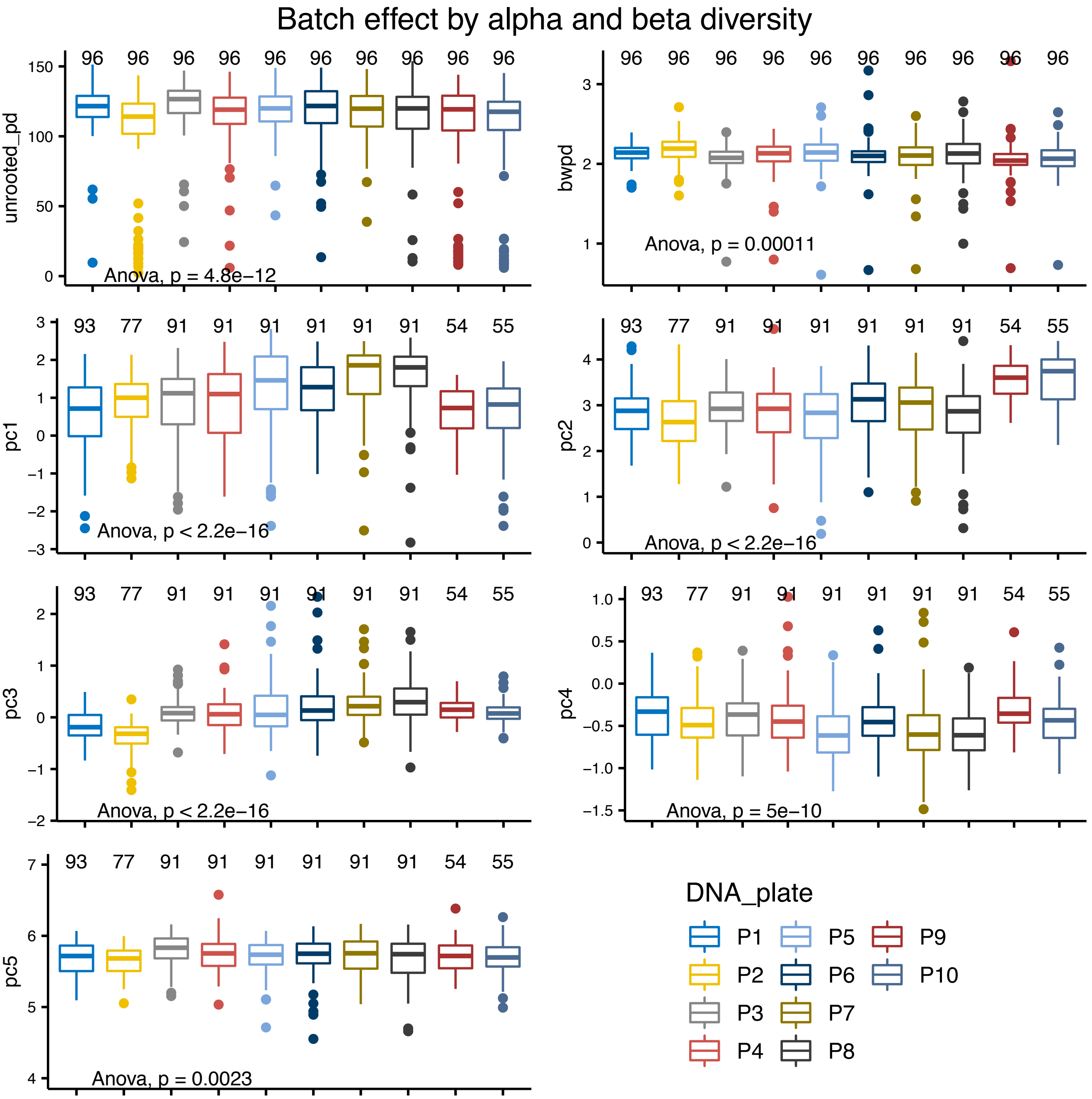

Supplementary Figure 6. Batch effect on alpha and beta diversity before batch effect removal.

Batch effect by alpha (top two plots) and beta diversity (bottom five plots) before batch effect removal. Samples are grouped by DNA extraction plate. The *p* values are derived from multiple comparison analysis with ANOVA, indicating equality of the means. *Post hoc* corrected *p* values for pairwise comparisons are provided in Supplementary File 5.

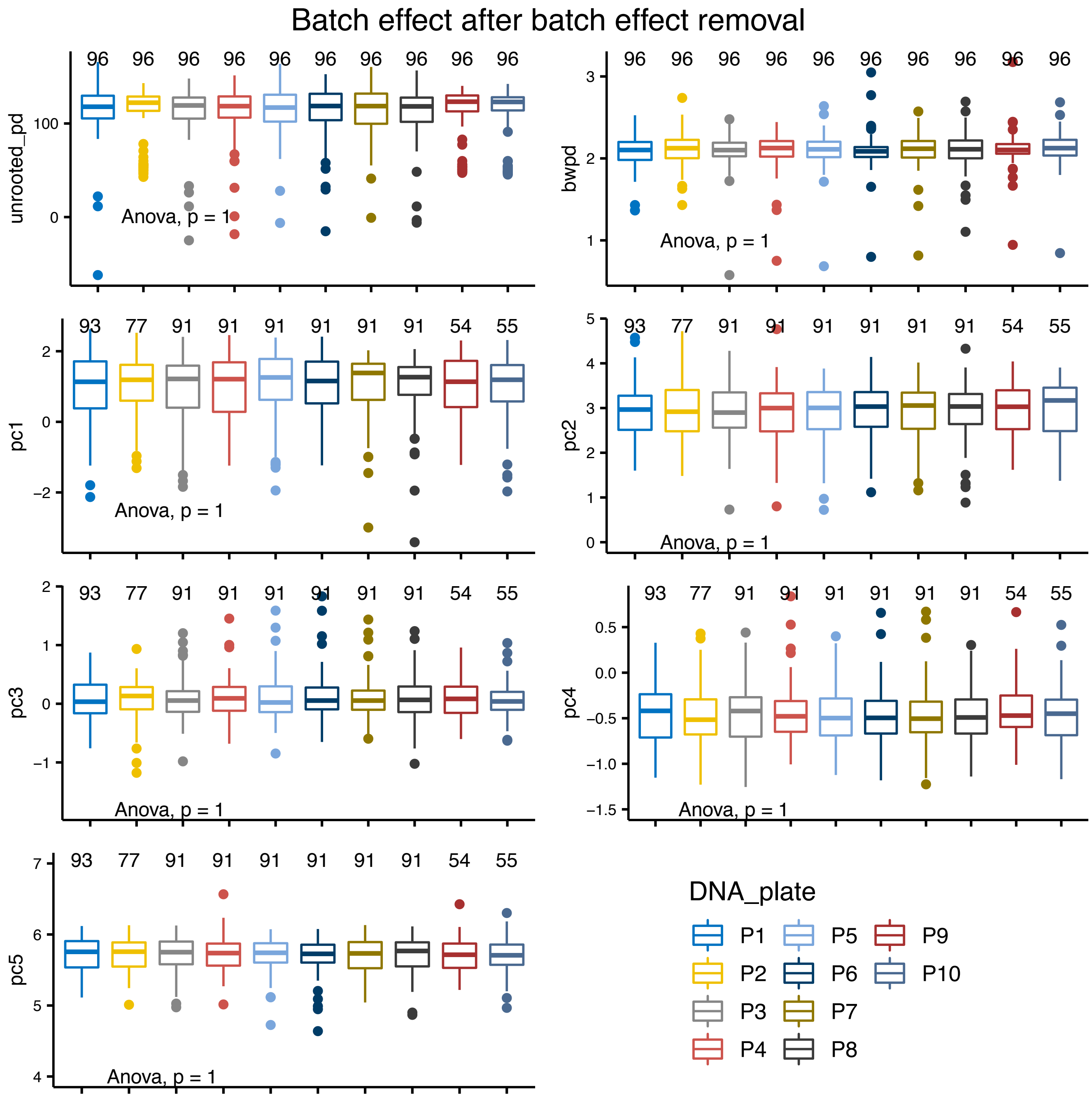

Supplementary Figure 7. Batch effect on alpha and beta diversity after batch effect removal.

Batch effect by alpha (top two plots) and beta diversity (bottom five plots) after batch effect removal. Samples are grouped by DNA extraction plate. The *p* values are derived from multiple comparison analysis with ANOVA, indicating equality of the means. *Post hoc* corrected *p* values for pairwise comparisons are provided in Supplementary File 5.

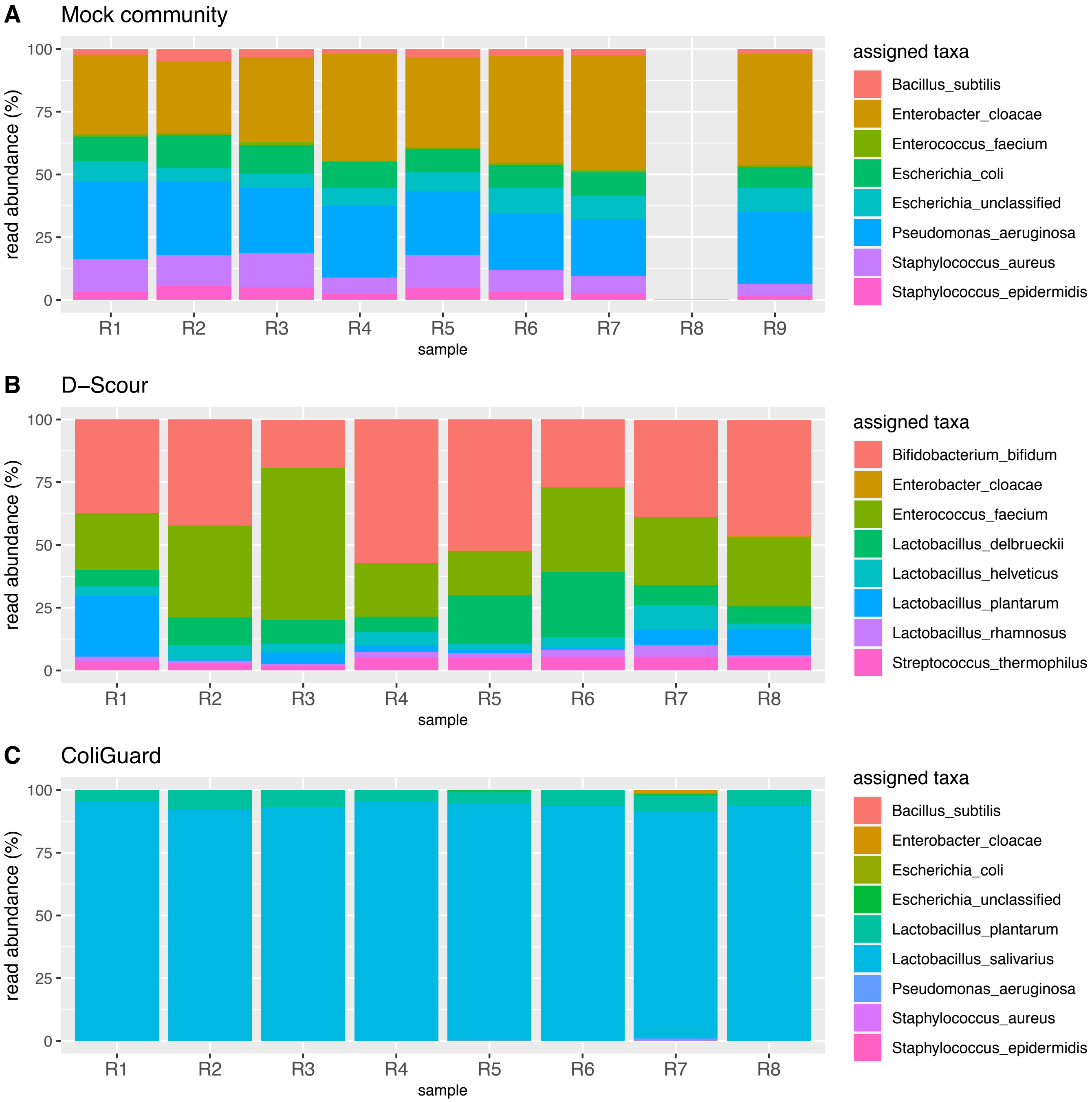

Supplementary Figure 8. Taxonomic assignment of reads from positive control samples.

Taxonomic profile of the positive controls used in this study obtained by mapping the reads against a ~1M bacterial genomes database with MetaPhlAn2. Each stacked barplot represents a technical replicate. Taxa of which reads are present in >0.1% are displayed. **A**. In-house made mock community; **B**. commercially available livestock probiotic D-Scour™ ; **C**. ColiGuard®.

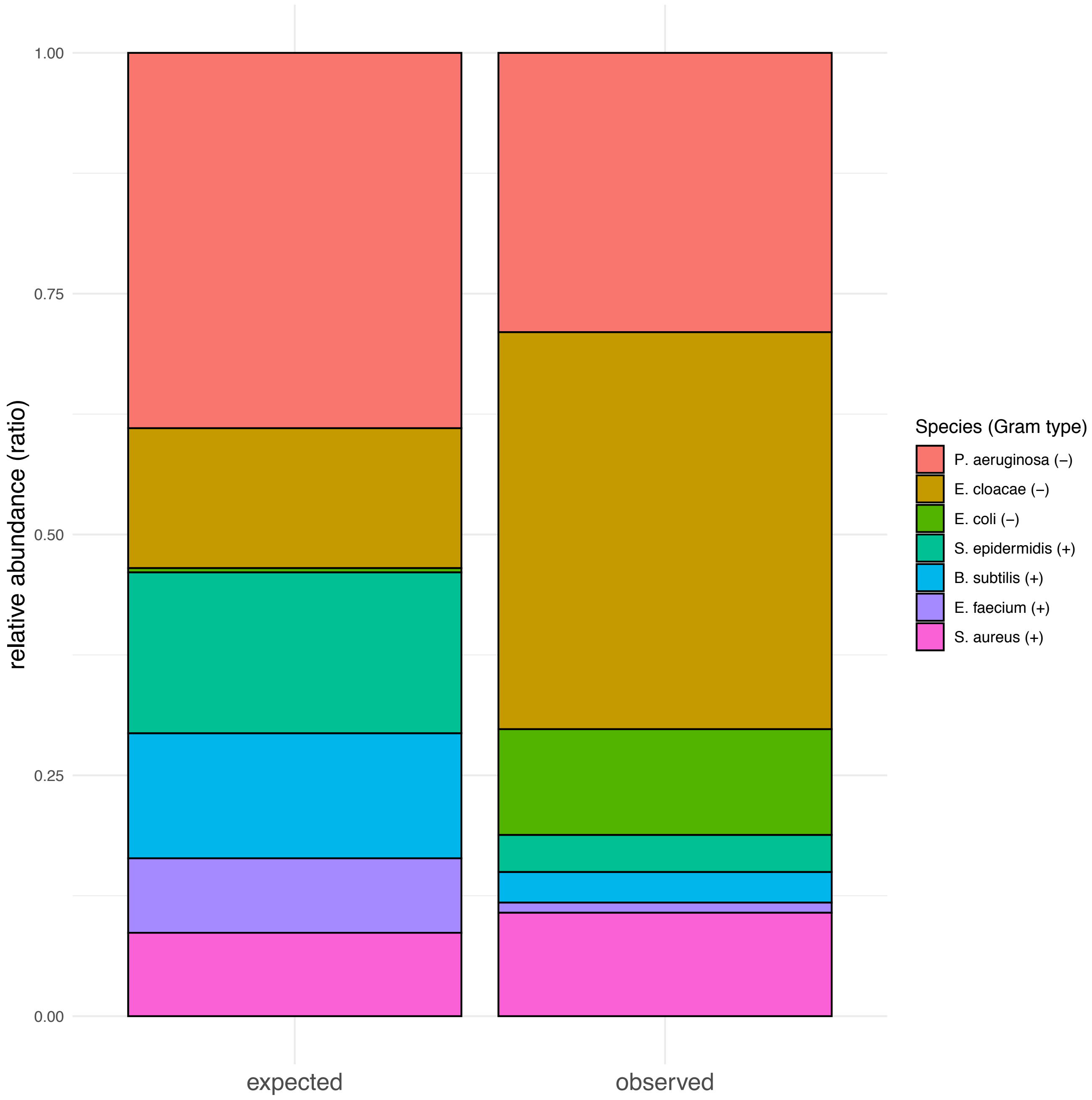

Supplementary Figure 9. Expected and observed relative abundance of mock community members.

Expected relative abundance is derived by multiplying CFU count by genome size. Observed relative abundance is derived by multiplying reads mapping with MetaPhlAn2. A higher observed/expected ratio is observed in two of the three Gram negative species and a lower observed/expected ratio is observed in three of the four Gram negative species.

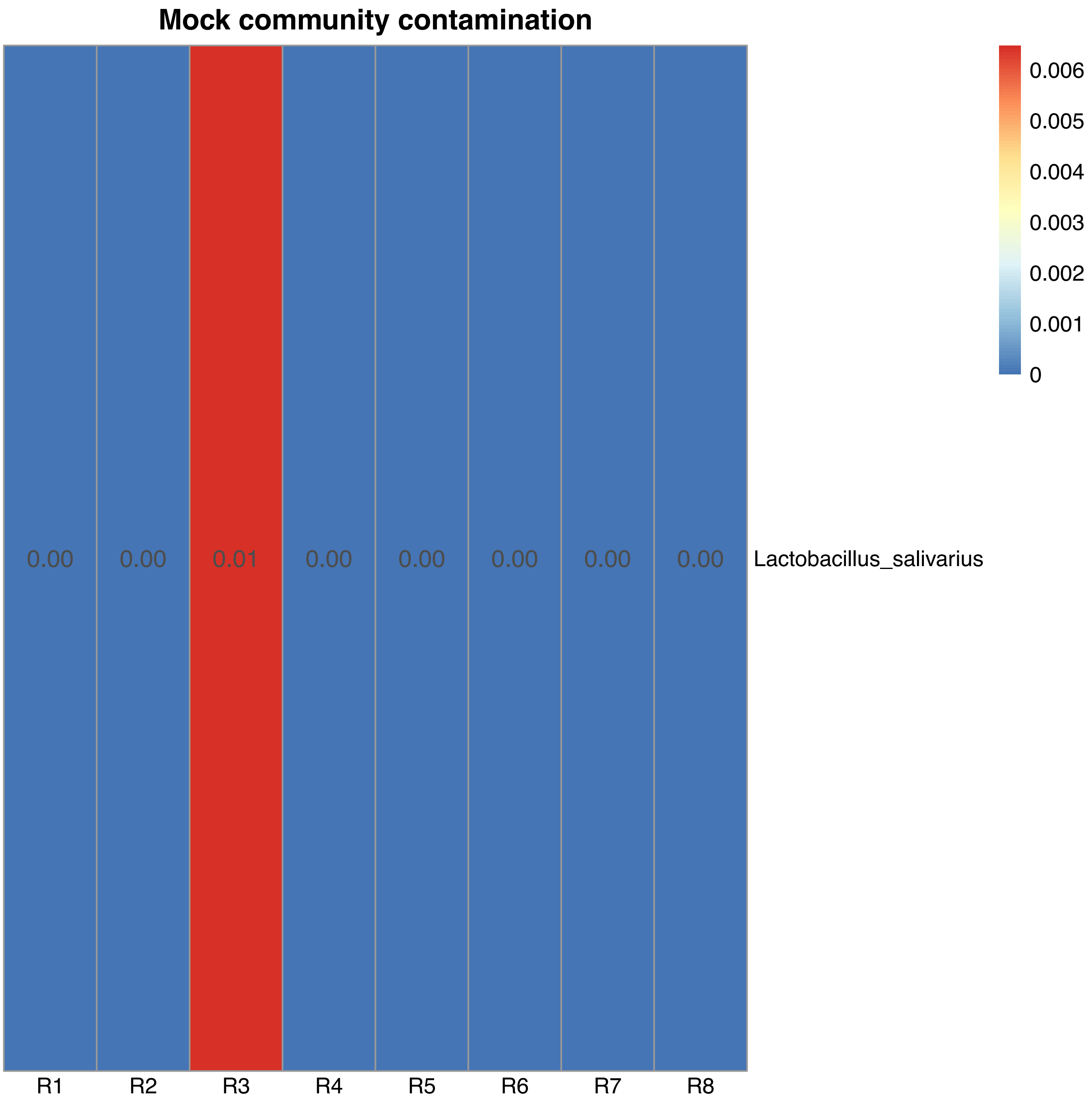

Supplementary Figure 10. Contamination rate in mock community samples.

Heatmap of contaminating species present in the positive control mock community technical replicates. *Lactobacillus salivarius* is found in one replicate at 0.01% of the total reads.

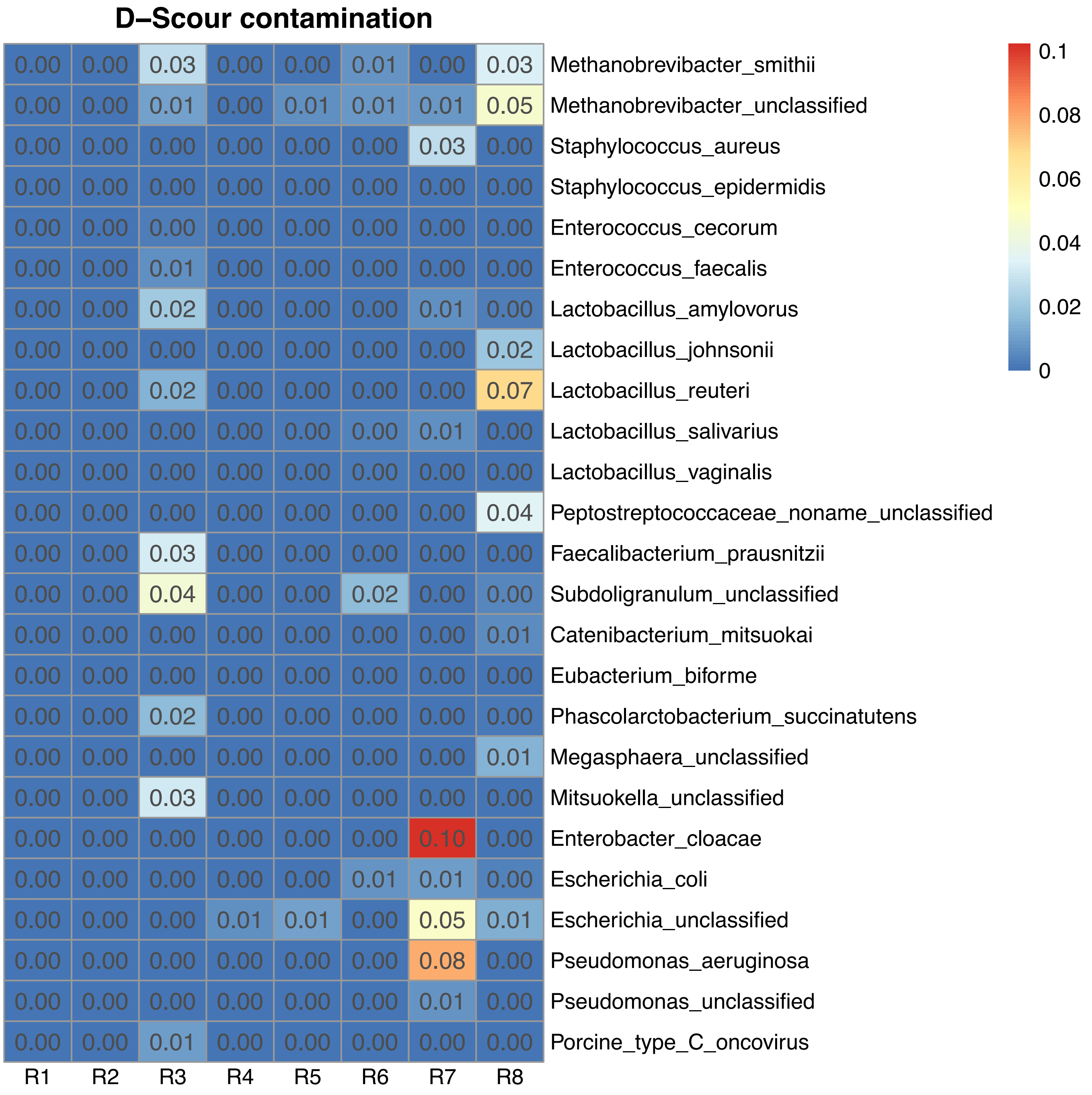

Supplementary Figure 11. Contamination rate in D-Scour™ samples.

Heatmap of contaminating species present in the positive control D-Scour™ technical replicates. The D-Scour™ technical replicates contained 25 contaminants, of which 18 and 7 were identified at the species and at genus level, respectively. Contaminants are majorly present in three technical replicates (R3, R7, R8) and the most frequent contaminant (*Metahobrevibacter*) was present in 5 of the 8 replicates.

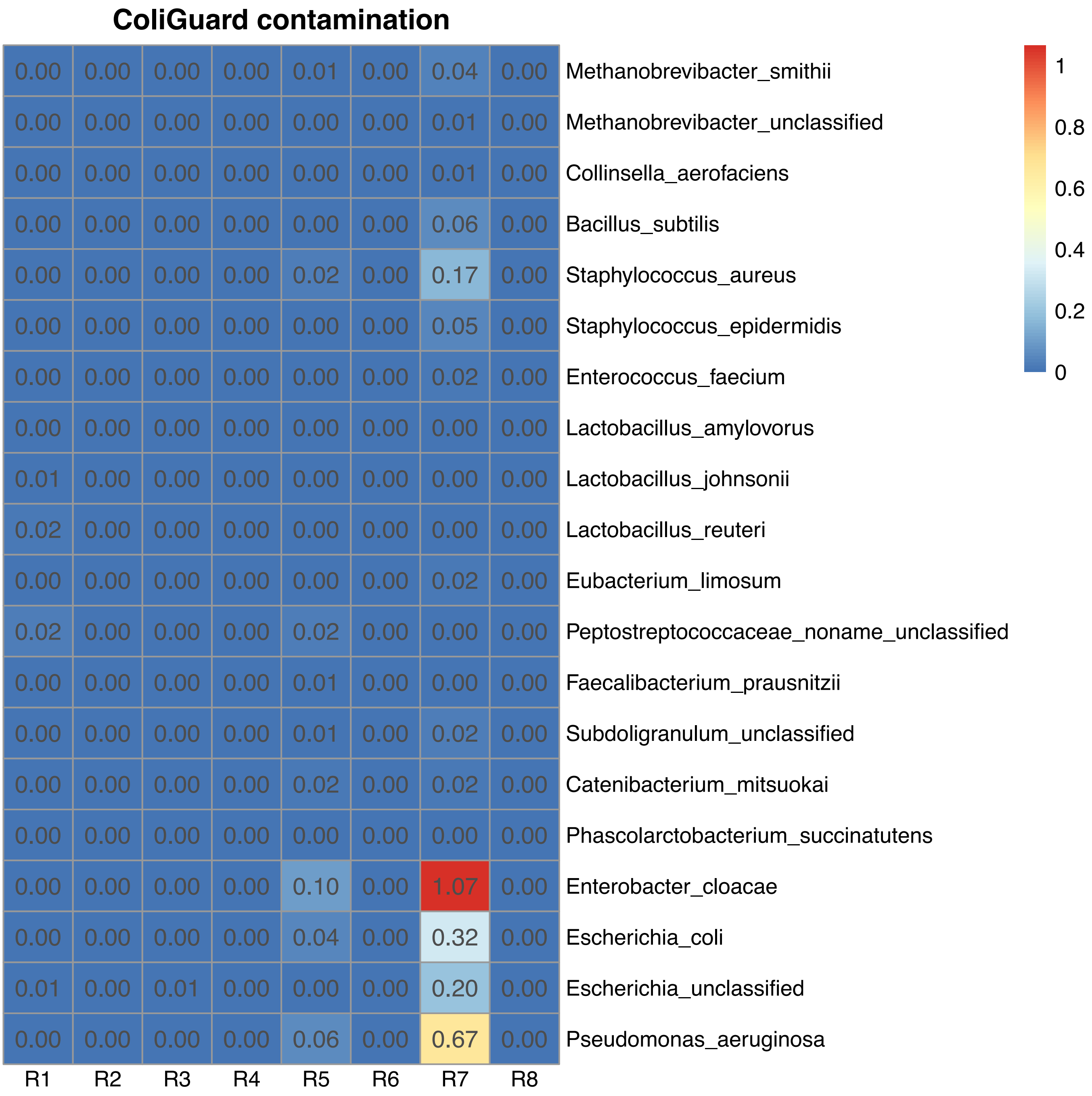

Supplementary Figure 12. Contamination rate in ColiGuard® samples.

Heatmap of contaminating species present in the positive control ColiGuard® technical replicates. ColiGuard® contained 20 contaminants, of which 16 and 4 were identified at the species and at genus level, respectively. Contaminants were present majorly in two technical replicates (R5, R7).

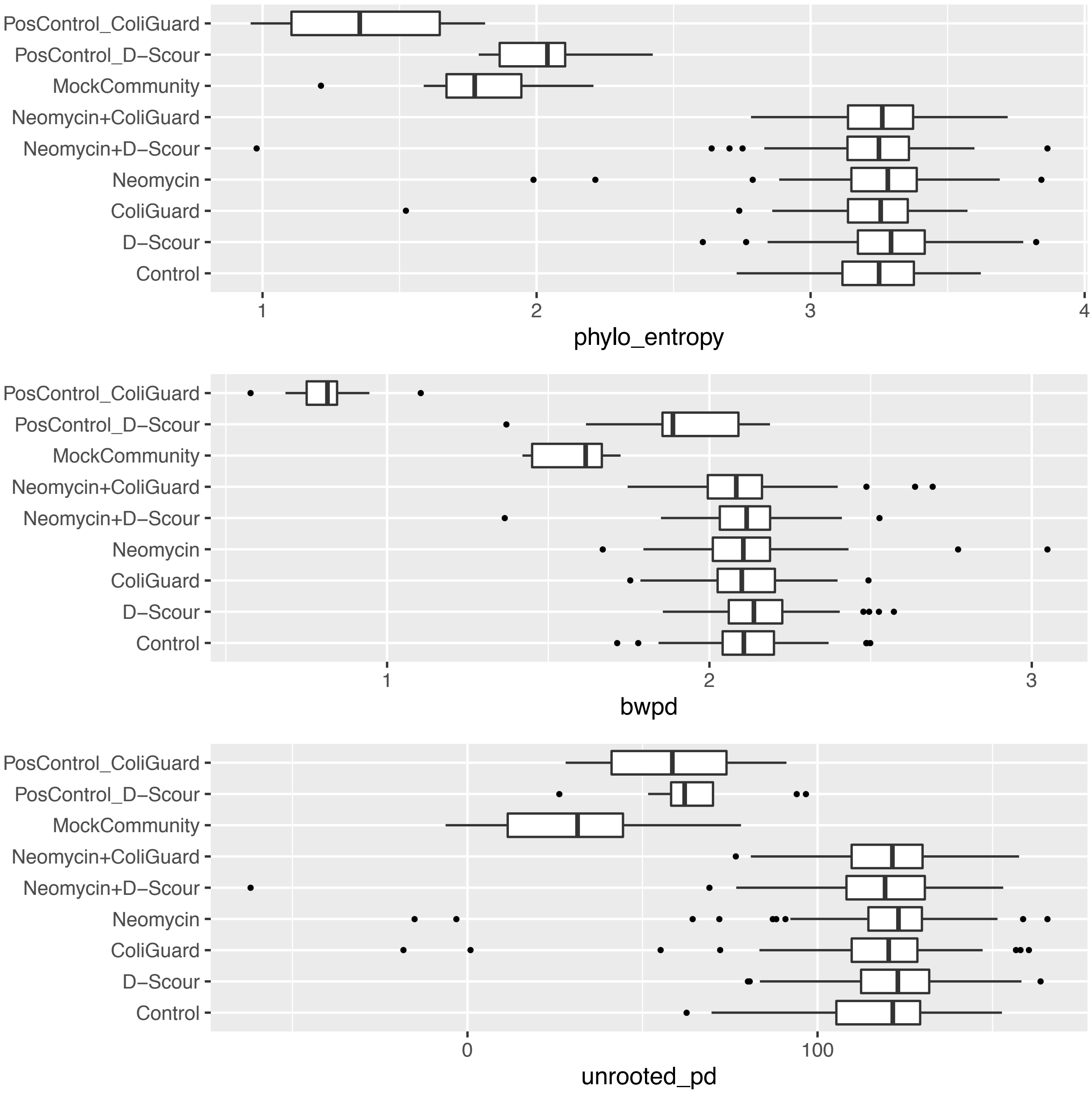

Supplementary Figure 13. Alpha phylogenetic diversity of cohorts.

Alpha phylogenetic diversity per cohort from samples across all time points. Phylogenetic entropy (top); Balance-weighted phylogenetic diversity (BWPD) (mean±SD: Positive control Mock community: 1.58±0.12; Positive control D-Scour™: 1.89±0.26; Positive control ColiGuard®: 0.82±0.15; Control: 2.12±0.14; D-Scour™: 2.14±0.14; ColiGuard®: 2.11±0.13; neomycin: 2.12±0.17; neomycin+D-Scour™: 2.12±0.14; neomycin+ColiGuard®: 2.10±0.15; sows: 2.13±0.14; all piglet cohorts: 2.12±0.14); (middle); Unrooted phylogenetic diversity (Positive control Mock community: 31.53±29.50; Positive control D-Scour™: 64.84±21.30; Positive control ColiGuard®: 58.52±21.70; Control: 117±16.8; D-Scour™: 121±14.7; ColiGuard®: 118±22.3; neomycin: 119±22.6; neomycin+D-Scour™: 116±23.4; neomycin+ColiGuard®: 121±15.8; sows: 125.39±7.56; all piglet cohorts: 118.54±19.70) (bottom).

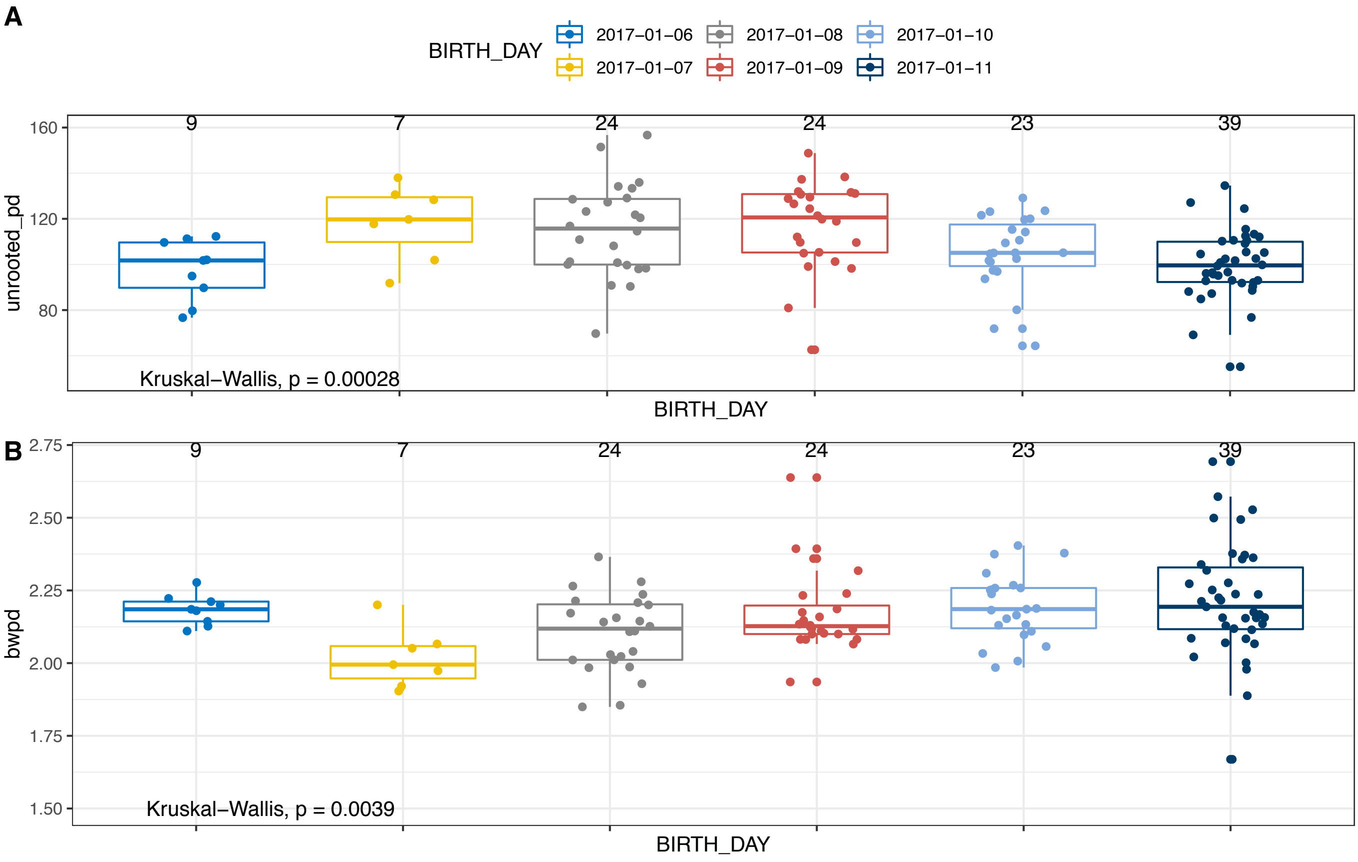

Supplementary Figure 14. Alpha phylogenetic diversity by age group.

Alpha diversity of samples from the start of the trial grouped by the date of birth of the piglet. Unrooted phylogenetic diversity (top) and balance weighted phylogenetic diversity (bottom). *P* values are derived from Kruskal-Wallis analysis of variance. Pairwise comparisons and *post hoc* corrected *p* values are provided in Supplementary File 5.

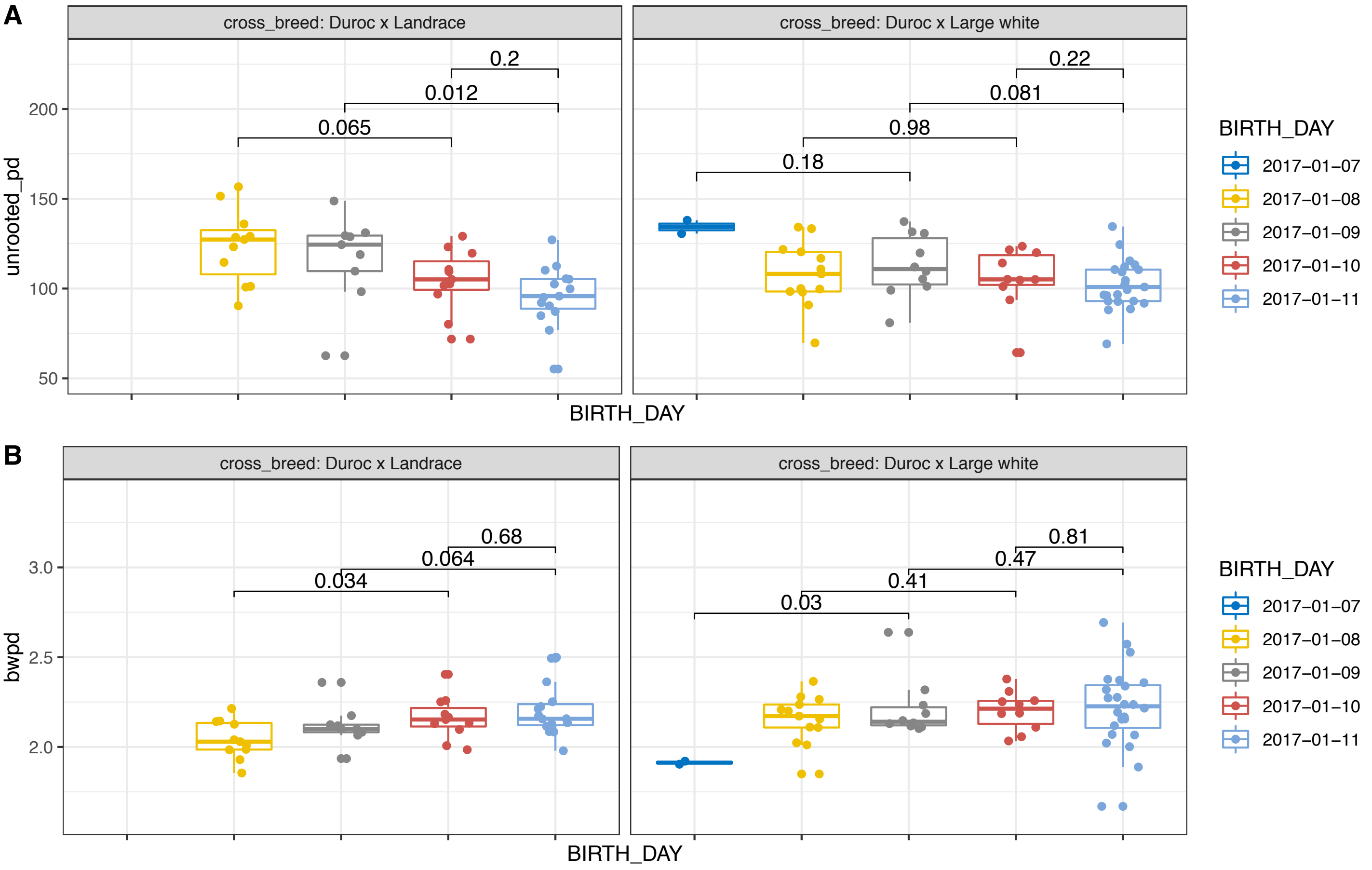

Supplementary Figure 15. Alpha phylogenetic diversity by age breed.

Alpha diversity of samples from the start of the trial grouped by breed and by date of birth. Unrooted phylogenetic diversity (top) and balance weighted phylogenetic diversity (bottom). *P* values are derived from Kruskal-Wallis analysis of variance. Pairwise comparisons and *post hoc* corrected *p* values are provided in Supplementary File 5.

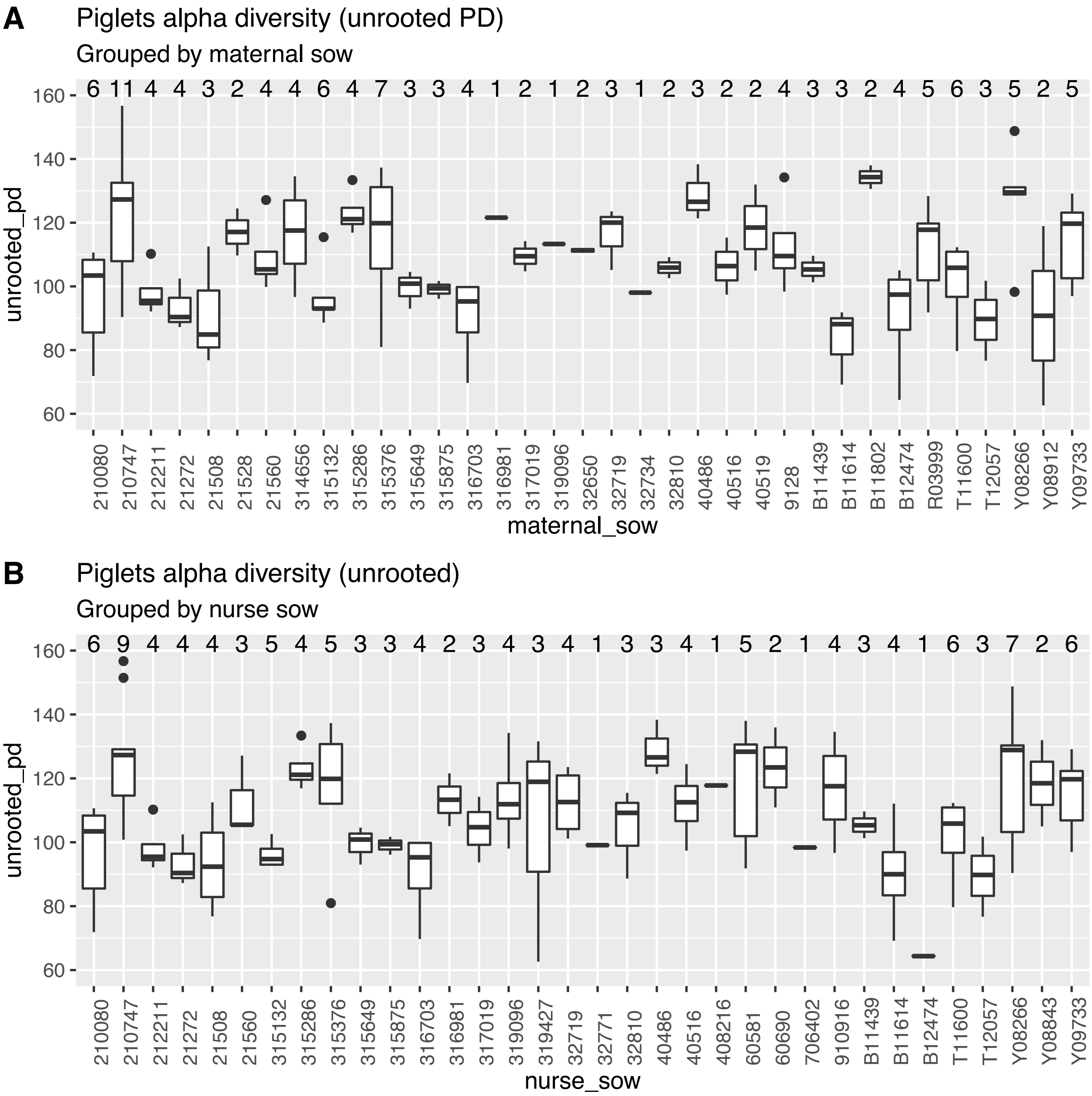

Supplementary Figure 16. Unrooted alpha phylogenetic diversity by maternal and nurse sow.

Alpha phylogenetic diversity (unrooted) of samples from piglets at the start of the trial (one time point -sample per piglet). The grouping is based on the maternal sow (**A**) or nurse sow (**B**). *P* values from multiple groups comparison by maternal sow: Kruskal-Wallis *p* values: 0.0011; BWPD: 0.0049. *P* values from multiple groups comparison by nurse sow: Kruskal-Wallis *p* values: 0.0036; BWPD: 0.0071. *Post-hoc* pairwise comparisons and adjusted *p* values are provided in Supplementary File 5.

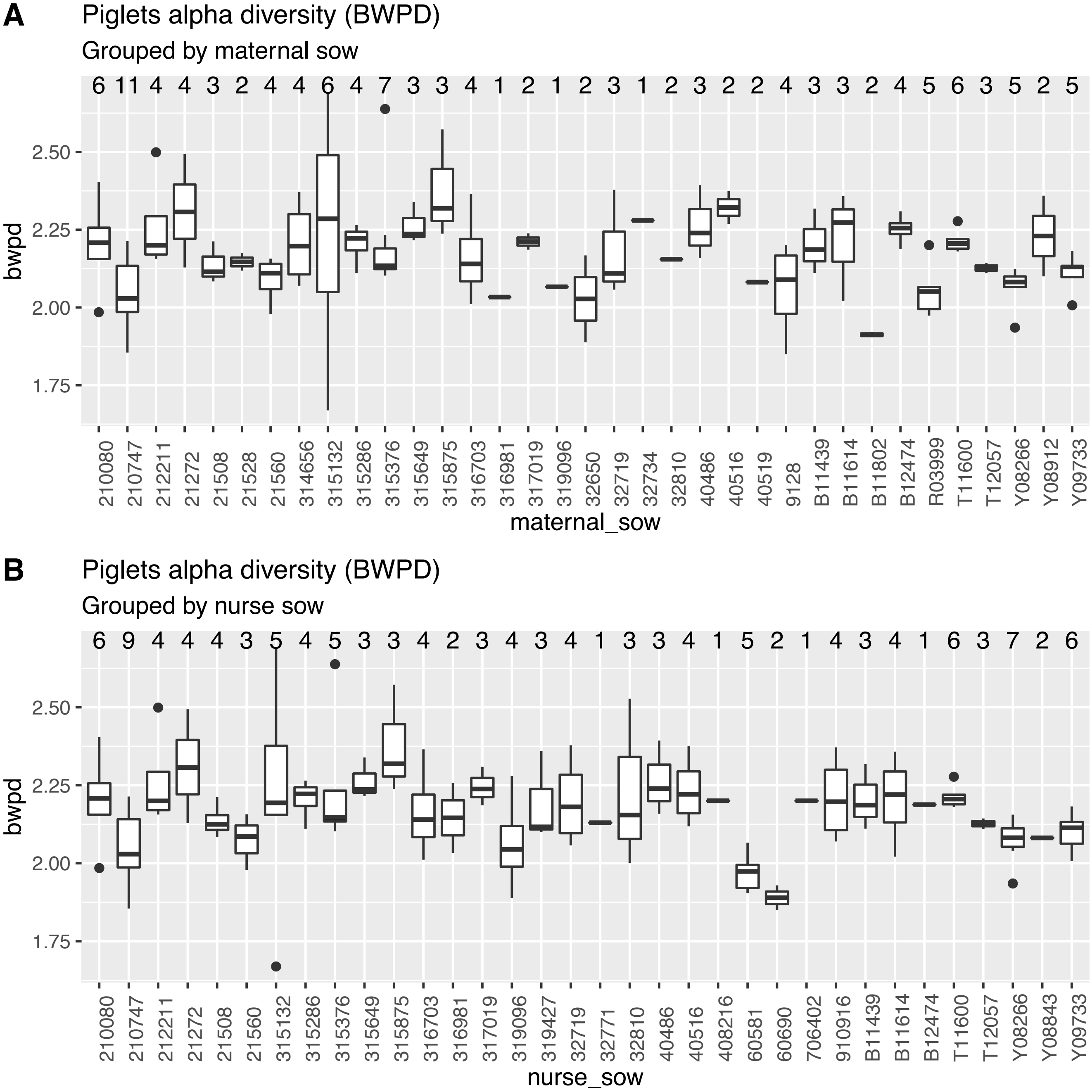

Supplementary Figure 17. Balance weighted alpha phylogenetic diversity by maternal and nurse sow.

Alpha phylogenetic diversity (unrooted) of samples from piglets at the start of the trial (one time point -sample per piglet). The grouping is based on the maternal sow (**A**) or nurse sow (**B**). *P* values from multiple groups comparison by maternal sow: Kruskal-Wallis *p*=0.0049. *P* values from multiple groups comparison by nurse sow: Kruskal-Wallis *p*=0.0071. *Post-hoc* pairwise comparisons and adjusted *p* values are provided in Supplementary File 5.

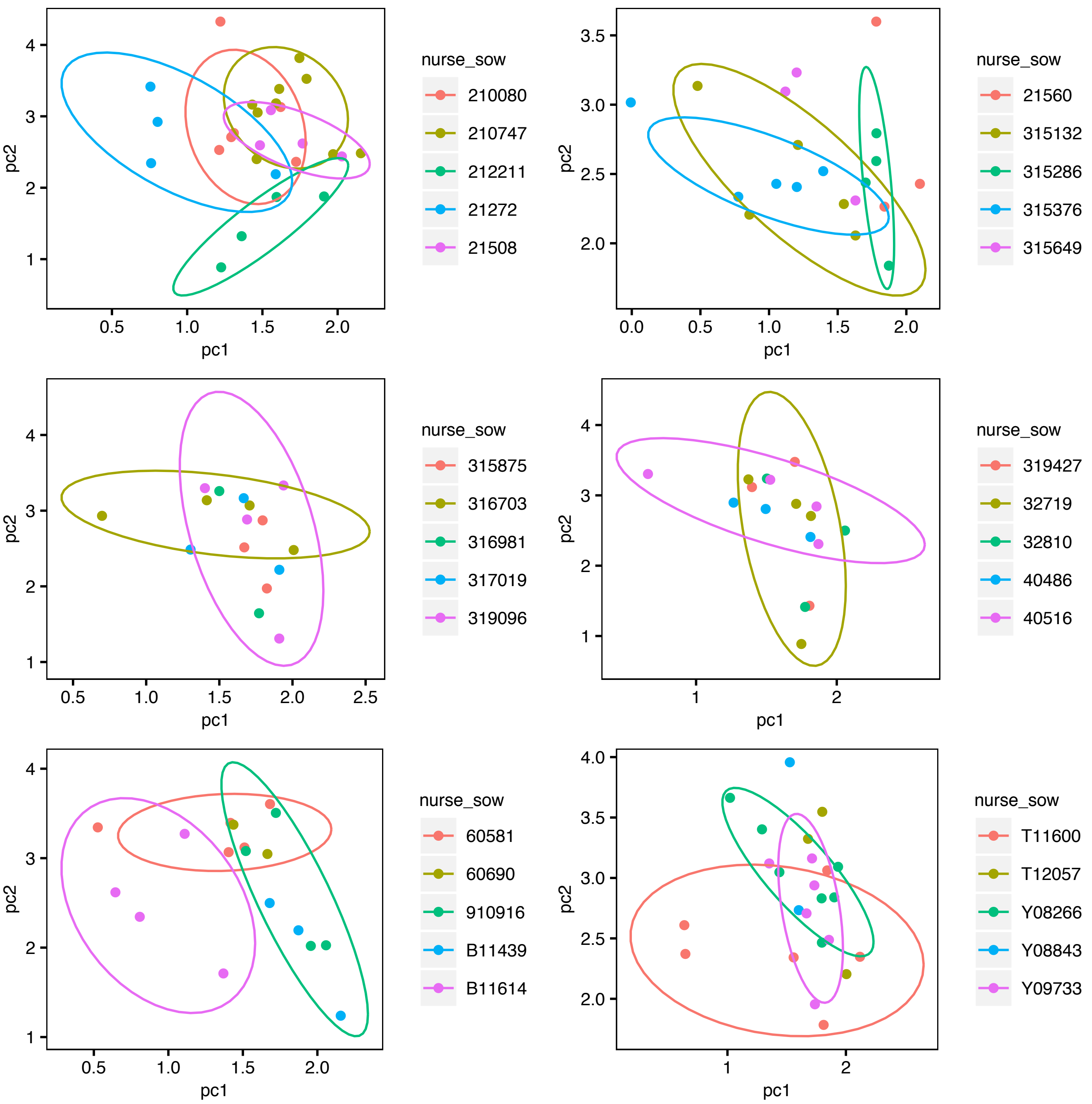

Supplementary Figure 18. Principal component analysis of piglet samples by nurse sow.

First two principal components of beta phylogenetic diversity. Principal component 1 (PC1) and principal component 2 (PC2) explaining 46.99% and 21.68% of the variation, respectively.
Samples from piglets at the start of the trial (one time point -sample per piglet) coloured by nurse sow (n=30). Ellipse is drawn at 0.80 confidence level.

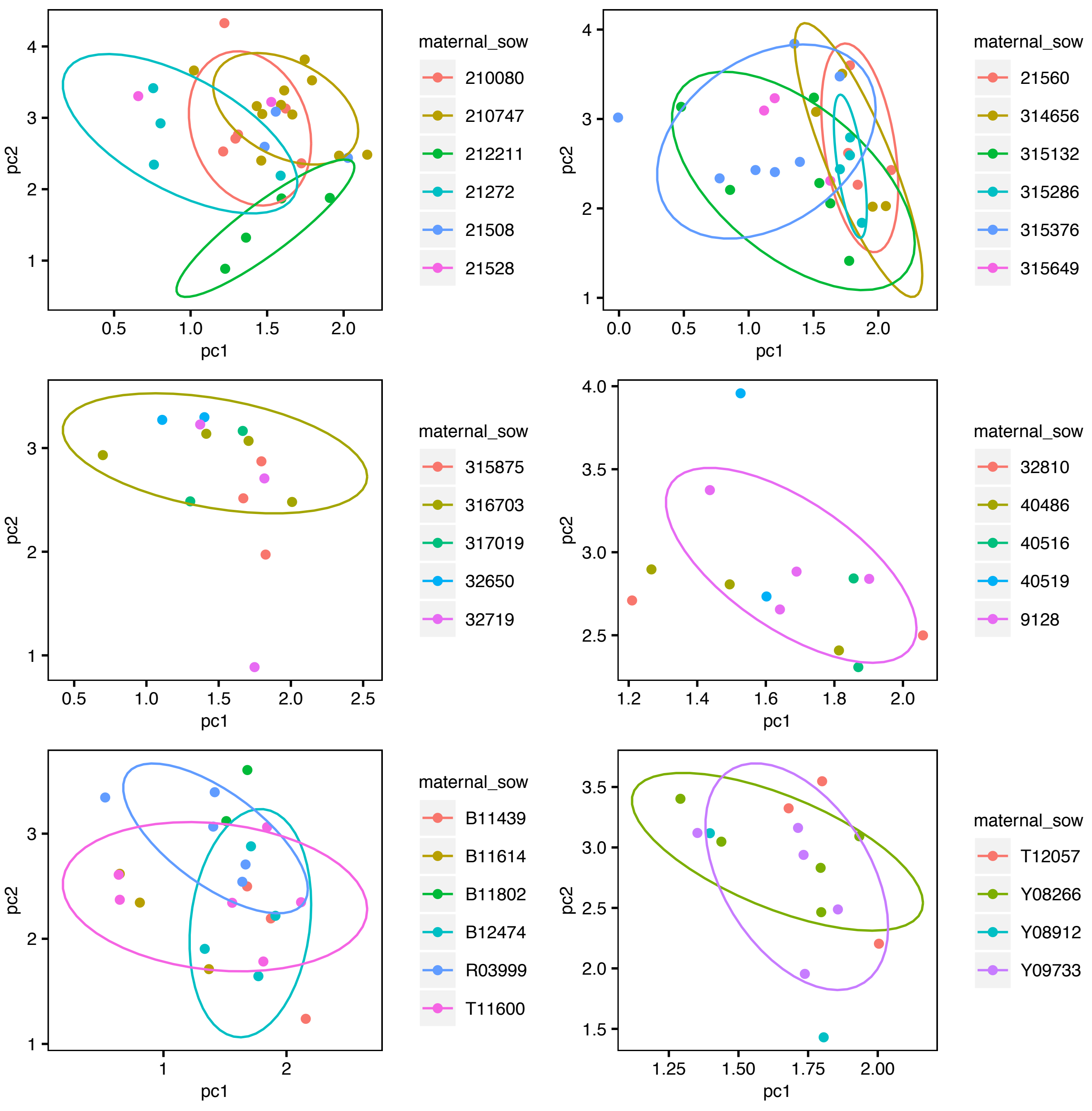

Supplementary Figure 19. Principal component analysis of piglet samples by maternal sow.

First two principal components of beta phylogenetic diversity. Principal component 1 (PC1) and principal component 2 (PC2) explaining 46.99% and 21.68% of the variation, respectively. Samples from piglets at the start of the trial (one time point -sample per piglet) colored by maternal sow (n=32). Ellipse is drawn at 0.80 confidence level.

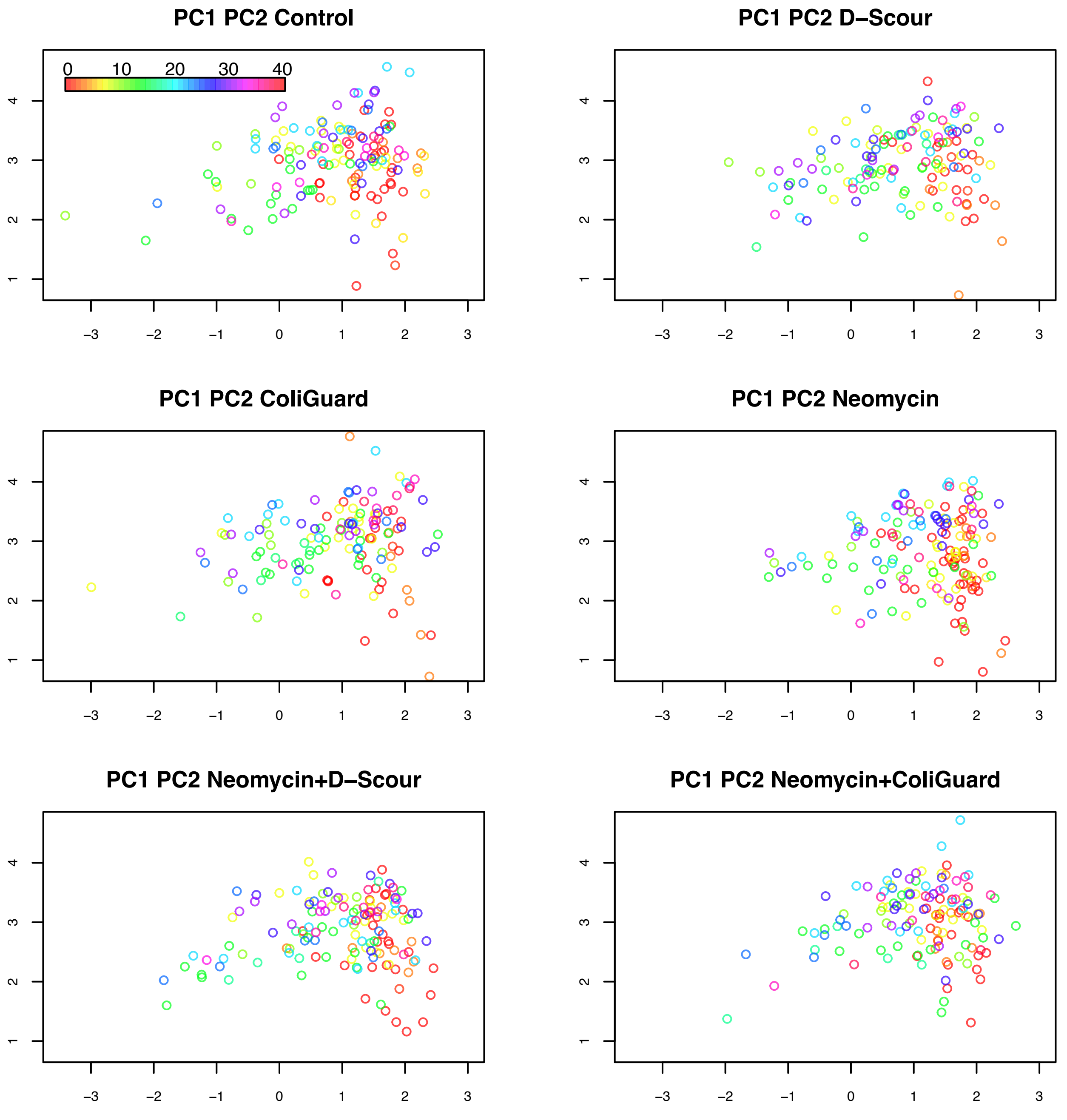

Supplementary Figure 20. Time effect on beta diversity per cohort.

Edge principal component analysis of all samples coloured by sample collection time across the trial (day 0: red; day 10: yellow; day 20: green; day 30: light blue; day 40: dark blue) where principal component 1 (PC1) explains 46.99% of the variation and principal component 2 (PC2) explains 21.68% of the variation. Each plot displays all samples within each cohort.

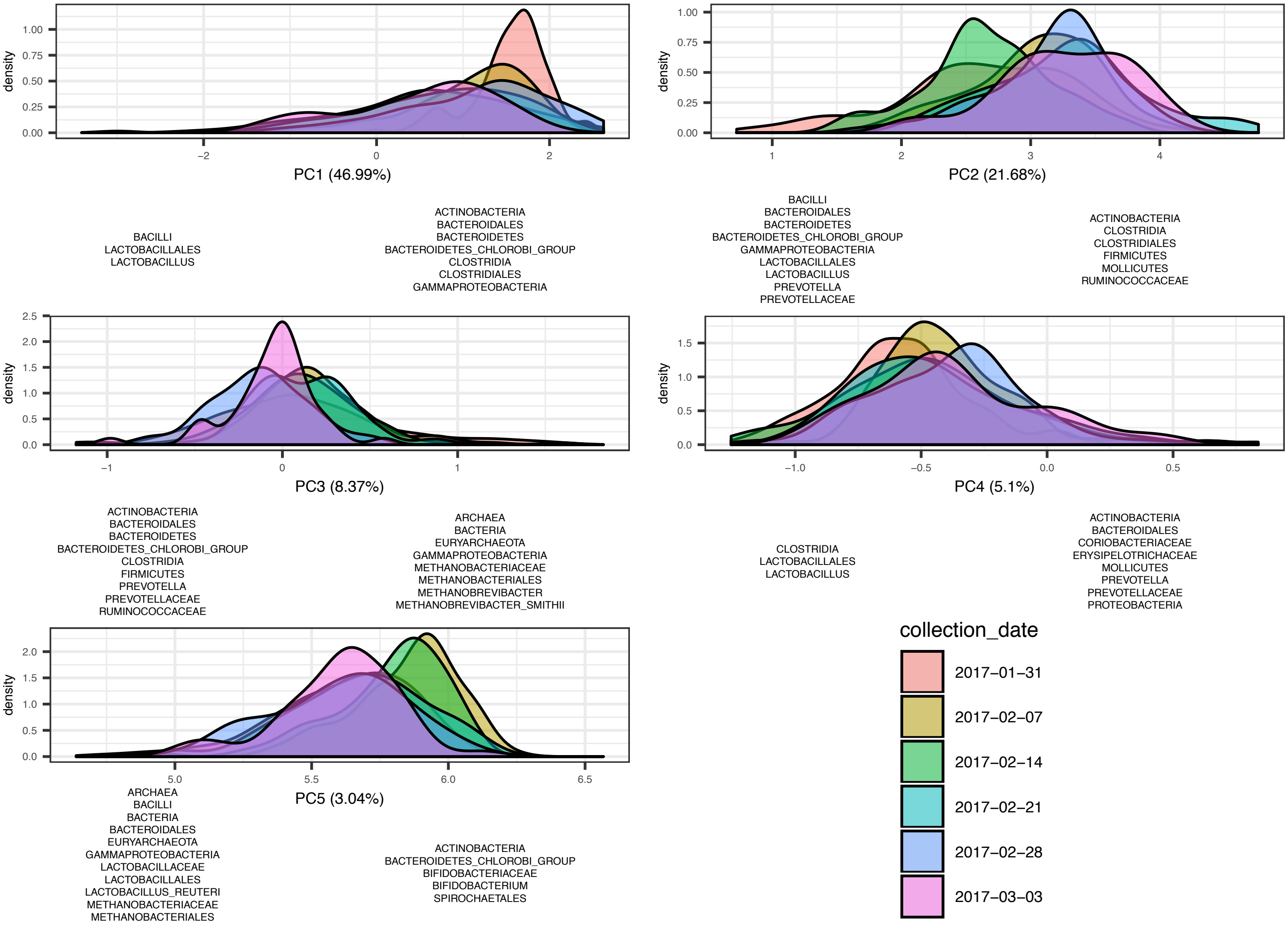

Supplementary Figure 21. Time effect on beta diversity.

Each plot represents the samples distribution along one of the five principal components across time. Most variation is explained by PC1 (46.99%), followed by PC2 (21.68%), PC3 (8.37%), PC4 (5.1%) and PC5 (3.04%). Distribution of the samples on either side of a plot (left *versus* right) reflects the taxa that were found to explain the variation. The distributions are colour coded by time point during the trial.

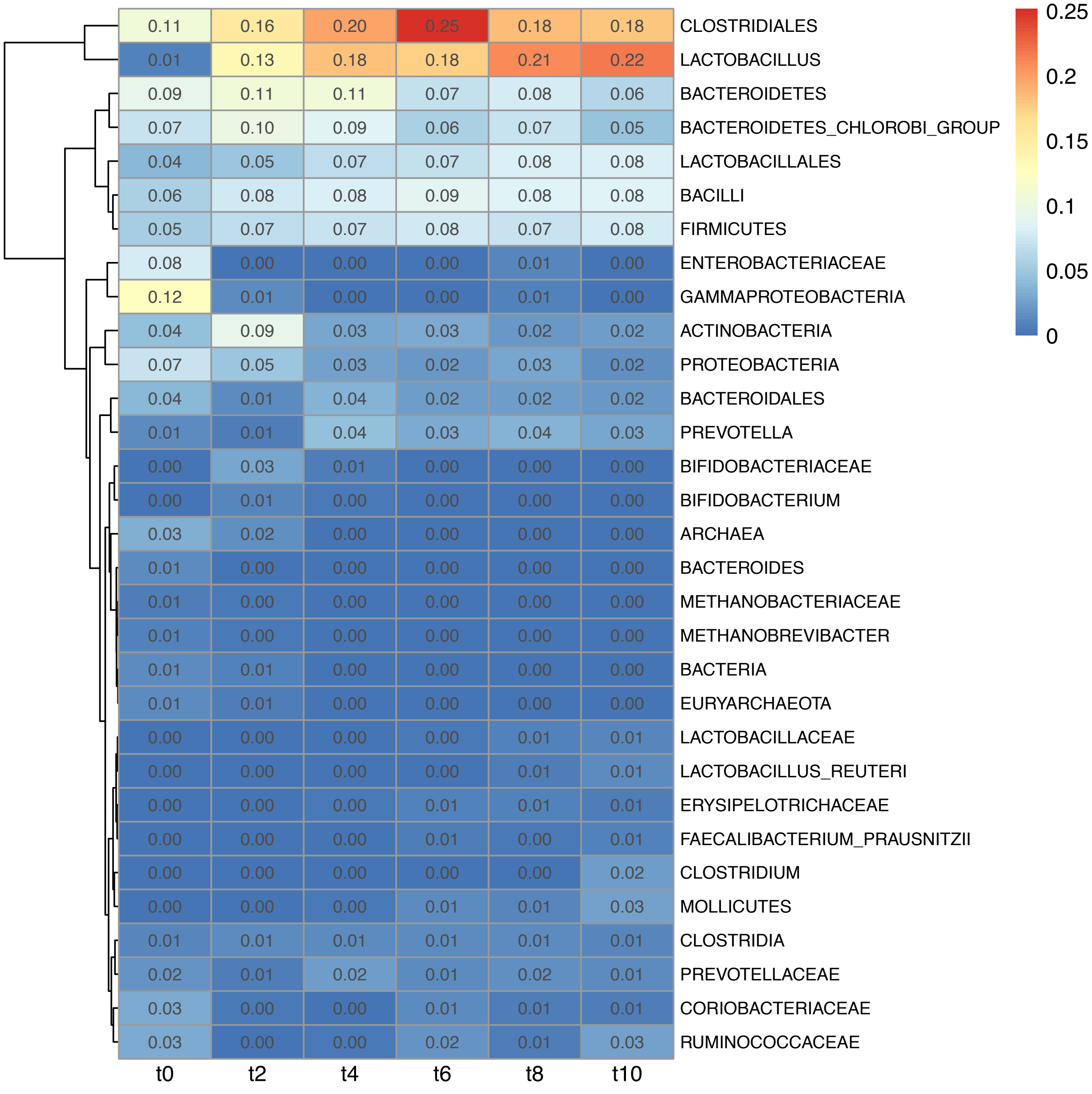

Supplementary Figure 22. Taxa displaying the highest variation in beta diversity across time.

Heatmap of taxa explaining the community composition of samples from separate time points of the trial (1 week interval between time points) derived from edge principal component analysis. Intensity is derived from branch width by the percentage of variability explained by the principal components.

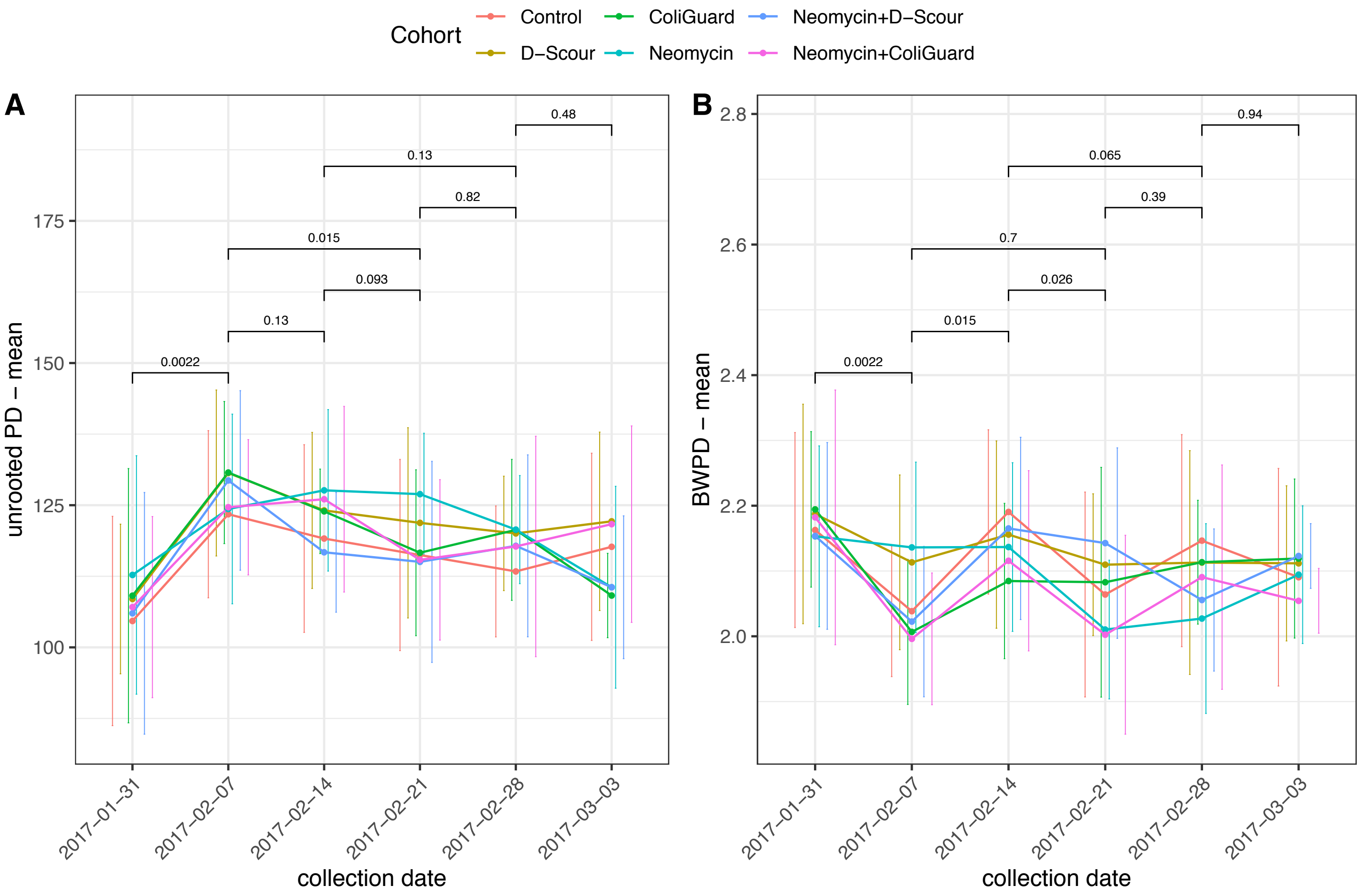

Supplementary Figure 23. Time trend of alpha diversity by cohort.

Unrooted PD (**A**) and BWPD (**B**) describe richness and evenness, respectively, of alpha phylogenetic diversity for all samples across time, grouped and colour coded by cohort. The *p* values derived from pairwise comparisons of time points within each of the cohorts. *P* values and *post-hoc* corrected *p* values of time points comparisons for each separate cohort are provided in Supplementary File 5.

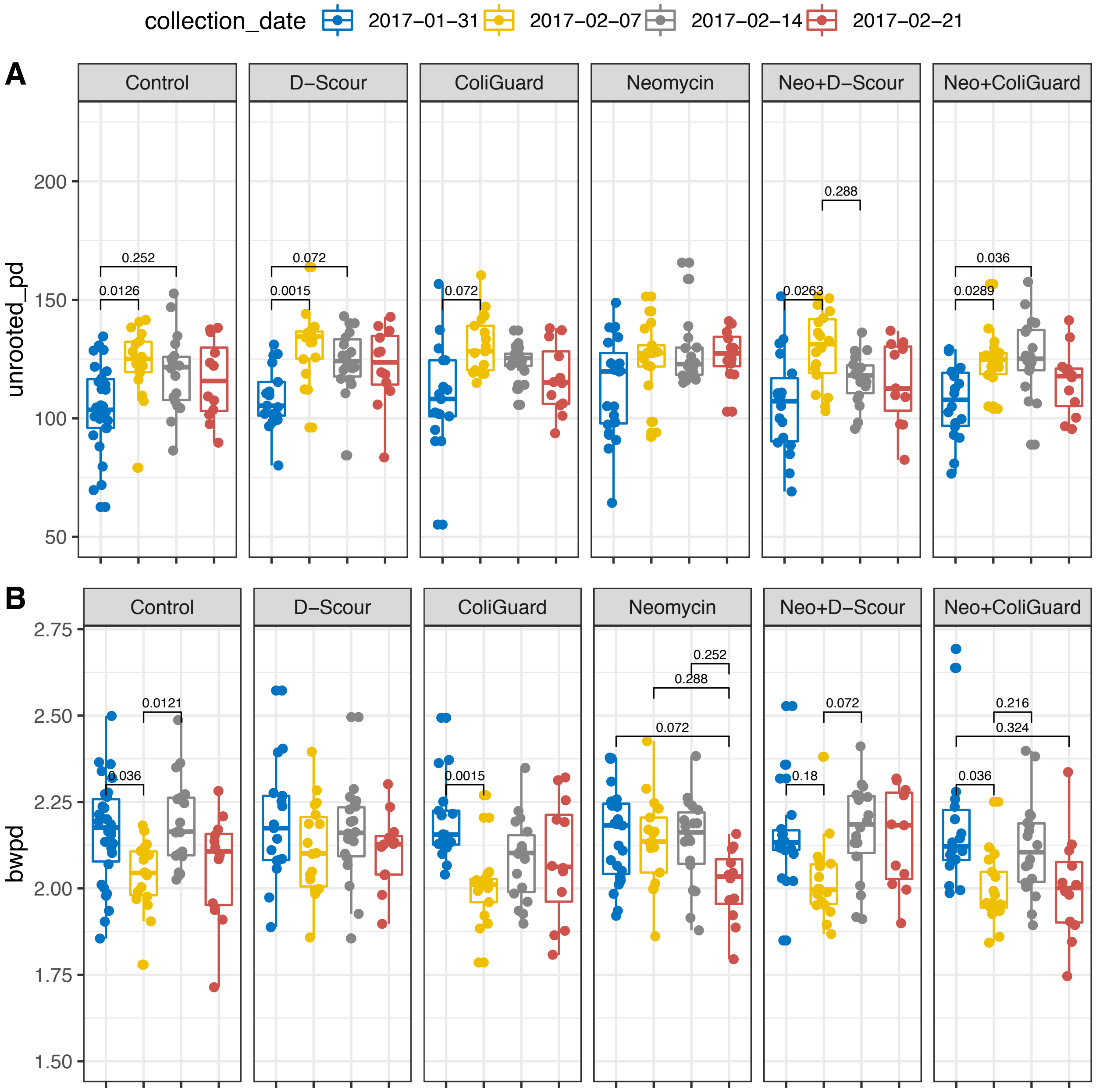

Supplementary Figure 24 Alpha diversity by cohort across four time points.

Alpha phylogenetic diversity for each cohort for a subset of 4 time points. *P* values are derived from pairwise comparisons of the means of each time point of (**A**) unrooted phylogenetic diversity and (**B**) balance weighted phylogenetic diversity. Bonferroni correction of *p* values is applied. More time points were tested and significance was determined; reported in Supplementary File 5.

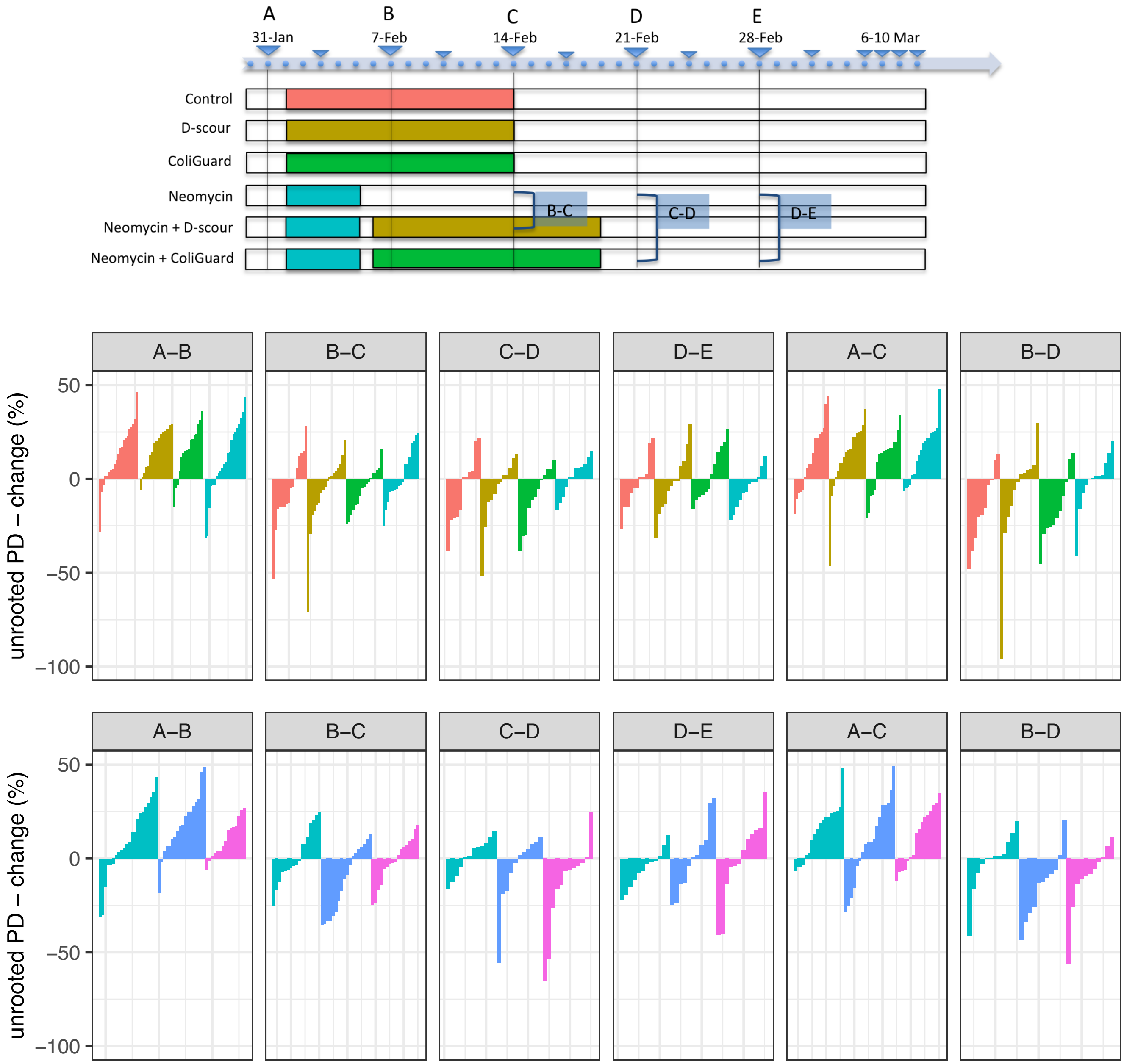

Supplementary Figure 25. Deltas of unrooted alpha phylogenetic diversity between time points.

Change of alpha unrooted phylogenetic diversity in samples between time points across the trial. On the y-axis of plots the percentage difference in unrooted PD between time points per cohort. Letters on the top left of each plot indicate the time points compared with one week interval (A-B, B-C, C-D, D-E) and two weeks interval (A-C, B-D). Pairwise t-test comparisons between cohorts were computed. Brackets in the timeline (top) indicate a significant *p* value ($p<0.05$) between cohorts (1-week interval); bold brackets indicate a significant *p* value ($p<0.05$) between cohorts (2-weeks interval). * indicate significance reached when applying Benjamini-Hochberg *post-hoc* correction.

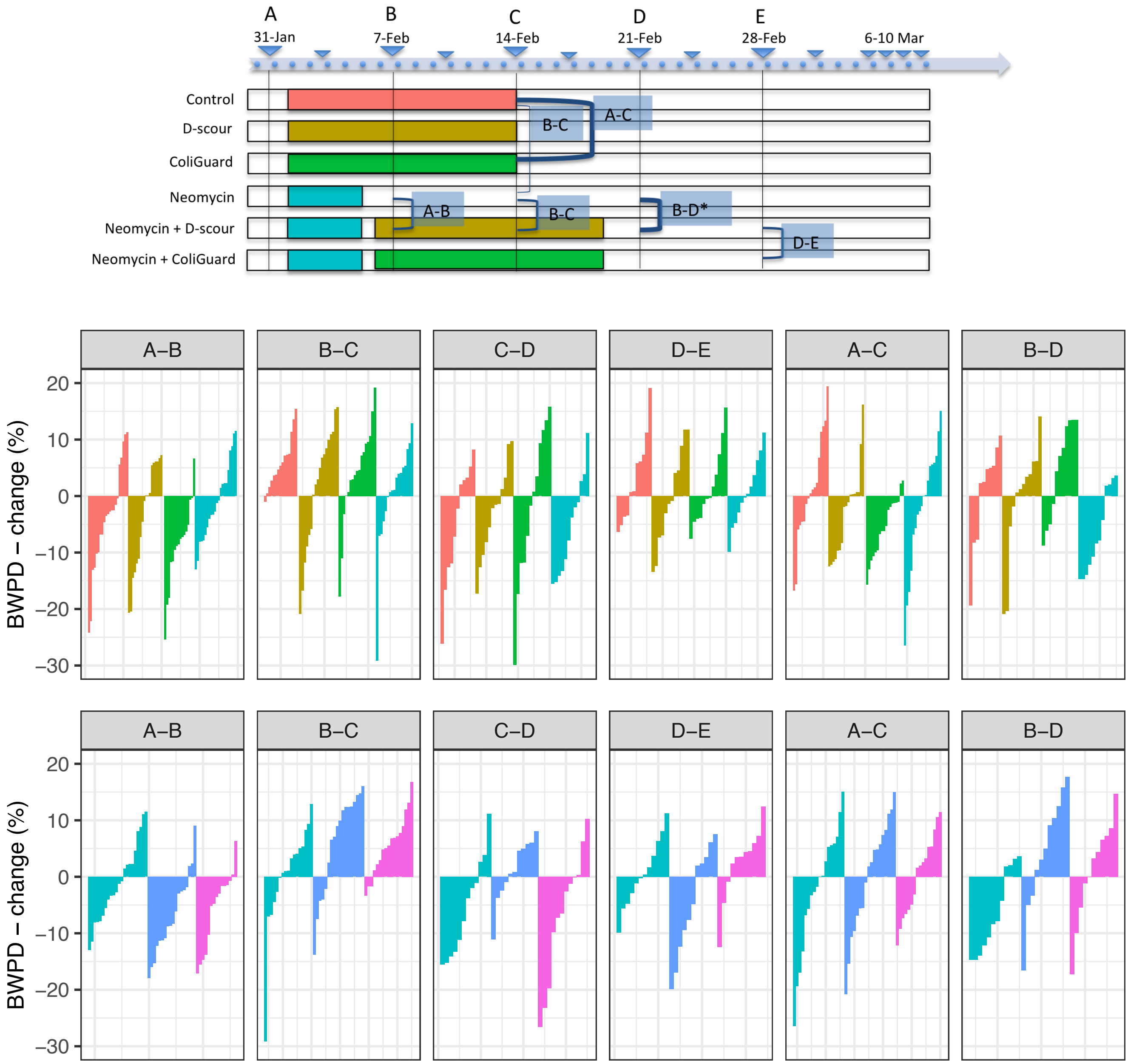

Supplementary Figure 26. Deltas of balance weighted alpha phylogenetic diversity between time points.

Change of alpha balance weighted phylogenetic diversity in samples between time points across the trial. On the y-axis of plots the percentage difference in balance weighted phylogenetic diversity between time points per cohort. Letters on the top left of each plot indicate the time points compared with one week interval (A-B, B-C, C-D, D-E) and two weeks interval (A-C, B-D). Pairwise t-test comparisons between cohorts were computed. Brackets in the timeline (top) indicate a significant *p* value ($p<0.05$) between cohorts (1-week interval); bold brackets indicate a significant *p* value ($p<0.05$) between cohorts (2-weeks interval). * indicate significance reached when applying Benjamini-Hochberg *post-hoc* correction.

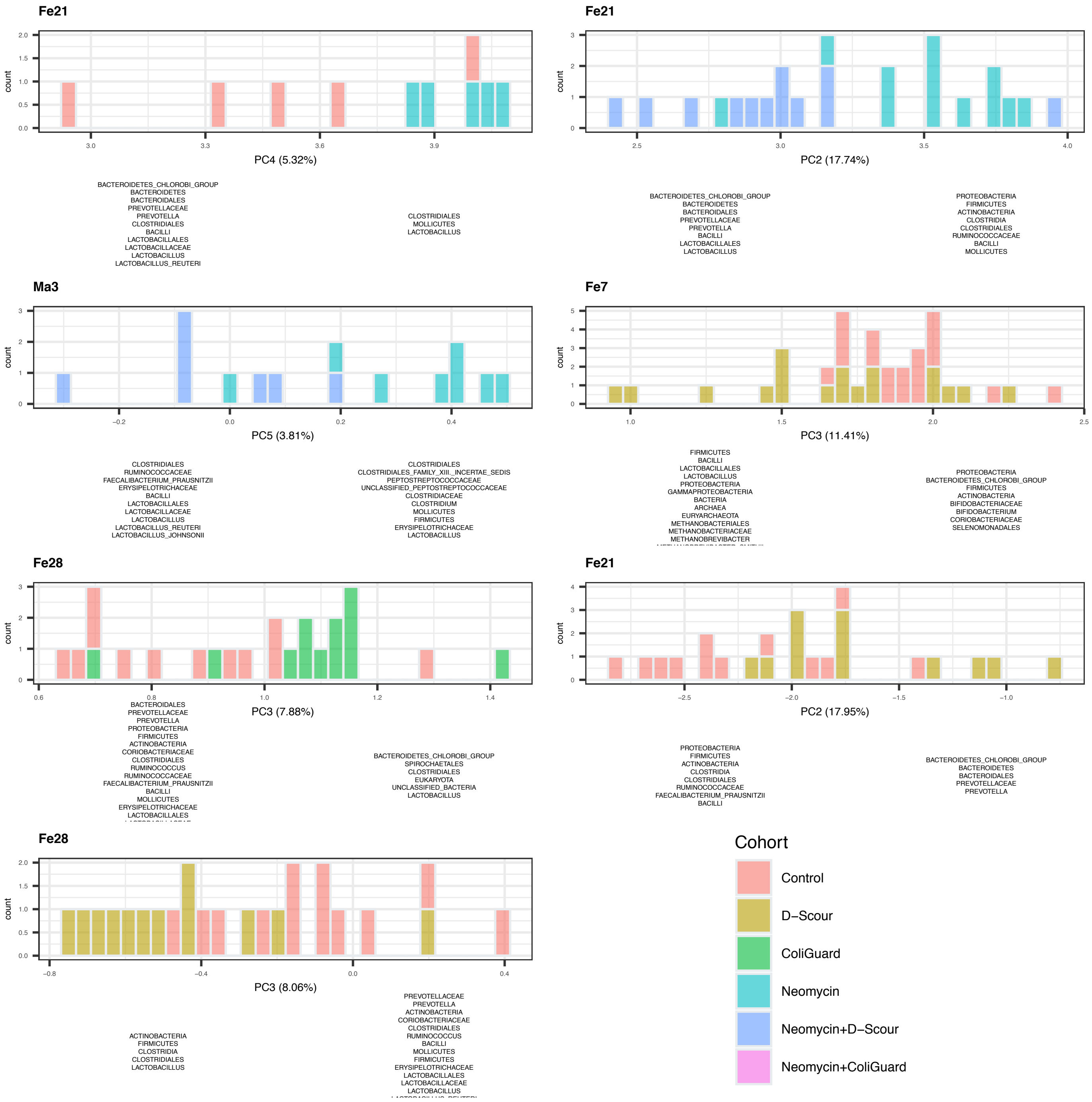

Supplementary Figure 27. Significant differences in beta diversity between cohorts at specific time points.

Significance was determined by comparing groups by pairwise t-test and the resulting *p* values where adjusted with the Bonferroni method. Significance values are provided in Supplementary File 5. The x-axes represent the principal component. As plots are derived from distinct guppy runs, each principal component explains variation to a different extent (percentage specified in parentheses). The number of samples is specified on the y-axis. Distribution of the samples on either side of a plot (left *versus* right) reflects the taxa that were found to explain the variation. Distributions are colour coded by cohort.

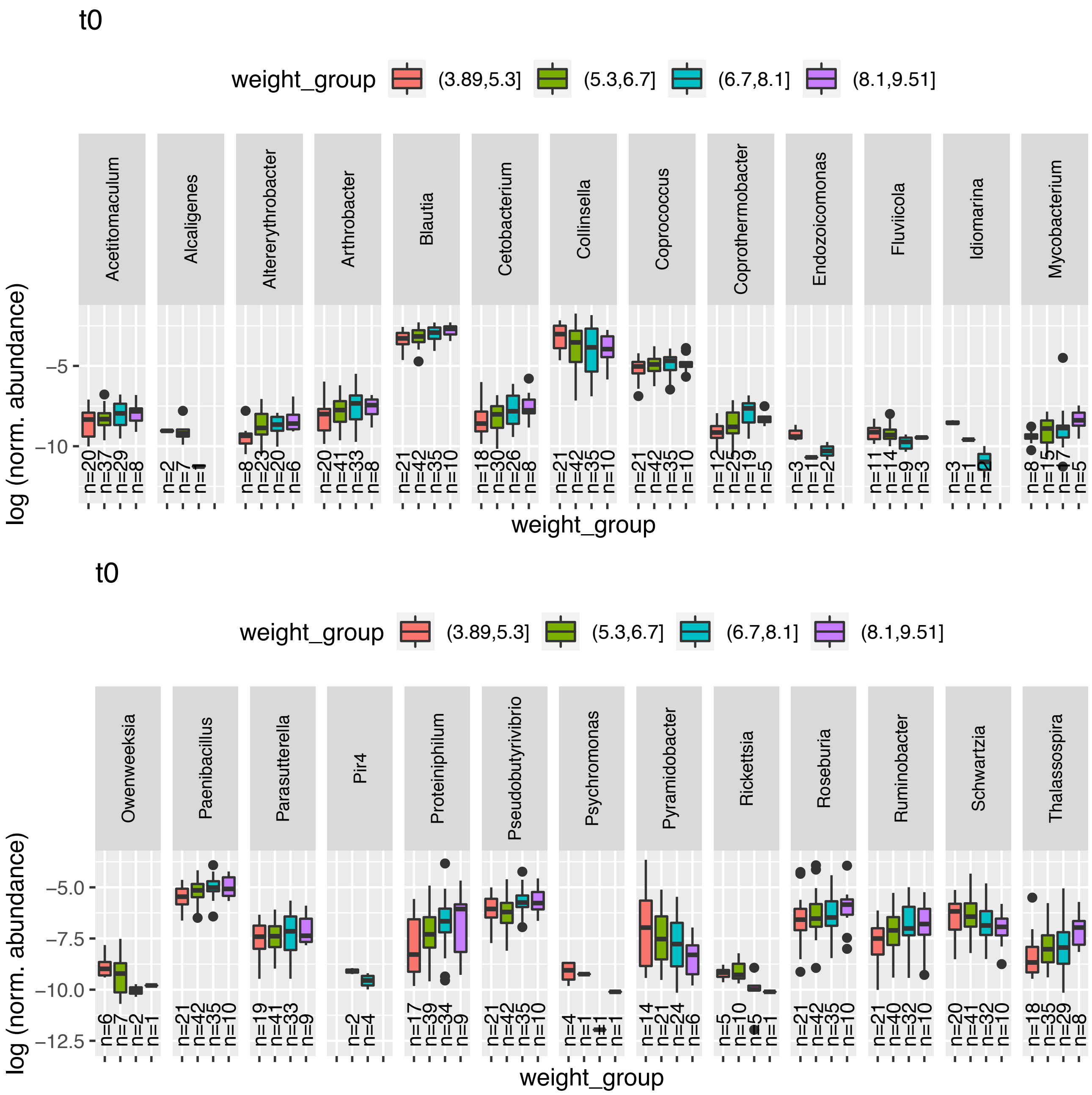

Supplementary Figure 28. Correlation of weight with taxonomy at time point 0.

16S rRNA reads were extracted with SortMeRNA. Abundance was estimated and correlation with weight was assessed with the Spearman method. Significance values are provided in Supplementary File 5. Plotted are the correlations with *p* value < 0.05. For visualization purposes, subjects are binned into weight groups (x-axis). Bins are formed with subjects whose weight falls below the first quartile (red), above the first quartile (green), below the third quartile (blue), above the third quartile (purple). At the bottom of each whisker plot, the samples size for each bin is provided.

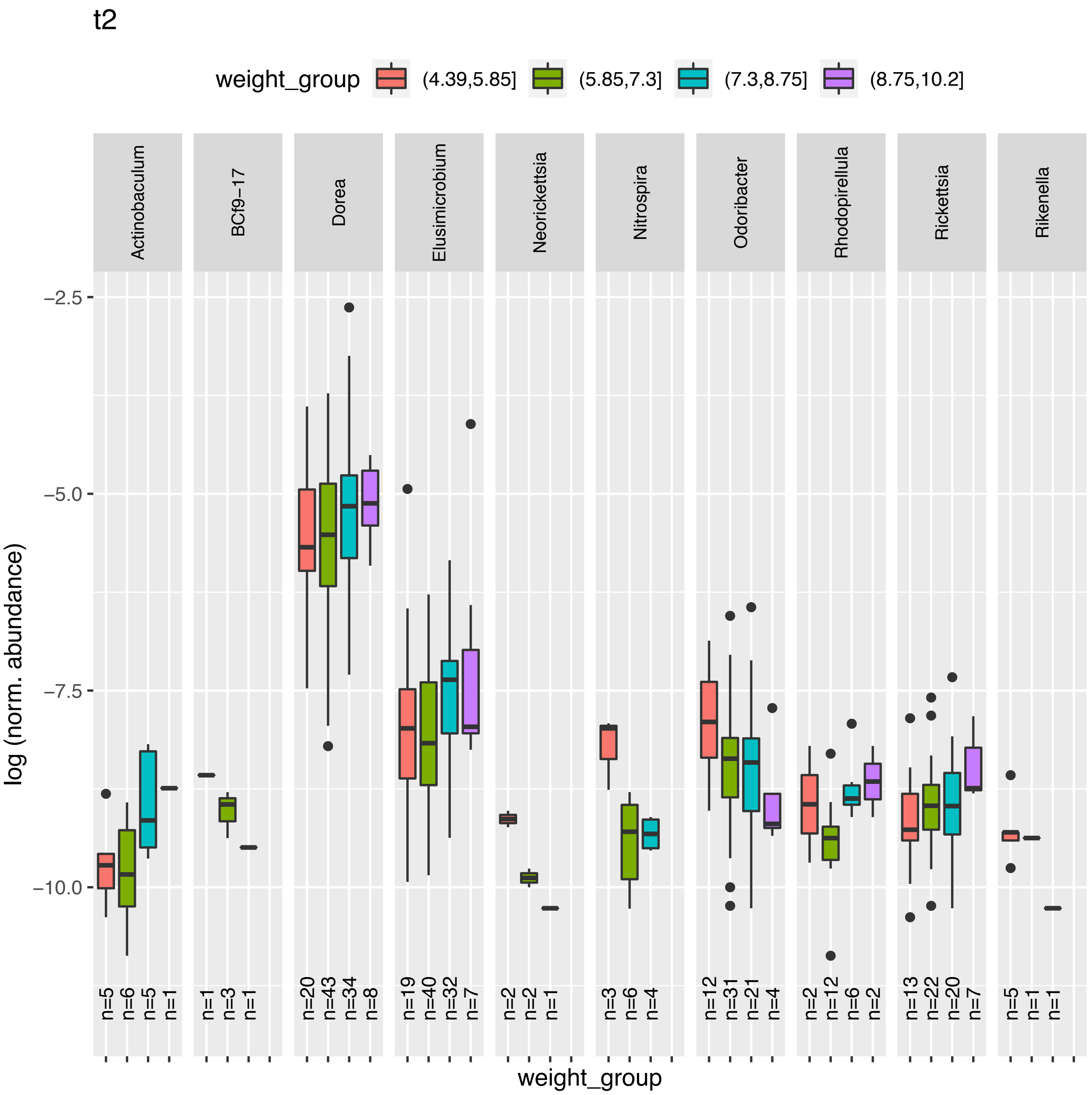

Supplementary Figure 29. Correlation of weight with taxonomy at time point 2.

16S rRNA reads were extracted with SortMeRNA. Abundance was estimated and correlation with weight was assessed with the Spearman method. Significance values are provided in Supplementary File 5. Plotted are the correlations with *p* value < 0.05. For visualization purposes, subjects are binned into weight groups (x-axis). Bins are formed with subjects whose weight falls below the first quartile (red), above the first quartile (green), below the third quartile (blue), above the third quartile (purple). At the bottom of each whisker plot, the samples size for each bin is provided.

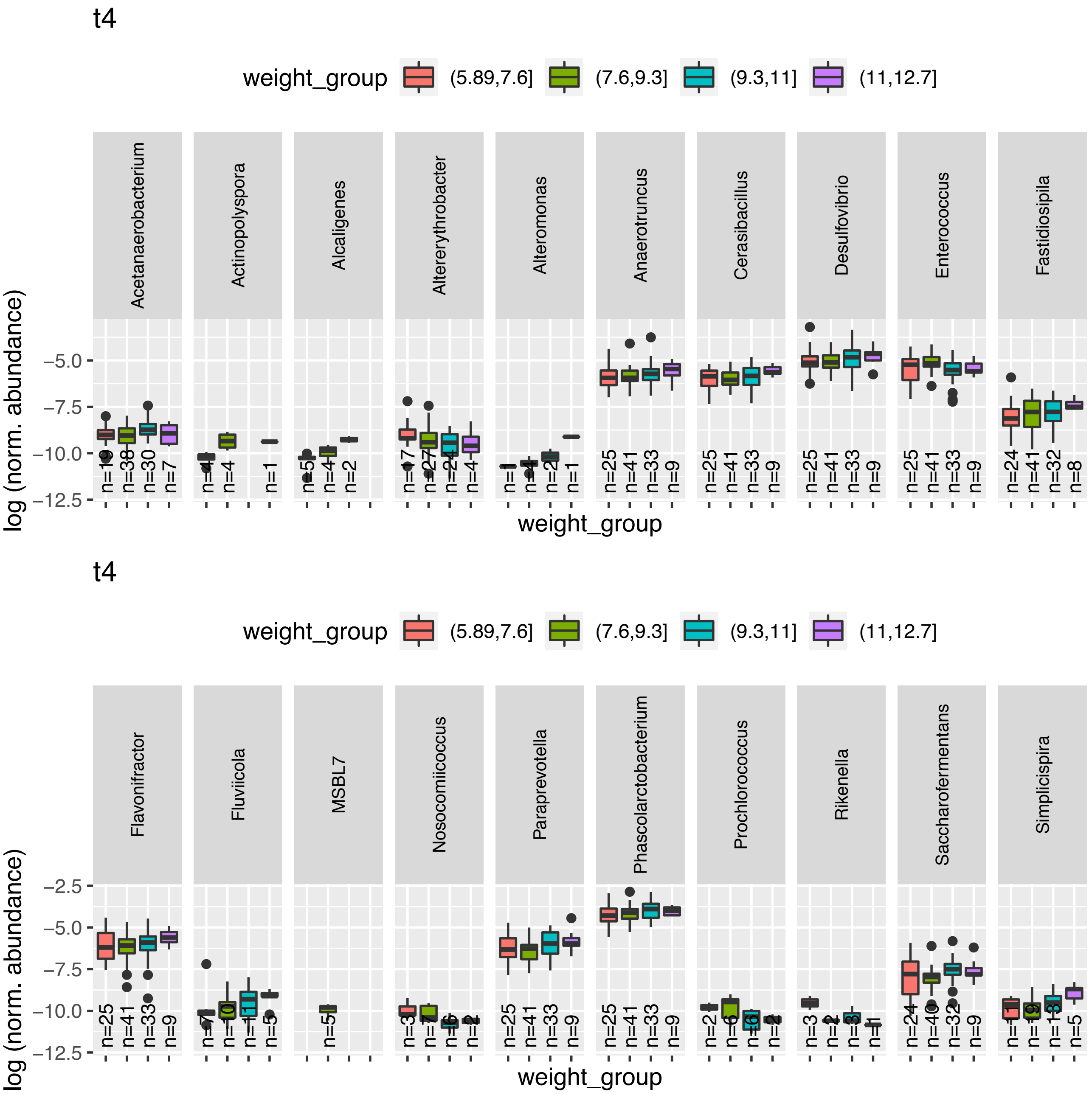

Supplementary Figure 30. Correlation of weight with taxonomy at time point 4.

16S rRNA reads were extracted with SortMeRNA. Abundance was estimated and correlation with weight was assessed with the Spearman method. Significance values are provided in Supplementary File 5. Plotted are the correlations with p value < 0.05. For visualization purposes, subjects are binned into weight groups (x-axis). Bins are formed with subjects whose weight falls below the first quartile (red), above the first quartile (green), below the third quartile (blue), above the third quartile (purple). At the bottom of each whisker plot, the samples size for each bin is provided.

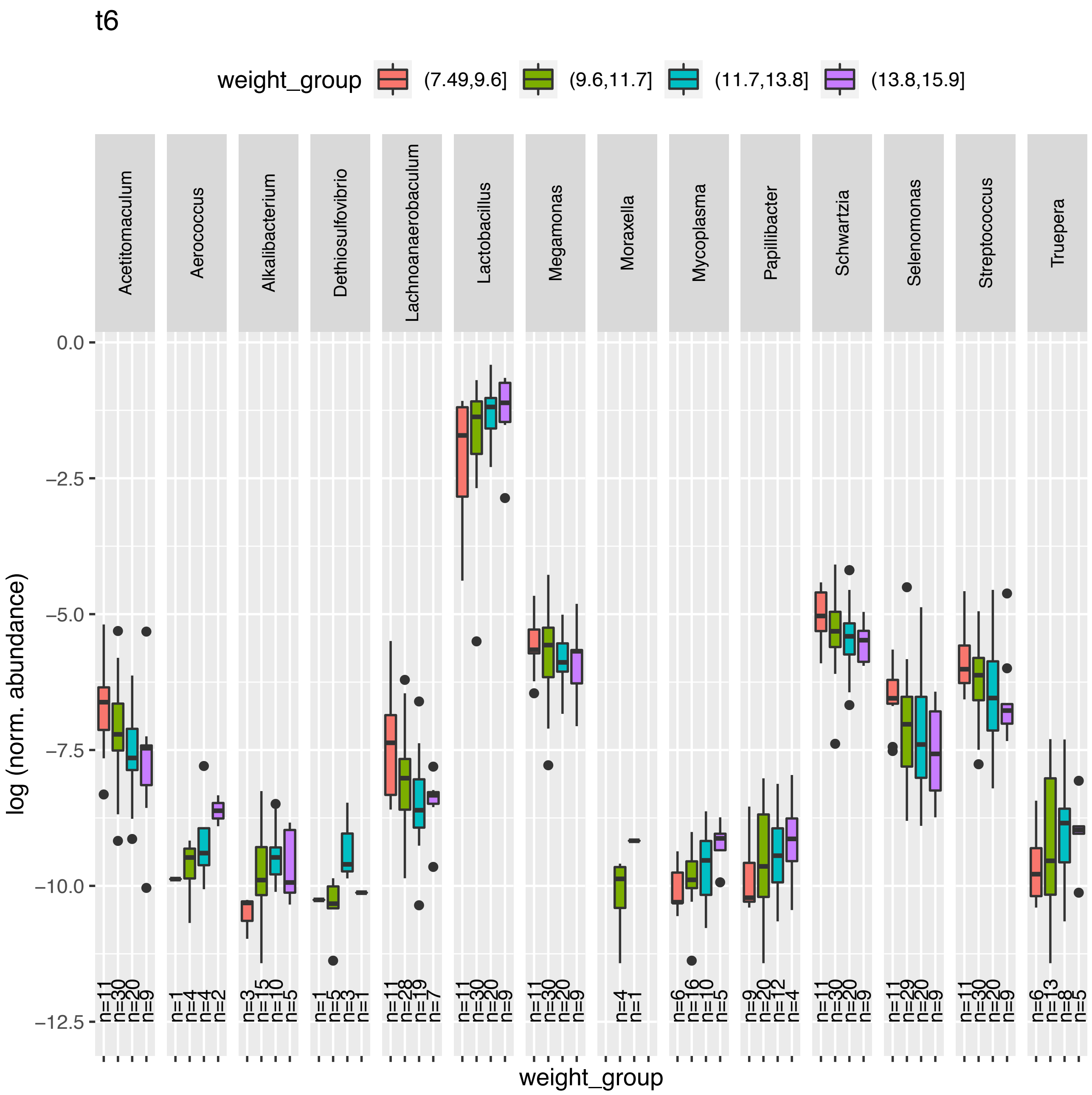

Supplementary Figure 31. Correlation of weight with taxonomy at time point 6.

16S rRNA reads were extracted with SortMeRNA. Abundance was estimated and correlation with weight was assessed with the Spearman method. Significance values are provided in Supplementary File 5. Plotted are the correlations with p value < 0.05. For visualization purposes, subjects are binned into weight groups (x-axis). Bins are formed with subjects whose weight falls below the first quartile (red), above the first quartile (green), below the third quartile (blue), above the third quartile (purple). At the bottom of each whisker plot, the samples size for each bin is provided.

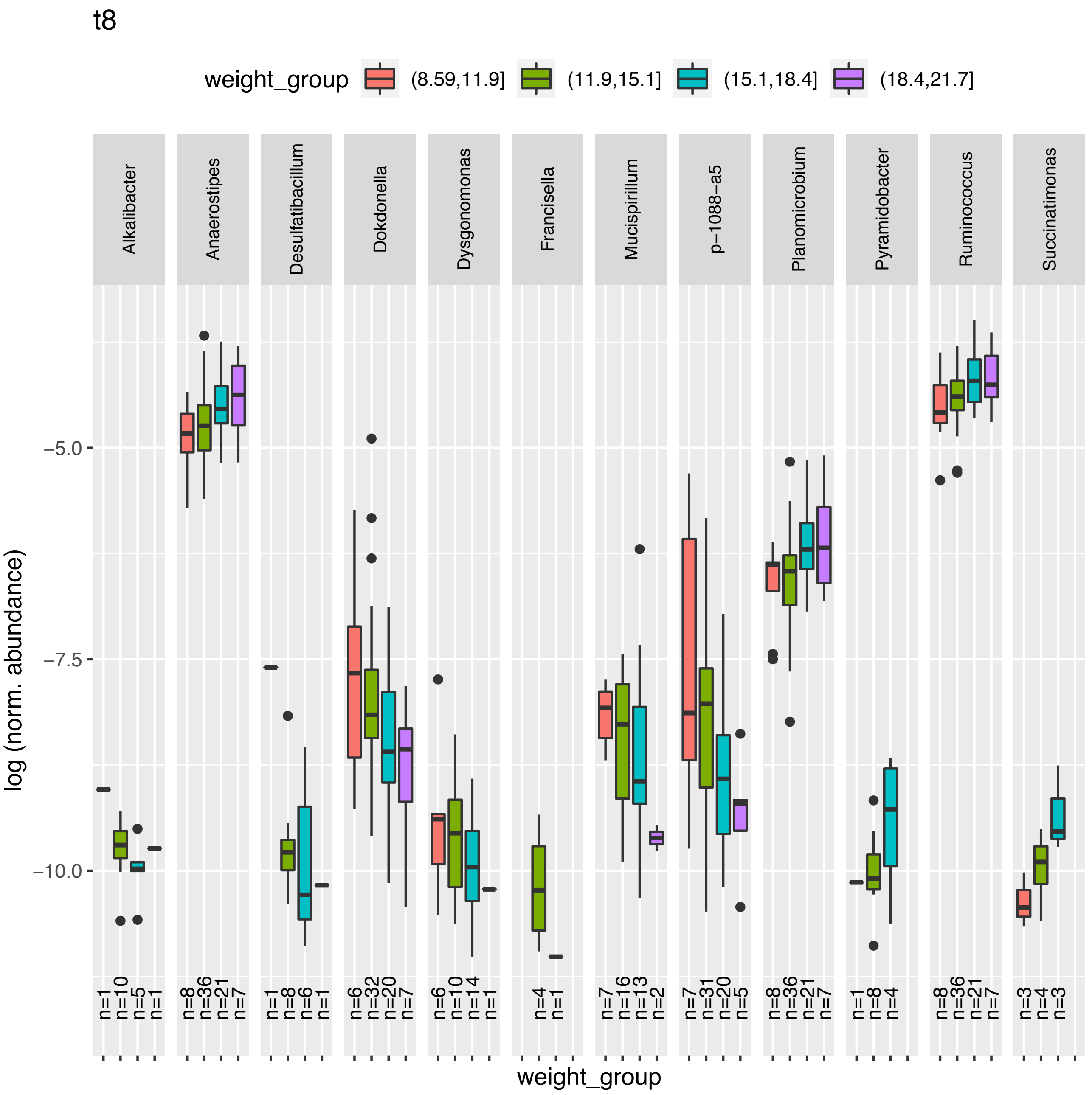

Supplementary Figure 32. Correlation of weight with taxonomy at time point 8.

16S rRNA reads were extracted with SortMeRNA. Abundance was estimated and correlation with weight was assessed with the Spearman method. Significance values are provided in Supplementary File 5. Plotted are the correlations with p value < 0.05. For visualization purposes, subjects are binned into weight groups (x-axis). Bins are formed with subjects whose weight falls below the first quartile (red), above the first quartile (green), below the third quartile (blue), above the third quartile (purple). At the bottom of each whisker plot, the samples size for each bin is provided.

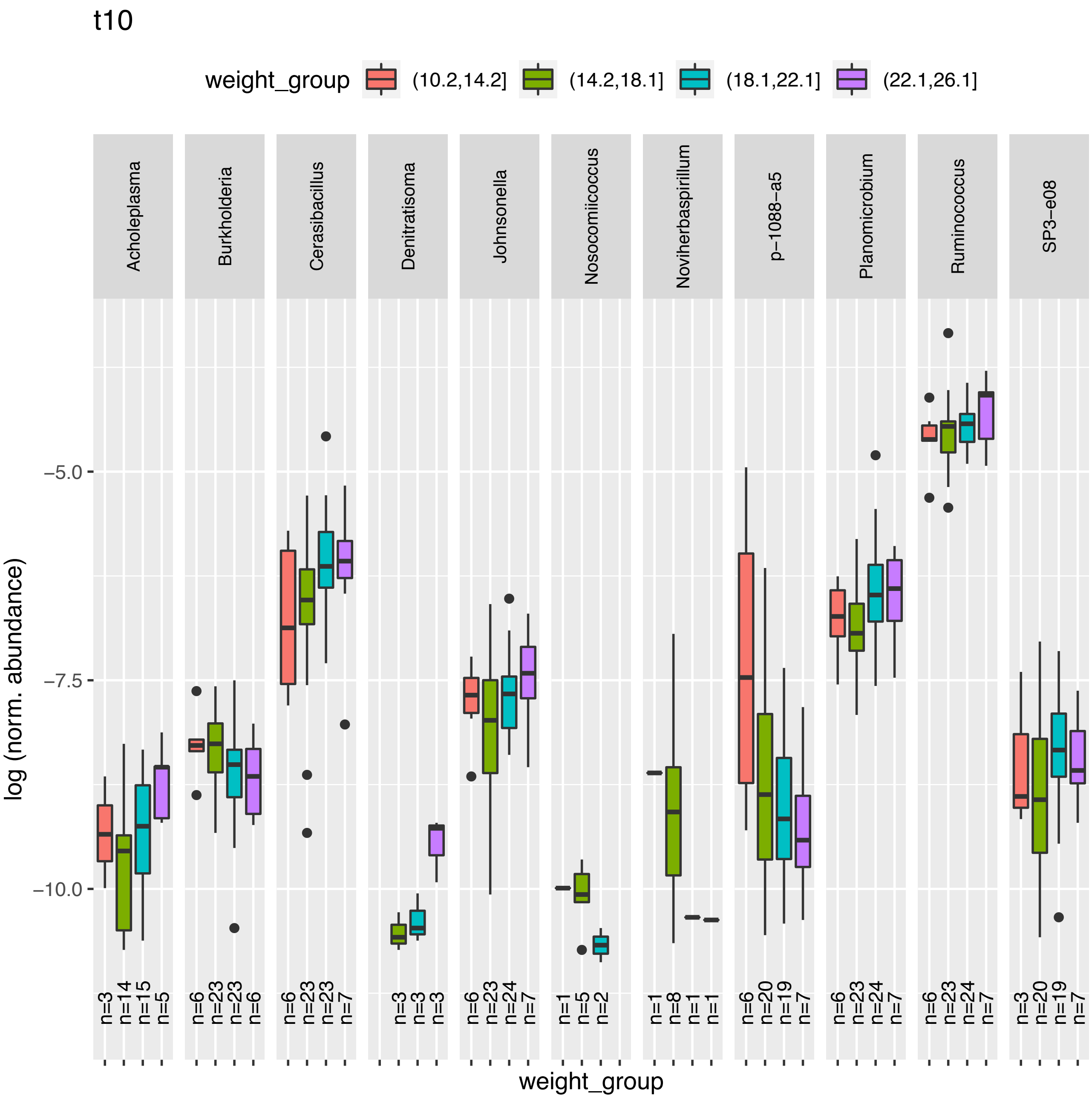

Supplementary Figure 33. Correlation of weight with taxonomy at time point 10.

16S rRNA reads were extracted with SortMeRNA. Abundance was estimated and correlation with weight was assessed with the Spearman method. Significance values are provided in Supplementary File 5. Plotted are the correlations with p value < 0.05. For visualization purposes, subjects are binned into weight groups (x-axis). Bins are formed with subjects whose weight falls below the first quartile (red), above the first quartile (green), below the third quartile (blue), above the third quartile (purple). At the bottom of each whisker plot, the samples size for each bin is provided.

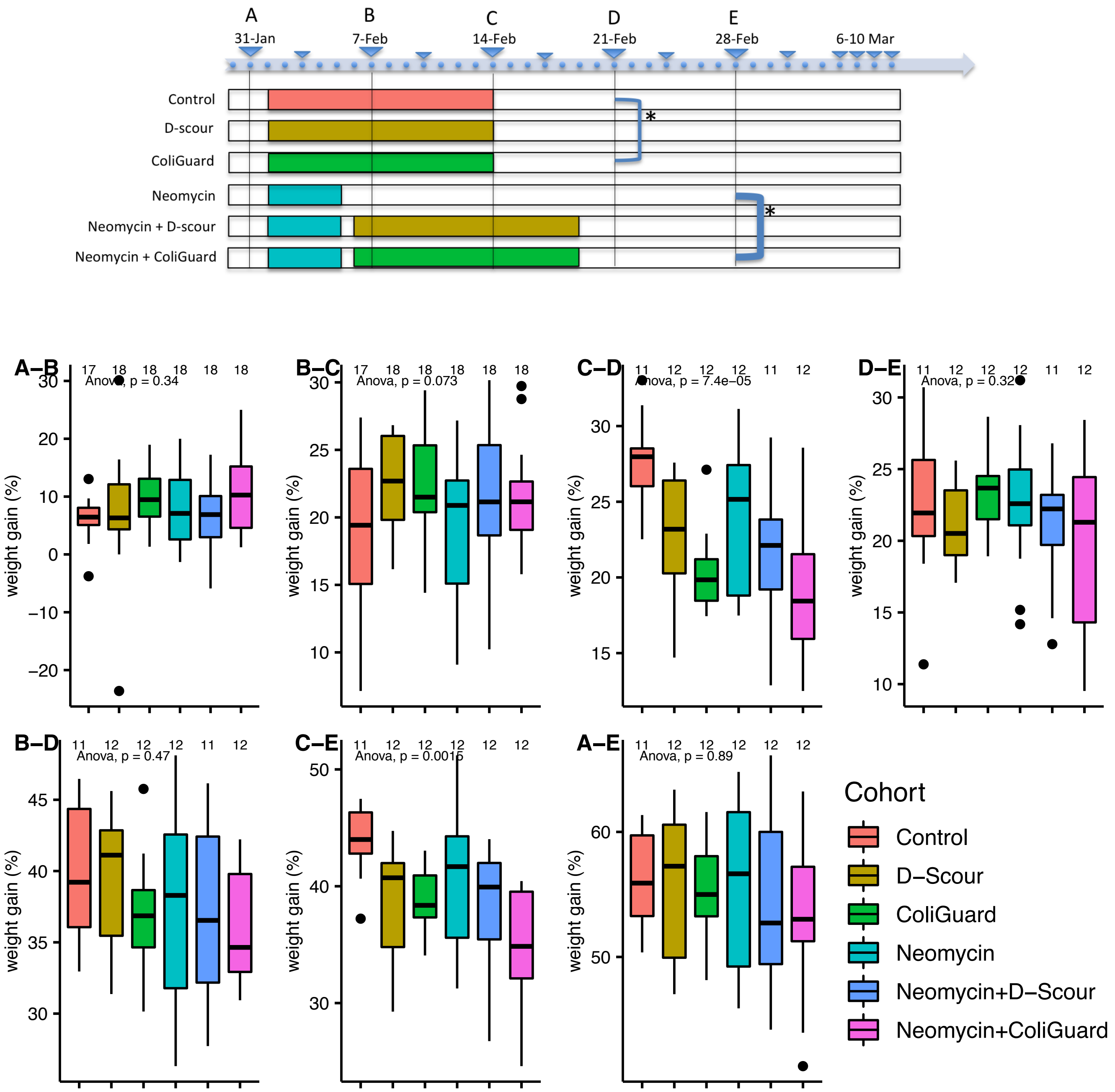

Supplementary Figure 34. Weight gain of piglets by cohort across the trial.

On the y-axis of plots the weight gain between time points is provided in percentage. Letters on the top left of each plot indicate the time points compared with one week interval (A-B, B-C, C-D, D-E) and with two weeks interval (B-D, C-E, A-E). Pairwise t-test comparisons between cohorts were computed. Brackets in the timeline (top) indicate a significant *p* value between cohorts (1-week interval); bold brackets indicate a significant *p* value between cohorts (2-weeks interval). A significant difference was found between Control and ColiGuard® (C-D, Tukey adjusted *p* value=0.000747) and between neomycin and neomycin+ColiGuard® (C-E, Tukey adjusted *p* value=0.039286). *in the timeline indicate that the significance was maintained after applying Tukey HSD *post-hoc* correction.

Click here to access/download
Supplementary Material
supp-1.xlsx

Click here to access/download
Supplementary Material
supp-2.xlsx

Click here to access/download
Supplementary Material
supp-3.csv

Click here to access/download
Supplementary Material
supp-4.zip

Click here to access/download
Supplementary Material
supp-5.xlsx
