# Supplementary material for: A large-scale metagenomic survey dataset of the post-weaning piglet gut lumen
Source: Gigascience. 2021 Jun 3;10(6):giab039. doi: 10.1093/gigascience/giab039 (PMC8173662; doi:10.1093/gigascience/giab039)
Supplement: giab039_GIGA-D-20-00347_Revision_1 [file giab039_giga-d-20-00347_revision_1.pdf]

GigaScience

A large-scale metagenomic survey dataset of the post-weaning piglet gut lumen

--Manuscript Draft--

**Manuscript Number:**	GIGA-D-20-00347R1
**Full Title:**	A large-scale metagenomic survey dataset of the post-weaning piglet gut lumen
**Article Type:**	Data Note
**Funding Information:**	Australian Research Council (LP150100912) — Dr Steven P Djordjevic
**Abstract:**	We report on the largest shotgun metagenomic analysis of the pig gut lumen microbiome to date. By processing over 800 faecal time-series samples from 126 piglets and 42 sows, we generated over 8Tbp of metagenomic shotgun sequence data. This study was conducted to generate a publicly available databank of the faecal metagenome of weaner piglets aged between 3 and 9 weeks old, treated with different probiotic formulations and intramuscular antibiotic treatment. Here we describe the animal trial procedures and the generation of our metagenomic dataset.
**Corresponding Author:**	Aaron Darling<br>University of Technology Sydney<br>Ultimo, AUSTRALIA
**Corresponding Author Secondary Information:**	
**Corresponding Author's Institution:**	University of Technology Sydney
**Corresponding Author's Secondary Institution:**	
**First Author:**	Daniela Gaio
**First Author Secondary Information:**	
**Order of Authors:**	Daniela Gaio
	Matthew Z DeMaere
	Kay Anantanawat
	Graeme J Eamens
	Michael Liu
	Tiziana Zingali
	Linda Falconer
	Toni A Chapman
	Steven P Djordjevic
	Aaron E Darling
**Order of Authors Secondary Information:**	
**Response to Reviewers:**	Reviewer #1: This study reported 8Tbp of shotgun metagenomic sequence data for 800 fecal microbiota collected<br>from 126 piglets and 42 sows. The samples were collected in a longitudinal manner and the treatments were well<br>controlled. However, your data analysis, especially shotgun sequence data, needs to be improved.<br>Here are my questions:<br><br>1. Why you did shotgun metagenomic sequencing rather than 16S rRNA gene sequencing as you only did<br>compositional analysis with this 8Tbp of sequence data. Analysis of 16S rRNA gene sequence could definitely do the

same thing and it's much easier and cheaper. It there is any advantages of your analysis over the common used
workflow with 16s rRNA gene sequence? The main advantage of shotgun sequence is the possibility to get more
functional information other than bacterial composition of our metagenomic samples. Why you didn't do any functional
analysis?

We generated the data with a view towards functional analysis, effects of antibiotic and probiotic treatment on
function, and particularly towards investigation of the resistome. Many analyses have been performed on the data,
including functional analysis, and it is too much material to describe in a single manuscript. Now that the Data Note
format has been adopted at the Editor's suggestion, this manuscript contains only a summary-level analysis of the
data that will be referenced by the other manuscripts which describe detailed analyses.

2. What is the significance of this study? The strong effect of time /age on gut microbiota of post-weanling pig have
been reported in many publications.

The main purpose of the study was to evaluate the effects of probiotic and intramuscular antibiotic interventions on
the gut microbiome. Secondly, to describe the antimicrobial resistance gene repertoire in an Australian setting and its
change throughout the post-weaning period, and in relation to antibiotic treatment. Now that the Data Note format has
been adopted, the potential uses are listed in the "Potential uses" section.

Detailed comments:
Line 160 Taxonomy of the genus Lactobacillus has been reclassified. Please see more details in this reference : "A
taxonomic note on the genus Lactobacillus: Description of 23 novel genera, emended description of the genus
Lactobacillus Beijerinck 1901, and union of Lactobacillaceae and Leuconostocaceae"

In the interest of reproducibility of analysis we used a public database that was integrated to an automated analysis
software package at the time the analysis was carried out.

Line 229 How is the quality of the extracted 16S gene reads? How many reads were extracted for each sample?
Further details on the 16S reads have been added to the "Taxonomic profiling of samples" section.

Line 230 How did you assign taxonomy to the extracted 16S gene reads? Based on sequencing identity? What are
your parameters for difference taxonomic levels (e.g genus/ family levels)???? Did you cluster the identical reads into
OTUs before assigning taxonomy?

Taxonomic profiling now, in the revised manuscript has been performed in the following manner:
1. rRNA gene containing reads were extracted with SortMeRNA (quality stats are obtained)
2. Reads extracted with SortMeRNA (fastq format) were filtered based on evalue, seq identity, and alignment
length, then fed to the RDP classifier, which assigns taxonomy via a Naive Bayes classifier algorithm
(Wang, Q., Garrity, G. M., Tiedje, J. M., & Cole, J. R. (2007). Naive Bayesian classifier for rapid
assignment of rRNA sequences into the new bacterial taxonomy. Applied and environmental

microbiology , 73 (16), 5261-5267)
This methodology has been reported in the revised Data Note format manuscript.

Line 231 Why yo only use e-value for hits filter? How about the query coverage and identity?

We have rerun the analysis and updated the results in the revised manuscript. We have used SortMeRNA to extract
16S rRNA reads, we have filtered those reads based on an e-value cutoff (1x10^-30), sequence identity of 80% and
alignment length of 100 bp. We fed the filtered reads to RDP classifier, then plotted with Krona.

Line 253 There are too many results and some of them are redundant and less informative. Some of the figures could
be integrated into one and the less relevant results should be deleted.

The Data Note format manuscript is now very short, it has a few figures, and only a few supplementary figures.

Reviewer #2: Gaio et al have analysed the phylogenetic diversity of the fecal pig bacterial communities obtained from
a large temporal piglet study in the period just after weaning. An interesting period due to all sorts of practical
problems that may arise during and after the weaning period. They used shotgun-metagenomics to determine that
phylogenetic diversities are influenced by litter and mainly age. Changes induced by intra muscular injection of an
aminoglycoside only had subtle effect on the community compositions. The metagenomic data described in this study
and shared to the public by NCBI SRA provide a great resource to standardized microbiota data in pigs in the early
age just after weaning. I thank the authors for learning other ways to approach metagenomic community structures.
We thank the reviewer for the extensive and detailed comments. We believe this manuscript (and its successors)
have significantly improved as a result of the reviewer's efforts.

Generic;
Even though the conclusions, mostly based on weighted phylogenetic diversity, seem supported by the data, overall
the paper confuses me and seems rather lengthy for the actual analysis performed. The same accounts for the too
many supplemental figures to this paper. Furthermore, I do have questions on why this approach was chosen in the
first place, since I cannot derive that directly from the paper in its current form and more simple approaches would
allow the same questions to be answered at the same or even better resolution.
The used shotgun-metagenomics seems rather overdone to the actual questions answered. I assume this paper will
be a predecessor of another follow-up paper describing changes in the resistome as well as functional turn-over in
time linked to the treatments. But I wonder whether salami-slicing this study would be the way to go. Furthermore, a
lot of the data and outcomes are presented as-is while interpretation of these outcomes in context is rather thin and
confusing using for instance both phylogenetic distance (PD) and the weighted forms.

We hope that the adoption of the Data Note format addressed most of these concerns.

More specific thoughts.
Even though they seem many, I hope they will help to improve the next version of the manuscript as constructive as
possible;

	1) A good balanced depth of sequencing per sample was chosen (around 30M PE150 clusters per sample) which<br>seems sufficient as seen in other studies that used SGS in a metagenomics approach in pigs. But why on earth were<br>only the first 100k reads for analysis?? The rationale for this as well as for instance rarefaction-like curves to<br>demonstrate 100k is enough, are missing.<br><br>This was initially done as a means to reduce the compute requirements for a preliminary analysis. However, that part<br>of the manuscript has been removed as a consequence of adopting the Data Note format.<br><br>2) Why was SG-metagenomics used in the first place? All questions could have been answered by a far cheaper<br>meta-taxonomic approach? SG-metagenomics studies are usually warranted if additional questions to bacterial<br>composition are answered like:<br>a. Non-bacterial microorganisms like simple eukaryotes, archaea and phages.<br>b. Other gene compositions like the (acquired) resistome.<br>c. Functional turn-over.<br>The analyses could probably suffice from fairly simple permanova community composition comparisons, weighted<br>unfirac beta-diversities and negative binomial modelled differential taxa analysis?<br><br>Yes, the purpose of the study is in fact much broader than presented in this particular manuscript, and we used, and<br>will use the data in future analyses, to answer additional questions. The revised manuscript lists several of these<br>downstream analyses.<br><br>3) I miss from the paper the rationale for using unweighted and weighted PD's instead of for instance a<br>straightforward weighted unifrac in beta-diversity analysis. It seems overcomplicated where the results describe every<br>time both weighted and unweighted PD's. Is this necessary? And if so why? If weighted PD would be to go, leave out<br>the rest. Would simplify and shorten considerably improving readability to a larger (metagenomic oriented) audience.<br><br>As weighted and unweighted PDs reveal different aspects of the microbial composition, the richness and the<br>evenness, respectively, we used both measures. For instance, a probiotic treatment could affect the evenness but not<br>the richness of a community, or vice versa. Therefore interrogating the data regarding the weighted, as well as the<br>unweighted PD, could deliver insights that would otherwise go unnoticed. However, this part of the analysis is now<br>excluded in the Data Note format.<br><br>4) The title says "…gut microbiome.". Actually the gut was only sampled using a proxy of fecal sampling. Fecal<br>composition is know not to be the same as in other regions of the gut. Revise throughout.<br>We agree, this is an important distinction and one that is often not made very clearly by the scientific community,<br>where “gut microbiome” is commonly used interchangeably with “faecal microbiome”.<br><br>We revised throughout.<br><br>5) The piglet/litter origin linked to the sows were found to be of influence on the piglet fecal community. It would be

	interesting to include analysis to demonstrate whether the sow is the actual driver for the piglet community<br>composition early and later in the study.<br><br>Yes it is an interesting analysis, but all specific analyses are now excluded in the Data Note format.<br><br>6) The interactions/influence of piglets "at random" in one pen may be interesting to investigate since it may influence<br>the cross-sectional outcomes instead of following the same animal over time. It is known that the order in which taxa<br>arrive disproportionate influence later community assembly (Sprochet et al 2018).<br><br>We agree this is an interesting analysis. However, all specific analyses are now excluded in the Data Note format.<br><br>7) A Major point in this paper is made about the potential dysbiosis due to (oral) antibiotic treatment. Therefore intra<br>muscular injection was used to study the effects on community composition.<br>a. However, since the microbiome and resistome diversity prior to starting antibiotic treatment is unknown and a<br>control group having administered antibiotics orally, seems a bit odd.<br>b. Furthermore, it would make the study stronger to show that antibiotic residues are actually measured in feces. This<br>can be done on fecal samples and would strengthen this part of the study.<br>c. The shotgun metagenomic data is there, why wasn't the effect of antibiotics on the resistome not included? This<br>can be fairly easy achieved using mapping strategies.<br><br>These are good suggestions that will likely be included in another Research-format manuscript.<br><br>8) Normalisation, sequencing and standardisation. The overall method is elegant using MiSeq to equilibrate libraries<br>before sequencing. Some remarks;<br>a. The authors find that DNA isolation efficiency is biased for groups of species using their community controls. Was<br>this a surprise? There is an elegant paper describing DNA isolation kit issues in metagenomic shotgun applications of<br>for instance pig feces (Knudsen et al 2016 mSystems 1(5):e00095-16). Why not adhere to described methods?<br><br>No, the extraction bias was not a surprise. The gram stain associated bias has been reported extensively before, and<br>we were aware of this. As it has been suggested by Knudsen et al (2016), methods without bead beating or<br>enzymatic treatment have extracted less DNA from Gram-positive bacteria. Based on the literature available, we<br>chose to perform bead beating as well as an enzymatic treatment during extraction. However, the MagAttract<br>PowerMicrobiome kit (Qiagen) had a 96-well option and its performance was well characterised in the literature at the<br>time. The QIAamp fast stool kit, suggested to have a better performance by Knudsen et al (2016), was a new product<br>and requires a QIAcube machine for high throughput processing, which we did not have access to. We edited the<br>new manuscript to report on this study and suggestion of better performance with this kit.<br><br>b. Input is "standardized" on total from fecal samples isolated DNA measurements. It is known that especially due to<br>dysbiosis (i.e. diarrhea) that the ratio of microbial DNA to host or feed-derived DNA may completely shift. The amount<br>of reads per sample mapped to host or non-microbial origin is helpful and the microbial

amounts should be used for
normalization unless it is demonstrated that no significant differences are found between samples for this.

Yes, this is part of the data analysis process. Given that no extensive analyses are described in the Data Note, this is
now not included.

c. Hackflex: Seems an elegant method to try. However, I am not sure on editorial policy referencing a pre-publication
by the same author in BioRxive of over a year old with apparent no peer-reviewed follow-up? Furthermore,
pre-publications do not free authors from discussing/reflecting on comments by readers (if they make sense) to their
pre-pub like the one over a year old without a response.

That manuscript is currently under peer review, however we have been advancing it very slowly due to timing
constraints of the Australian PhD system, which demands that attention be focused first on thesis submission and
peer review, with journal publications taking 2nd priority.

d. Mock communities / control libraries; elegantly made using a cfu approach.
i. However, also dead-bacteria will contribute DNA to the library. Since no strong cfu correlations are made this might
be only of minor effect.

We agree. This is a limitation, as f requency of dead bacterial cells would not be measured by the CFU approach, yet
they could contribute to total DNA and modify relative abundances from read count.

ii. Did the authors check for the presence of plasmids?

Plasmids were not considered in the analysis and a simplifying assumption was made that each bacterial cell
contains 1 copy of the chromosome. That is, the presence of multiple partially replicated copies that are commonly
found in fast growing cells was not considered.

e. Why is a different method used for analyzing the (positive) controls using MethaPhlan2 while the rest of the
samples is analysed by other means like PhyloSift. Positive controls should be used to benchmark the flow and
analysis of the actual samples and therefore I would argue the methods should be the same.

MetaPhlAn2 has high precision in taxonomic assignment and unlike PhyloSift, it is designed to give taxonomic names
so that we could check whether the species expected in the controls were also observed, and to estimate the
contamination rates. We did not run MetaPhlAn2 on pig samples as were not purely after a taxonomic profiling of the
pig microbiome, but the main purpose of the previously submitted manuscript was to determine whether or not an
effect on composition was detected following probiotic or antibiotic treatment, and this was obtainable using
phylogenetic diversity measures, which was done on all samples, positive controls as well as pig samples. Another
major advantage of the use of phylogenetic diversity measures is that the approach circumvents the library size
normalization, with all the issues that accompany the normalization of samples of compositional nature. However,
now that the extensive data analysis is excluded from the Data Note manuscript, this should no longer be a concern.

We also made a remark in the first paragraph of the section "Technical controls in metagenomic studies and
methodological limitations" on the biased representation of organisms in databases, and the narrowed view that can
be obtained when mapping real samples against such a database.

f. Unfortunate some contaminants were detected in the positive (and probable negative) controls. How did the
authors handle these outcomes? Were corrections applied to the data? Were taxa blacklisted or even removed?

We strongly suspect that the contamination derived from cross-well contamination. The "blacklisting" or removal
belongs to the data analysis step, which is now excluded in the Data Note format.

9) Analysis methods;
a. The KR distance metric was used which seems rather comparable to weighted unifrac. I could not quickly recover
if the KR metric returns Euclidian distances, if not revise the PCA into PCoA.

This part is no longer present in the Data Note format.

b. The weighted and unweighted PD is used. Shouldn't the unweighted PD be performed on rarefied data (at least for
alpha-diversity analysis)? See for instance "McCoy and Matsen (2013) PeerJ 1:e157; DOI 10.7717/peerj.157".

This part is no longer present in the Data Note format.

c. Correcting for bath-effects was done using COMBAT. There is a no-go for me on this. The batch-effect should be
taken as a covariate in the used statistical models and not used to correct the raw-data. This is elegantly
demonstrated by Nygaard et al 2016 Biostatistics 17:29-39.

This part is no longer present in the Data Note format.

10) The section on sortmeRNA method and results are not well integrated in the manuscript and in its current state
only deviate from the main message.

Now that the phylogenetic diversity analysis is removed from the Data Note format manuscript this should no longer
be a concern. The SortMeRNA analysis remains, with the purpose of providing a high level taxonomic overview of the
dataset.

11) Lines 641-646: I found this a very honest remark but a major flaw in processing. If samples get contaminated
from neighboring wells…. What do the actual results tell us?

Even though there was cross-contamination, the amount of contamination was still relatively low. In fact, it
did not prevent us from observing the strong (time trend) as well as the minor trends (treatment effects; small
differences in age of the piglets). While the relative abundances in each sample are preserved, analyses of low
abundance components are rendered much more challenging, as a result of the cross-well contamination.
Cross-well contamination has also been reported by Minich et al (2019) (see " Quantifying and understanding
well-to-well contamination in microbiome research "). This is a flaw that derived from the design of the rubber sealing
mats of the DNA extraction kit, described in the revised manuscript.

Powered by Editorial Manager® and ProduXion Manager® from Aries Systems Corporation

Figures:
- The message from figure 2 is difficult to get? Change for instance its display into line plots and only marking
significant differences? Since results fulfill a tested hypothesis or not and are significant different or not. Having a
numerical scale for raw and post-hoc corrected p-values seems odd. Unless a point is made in the paper why to do
so.

This part is no longer present in the Data Note format.

- Figure 3; I understand the power of the chosen analysis an display is the phylogenetic linking to the axes on display.
Readability of the top figure is however zero. Should be improved. The below figure using 16S seems out-of-context
in the paper (see earlier remark). Furthermore it is unreadable. Maybe improve by substituting species names to
letters and provide these in a legend.

This part is no longer present in the Data Note format.

- Figure 4: From which data the heatmap is inferred from? From all the different treatments or subset of only the
controls?

This part is no longer present in the Data Note format.

- Supplementary figure4; perhaps move to main part of the paper?

Given the major edits in the Data Note format, we edited this figure to reflect the workflow described in the current
manuscript, and as requested, included it as a figure, rather than as a supplementary figure.

- Remaining supplementary figures are too many for me (see earlier remark). Make a choice and condense to the
main messages and explain these in the text.

Many figures and supplementary figures have now been removed in the Data Note format.

Textual:
- Line 206: revise NovaSeq S4 into NovaSeq 6000 (S4) or similar. The NCBI archive has it correct.

Revised.

- Line 186: amplification step should be elaborated.

Elaborated.

- Analysis methods:
o R exact version should be given as well as its used packages.

Only a few packages are now used in the Data Note format.

Versions are provided for all the tools used.

o The used random seed in all analysis is missing.

This part is no longer present in the Data Note format.

o To increase reproducibility the github repository should provide either a version-fixed

Powered by Editorial Manager® and ProduXion Manager® from Aries Systems Corporation

	set of scripts exactly as used<br>for the manuscript or a version tag to the exact used scripts. Otherwise, if scripts get updated in time reproduction of<br>results is no longer possible.<br><br>Good suggestion, we agree. We created a version tag.<br><br>- Line 508: …trend in community composition -changes?- over time?<br><br>This part is no longer present in the Data Note format.<br><br>- Lines 624-631 seem disjoint from the paper. Remove?<br><br>This part is no longer present in the Data Note format.<br><br>- Line 639: See remark above on DNA isolation bias and the Knudsen paper.<br><br>Reported on gram-pos/neg bias, and cited the Knudsen paper.
Additional Information:	
**Question**	**Response**
Are you submitting this manuscript to a special series or article collection?	No
**Experimental design and statistics**<br><br>Full details of the experimental design and statistical methods used should be given in the Methods section, as detailed in our Minimum Standards Reporting Checklist. Information essential to interpreting the data presented should be made available in the figure legends.<br><br>Have you included all the information requested in your manuscript?	Yes
**Resources**<br><br>A description of all resources used, including antibodies, cell lines, animals and software tools, with enough information to allow them to be uniquely identified, should be included in the Methods section. Authors are strongly encouraged to cite Research Resource Identifiers (RRIDs) for antibodies, model organisms and tools, where possible.<br><br>Have you included the information requested as detailed in our Minimum	Yes

Standards Reporting Checklist?	
**Availability of data and materials**<br><br>All datasets and code on which the conclusions of the paper rely must be either included in your submission or deposited in publicly available repositories (where available and ethically appropriate), referencing such data using a unique identifier in the references and in the “Availability of Data and Materials” section of your manuscript.<br><br>Have you have met the above requirement as detailed in our Minimum Standards Reporting Checklist?	Yes

Manuscript Click here to access/download;Manuscript;metapigs_base_DataNote_ms.do

A large-scale metagenomic survey dataset of the post-weaning piglet gut lumen

Abstract

We report on the largest shotgun metagenomic analysis of the pig gut lumen microbiome to date. By processing over 800 faecal time-series samples from 126 piglets and 42 sows, we generated over 8Tbp of metagenomic shotgun sequence data. This study was conducted to generate a publicly available databank of the faecal metagenome of weaner piglets aged between 3 and 9 weeks old, treated with different probiotic formulations and intramuscular antibiotic treatment. Here we describe the animal trial procedures and the generation of our metagenomic dataset.

Data description

The dataset includes 911 samples, comprising a total of 27 billion raw sequence reads. Preliminary analysis of the dataset consisted in the extraction of 16S rRNA gene containing reads with SortMeRNA [1] and their classification with the RDP classifier [2]. In terms of taxonomic diversity, most OTUs (75.71%) were assigned to the *Firmicutes* phylum. The next most abundant bacterial phyla were: *Bacteroidetes* (13.21%), *Actinobacteria* (5.10%), *Proteobacteria* (3.36%), and *Spirochates* (0.69%). A representation of the microbial diversity, obtained with Krona [3], is shown of the post-weaning piglets (**Figure 1A**) and of the mothers (**Figure 1B**). Interactive maps are available as html files in our Github repository (https://github.com/GaioTransposon/metapigs_base).

Pig trial and sample collection

Animal studies were conducted at the Elizabeth Macarthur Agricultural Institute (EMAI) NSW, Australia and were approved by the EMAI Ethics Committee (Approval M16/04). The trial animals comprised 4-week old male weaner pigs (*n*=126) derived from a commercial swine farm and transferred to the study facility in January 2017. These were cross-bred animals of “Landrace”, “Duroc” and “Large White” breeds and had been weaned at approximately 3 weeks of age (**Supplementary Table 1**).

The pig facility consisted of four environmentally controlled rooms (Rooms 1-4) with air conditioning, concrete slatted block flooring with underground drainage and open rung steel pens (**Supplementary Figure 1**). Each room had nine pens, consisting of a set of six and a set of three pens, designated a-f and g-i respectively, with the two sets of pens being physically separate, *i.e.* animals could come in contact with each other through the pen’s bars within each set of pens, but not between sets. The rooms were physically separated by concrete walls and contamination between rooms was minimized by using separate equipment (boots, gloves, coveralls) for each room. In addition, under-floor drainage was flushed twice weekly and the flushed faeces/urine was retained in under-floor channels that ran the length of the facility, so that Rooms 1, 2 were separate from Rooms 3, 4 and flushing was in the direction 1 to 2 and 3 to 4.

The pigs were fed *ad libitum* a commercial pig grower mix of 17.95% protein free of antibiotics, via self-feeders. On the day of arrival (day 1) 30, 18, 18, and 60 pigs were allocated randomly to Rooms 1, 2, 3 and 4 respectively in groups of 6, 6, 6 and 6-7 pigs per pen respectively (**Supplementary Figure 1A**). Pigs were initially weighed on day 2, and some pigs were moved between pens to achieve an initial mean pig weight per treatment of approximately 6.5 kg (range: 6.48-6.70; mean±SD: 6.53±0.08). Pigs were weighed weekly throughout the trial, and behaviour and faecal consistency scores were taken daily over the 6-week period of the trial (**Supplementary Table 2**). Developmental and commercial probiotic paste preparations ColiGuard® and D-Scour™ from International Animal Health, were used in some treatment groups.

The animals were acclimatised for 2 days before the following treatments were administered: Room 1 - oral 1 g/pig of placebo paste daily for 14 d; Room 2 - oral 1 g/pig of D-Scour™ paste daily for 14 d; Room 3 - oral 1 g/pig of ColiGuard® paste

daily for 14 d; Room 4 - intramuscular (IM) injection of antibiotic administered at 0.1 mL per pig daily from a 200 mg/mL solution for a total treatment duration of 5 d.

On the day following the final neomycin treatment (day 8), 36 pigs were moved from Room 4 to Room 2 (*n*=18, 6 in each pen, pens g-i), and to Room 3 (*n*=18, 6 in each pen, pens g-i) (**Supplementary Figure 1B**). The following day (day 9), oral administration of D-Scour™ (1 g/pig) and of ColiGuard® (1 g/pig) commenced for pigs in Room 2 pens g-i and in Room 3 pens g-i, respectively, and continued for a period of 14 days. Assignment of the 36 neomycin-treated pigs to the treatment groups neomycin+D-Scour™ (*n*=18; Room 2 pens g-i) and neomycin+ColiGuard® (*n*=18; Room 3 pens g-i), was carried out by distributing them so that the mean weight of the animals distributed across pens and rooms was similar. By this time point, each occupied pen in the trial housed six pigs. (**Supplementary Figure 1B**) From that time, twelve piglets from the original 126 were no longer present, as they had been euthanised as pre-treatment controls at the start of the trial.

Faecal samples were collected from all piglets once per week and from a subset (*n*=48 pigs; 8 from each of the six cohorts) twice per week over the 6-week study period (**Figure 2**). From each piglet, faeces were collected per rectum with new disposable gloves; where minimal or no faeces could be collected on a collection day, sampling was performed the following morning. Samples were placed in 50 mL Falcon tubes and stored at 4°C within 30 mins of sample collection for a minimum of 30 mins and a maximum period of 6 h.

Faecal sample processing

Samples (3g/pig) were mixed with 15 mL PBS (200 mg/mL), in sterile stomacher bags and homogenized with a Bio-Rad stomacher. The homogenised samples were divided in replicates: one replicate was stored directly at -80°C and one replicate was supplemented with glycerol (20% v/v) (Sigma-Aldrich) then stored at -80° C. In addition, single time-point faecal samples from the dams of the trial pigs (*n*=42) were obtained from the commercial facility of origin and were pre-processed at EMAI as described above. Thus, a total of 911 unique samples, between one and ten samples per subject (mean: 4.8; median: 3) (**Supplementary Table 2**), were obtained throughout this study. At the end of the trial period, all samples were transported from EMAI to

the University of Technology Sydney (UTS) for further processing. The experimental workflow is schematically represented in **Figure 3**.

Positive controls

As a positive control "mock community" for this study, four Gram positive (*Bacillus subtilis* strain 168, *Enterococcus faecium*, *Staphylococcus aureus* ATCC25923, *Staphylococcus epidermidis* ATCC35983) and three Gram negative (*Enterobacter hormaechei* CP_032842, *Escherchia coli* K-12 MG1655, *Pseudomonas aeruginosa* PAO1) bacterial strains from -80℃ stocks were cultured at 37°C for 16 h in LB (Luria-Bertani) then centrifuged at 14,000 rpm for 10 mins. From the resulting pellets, 1 g was transferred to 1 mL of LB and homogenised and a 1:10 dilution of this was made for each bacterial culture. Ten microliters of bacterial suspension from each of the cultures was used to determine the number of colony forming units (CFU) in the original suspension in the following manner: by further diluting tenfold in LB and by plating onto 1.6% LB agar plates and incubated overnight. The remaining suspensions (990 µL from each bacterial culture) were pooled into a sterile tube, then aliquoted into Eppendorf tubes in 500 µL volumes/tube. As a washing step, Eppendorf tubes were centrifuged at 14,000 rpm for 10 mins, 500 µL PBS was added to the pellet and subsequently resuspended. These tubes constituted the mock community samples and were stored at -80°C. Expected proportions of the mock community members were determined from the estimated colony forming units (CFU) multiplied by the genome size and were as follows: 8.7:13.0:7.7:16.7:38.9:14.5:0.4 for *S. aureus*, *B. subtilis*, *E. faecium, S. epidermidis*, *P. aeruginosa*, *E. cloacae, and E.coli* respectively.

The two probiotic formulations used in this study were used as two additional positive controls. D-Scour™ is a commercially available probiotic formulation for livestock, with each gram containing 180 million CFU of the following: *Lactobacillus acidophilus*, *Lactobacillus delbrueckii* subspecies *bulgaricus*, *Lactobacillus plantarum*, *Lactobacillus rhamnosus*, *Bifidobacterium bifidum*, *Enterococcus faecium*, *Streptococcus salivarius* subspecies *thermophilus*, with an additional 20 mg of garlic extract (*Allium sativum*). The probiotic ColiGuard is a probiotic formulation developed for the treatment of entero-toxigenic *Escherichia coli* (ETEC) in weaner pigs, developed in collaboration between the NSW DPI and International Animal Health

Products, containing undefined concentrations of *Lactobacillus plantarum* and *Lactobacillus salivarius*.

DNA extraction

Piglet and sow faecal samples, mock community samples, negative controls and probiotic samples (D-Scour™ and ColiGuard® paste) were allocated to a randomized block design to control for batch effects in DNA extraction and library preparation. The faecal samples were thawed on ice first, followed by the probiotics and mock community samples. MetaPolyzyme (Sigma-Aldrich) treatment was performed according to the manufacturer's instructions except for the dilution factor, which we allowed to be 4.6 times higher. Immediately after incubation, DNA extraction was performed with the MagAttract PowerMicrobiome DNA/RNA EP kit (Qiagen) according to the manufacturer's instructions. Quantification of DNA was performed using PicoGreen (Thermofisher) and measurements were performed with a plate reader (Tecan, Life Sciences) using 50 and 80 gain settings. All samples were diluted to 10 ng/μL.

Library preparation

Sample index barcode design using a previously introduced method [4] yielded a set of 96 x 8nt sequences with a 0.5 mean GC content and none of the barcodes containing 3 or more identical bases in a row. Nine hundred sixty different combinations of i5 and i7 primers were used to create a uniquely barcoded library for each sample. The detailed sample-to-barcode assignment is given in **Supplementary Table 3**. Library preparation was carried out using a modification of the Nextera Flex protocol to produce low bias, called Hackflex, that allows the production of low cost shotgun libraries [4]. For each sample, 10 nanograms of input gDNA in 10 ul ultrapure water (Invitrogen) was mixed with 10 ul of 1:50 diluted BLT beads, 25 ul of 2x laboratory-made tagmentation buffer 20 mM Tris (pH 7.6) (Chem-Supply), 20 mM MgCl (Sigma), and 50% (v/v) Dimethylformamide (DMF) (Sigma); the final volume for each tagmentation reaction was 45 ul. Following, 10 ul of 0.2% of sodium dodecyl sulphate (SDS; Sigma) was added into each sample to stop tagmentation. Beads were then washed three times using 100 ul of washing solution which was filtered prior to

use (0.22 µm MF-Millipore™ membrane). The washing solution consisted of 10% polyethylene glycol (PEG) 8000 (Sigma), 0.25M NaCl (Chem-Supply) in Tris-EDTA buffer (TE) (Sigma). Library amplification was carried out using the PrimeSTAR GXL DNA Polymerase kit (Takara), according to the manufacturer protocol. Each PCR reaction contained 10 ul of 5x GXL buffer, 4 ul of 25 mM dNTPs, 2 ul of PrimeStar GXL polymerase, and 19 ul of nuclease free water. The PCR mix was added into washed BLT beads. Then, 5 ul of each custom synthesized 96-well plate Illumina Adapter Oligos i5 and i7 (i7: IDT plate#: 11680765; i5: IDT plate#: 11680754) was added to a final concentration of 0.555 uM to each reaction. Each sample's PCR reaction had a final volume of 45 ul. The following conditions were used: 3 min at 68ºC, 3 min at 98ºC, 12 cycles of [45 sec at 98ºC – 30 sec at 62ºC – 2 min at 68ºC], 1 min at 68ºC and hold at 10ºC. Following the amplification step, samples were centrifuged at 280 x *g* for 1 min and stored between 1 and 5 days at 4°C.

Size selection and purification

Samples from the same 96-well plates were pooled into one tube by taking 5 µL from each library. This generated 10 pooled samples, one for each plate. A master pool was created by pooling 5 µL from the pool of each plate into a single pool. Forty microliters from each of the 10 plate pools and 40 µL from the master pool underwent library size selection and purification using equal volumes of SPRIselect beads (Beckman Coulter) and ultrapure water (Invitrogen). Sample cleaning with SPRI-beads was performed as described previously [4]. A purified master pool comprising samples from all plates, and purified pools of individual plates to check for plate-specific anomalies, were diluted to 4 nM and fragment size distribution was assessed using the High Sensitivity DNA kit on the Bioanalyzer (Agilent Technologies, USA).

Normalization and sequencing

The master pool was sequenced on an Illumina MiSeq v2 300 cycle nano flow cell (Illumina, USA). Read counts were obtained and used to normalise libraries. The liquid handling robot OT-One (Opentrons) was programmed to re-pool libraries based on read counts obtained from the previous MiSeq run. The code used to achieve the normalization is available through our Github repository.

The read count distribution after normalisation is displayed in **Supplementary Figure 2**. The normalized and purified pooled library was sequenced on an Illumina NovaSeq 6000 S4 flow cell at the Ramaciotti Centre for Genomics (Sydney, NSW, Australia), generating a total of 27 billion read pairs from 911 samples.

Sequence data processing

Adapter trimming (parameters: k=23 hdist=1 tpe tbo mink=11), PhiX DNA removal (parameters: k=31 hdist=1), and quality filtering (parameters: ftm=0 qtrim=r trimq=20), were performed using bbduk.sh (http://jgi.doe.gov/data-and-tools/bbtools; bbmap version 38.22). Piglet samples (*n*=825) had a median count of 32,949,208 clean paired reads (mean=35,557,149) (script: *readcounts.R*). Quality assessment of raw reads was carried out using FASTQC (http://www.bioinformatics.babraham.ac.uk/projects/fastqc/) and a combined report of all samples was obtained with MULTIQC [5]. The presence of PCR duplicates was assessed by feeding read pairs to dedupe.sh (http://jgi.doe.gov/data-and-tools/bbtools) (parameters: ac=f). Nextflow [6] (version 18.10.1) was used to manage processing of the data on the HPC.

Comparison of the expected and the observed taxonomic profile of the positive controls

All the mock community members, in seven of the eight technical replicates, were detected by MetaPhlAn2 (version 2.7.7) (**Supplementary Figure 3**). One sample failed to sequence, reporting zero counts for any species. The observed mean relative abundances were as follows: *B. subtilis* (mean±SD: 2.92±0.994), *E. cloacae* (mean±SD: 38.0±6.404), *E. faecium* (mean±SD: 0.97±0.081), *E.coli* (mean±SD: 10.12±1.480), *E.coli* unclassified (mean±SD: 7.83± 1.755), *P. aeruginosa* (mean±SD: 26.72±3.026), *S. aureus* (mean±SD: 9.90±3.613), *S. epidermidis* (mean±SD: 3.54±1.435). Isolate *E. cloacae* C15117, used in this study for the make-up of the mock community, was recently found to be most closely related to the *Enterobacter hormaechei* phylogenomic group C type strain DSM 16687 and therefore re-identified as *Enterobacter hormaechei* subsp. *oharae*[7] . For this reason, taxonomic assignment by MetaPhlAn2 attributed the reads to *E. cloacae* instead. The expected proportions of

the mock community members were derived from the CFU by the genome size. Based on the expected and the observed relative abundance, we found, with the exception of *S. aureus* (exp: 8.7% obs: 9.9%), three Gram positive members to be under-represented (*B. subtilis*: exp: 13.0% obs: 2.9%; *E. faecium:* exp: 7.7% obs: 1.0%; *S. epidermidis*: exp: 16.7% obs: 3.5%) and, with the exception of *P. aeruginosa* (exp: 38.9% obs: 26.8%), two Gram negative members to be over-represented (*E. cloacae:* exp: 14.5% obs: 38.0%; *E.coli*: exp: 0.4% obs: 7.8-10.1%) (**Supplementary Figure 4**). Taxonomic assignment of the mock community samples reported one contaminating species in one of the eight replicates: *Lactobacillus salivarius* (mean: 0.008) (**Supplementary Figure 5**).

The probiotic D-Scour™ is expected to contain, per gram, a total of 180 million CFU of *Lactobacillus acidophilus*, *Lactobacillus delbrueckii* subspecies *bulgaricus*, *Lactobacillus plantarum*, *Lactobacillus rhamnosus*, *Bifidobacterium bifidum*, *Enterococcus faecium*, *Streptococcus salivarius* subspecies *thermophilus* in unknown proportions. From taxonomic analysis with MetaPhlAn2, we can conclude that 6 of the 7 expected species were determined to be present in the replicates in the following mean relative abundances: *Bifidobacterium bifidum*: mean±SD: 40.01±12.558; *Enterococcus faecium*: mean±SD: 30.98±13.472; *Lactobacillus delbrueckii*: mean±SD: 11.56±7.148; *Lactobacillus plantarum*: mean±SD: 6.23±7.863; *Lactobacillus rhamnosus*: mean±SD: 2.08±1.226; *Streptococcus thermophilus*: mean±SD: 4.28±1.523. *Lactobacillus acidophilus* was not detected and *Lactobacillus helveticus* was detected instead (*Lactobacillus helveticus*: mean±SD: 4.75±2.431) (**Supplementary Figure 3**). An additional 25 taxa were detected, of which 18 and 7 were identified at the species and at the genus level, respectively. Contaminants were present at a higher concentration in three technical replicates (R3, R7, R8) with the most frequent contaminant (*Methanobrevibacter* spp.) being present in 5 of the 8 replicates (**Supplementary Figure 5**).

Taxonomic analysis of the technical replicates of the probiotic ColiGuard® also showed a species profile consistent with the expected profile, with *Lactobacillus salivarius* and *Lactobacillus plantarum* in a 9:1 ratio (*Lactobacillus salivarius*: mean±SD: 93.52±1.617; *Lactobacillus plantarum*: mean±SD: 6.10±1.134) across the replicates (**Supplementary Figure 3**). ColiGuard® contained a total of 20

contaminants, of which 16 and 4 were identified at the species and the genus level, respectively. Contaminants were present at a higher level in two technical replicates (R5, R7), with R7 displaying the most diverse and highest contamination rate (R7: 14 taxa; total contaminating reads: 2.67%; R5: 9 taxa; total contaminating reads: 0.30%). (**Supplementary Figure 5**).

Technical controls in metagenomic studies and methodological limitations

Taxonomic assignment of the raw reads from the positive controls was performed with MetaPhlAn2 [8] which relies on a ca. 1M unique clade-specific markers derived from 17,000 reference genomes. Such a database to map against the positive controls suffices as these organisms are cultivable, and for this reason they are widely studied hence the sequences are known. This is not the case for real-world samples where mapping against a database (the completeness of which relies on studied and often cultivable organisms) would narrow the view on the true diversity within the sample.

Positive controls with well-studied members and known ratios within the samples have proven to be a valuable approach to assess consistency among technical replicates across batches and to detect possible biases derived from the DNA extraction method. Systematic taxonomic bias in microbiome studies, resulting from differences in cell wall structures between Gram positive and Gram negative bacteria, have previously been reported; bead beating and sample treatment with enzymatic cocktails can modestly reduce this bias [9–12]. Although we implemented such steps in our workflow, it seems that, from the read abundance of our mock community, which contained three Gram negative and four Gram positive strains, a bias towards Gram negative taxa may still be present. Knudsen *et al* (2016) compared various DNA isolation methods with distinct sample types and reported a reduced bias when using an adapted version of the QIAamp Fast DNA Stool Mini Kit (Qiagen) [12].

In terms of contamination we concluded that: a) contamination in our study was not batch specific; b) a problem of sample cross-contamination may have occurred at the DNA extraction step between neighbouring wells. During the bead-beating step of DNA extraction, the deep-well plate is sealed with a rubber sealing mat, rotated and placed in a plate shaker for the bead beating to take place. As leakage was observed

around the wells despite the presence of the sealing mat, we consider that sample cross-contamination is most likely to occur during this step.

Taxonomic profiling of samples

All raw reads were analysed with SortMeRNA [1] (version 4.0.0) to extract reads containing 16S rRNA genes. Extraction was performed by mapping reads against the silva-bac-16s-id90.fasta database with –fastx --blast 1 –num_alignments 1 parameters settings (script: *sortmerna.sh*). Over 60 million reads (*n*=60,584,650) contained 16S rRNA genes passing the E-value threshold for filtering (E-value ≤ 0.0001). These reads occupy between 36.4% and 37.1% of each sample (script: *sortmerna_counts.R*). Reads were further filtered based on E-value cutoff (E-value ≤ $1x10^{-30}$), sequence identity (identity ≥ 80%), and alignment length (length ≥ 100 bp). Over half of the reads (*n*=32,419,310) passed the filtering (script: *sortmerna_filter.sh*) and were classified using the RDP classifier [2] (version 2.13), a naïve Bayesian classifier, that classifies 16S rRNA sequences into the new higher-order taxonomy proposed in Bergey's Taxonomic Outline of the Prokaryotes (2nd ed., release 5.0, Springer-Verlag, New York, NY, 2004) (script: *RDP_Krona.sh*). Most abundant Phyla in the piglet population (*n*=126) were: *Firmicutes* (75.14%), *Bacteroidetes* (13.70%), *Actinobacteria* (5.31%), *Proteobacteria* (3.17%), *Spirochaetes* (0.72%), *Synergistetes* (0.40%). Most abundant Phyla in the mothers (*n*=42) were: *Firmicutes* (84.75%), *Proteobacteria* (6.30%), *Bacteroidetes* (5.44%), *Actinobacteria* (1.85%), *Verrucomicrobia* (0.36%), *Synergistetes* (0.23%). The RDP classifier estimates the confidence of an assignment using the number of times a genus is selected out of 100 bootstrap trials. Assignments at the phylum level had a mean confidence of 0.93 (scale 0-1; median=1.00) (script: *RDP_analyze.R*). Classifications were displayed using Krona [3] (version 2.7.1).

Potential uses

This dataset can be utilised to assess a broad range of ecological questions pertaining to host-associated microbial communities of the post-weaning piglet. These include the assessment of: 1. the compositional and functional core faecal microbiome of the post-weaning piglet, 2. the microbial changes that piglets undergo between the first and the

5th week after weaning, 3. the degree of strain-host specificity, 4. the variability of microbiomes within or between host species, 5. the variability of microbiomes between different cross-breeds and small age differences of the hosts, 6. the degree of strain transfer from mothers to piglets, 7. the effects of two probiotic treatments and of intramuscular antibiotic treatment on the post-weaning pig faecal microbiome, 8. species co-occurrence and co-exclusion, 9. the repertoire of antimicrobial resistance genes and how it is impacted by antibiotic and probiotic treatment, 10. the extent of within-host and population evolution of microbes over a 5-week period.

Data availability

The sequencing reads from each sequencing library have been deposited at NCBI Short Read Archive under project PRJNA526405. All supplementary figures and tables are provided as additional files. The scripts for the automated robot pooling (*robot_pooling.py*), for the sequence data processing (*initial.nf*), and for the data analysis can be found in our Github repository (https://github.com/GaioTransposon/metapigs_base; tag: GigaScience). For the data analysis scripts, the R language (version 3.6.3) and the following packages were used: readr (v1.4), readxl (v1.3.1), tidyr (v1.1.2), tidyverse (v1.3.0), ggplot2 (v3.3.3), dplyr (v1.0.3), gridExtra (v2.3), pheatmap (v1.0.12), cowplot (v1.1.1), splitstackshape (v1.4.8).

Figures

Figure 1. Taxonomic profile of the porcine microbiome.

Microbial taxonomic composition of post-weaning piglets (**A**) and their mothers (**B**). Taxonomic profiling is based on the analysis of reads containing bacterial 16S rRNA genes extracted from shotgun metagenomic data. Plots were generated using Krona [3], which displays hierarchically organized nodes of the taxonomic tree based on their relative abundance. Distinct colours represent separate domains of life.

Figure 2. Timeline.

Timeline of the animal trial indicating the start and the length of the treatment for each cohort, the sample collection points, and the piglets' age during the trial. Piglets were allowed 2 days of acclimatisation after the arrival on the site of the trial and before the start of the treatments (pink: placebo paste; yellow: probiotic D-Scour™ formulation; green: probiotic ColiGuard® formulation; blue: antibiotic neomycin intramuscular injection). Large triangles (dark) indicate main days of sampling where all piglets were sampled (*n*=126). Small triangles (light) indicate sampling points from a subset of the piglets (8 per cohort; *n*=48).

Figure 3. Workflow.

A schematic representation of the experimental workflow from sample collection (yellow), through sample processing and sequencing (orange), to the preliminary data analysis (blue).

Additional files

Supplementary Figure 1: Piglets placements across rooms and pens.

Supplementary Figure 2: Read count distribution.

Supplementary Figure 3: Taxonomic assignment of reads from positive control samples.

Supplementary Figure 4: Expected and observed relative abundance of mock community members.

Supplementary Figure 5: Heatmap reporting the contaminating species found within the technical replicates of the positive controls.

Supplementary Table 1: Animal details**.**

Supplementary Table 2: Metadata.

Supplementary Table 3: Barcodes.

Funding information

This work was supported by the Australian Research Council, linkage grant LP150100912. This project was funded by the Australian Centre for Genomic Epidemiological Microbiology (Ausgem), a collaborative partnership between the

NSW Department of Primary Industries and the University of Technology Sydney. TZ and DG are recipients of UTS International Research and UTS President's Scholarships. NSW DPI approved the paper before submission for publication.

Competing interests

D-Scour™ was sourced from International Animal Health Products (IAHP). ColiGuard® was developed in a research project with NSW DPI, IAHP and AusIndustry Commonwealth government funding.

Author contributions

Pig Trial: TC, LF, DG, TZ, GJE, AED, SPD

DNA extraction: DG, ML

Library prep, robot pooling: DG, ML, KA, AED

Sequencing data processing: MZD, DG, AED

Data analysis: DG, AED

Manuscript: DG

Manuscript editing: DG, AED, GJE, John Webster

Acknowledgements

We would like to thank Shayne Fell for the on site help in the pig trial. Thank you Amy Bottomley, Giulia Ballerin, Rosy Cavaliere, for providing the mock community strains. Thank you Akane Tanaka, Leigh Monahan and Joyce To for the technical support. Thank you John Webster for providing DPI internal review of this manuscript and for the helpful suggestion about its content. Thanks to International Animal Health Products for providing access to the probiotic supplements and to the Australian Centre for Genomic Epidemiological Microbiology (Ausgem) for financially supporting this study.

REFERENCES

1. Kopylova, E., Noé, L. & Touzet, H. SortMeRNA: fast and accurate filtering of ribosomal RNAs in metatranscriptomic data. *Bioinformatics* **28**, 3211–3217 (2012).
2. Wang, Q., Garrity, G. M., Tiedje, J. M. & Cole, J. R. Naive Bayesian classifier for rapid assignment of rRNA sequences into the new bacterial taxonomy. *Appl. Environ. Microbiol.* **73**, 5261–5267 (2007).
3. Ondov, B. D., Bergman, N. H. & Phillippy, A. M. Interactive metagenomic visualization in a Web browser. *BMC Bioinformatics* **12**, 1–10 (2011).
4. Gaio, D. *et al.* Hackflex: low cost Illumina sequencing library construction for high sample counts. *bioRxiv* 779215 (2019).
5. Ewels, P., Magnusson, M., Lundin, S. & Käller, M. MultiQC: summarize analysis results for multiple tools and samples in a single report. *Bioinformatics* **32**, 3047–3048 (2016).
6. Ewels, P. A. *et al.* nf-core *: Community curated bioinformatics pipelines*. (2019).
7. Monahan, L. G. *et al.* High contiguity genome sequence of a multidrug-resistant hospital isolate of Enterobacter hormaechei. *Gut Pathog.* **11**, 3 (2019).
8. Truong, D. T. *et al.* MetaPhlAn2 for enhanced metagenomic taxonomic profiling. *Nat. Methods* **12**, 902–903 (2015).
9. von Wintzingerode, F., Göbel, U. B. & Stackebrandt, E. Determination of microbial diversity in environmental samples: pitfalls of PCR-based rRNA analysis. *FEMS Microbiol. Rev.* **21**, 213–229 (1997).

10. Han, Z., Sun, J., Lv, A. & Wang, A. Biases from different DNA extraction methods in intestine microbiome research based on 16S rDNA sequencing: a case in the koi carp, Cyprinus carpio var. Koi. *MicrobiologyOpen* **8**, e00626 (2019).
11. Moss, E. L., Maghini, D. G. & Bhatt, A. S. Complete, closed bacterial genomes from microbiomes using nanopore sequencing. *Nat. Biotechnol.* (2020) doi:10.1038/s41587-020-0422-6.
12. Knudsen, B. E. *et al.* Impact of sample type and DNA isolation procedure on genomic inference of microbiome composition. *MSystems* **1**, (2016).

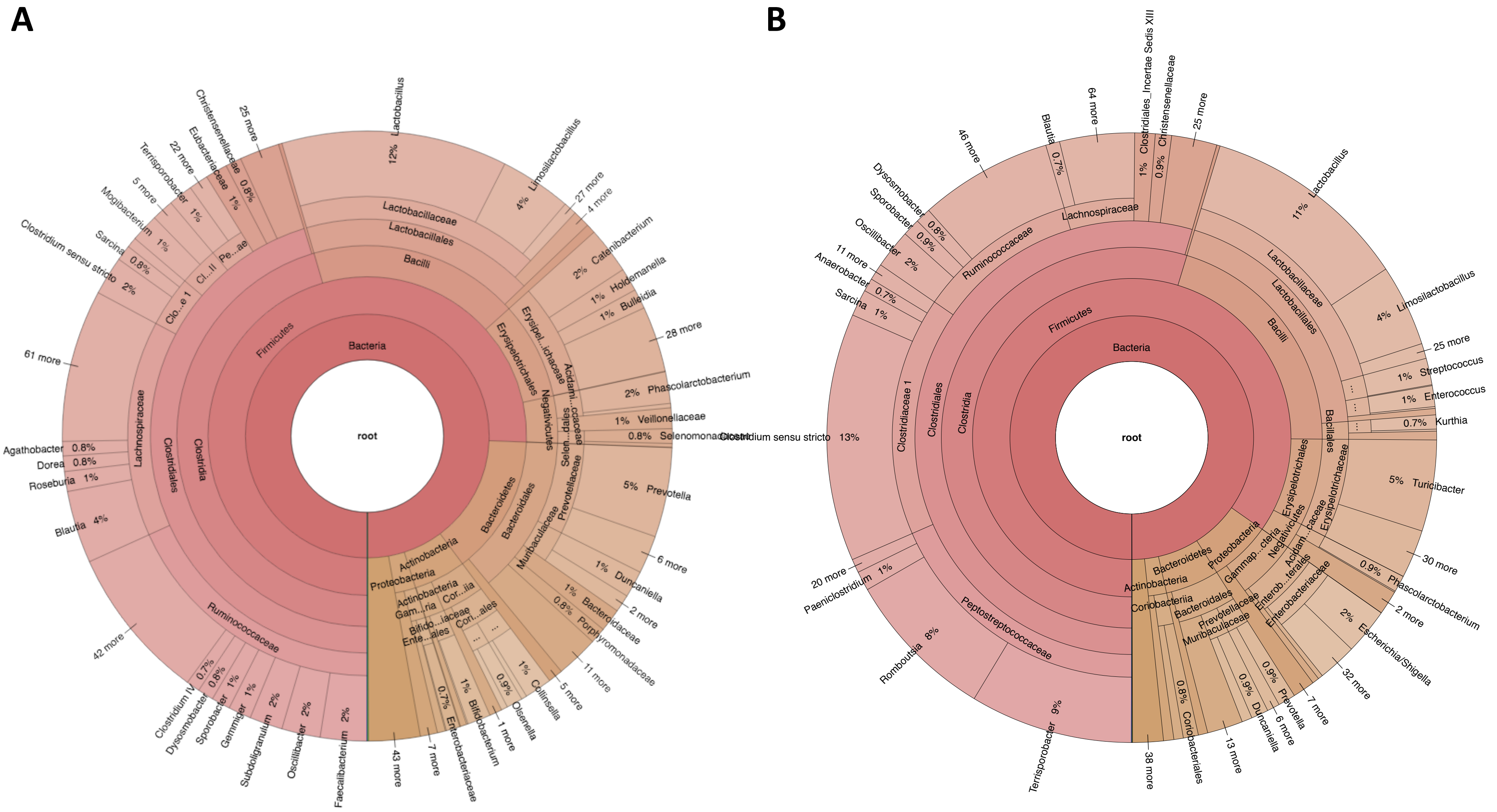
A
B
root
Bacteria
Firmicutes
Clostridia
Clostridiales
Lachnospiraceae
Ruminococcaceae
Bacilli
Lactobacillales
Lactobacillaceae
Lactobacillus 12%
Limosilactobacillus 4%
Bacteroidetes
Bacteroidales
Prevotellaceae
Prevotella 5%
Blautia 4%
Proteobacteria
Actinobacteria
Clostridium sensu stricto 13%
Peptostreptococcaceae
Terrisporobacter 9%
Romboutsia 8%
Lactobacillus 11%
Turicibacter 5%
Escherichia/Shigella 2%

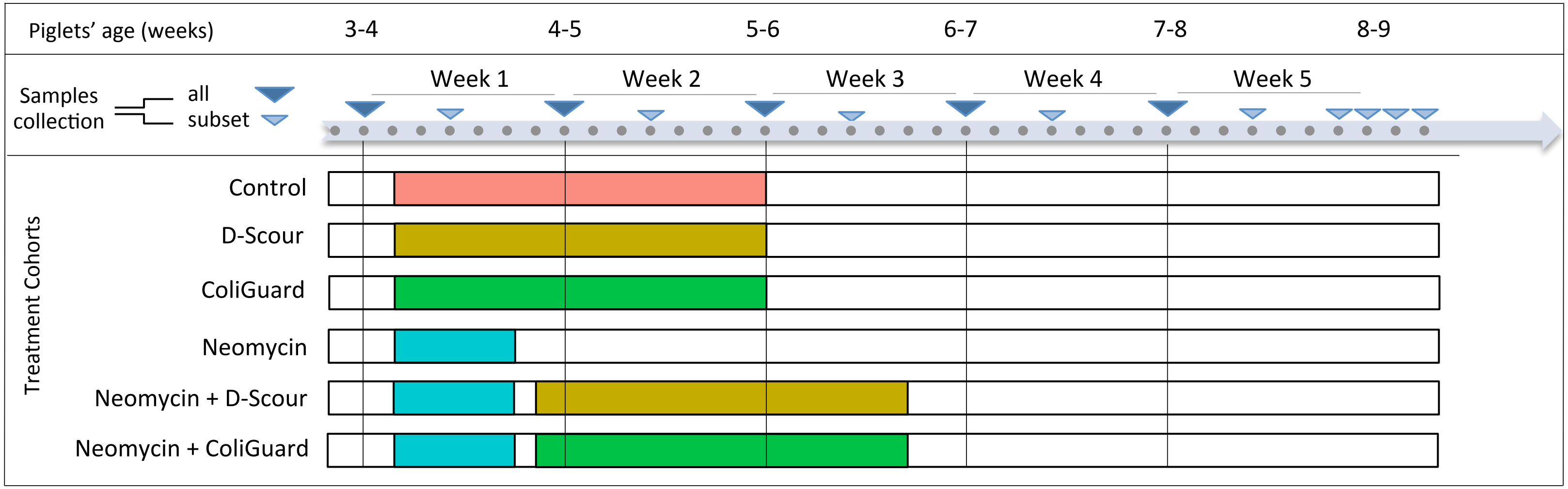

Piglets' age (weeks)
3-4
4-5
5-6
6-7
7-8
8-9
Samples collection
all
subset
Week 1
Week 2
Week 3
Week 4
Week 5
Treatment Cohorts
Control
D-Scour
ColiGuard
Neomycin
Neomycin + D-Scour
Neomycin + ColiGuard

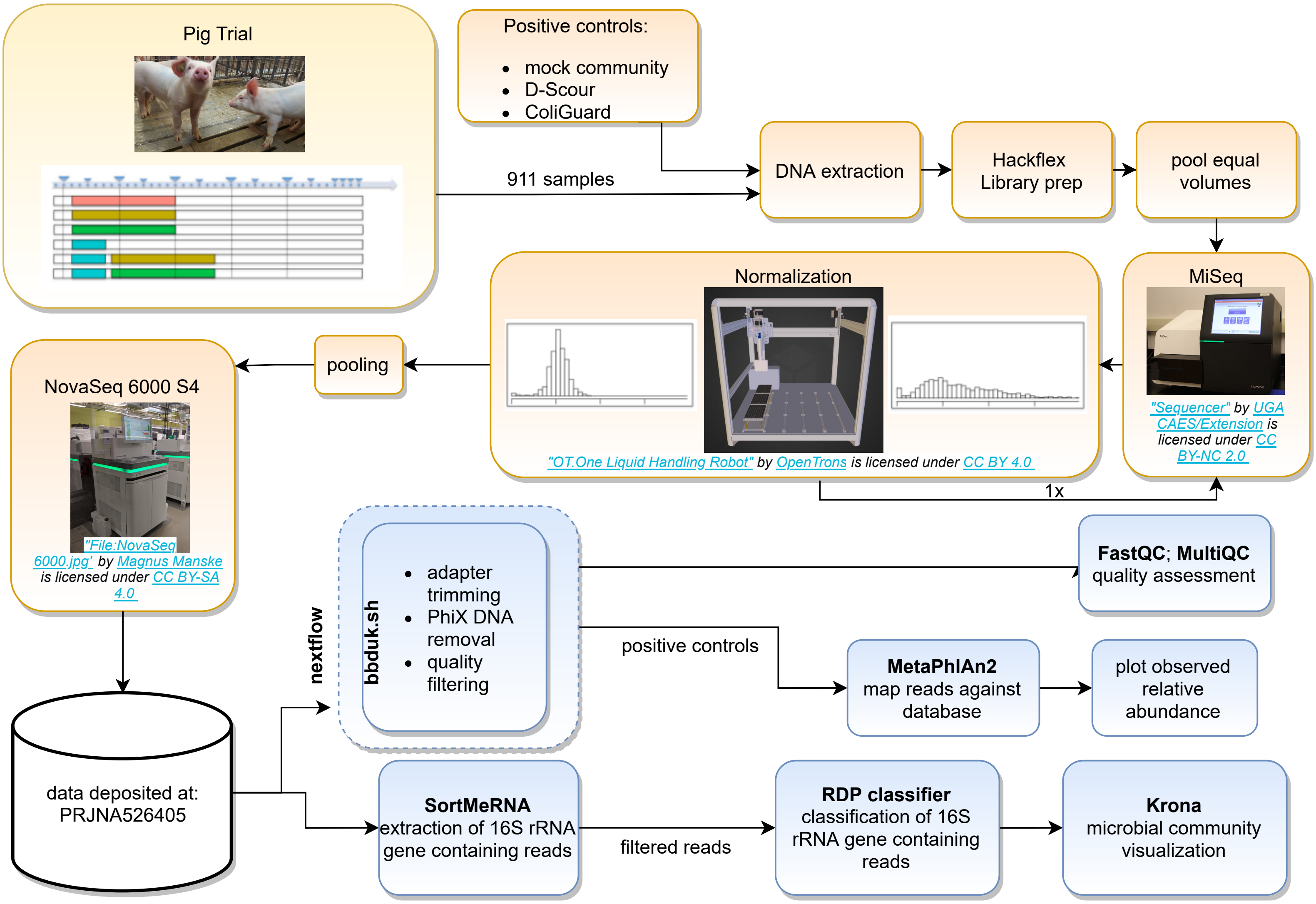

Pig Trial
Positive controls:
mock community
D-Scour
ColiGuard
911 samples
DNA extraction
Hackflex Library prep
pool equal volumes
MiSeq
"Sequencer" by UGA CAES/Extension is licensed under CC BY-NC 2.0
Normalization
"OT.One Liquid Handling Robot" by OpenTrons is licensed under CC BY 4.0
1x
pooling
NovaSeq 6000 S4
"File:NovaSeq 6000.jpg" by Magnus Manske is licensed under CC BY-SA 4.0
data deposited at: PRJNA526405
nextflow
bbduk.sh
adapter trimming
PhiX DNA removal
quality filtering
FastQC; MultiQC quality assessment
positive controls
MetaPhlAn2 map reads against database
plot observed relative abundance
SortMeRNA extraction of 16S rRNA gene containing reads
filtered reads
RDP classifier classification of 16S rRNA gene containing reads
Krona microbial community visualization

Supplementary Figures

Click here to access/download
Supplementary Material
metapigs_base_DataNote_SupplFigures.pdf

Supplementary Table 1

Click here to access/download
Supplementary Material
metapigs_base_DataNote_SupplTable1.xlsx

Supplementary Table 2

Click here to access/download
Supplementary Material
metapigs_base_DataNote_SupplTable2.xlsx

Supplementary Table 3

Click here to access/download
Supplementary Material
metapigs_base_DataNote_SupplTable3.csv
