# Supplementary material for: A large-scale metagenomic survey dataset of the post-weaning piglet gut lumen
Source: Gigascience. 2021 Jun 3;10(6):giab039. doi: 10.1093/gigascience/giab039 (PMC8173662; doi:10.1093/gigascience/giab039)
Supplement: giab039_GIGA-D-20-00347_Revision_2 [file giab039_giga-d-20-00347_revision_2.pdf]

GigaScience

A large-scale metagenomic survey dataset of the post-weaning piglet gut lumen

--Manuscript Draft--

**Manuscript Number:**	GIGA-D-20-00347R2
**Full Title:**	A large-scale metagenomic survey dataset of the post-weaning piglet gut lumen
**Article Type:**	Data Note
**Funding Information:**	Australian Research Council (LP150100912) — Dr Steven P Djordjevic
**Abstract:**	We report on the largest shotgun metagenomic analysis of the pig gut lumen microbiome to date. By processing over 800 faecal time-series samples from 126 piglets and 42 sows, we generated over 8Tbp of metagenomic shotgun sequence data. This study was conducted to generate a publicly available databank of the faecal metagenome of weaner piglets aged between 3 and 9 weeks old, treated with different probiotic formulations and intramuscular antibiotic treatment. Here we describe the animal trial procedures and the generation of our metagenomic dataset.
**Corresponding Author:**	Aaron Darling<br>University of Technology Sydney<br>Ultimo, AUSTRALIA
**Corresponding Author Secondary Information:**	
**Corresponding Author's Institution:**	University of Technology Sydney
**Corresponding Author's Secondary Institution:**	
**First Author:**	Daniela Gaio
**First Author Secondary Information:**	
**Order of Authors:**	Daniela Gaio
	Matthew Z DeMaere
	Kay Anantanawat
	Graeme J Eamens
	Michael Liu
	Tiziana Zingali
	Linda Falconer
	Toni A Chapman
	Steven P Djordjevic
	Aaron E Darling
**Order of Authors Secondary Information:**	
**Response to Reviewers:**	Reviewer reports:<br>Reviewer #2: The authors have revised their Research paper into a Data Note. Most of the initially raised concerns on data analysis are now redundant and consequently removed from the manuscript. Please note that this will not invalidate those comments for future publications.<br><br>RE: Thank you, we will indeed continue to revise and eventually resubmit that part of the work elsewhere as a separate manuscript. Your comments have already led to great improvements and we intend to acknowledge that in the "Acknowledgements" section of the next manuscript.<br><br>Main focus of the data note is/should be such that other researchers reaching out to

	this dataset can find all relevant details on experimental design, experimental approach and primary data processing in this manuscript.<br><br>- The authors have removed most of the concerning analysis details.<br><br>Few comments;<br>1) Data description / OTUs. Are OTUs used or "ASVs". I understand sortMeRNA was used that includes qiime v1.x but current methods use a DaDa2 similar approach moving away from OTUs (as is qiime v2.x for over a long period already)?<br><br>RE: Although we are aware of the advances that ASV inference methods have led to in the analysis of 16S amplicon sequencing data, there are no rigorous performant methods, that we are aware of, to obtain ASVs from shotgun metagenomic reads. We therefore left the existing analysis unchanged.<br><br>2) Data description: to largely describe (overall picture) a simple alpha-diversity plot and beta-diversity ordination plot would be an easy/quick way that brings more meaningful insight in describing the overall samples and individual variation observed. Now all data of the various groups is averaged per piglet or mother in a bardiagram/Krona plot which does not make sense since there is a large diversity within those groups of treatments.<br><br>RE: With regards to the diversity, an analysis of diversity (included in our first submission) suggested that differences in microbial community composition (alpha and beta diversity) between treatment groups was mild, while more prominent shifts of diversity were detected between samples from distinct time points (age of the piglets). The revised manuscript now includes a PCoA to describe the beta diversity of samples. We removed the Krona plot of the mothers, and merged the Krona plot of the piglets to a panel in a combined figure with the PCoA (beta diversity). The PCoA plots highlight the strong effect of time/aging, and the (dis)similarity between the mothers and piglets at distinct time points in the trial. In the revised submission we also report alpha diversity indices.<br><br>3) Supplement Fig2; make more clear that frequency is N samples. Also add bin size in legend for both sub figures.<br><br>RE: Done.<br><br>4) Supplement Fig4; would this be a possible result of using cfu which does not take into account dead/alive ratio's? That discussion/mentioning seems missing in the current text.<br><br>RE: Yes, that is true. This has now been added to the manuscript.
**Additional Information:**	
**Question**	**Response**
Are you submitting this manuscript to a special series or article collection?	No
**Experimental design and statistics**<br><br>Full details of the experimental design and statistical methods used should be given in the Methods section, as detailed in our Minimum Standards Reporting Checklist. Information essential to interpreting the data presented should be made available in the figure legends.	Yes

Powered by Editorial Manager® and ProduXion Manager® from Aries Systems Corporation

Have you included all the information requested in your manuscript?	
**Resources**<br><br>A description of all resources used, including antibodies, cell lines, animals and software tools, with enough information to allow them to be uniquely identified, should be included in the Methods section. Authors are strongly encouraged to cite Research Resource Identifiers (RRIDs) for antibodies, model organisms and tools, where possible.<br><br>Have you included the information requested as detailed in our Minimum Standards Reporting Checklist?	Yes
**Availability of data and materials**<br><br>All datasets and code on which the conclusions of the paper rely must be either included in your submission or deposited in publicly available repositories (where available and ethically appropriate), referencing such data using a unique identifier in the references and in the “Availability of Data and Materials” section of your manuscript.<br><br>Have you have met the above requirement as detailed in our Minimum Standards Reporting Checklist?	Yes

Manuscript
Click here to access/download;Manuscript;metapigs_base_DataNote_ms.do

A large-scale metagenomic survey dataset of the post-weaning piglet gut lumen

Abstract

We report on the largest shotgun metagenomic analysis of the pig gut lumen microbiome to date. By processing over 800 faecal time-series samples from 126 piglets and 42 sows, we generated over 8Tbp of metagenomic shotgun sequence data. This study was conducted to generate a publicly available databank of the faecal metagenome of weaner piglets aged between 3 and 9 weeks old, treated with different probiotic formulations and intramuscular antibiotic treatment. Here we describe the animal trial procedures and the generation of our metagenomic dataset.

Data description

The dataset includes 911 samples, comprising a total of 27 billion raw sequence reads. Preliminary analysis of the dataset consisted in the extraction of 16S rRNA gene containing reads with SortMeRNA [1] and their classification with the RDP classifier [2]. In terms of taxonomic diversity, most OTUs (75.71%) were assigned to the *Firmicutes* phylum. The next most abundant bacterial phyla were: *Bacteroidetes* (13.21%), *Actinobacteria* (5.10%), *Proteobacteria* (3.36%), and *Spirochates* (0.69%). A visualization of the microbial composition, obtained with Krona [3], is shown of the post-weaning piglets (**Figure 1A**) along with the beta diversity of the mothers and the piglets during the first 5 weeks post-weaning (**Figure 1B**). Interactive Krona maps are

available as html files in our Github repository (https://github.com/GaioTransposon/metapigs_base).

Pig trial and sample collection

Animal studies were conducted at the Elizabeth Macarthur Agricultural Institute (EMAI) NSW, Australia and were approved by the EMAI Ethics Committee (Approval M16/04). The trial animals comprised 4-week old male weaner pigs (*n*=126) derived from a commercial swine farm and transferred to the study facility in January 2017. These were cross-bred animals of "Landrace", "Duroc" and "Large White" breeds and had been weaned at approximately 3 weeks of age (**Supplementary Table 1**).

The pig facility consisted of four environmentally controlled rooms (Rooms 1-4) with air conditioning, concrete slatted block flooring with underground drainage and open rung steel pens (**Supplementary Figure 1**). Each room had nine pens, consisting of a set of six and a set of three pens, designated a-f and g-i respectively, with the two sets of pens being physically separate, *i.e.* animals could come in contact with each other through the pen's bars within each set of pens, but not between sets. The rooms were physically separated by concrete walls and contamination between rooms was minimized by using separate equipment (boots, gloves, coveralls) for each room. In addition, under-floor drainage was flushed twice weekly and the flushed faeces/urine was retained in under-floor channels that ran the length of the facility, so that Rooms 1, 2 were separate from Rooms 3, 4 and flushing was in the direction 1 to 2 and 3 to 4.

The pigs were fed *ad libitum* a commercial pig grower mix of 17.95% protein free of antibiotics, via self-feeders. On the day of arrival (day 1) 30, 18, 18, and 60 pigs were allocated randomly to Rooms 1, 2, 3 and 4 respectively in groups of 6, 6, 6 and 6-7 pigs per pen respectively (**Supplementary Figure 1A**). Pigs were initially weighed on day 2, and some pigs were moved between pens to achieve an initial mean pig weight per treatment of approximately 6.5 kg (range: 6.48-6.70; mean±SD: 6.53±0.08). Pigs were weighed weekly throughout the trial, and behaviour and faecal consistency scores were taken daily over the 6-week period of the trial (**Supplementary Table 2**). Developmental and commercial probiotic paste

preparations ColiGuard® and D-Scour™ from International Animal Health, were used in some treatment groups.

The animals were acclimatised for 2 days before the following treatments were administered: Room 1 - oral 1 g/pig of placebo paste daily for 14 d; Room 2 - oral 1 g/pig of D-Scour™ paste daily for 14 d; Room 3 - oral 1 g/pig of ColiGuard® paste daily for 14 d; Room 4 - intramuscular (IM) injection of antibiotic administered at 0.1 mL per pig daily from a 200 mg/mL solution for a total treatment duration of 5 d.

On the day following the final neomycin treatment (day 8), 36 pigs were moved from Room 4 to Room 2 (*n*=18, 6 in each pen, pens g-i), and to Room 3 (*n*=18, 6 in each pen, pens g-i) (**Supplementary Figure 1B**). The following day (day 9), oral administration of D-Scour™ (1 g/pig) and of ColiGuard® (1 g/pig) commenced for pigs in Room 2 pens g-i and in Room 3 pens g-i, respectively, and continued for a period of 14 days. Assignment of the 36 neomycin-treated pigs to the treatment groups neomycin+D-Scour™ (*n*=18; Room 2 pens g-i) and neomycin+ColiGuard® (*n*=18; Room 3 pens g-i), was carried out by distributing them so that the mean weight of the animals distributed across pens and rooms was similar. By this time point, each occupied pen in the trial housed six pigs. (**Supplementary Figure 1B**) From that time, twelve piglets from the original 126 were no longer present, as they had been euthanised as pre-treatment controls at the start of the trial.

Faecal samples were collected from all piglets once per week and from a subset (*n*=48 pigs; 8 from each of the six cohorts) twice per week over the 6-week study period (**Figure 2**). From each piglet, faeces were collected per rectum with new disposable gloves; where minimal or no faeces could be collected on a collection day, sampling was performed the following morning. Samples were placed in 50 mL Falcon tubes and stored at 4°C within 30 mins of sample collection for a minimum of 30 mins and a maximum period of 6 h.

Faecal sample processing

Samples (3g/pig) were mixed with 15 mL PBS (200 mg/mL), in sterile stomacher bags and homogenized with a Bio-Rad stomacher. The homogenised samples were divided in replicates: one replicate was stored directly at -80°C and one replicate was supplemented with glycerol (20% v/v) (Sigma-Aldrich) then stored at -80° C. In addition, single time-point faecal samples from the dams of the trial pigs (*n*=42) were

obtained from the commercial facility of origin and were pre-processed at EMAI as described above. Thus, a total of 911 unique samples, between one and ten samples per subject (mean: 4.8; median: 3) (**Supplementary Table 2**), were obtained throughout this study. At the end of the trial period, all samples were transported from EMAI to the University of Technology Sydney (UTS) for further processing. The experimental workflow is schematically represented in **Figure 3**.

Positive controls

As a positive control "mock community" for this study, four Gram positive (*Bacillus subtilis* strain 168, *Enterococcus faecium*, *Staphylococcus aureus* ATCC25923, *Staphylococcus epidermidis* ATCC35983) and three Gram negative (*Enterobacter hormaechei* CP_032842, *Escherchia coli* K-12 MG1655, *Pseudomonas aeruginosa* PAO1) bacterial strains from -80°C stocks were cultured at 37°C for 16 h in LB (Luria-Bertani) then centrifuged at 14,000 rpm for 10 mins. From the resulting pellets, 1 g was transferred to 1 mL of LB and homogenised and a 1:10 dilution of this was made for each bacterial culture. Ten microliters of bacterial suspension from each of the cultures was used to determine the number of colony forming units (CFU) in the original suspension in the following manner: by further diluting tenfold in LB and by plating onto 1.6% LB agar plates and incubated overnight. The remaining suspensions (990 µL from each bacterial culture) were pooled into a sterile tube, then aliquoted into Eppendorf tubes in 500 µL volumes/tube. As a washing step, Eppendorf tubes were centrifuged at 14,000 rpm for 10 mins, 500 µL PBS was added to the pellet and subsequently resuspended. These tubes constituted the mock community samples and were stored at -80°C. Expected proportions of the mock community members were determined from the estimated colony forming units (CFU) multiplied by the genome size and were as follows: 8.7:13.0:7.7:16.7:38.9:14.5:0.4 for *S. aureus*, *B. subtilis*, *E. faecium, S. epidermidis*, *P. aeruginosa*, *E. cloacae, and E.coli* respectively.

The two probiotic formulations used in this study were used as two additional positive controls. D-Scour™ is a commercially available probiotic formulation for livestock, with each gram containing 180 million CFU of the following: *Lactobacillus acidophilus*, *Lactobacillus delbrueckii* subspecies *bulgaricus*, *Lactobacillus plantarum*, *Lactobacillus rhamnosus*, *Bifidobacterium bifidum*, *Enterococcus faecium*,

Streptococcus salivarius subspecies *thermophilus*, with an additional 20 mg of garlic extract (*Allium sativum*). The probiotic ColiGuard is a probiotic formulation developed for the treatment of entero-toxigenic *Escherichia coli* (ETEC) in weaner pigs, developed in collaboration between the NSW DPI and International Animal Health Products, containing undefined concentrations of *Lactobacillus plantarum* and *Lactobacillus salivarius*.

DNA extraction

Piglet and sow faecal samples, mock community samples, negative controls and probiotic samples (D-Scour™ and ColiGuard® paste) were allocated to a randomized block design to control for batch effects in DNA extraction and library preparation. The faecal samples were thawed on ice first, followed by the probiotics and mock community samples. MetaPolyzyme (Sigma-Aldrich) treatment was performed according to the manufacturer's instructions except for the dilution factor, which we allowed to be 4.6 times higher. Immediately after incubation, DNA extraction was performed with the MagAttract PowerMicrobiome DNA/RNA EP kit (Qiagen) according to the manufacturer's instructions. Quantification of DNA was performed using PicoGreen (Thermofisher) and measurements were performed with a plate reader (Tecan, Life Sciences) using 50 and 80 gain settings. All samples were diluted to 10 ng/μL.

Library preparation

Sample index barcode design using a previously introduced method [4] yielded a set of 96 x 8nt sequences with a 0.5 mean GC content and none of the barcodes containing 3 or more identical bases in a row. Nine hundred sixty different combinations of i5 and i7 primers were used to create a uniquely barcoded library for each sample. The detailed sample-to-barcode assignment is given in **Supplementary Table 3**. Library preparation was carried out using a modification of the Nextera Flex protocol to produce low bias, called Hackflex, that allows the production of low cost shotgun libraries [4]. For each sample, 10 nanograms of input gDNA in 10 ul ultrapure water (Invitrogen) was mixed with 10 ul of 1:50 diluted BLT beads, 25 ul of 2x laboratory-made tagmentation buffer 20 mM Tris (pH 7.6) (Chem-Supply), 20 mM MgCl

(Sigma), and 50% (v/v) Dimethylformamide (DMF) (Sigma); the final volume for each tagmentation reaction was 45 ul. Following, 10 ul of 0.2% of sodium dodecyl sulphate (SDS; Sigma) was added into each sample to stop tagmentation. Beads were then washed three times using 100 ul of washing solution which was filtered prior to use (0.22 µm MF-Millipore™ membrane). The washing solution consisted of 10% polyethylene glycol (PEG) 8000 (Sigma), 0.25M NaCl (Chem-Supply) in Tris-EDTA buffer (TE) (Sigma). Library amplification was carried out using the PrimeSTAR GXL DNA Polymerase kit (Takara), according to the manufacturer protocol. Each PCR reaction contained 10 ul of 5x GXL buffer, 4 ul of 25 mM dNTPs, 2 ul of PrimeStar GXL polymerase, and 19 ul of nuclease free water. The PCR mix was added into washed BLT beads. Then, 5 ul of each custom synthesized 96-well plate Illumina Adapter Oligos i5 and i7 (i7: IDT plate#: 11680765; i5: IDT plate#: 11680754) was added to a final concentration of 0.555 uM to each reaction. Each sample's PCR reaction had a final volume of 45 ul. The following conditions were used: 3 min at 68ºC, 3 min at 98ºC, 12 cycles of [45 sec at 98ºC – 30 sec at 62ºC – 2 min at 68ºC], 1 min at 68ºC and hold at 10ºC. Following the amplification step, samples were centrifuged at 280 x *g* for 1 min and stored between 1 and 5 days at 4°C.

Size selection and purification

Samples from the same 96-well plates were pooled into one tube by taking 5 µL from each library. This generated 10 pooled samples, one for each plate. A master pool was created by pooling 5 µL from the pool of each plate into a single pool. Forty microliters from each of the 10 plate pools and 40 µL from the master pool underwent library size selection and purification using equal volumes of SPRIselect beads (Beckman Coulter) and ultrapure water (Invitrogen). Sample cleaning with SPRI-beads was performed as described previously [4]. A purified master pool comprising samples from all plates, and purified pools of individual plates to check for plate-specific anomalies, were diluted to 4 nM and fragment size distribution was assessed using the High Sensitivity DNA kit on the Bioanalyzer (Agilent Technologies, USA).

Normalization and sequencing

The master pool was sequenced on an Illumina MiSeq v2 300 cycle nano flow cell (Illumina, USA). Read counts were obtained and used to normalise libraries. The liquid handling robot OT-One (Opentrons) was programmed to re-pool libraries based on read counts obtained from the previous MiSeq run. The code used to achieve the normalization is available through our Github repository.

The read count distribution after normalisation is displayed in **Supplementary Figure 2**. The normalized and purified pooled library was sequenced on an Illumina NovaSeq 6000 S4 flow cell at the Ramaciotti Centre for Genomics (Sydney, NSW, Australia), generating a total of 27 billion read pairs from 911 samples.

Sequence data processing

Adapter trimming (parameters: k=23 hdist=1 tpe tbo mink=11), PhiX DNA removal (parameters: k=31 hdist=1), and quality filtering (parameters: ftm=0 qtrim=r trimq=20), were performed using bbduk.sh (http://jgi.doe.gov/data-and-tools/bbtools; bbmap version 38.22). Piglet samples (*n*=825) had a median count of 32,949,208 clean paired reads (mean=35,557,149) (script: *readcounts.R*). Quality assessment of raw reads was carried out using FASTQC (http://www.bioinformatics.babraham.ac.uk/projects/fastqc/) and a combined report of all samples was obtained with MULTIQC [5]. The presence of PCR duplicates was assessed by feeding read pairs to dedupe.sh (http://jgi.doe.gov/data-and-tools/bbtools) (parameters: ac=f). Nextflow [6] (version 18.10.1) was used to manage processing of the data on the HPC.

Comparison of the expected and the observed taxonomic profile of the positive controls

All the mock community members, in seven of the eight technical replicates, were detected by MetaPhlAn2 (version 2.7.7) (**Supplementary Figure 3**). One sample failed to sequence, reporting zero counts for any species. The observed mean relative abundances were as follows: *B. subtilis* (mean±SD: 2.92±0.994), *E. cloacae* (mean±SD: 38.0±6.404), *E. faecium* (mean±SD: 0.97±0.081), *E.coli* (mean±SD: 10.12±1.480), *E.coli* unclassified (mean±SD: 7.83± 1.755), *P. aeruginosa* (mean±SD:

26.72±3.026), *S. aureus* (mean±SD: 9.90±3.613), *S. epidermidis* (mean±SD: 3.54±1.435). Isolate *E. cloacae* C15117, used in this study for the make-up of the mock community, was recently found to be most closely related to the *Enterobacter hormaechei* phylogenomic group C type strain DSM 16687 and therefore re-identified as *Enterobacter hormaechei* subsp. *oharae*[7] . For this reason, taxonomic assignment by MetaPhlAn2 attributed the reads to *E. cloacae* instead. The expected proportions of the mock community members were derived from the CFU by the genome size. Based on the expected and the observed relative abundance, we found, with the exception of *S. aureus* (exp: 8.7% obs: 9.9%), three Gram positive members to be under-represented (*B. subtilis*: exp: 13.0% obs: 2.9%; *E. faecium:* exp: 7.7% obs: 1.0%; *S. epidermidis*: exp: 16.7% obs: 3.5%) and, with the exception of *P. aeruginosa* (exp: 38.9% obs: 26.8%), two Gram negative members to be over-represented (*E. cloacae:* exp: 14.5% obs: 38.0%; *E.coli*: exp: 0.4% obs: 7.8-10.1%) (**Supplementary Figure 4**). Taxonomic assignment of the mock community samples reported one contaminating species in one of the eight replicates: *Lactobacillus salivarius* (mean: 0.008) (**Supplementary Figure 5**).

The probiotic D-Scour™ is expected to contain, per gram, a total of 180 million CFU of *Lactobacillus acidophilus*, *Lactobacillus delbrueckii* subspecies *bulgaricus*, *Lactobacillus plantarum*, *Lactobacillus rhamnosus*, *Bifidobacterium bifidum*, *Enterococcus faecium*, *Streptococcus salivarius* subspecies *thermophilus* in unknown proportions. From taxonomic analysis with MetaPhlAn2, we can conclude that 6 of the 7 expected species were determined to be present in the replicates in the following mean relative abundances: *Bifidobacterium bifidum*: mean±SD: 40.01±12.558; *Enterococcus faecium*: mean±SD: 30.98±13.472; *Lactobacillus delbrueckii*: mean±SD: 11.56±7.148; *Lactobacillus plantarum*: mean±SD: 6.23±7.863; *Lactobacillus rhamnosus*: mean±SD: 2.08±1.226; *Streptococcus thermophilus*: mean±SD: 4.28±1.523. *Lactobacillus acidophilus* was not detected and *Lactobacillus helveticus* was detected instead (*Lactobacillus helveticus*: mean±SD: 4.75±2.431) (**Supplementary Figure 3**). An additional 25 taxa were detected, of which 18 and 7 were identified at the species and at the genus level, respectively. Contaminants were present at a higher concentration in three technical replicates (R3, R7, R8) with the most frequent contaminant (*Methanobrevibacter* spp.) being present in 5 of the 8 replicates (**Supplementary Figure 5**).

Taxonomic analysis of the technical replicates of the probiotic ColiGuard® also showed a species profile consistent with the expected profile, with *Lactobacillus salivarius* and *Lactobacillus plantarum* in a 9:1 ratio (*Lactobacillus salivarius*: mean±SD: 93.52±1.617; *Lactobacillus plantarum*: mean±SD: 6.10±1.134) across the replicates (**Supplementary Figure 3**). ColiGuard® contained a total of 20 contaminants, of which 16 and 4 were identified at the species and the genus level, respectively. Contaminants were present at a higher level in two technical replicates (R5, R7), with R7 displaying the most diverse and highest contamination rate (R7: 14 taxa; total contaminating reads: 2.67%; R5: 9 taxa; total contaminating reads: 0.30%). (**Supplementary Figure 5**).

Technical controls in metagenomic studies and methodological limitations

Taxonomic assignment of the raw reads from the positive controls was performed with MetaPhlAn2 [8] which relies on a ca. 1M unique clade-specific markers derived from 17,000 reference genomes. Such a database to map against the positive controls suffices as these organisms are cultivable, and for this reason they are widely studied hence the sequences are known. This is not the case for real-world samples where mapping against a database (the completeness of which relies on studied and often cultivable organisms) would narrow the view on the true diversity within the sample.

Positive controls with well-studied members and known ratios within the samples have proven to be a valuable approach to assess consistency among technical replicates across batches and to detect possible biases derived from the DNA extraction method. Systematic taxonomic bias in microbiome studies, resulting from differences in cell wall structures between Gram positive and Gram negative bacteria, have previously been reported; bead beating and sample treatment with enzymatic cocktails can modestly reduce this bias [9–12]. Although we implemented such steps in our workflow, it seems that, from the read abundance of our mock community, which contained three Gram negative and four Gram positive strains, a bias towards Gram negative taxa may still be present. However, this cannot be conclusively determined by our study as the expected amount of cells was derived from the colony forming units (CFUs) method, which does not account for dead cells. Those cells would nonetheless contribute to gDNA to the sample [13–16]. Knudsen *et al* (2016) compared various DNA isolation

methods with distinct sample types and reported a reduced bias when using an adapted version of the QIAamp Fast DNA Stool Mini Kit (Qiagen) [12].

In terms of contamination we concluded that: a) contamination in our study was not batch specific; b) a problem of sample cross-contamination may have occurred at the DNA extraction step between neighbouring wells. During the bead-beating step of DNA extraction, the deep-well plate is sealed with a rubber sealing mat, rotated and placed in a plate shaker for the bead beating to take place. As leakage was observed around the wells despite the presence of the sealing mat, we consider that sample cross-contamination is most likely to occur during this step.

Taxonomic profiling of samples

All raw reads were analysed with SortMeRNA [1] (version 4.0.0) to extract reads containing 16S rRNA genes. Extraction was performed by mapping reads against the silva-bac-16s-id90.fasta database with –fastx --blast 1 –num_alignments 1 parameters settings (script: *sortmerna.sh*). Over 60 million reads (*n*=60,584,650) contained 16S rRNA genes passing the E-value threshold for filtering (E-value $\leq$ 0.0001). These reads occupy between 36.4% and 37.1% of each sample (script: *sortmerna_counts.R*). Reads were further filtered based on E-value cutoff (E-value $\leq 1x10^{-30}$), sequence identity (identity $\geq$ 80%), and alignment length (length $\geq$ 100 bp). Over half of the reads (*n*=32,419,310) passed the filtering (script: *sortmerna_filter.sh*) and were classified using the RDP classifier [2] (version 2.13), a naïve Bayesian classifier, that classifies 16S rRNA sequences into the new higher-order taxonomy proposed in Bergey's Taxonomic Outline of the Prokaryotes (2nd ed., release 5.0, Springer-Verlag, New York, NY, 2004) (script: *RDP_Krona.sh*). Most abundant Phyla in the piglet population (*n*=126) were: *Firmicutes* (75.14%), *Bacteroidetes* (13.70%), *Actinobacteria* (5.31%), *Proteobacteria* (3.17%), *Spirochaetes* (0.72%), *Synergistetes* (0.40%). Most abundant Phyla in the mothers (*n*=42) were: *Firmicutes* (84.75%), *Proteobacteria* (6.30%), *Bacteroidetes* (5.44%), *Actinobacteria* (1.85%), *Verrucomicrobia* (0.36%), *Synergistetes* (0.23%). The RDP classifier estimates the confidence of an assignment using the number of times a genus is selected out of 100 bootstrap trials. Assignments at the phylum level had a mean confidence of 0.93 (scale

0-1; median=1.00) (script: *RDP_analyze.R*). Classifications were displayed using Krona [3] (version 2.7.1).

Alpha and beta diversity

The abundance profile of all samples, based on the 16S rRNA reads that passed filtering (E-value ≤ $1x10^{-30}$; identity ≥ 80%; length ≥ 100 bp; *n*=32,419,310; script: *sortmerna_filter.sh*) was used to estimate alpha and beta diversity with PhyloSeq [17] (version 1.28.0) (script: *sortmerna_diversity_giga.R*). Samples with less than 10,000 read counts were excluded.

For α diversity, library normalisation was obtained by rarefaction and the Chao1, Shannon, and Simpson diversity indices were obtained. Confidence intervals (CI) for differences between time points were obtained using the R package pairwiseCI (v0.1-27). Sample diversity estimates were compared between time points using the t-test and adjusting significance with the Bonferroni method. Alpha diversity decreased in terms of species richness and evenness between the first week (t0) and the 5th week post weaning (t10) (Shannon diversity estimate=-0.18 se=0.10 *p*=0.0078). The mothers had a higher species richness compared to the piglets in the first week post-weaning (t0; Chao1 estimate=162.28 se=64.20 *p*<0.0001) and in the 5th week post-weaning (t10; Chao1 estimate=146.20 se=59.64 *p*=0.0001).

For β diversity, library normalisation was obtained by rarefaction. A principal coordinate analysis (PCoA) was performed. Multivariate analysis of variance (ANOVA) was used to compare groups, and t-tests were used for pairwise comparisons, adjusting the significance with the Bonferroni method. Time points (piglet age) were found to be predictive of variance in microbial gut communities (F=294.6, *p*<0.001). Samples from the piglets (all time points) and the mothers significantly separated in β diversity (F=7.692; *p*=0.00572)

Potential uses

This dataset can be utilised to assess a broad range of ecological questions pertaining to host-associated microbial communities of the post-weaning piglet. These include the assessment of: 1. the compositional and functional core faecal microbiome of the post-weaning piglet, 2. the microbial changes that piglets undergo between the first and the

5th week after weaning, 3. the degree of strain-host specificity, 4. the variability of microbiomes within or between host species, 5. the variability of microbiomes between different cross-breeds and small age differences of the hosts, 6. the degree of strain transfer from mothers to piglets, 7. the effects of two probiotic treatments and of intramuscular antibiotic treatment on the post-weaning pig faecal microbiome, 8. species co-occurrence and co-exclusion, 9. the repertoire of antimicrobial resistance genes and how it is impacted by antibiotic and probiotic treatment, 10. the extent of within-host and population evolution of microbes over a 5-week period.

Data availability

The sequencing reads from each sequencing library have been deposited at NCBI Short Read Archive under project PRJNA526405. All supplementary figures and tables are provided as additional files. The scripts for the automated robot pooling (*robot_pooling.py*), for the sequence data processing (*initial.nf*), and for the data analysis can be found in our Github repository (https://github.com/GaioTransposon/metapigs_base; tag: GigaScience). For the data analysis scripts, the R language (version 3.6.3) and the following packages were used: readr (v1.4), readxl (v1.3.1), tidyr (v1.1.2), tidyverse (v1.3.0), ggplot2 (v3.3.3), dplyr (v1.0.3), gridExtra (v2.3), pheatmap (v1.0.12), cowplot (v1.1.1), splitstackshape (v1.4.8), pairwiseCI (v0.1-27).

Figures

Figure 1. Microbial composition and diversity of the porcine microbiome.

Taxonomic profiling based on the analysis of reads containing bacterial 16S rRNA genes extracted from shotgun metagenomic data. **A**) The chart was generated using Krona [3], which displays hierarchically organized nodes of the taxonomic tree based on their relative abundance. Distinct colours represent separate domains of life. **B**) Principal coordinate analysis (PCoA) plots of the pig faecal microbiomes explaining 19.3% of variance (Axis.1) and 12.5% of variance (Axis.2). Samples are colored by

day of collection from the first week post-weaning (t0) to the 5th week post-weaning (t10). Panels are split by cohort: Mothers (tM; bright pink; *n*=42); Control (*n*=30); D-Scour (*n*=18); ColiGuard (*n*=18); Neomycin (*n*=24); Neomycin+D-Scour (*n*=18); Neomycin+ColiGuard (*n*=18).

Figure 2. Timeline.

Timeline of the animal trial indicating the start and the length of the treatment for each cohort, the sample collection points, and the piglets' age during the trial. Piglets were allowed 2 days of acclimatisation after the arrival on the site of the trial and before the start of the treatments (pink: placebo paste; yellow: probiotic D-Scour™ formulation; green: probiotic ColiGuard® formulation; blue: antibiotic neomycin intramuscular injection). Large triangles (dark blue) indicate main days of sampling where all piglets were sampled (*n*=126). Small triangles (light blue) indicate sampling points from a subset of the piglets (8 per cohort; *n*=48).

Figure 3. Workflow.

A schematic representation of the experimental workflow from sample collection (yellow), through sample processing and sequencing (orange), to the preliminary data analysis (blue).

Additional files

Supplementary Figure 1: Piglets placements across rooms and pens.

Supplementary Figure 2: Read count distribution.

Supplementary Figure 3: Taxonomic assignment of reads from positive control samples.

Supplementary Figure 4: Expected and observed relative abundance of mock community members.

Supplementary Figure 5: Heatmap reporting the contaminating species found within the technical replicates of the positive controls.

Supplementary Table 1: Animal details**.**

Supplementary Table 2: Metadata.

Supplementary Table 3: Barcodes.

Funding information

This work was supported by the Australian Research Council, linkage grant LP150100912. This project was funded by the Australian Centre for Genomic Epidemiological Microbiology (Ausgem), a collaborative partnership between the NSW Department of Primary Industries and the University of Technology Sydney. TZ and DG are recipients of UTS International Research and UTS President's Scholarships. NSW DPI approved the paper before submission for publication.

Competing interests

D-Scour™ was sourced from International Animal Health Products (IAHP). ColiGuard® was developed in a research project with NSW DPI, IAHP and AusIndustry Commonwealth government funding.

Author contributions

Pig Trial: TC, LF, DG, TZ, GJE, AED, SPD

DNA extraction: DG, ML

Library prep, robot pooling: DG, ML, KA, AED

Sequencing data processing: MZD, DG, AED

Data analysis: DG, AED

Manuscript: DG

Manuscript editing: DG, AED, GJE, John Webster

Acknowledgements

We would like to thank Shayne Fell for the on site help in the pig trial. Thank you Amy Bottomley, Giulia Ballerin, Rosy Cavaliere, for providing the mock community strains. Thank you Akane Tanaka, Leigh Monahan and Joyce To for the technical support. Thank you John Webster for providing DPI internal review of this manuscript

and for the helpful suggestion about its content. Thanks to International Animal Health Products for providing access to the probiotic supplements and to the Australian Centre for Genomic Epidemiological Microbiology (Ausgem) for financially supporting this study. We thank the peer reviewers whose comments improved the quality of this manuscript.

REFERENCES

1. Kopylova, E., Noé, L. & Touzet, H. SortMeRNA: fast and accurate filtering of ribosomal RNAs in metatranscriptomic data. *Bioinformatics* **28**, 3211–3217 (2012).
2. Wang, Q., Garrity, G. M., Tiedje, J. M. & Cole, J. R. Naive Bayesian classifier for rapid assignment of rRNA sequences into the new bacterial taxonomy. *Appl. Environ. Microbiol.* **73**, 5261–5267 (2007).
3. Ondov, B. D., Bergman, N. H. & Phillippy, A. M. Interactive metagenomic visualization in a Web browser. *BMC Bioinformatics* **12**, 1–10 (2011).
4. Gaio, D. *et al.* Hackflex: low cost Illumina sequencing library construction for high sample counts. *bioRxiv* 779215 (2019).
5. Ewels, P., Magnusson, M., Lundin, S. & Käller, M. MultiQC: summarize analysis results for multiple tools and samples in a single report. *Bioinformatics* **32**, 3047–3048 (2016).
6. Ewels, P. A. *et al.* nf-core *: Community curated bioinformatics pipelines*. (2019).
7. Monahan, L. G. *et al.* High contiguity genome sequence of a multidrug-resistant hospital isolate of Enterobacter hormaechei. *Gut Pathog.* **11**, 3 (2019).

8. Truong, D. T. *et al.* MetaPhlAn2 for enhanced metagenomic taxonomic profiling. *Nat. Methods* **12**, 902–903 (2015).
9. von Wintzingerode, F., Göbel, U. B. & Stackebrandt, E. Determination of microbial diversity in environmental samples: pitfalls of PCR-based rRNA analysis. *FEMS Microbiol. Rev.* **21**, 213–229 (1997).
10. Han, Z., Sun, J., Lv, A. & Wang, A. Biases from different DNA extraction methods in intestine microbiome research based on 16S rDNA sequencing: a case in the koi carp, Cyprinus carpio var. Koi. *MicrobiologyOpen* **8**, e00626 (2019).
11. Moss, E. L., Maghini, D. G. & Bhatt, A. S. Complete, closed bacterial genomes from microbiomes using nanopore sequencing. *Nat. Biotechnol.* (2020) doi:10.1038/s41587-020-0422-6.
12. Knudsen, B. E. *et al.* Impact of sample type and DNA isolation procedure on genomic inference of microbiome composition. *MSystems* **1**, (2016).
13. McLaren, M. R., Willis, A. D. & Callahan, B. J. Consistent and correctable bias in metagenomic sequencing experiments. *Elife* **8**, e46923 (2019).
14. Cruz, G. N. F., Christoff, A. P. & de Oliveira, L. F. V. Equivolumetric Protocol Generates Library Sizes Proportional to Total Microbial Load in 16S Amplicon Sequencing. *Front. Microbiol.* **12**, 425 (2021).
15. Ou, F., McGoverin, C., Swift, S. & Vanholsbeeck, F. Absolute bacterial cell enumeration using flow cytometry. *J. Appl. Microbiol.* **123**, 464–477 (2017).
16. Bensch, G. *et al.* Flow cytometric viability assessment of lactic acid bacteria starter cultures produced by fluidized bed drying. *Appl. Microbiol. Biotechnol.* **98**, 4897–4909 (2014).

17. McMurdie, P. J. & Holmes, S. phyloseq: an R package for reproducible interactive analysis and graphics of microbiome census data. *PloS One* **8**, (2013).

Figure

Click here to access/download;Figure;metapigs_base_DataNote_Figu

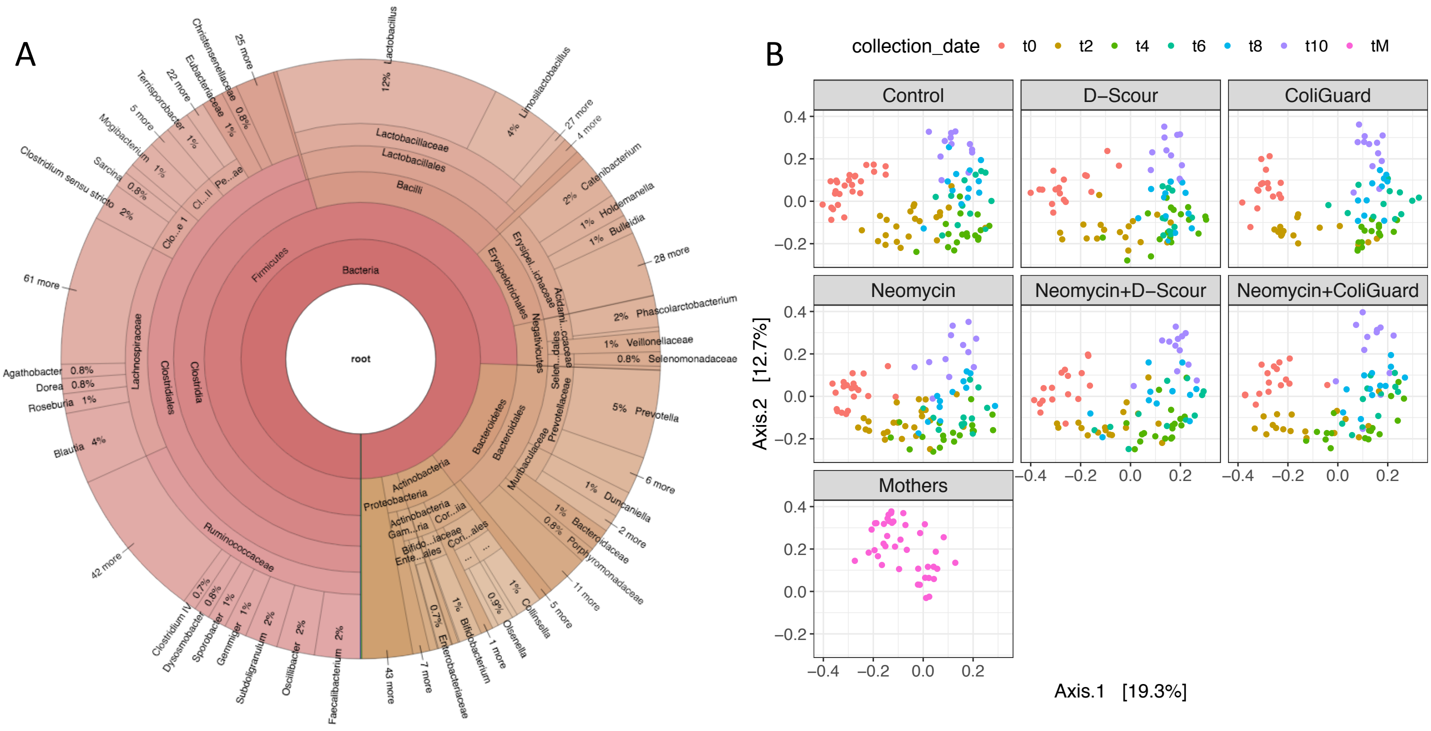

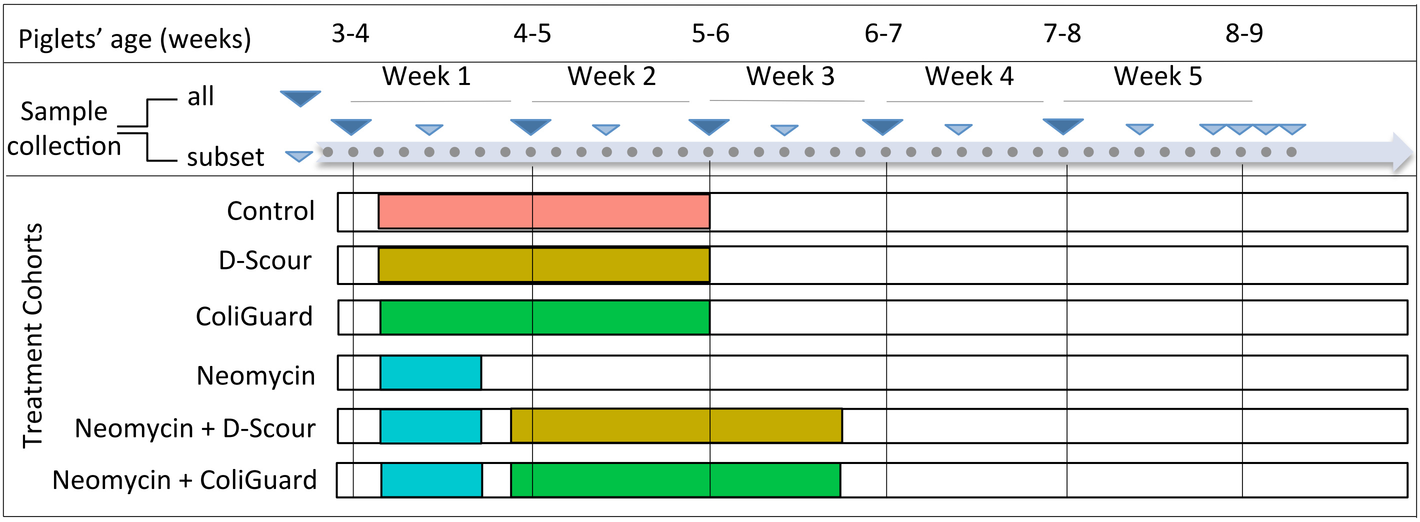
Piglets' age (weeks)
3-4
4-5
5-6
6-7
7-8
8-9
Week 1
Week 2
Week 3
Week 4
Week 5
Sample collection
all
subset
Treatment Cohorts
Control
D-Scour
ColiGuard
Neomycin
Neomycin + D-Scour
Neomycin + ColiGuard

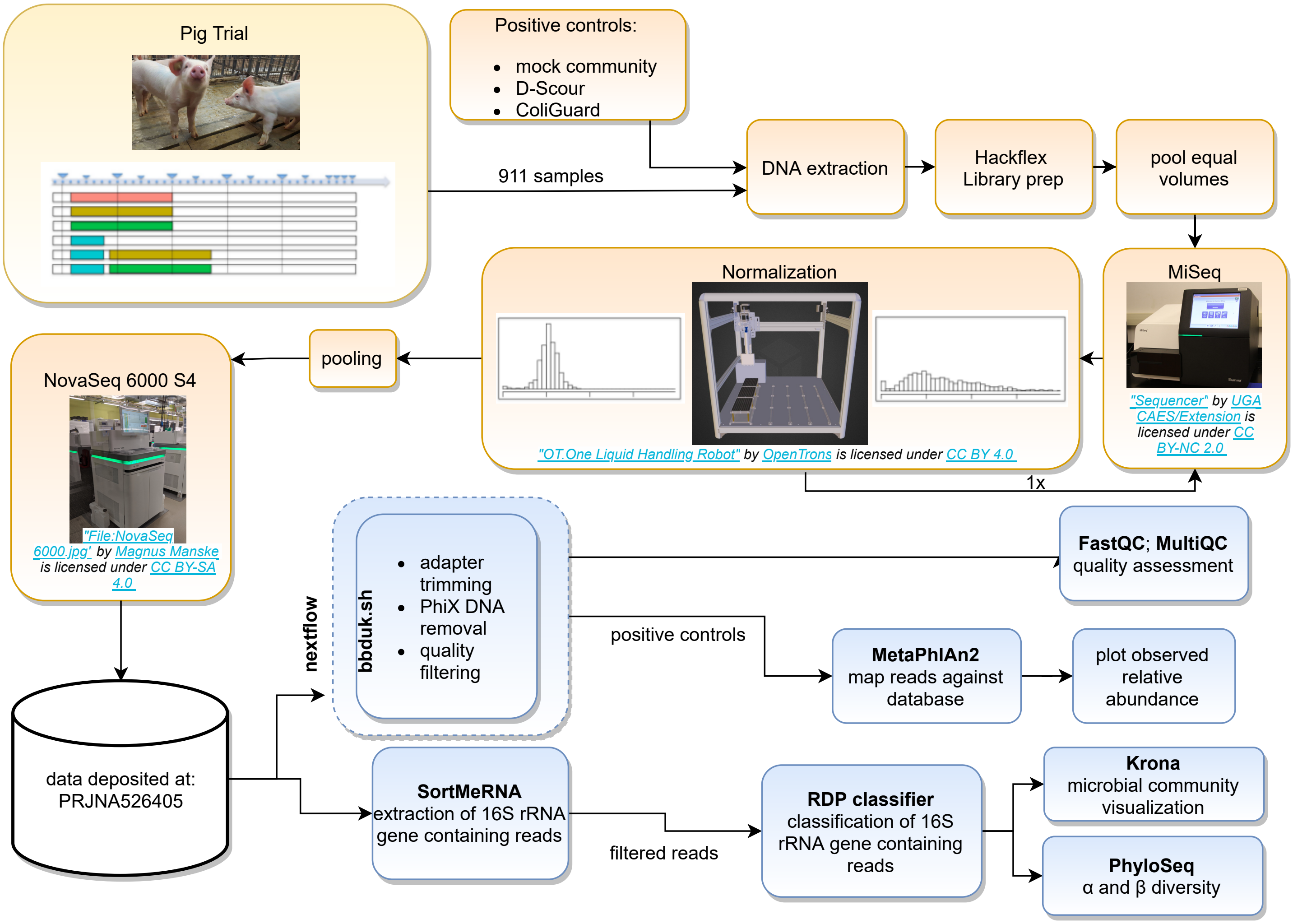

Pig Trial
Positive controls:
mock community
D-Scour
ColiGuard
911 samples
DNA extraction
Hackflex Library prep
pool equal volumes
MiSeq
"Sequencer" by UGA CAES/Extension is licensed under CC BY-NC 2.0
Normalization
"OT.One Liquid Handling Robot" by OpenTrons is licensed under CC BY 4.0
1x
pooling
NovaSeq 6000 S4
"File:NovaSeq 6000.jpg" by Magnus Manske is licensed under CC BY-SA 4.0
data deposited at: PRJNA526405
nextflow
bbduk.sh
adapter trimming
PhiX DNA removal
quality filtering
FastQC; MultiQC quality assessment
positive controls
MetaPhlAn2 map reads against database
plot observed relative abundance
SortMeRNA extraction of 16S rRNA gene containing reads
filtered reads
RDP classifier classification of 16S rRNA gene containing reads
Krona microbial community visualization
PhyloSeq α and β diversity

Supplementary Table 1

Click here to access/download

Supplementary Material

metapigs_base_DataNote_SupplTable1.xlsx

Supplementary Table 2

Click here to access/download
Supplementary Material
metapigs_base_DataNote_SupplTable2.csv

Supplementary Figures

Click here to access/download
Supplementary Material
metapigs_base_DataNote_SupplFigures.pdf
