# Supplementary material for: A large-scale metagenomic survey dataset of the post-weaning piglet gut lumen
Source: Gigascience. 2021 Jun 3;10(6):giab039. doi: 10.1093/gigascience/giab039 (PMC8173662; doi:10.1093/gigascience/giab039)
Supplement: giab039_Supplemental_Files [file giab039_supplemental_files.zip › metapigs_base_DataNote_SupplFigures.pdf]

A. Allocation of piglets in rooms and pens on the day of arrival

B. Re-distribution of piglets on February 6th

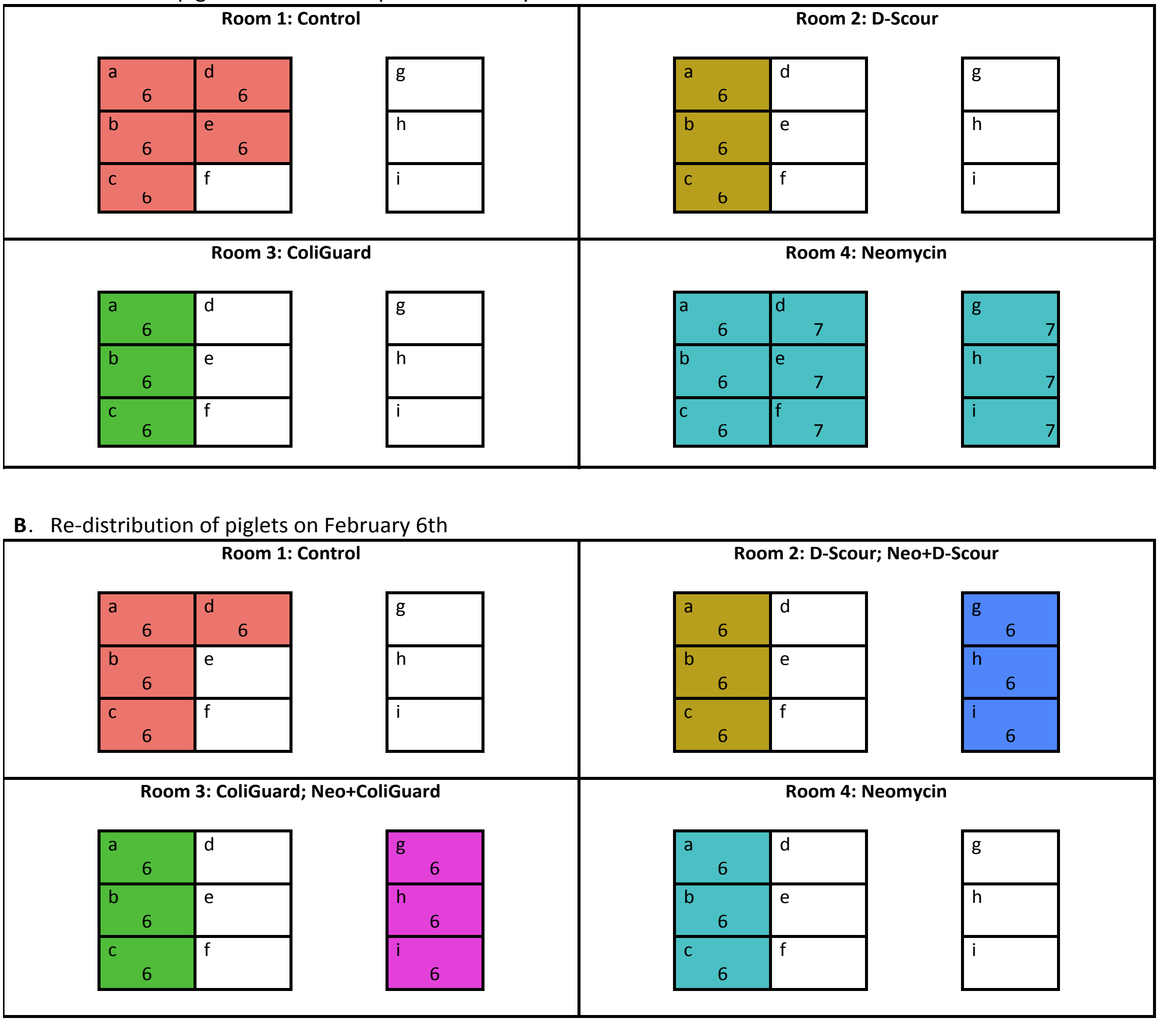

Supplementary Figure 1. Piglet placements across rooms and pens.

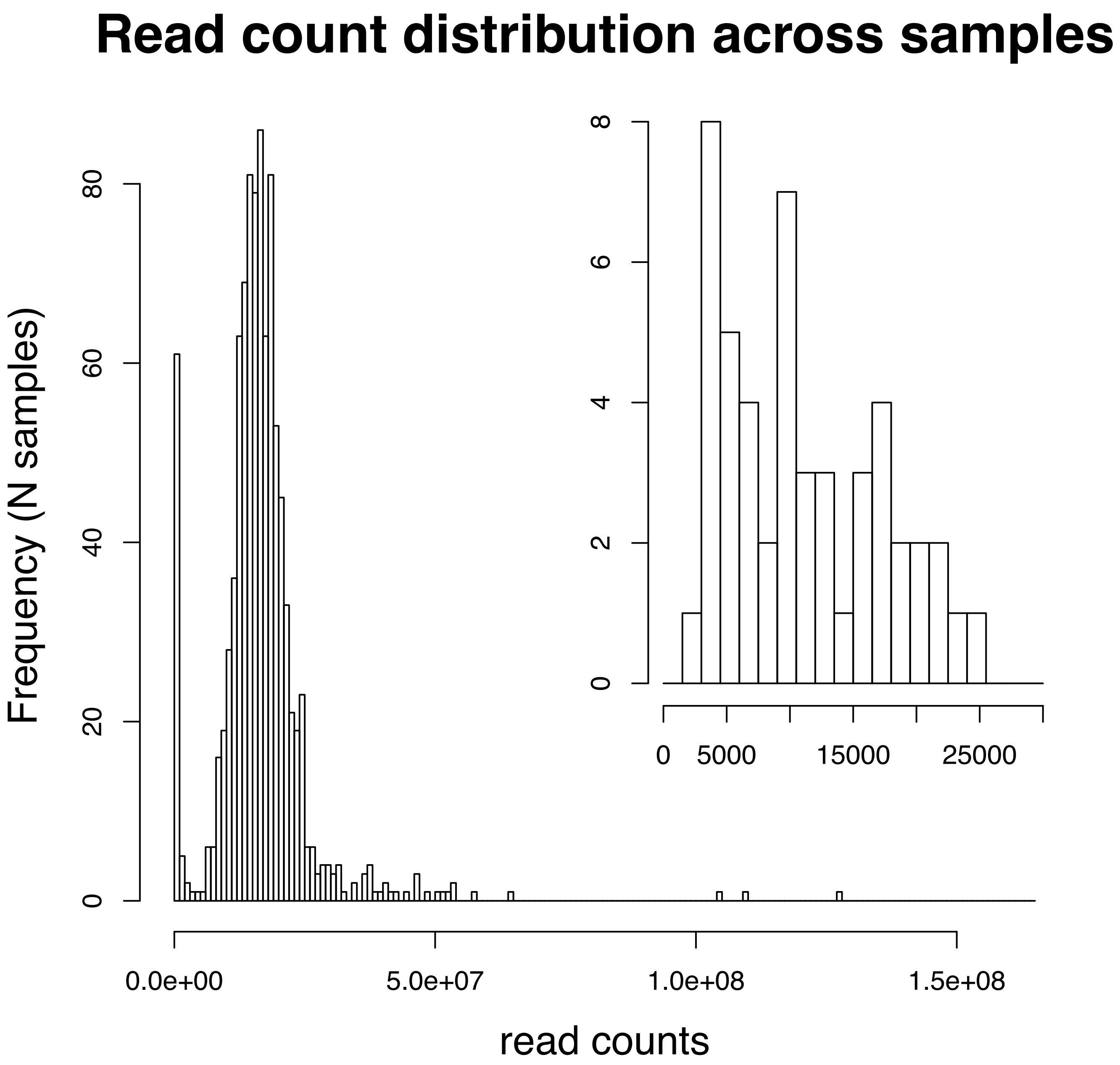

Supplementary Figure 2 Read count distribution.

Read count distribution of all samples (main histogram; bin size = 1,000,000) and low read count samples (top right histogram; bin size = 1,500). A small number of samples were pooled at higher molarity to generate higher coverage for *de novo* assembly, reflected here in the high read count – low frequency bins.

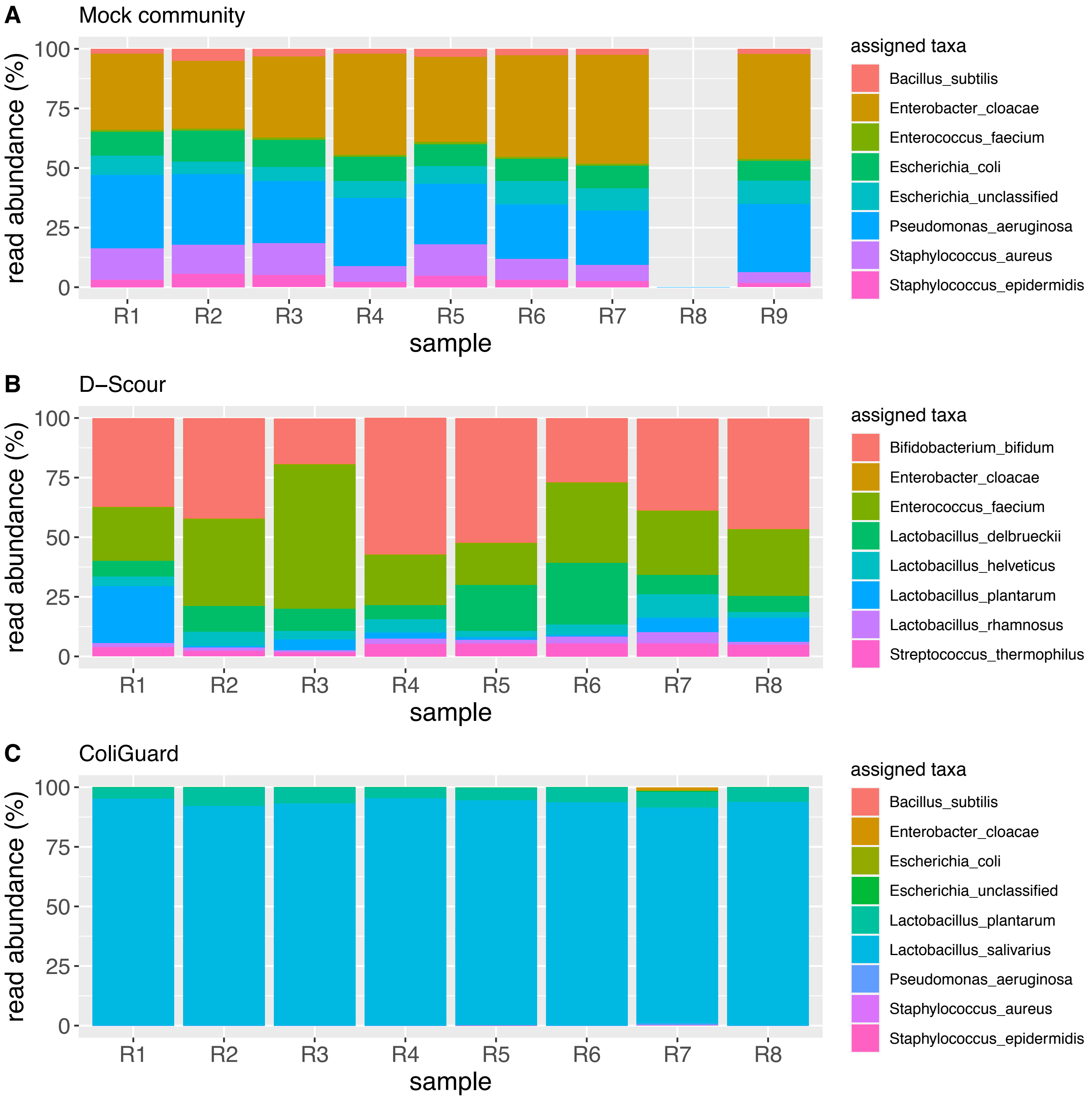

Supplementary Figure 3 Taxonomic assignment of reads from positive control samples.

Taxonomic profile of the positive controls used in this study obtained by analysing the reads with a database of ~1M bacterial genomes with MetaPhlAn2. Each stacked barplot represents a technical replicate. Taxonomic groups appearing in >0.1% of reads are displayed. **A**. In-house made mock community; **B**. Commercially available livestock probiotic D-Scour™ ; **C**. ColiGuard®.

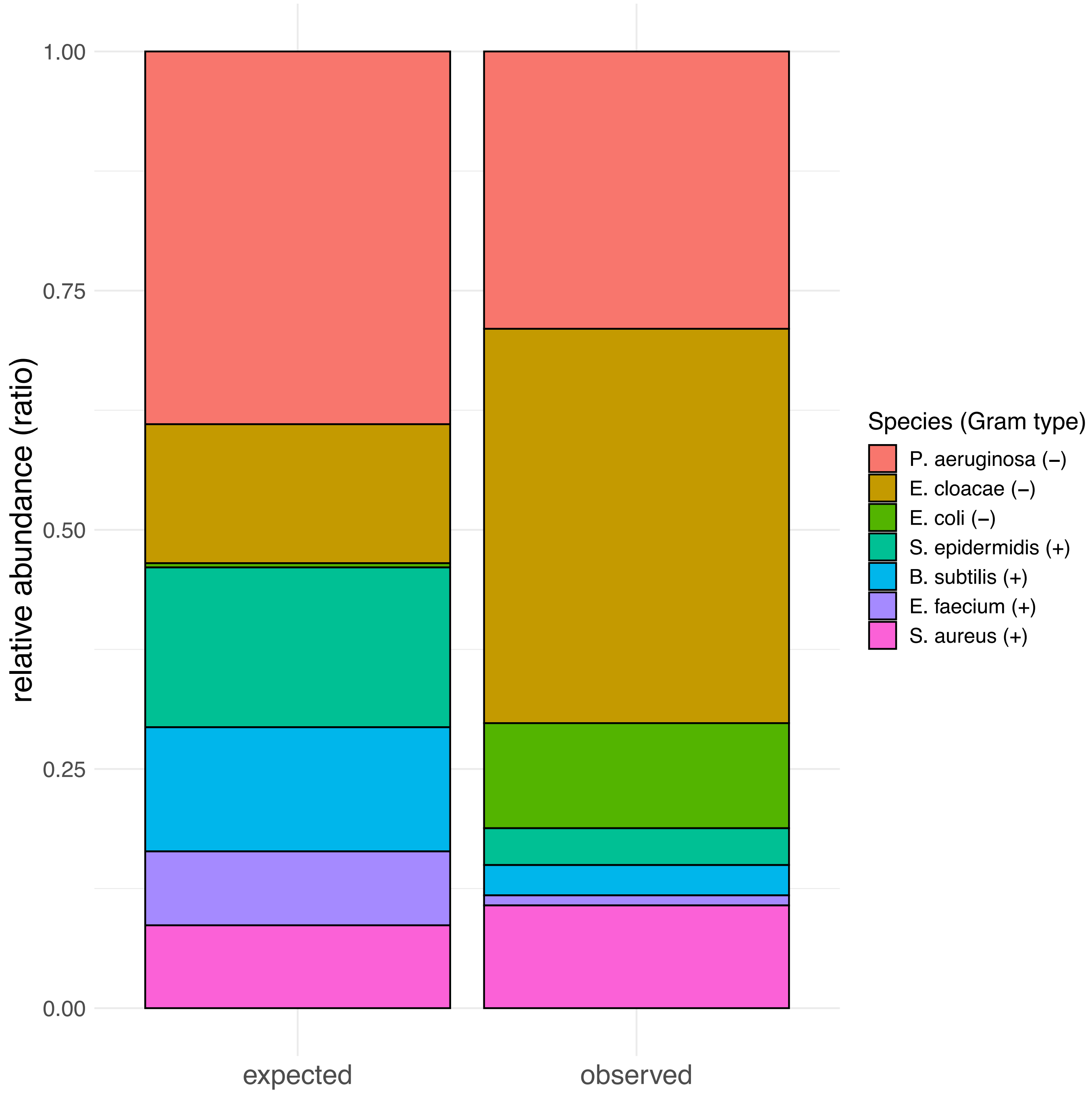

Supplementary Figure 4 Expected and observed relative abundance of mock community members.

Expected relative abundance is derived by multiplying CFU count by genome size. Observed relative abundance is derived by taxonomic assignment of reads with MetaPhlAn2. A higher observed/expected ratio is seen in two of the three Gram negative species and a lower observed/expected ratio is seen in three of the four Gram negative species.

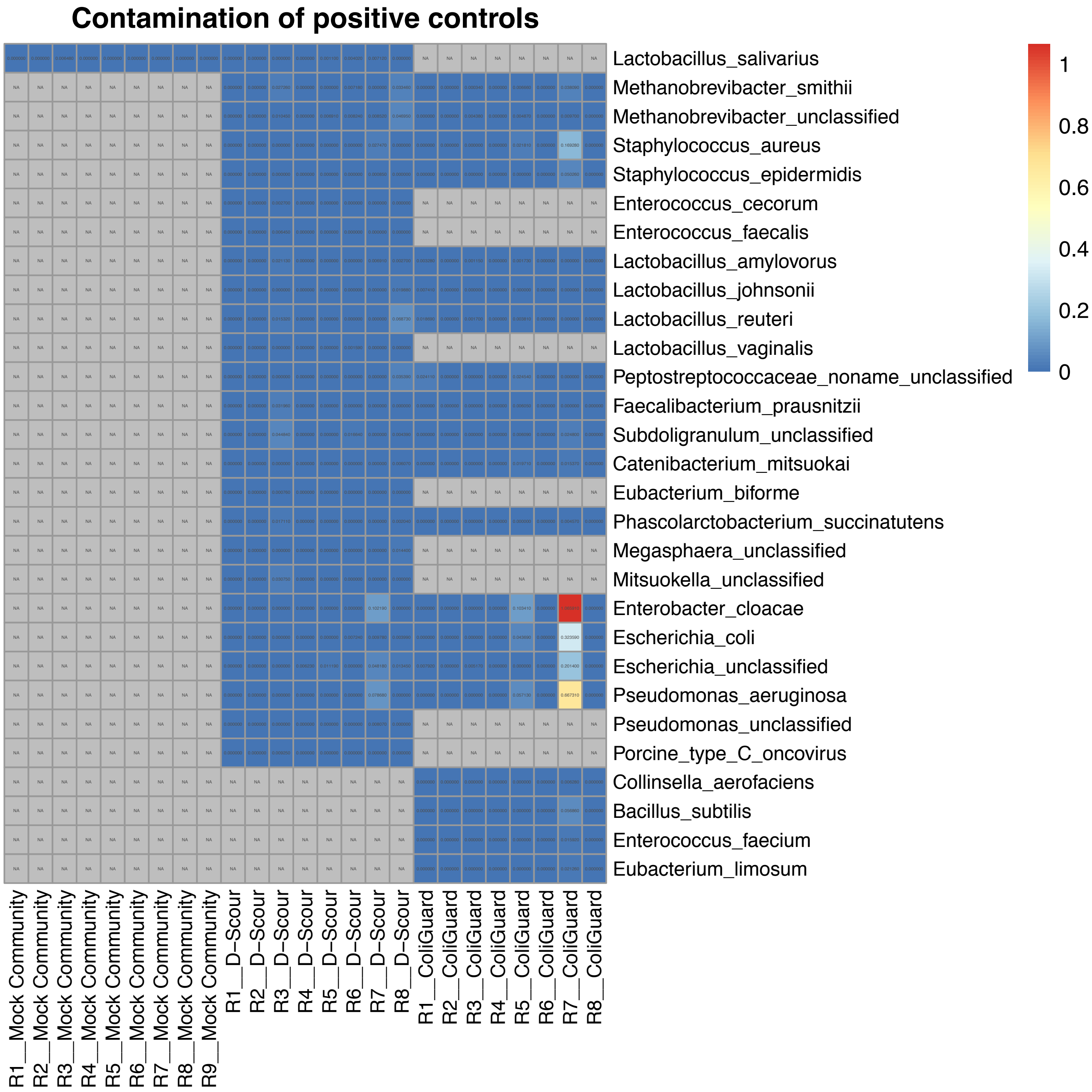

Supplementary Figure 5. Heatmap reporting the contaminating species found within the technical replicates of the positive controls.

The mock community technical replicates contained one apparent contaminant, *Lactobacillus salivarius,* in one replicate at 0.01% of the total reads; The D-Scour™ technical replicates contained 25 apparent contaminants, of which 18 and 7 were identified at the species and at the genus level, respectively. Contaminants of the positive control D-Scour™ are most abundant in three technical replicates (R3, R7, R8) and the most prevalent contaminant (*Methanobrevibacter*) was present in 5 of the 8 replicates; ColiGuard® contained 20 contaminants, of which 16 and 4 were identified at the species and at genus level, respectively. Contaminants of the positive control ColiGuard® were present primarily in two technical replicates (R5, R7). None of the contaminants were present in all the technical replicates of a positive control, suggesting that these contaminants were not present in the positive controls prior to DNA extraction and library preparation.
