# Supplementary material for: A large-scale metagenomic survey dataset of the post-weaning piglet gut lumen
Source: Gigascience. 2021 Jun 3;10(6):giab039. doi: 10.1093/gigascience/giab039 (PMC8173662; doi:10.1093/gigascience/giab039)
Supplement: giab039_Response_to_Reviewer_Comments_Original_Submission [file giab039_response_to_reviewer_comments_original_submission.pdf]

Author's Response To Reviewer Comments

Close

Reviewer #1: This study reported 8Tbp of shotgun metagenomic sequence data for 800 fecal microbiota collected
from 126 piglets and 42 sows. The samples were collected in a longitudinal manner and the treatments were well
controlled. However, your data analysis, especially shotgun sequence data, needs to be improved.
Here are my questions:

1. Why you did shotgun metagenomic sequencing rather than 16S rRNA gene sequencing as you only did
compositional analysis with this 8Tbp of sequence data. Analysis of 16S rRNA gene sequence could definitely do the
same thing and it's much easier and cheaper. It there is any advantages of your analysis over the common used
workflow with 16s rRNA gene sequence? The main advantage of shotgun sequence is the possibility to get more
functional information other than bacterial composition of our metagenomic samples. Why you didn't do any functional
analysis?

We generated the data with a view towards functional analysis, effects of antibiotic and probiotic treatment on
function, and particularly towards investigation of the resistome. Many analyses have been performed on the data,
including functional analysis, and it is too much material to describe in a single manuscript. Now that the Data Note
format has been adopted at the Editor's suggestion, this manuscript contains only a summary-level analysis of the
data that will be referenced by the other manuscripts which describe detailed analyses.

2. What is the significance of this study? The strong effect of time /age on gut microbiota of post-weanling pig have
been reported in many publications.

The main purpose of the study was to evaluate the effects of probiotic and intramuscular antibiotic interventions on
the gut microbiome. Secondly, to describe the antimicrobial resistance gene repertoire in an Australian setting and its
change throughout the post-weaning period, and in relation to antibiotic treatment. Now that the Data Note format has
been adopted, the potential uses are listed in the "Potential uses" section.

Detailed comments:
Line 160 Taxonomy of the genus Lactobacillus has been reclassified. Please see more details in this reference : "A
taxonomic note on the genus Lactobacillus: Description of 23 novel genera, emended description of the genus
Lactobacillus Beijerinck 1901, and union of Lactobacillaceae and Leuconostocaceae"

In the interest of reproducibility of analysis we used a public database that was integrated to an automated analysis
software package at the time the analysis was carried out.

Line 229 How is the quality of the extracted 16S gene reads? How many reads were extracted for each

sample?
Further details on the 16S reads have been added to the “Taxonomic profiling of samples” section.

Line 230 How did you assign taxonomy to the extracted 16S gene reads? Based on sequencing identity? What are
your parameters for difference taxonomic levels (e.g genus/ family levels)???? Did you cluster the identical reads into
OTUs before assigning taxonomy?

Taxonomic profiling now, in the revised manuscript has been performed in the following manner:
1. rRNA gene containing reads were extracted with SortMeRNA (quality stats are obtained)
2. Reads extracted with SortMeRNA (fastq format) were filtered based on evalue, seq identity, and alignment
length, then fed to the RDP classifier, which assigns taxonomy via a Naive Bayes classifier algorithm (Wang, Q., Garrity, G. M., Tiedje, J. M., & Cole, J. R. (2007). Naive Bayesian classifier for rapid assignment of rRNA sequences into the new bacterial taxonomy. Applied and environmental microbiology , 73 (16), 5261-5267)
This methodology has been reported in the revised Data Note format manuscript.

Line 231 Why yo only use e-value for hits filter? How about the query coverage and identity?

We have rerun the analysis and updated the results in the revised manuscript. We have used SortMeRNA to extract
16S rRNA reads, we have filtered those reads based on an e-value cutoff (1x10^-30), sequence identity of 80% and
alignment length of 100 bp. We fed the filtered reads to RDP classifier, then plotted with Krona.

Line 253 There are too many results and some of them are redundant and less informative. Some of the figures could
be integrated into one and the less relevant results should be deleted.

The Data Note format manuscript is now very short, it has a few figures, and only a few supplementary figures.

Reviewer #2: Gaio et al have analysed the phylogenetic diversity of the fecal pig bacterial communities obtained from
a large temporal piglet study in the period just after weaning. An interesting period due to all sorts of practical
problems that may arise during and after the weaning period. They used shotgun-metagenomics to determine that
phylogenetic diversities are influenced by litter and mainly age. Changes induced by intra muscular injection of an
aminoglycoside only had subtle effect on the community compositions. The metagenomic data described in this study
and shared to the public by NCBI SRA provide a great resource to standardized microbiota data in pigs in the early
age just after weaning. I thank the authors for learning other ways to approach metagenomic community structures.
We thank the reviewer for the extensive and detailed comments. We believe this manuscript (and its successors)
have significantly improved as a result of the reviewer’s efforts.

Generic;
Even though the conclusions, mostly based on weighted phylogenetic diversity, seem supported by the data, overall
the paper confuses me and seems rather lengthy for the actual analysis performed. The same accounts for the too
many supplemental figures to this paper. Furthermore, I do have questions on why this approach was chosen in the
first place, since I cannot derive that directly from the paper in its current form and more simple approaches would
allow the same questions to be answered at the same or even better resolution.

The used shotgun-metagenomics seems rather overdone to the actual questions answered. I assume this paper will be a predecessor of another follow-up paper describing changes in the resistome as well as functional turn-over in time linked to the treatments. But I wonder whether salami-slicing this study would be the way to go. Furthermore, a lot of the data and outcomes are presented as-is while interpretation of these outcomes in context is rather thin and confusing using for instance both phylogenetic distance (PD) and the weighted forms.

We hope that the adoption of the Data Note format addressed most of these concerns.

More specific thoughts.
Even though they seem many, I hope they will help to improve the next version of the manuscript as constructive as possible;

1) A good balanced depth of sequencing per sample was chosen (around 30M PE150 clusters per sample) which seems sufficient as seen in other studies that used SGS in a metagenomics approach in pigs. But why on earth were only the first 100k reads for analysis?? The rationale for this as well as for instance rarefaction-like curves to demonstrate 100k is enough, are missing.

This was initially done as a means to reduce the compute requirements for a preliminary analysis. However, that part of the manuscript has been removed as a consequence of adopting the Data Note format.

2) Why was SG-metagenomics used in the first place? All questions could have been answered by a far cheaper meta-taxonomic approach? SG-metagenomics studies are usually warranted if additional questions to bacterial composition are answered like:
a. Non-bacterial microorganisms like simple eukaryotes, archaea and phages.
b. Other gene compositions like the (acquired) resistome.
c. Functional turn-over.
The analyses could probably suffice from fairly simple permanova community composition comparisons, weighted unifrac beta-diversities and negative binomial modelled differential taxa analysis?

Yes, the purpose of the study is in fact much broader than presented in this particular manuscript, and we used, and will use the data in future analyses, to answer additional questions. The revised manuscript lists several of these downstream analyses.

3) I miss from the paper the rationale for using unweighted and weighted PD's instead of for instance a straightforward weighted unifrac in beta-diversity analysis. It seems overcomplicated where the results describe every time both weighted and unweighted PD's. Is this necessary? And if so why? If weighted PD would be to go, leave out the rest. Would simplify and shorten considerably improving readability to a larger (metagenomic oriented) audience.

As weighted and unweighted PDs reveal different aspects of the microbial composition, the richness and the evenness, respectively, we used both measures. For instance, a probiotic treatment could affect the evenness but not the richness of a community, or vice versa. Therefore interrogating the data regarding the weighted, as well as the unweighted PD, could deliver insights that would otherwise go unnoticed. However, this part of the

analysis is now
excluded in the Data Note format.

4) The title says "…gut microbiome.". Actually the gut was only sampled using a proxy of fecal sampling. Fecal
composition is know not to be the same as in other regions of the gut. Revise throughout.
We agree, this is an important distinction and one that is often not made very clearly by the scientific community,
where "gut microbiome" is commonly used interchangeably with "faecal microbiome".

We revised throughout.

5) The piglet/litter origin linked to the sows were found to be of influence on the piglet fecal community. It would be
interesting to include analysis to demonstrate whether the sow is the actual driver for the piglet community
composition early and later in the study.

Yes it is an interesting analysis, but all specific analyses are now excluded in the Data Note format.

6) The interactions/influence of piglets "at random" in one pen may be interesting to investigate since it may influence
the cross-sectional outcomes instead of following the same animal over time. It is known that the order in which taxa
arrive disproportionate influence later community assembly (Sprochet et al 2018).

We agree this is an interesting analysis. However, all specific analyses are now excluded in the Data Note format.

7) A Major point in this paper is made about the potential dysbiosis due to (oral) antibiotic treatment. Therefore intra
muscular injection was used to study the effects on community composition.
a. However, since the microbiome and resistome diversity prior to starting antibiotic treatment is unknown and a
control group having administered antibiotics orally, seems a bit odd.
b. Furthermore, it would make the study stronger to show that antibiotic residues are actually measured in feces. This
can be done on fecal samples and would strengthen this part of the study.
c. The shotgun metagenomic data is there, why wasn't the effect of antibiotics on the resistome not included? This
can be fairly easy achieved using mapping strategies.

These are good suggestions that will likely be included in another Research-format manuscript.

8) Normalisation, sequencing and standardisation. The overall method is elegant using MiSeq to equilibrate libraries
before sequencing. Some remarks;
a. The authors find that DNA isolation efficiency is biased for groups of species using their community controls. Was
this a surprise? There is an elegant paper describing DNA isolation kit issues in metagenomic shotgun applications of
for instance pig feces (Knudsen et al 2016 mSystems 1(5):e00095-16). Why not adhere to described methods?

No, the extraction bias was not a surprise. The gram stain associated bias has been reported extensively before, and
we were aware of this. As it has been suggested by Knudsen et al (2016), methods without bead beating or
enzymatic treatment have extracted less DNA from Gram-positive bacteria. Based on the literature available, we
chose to perform bead beating as well as an enzymatic treatment during extraction. However, the

MagAttract
PowerMicrobiome kit (Qiagen) had a 96-well option and its performance was well characterised in the literature at the
time. The QIAamp fast stool kit, suggested to have a better performance by Knudsen et al (2016), was a new product
and requires a QIAcube machine for high throughput processing, which we did not have access to. We edited the
new manuscript to report on this study and suggestion of better performance with this kit.

b. Input is "standardized" on total from fecal samples isolated DNA measurements. It is known that especially due to
dysbiosis (i.e. diarrhea) that the ratio of microbial DNA to host or feed-derived DNA may completely shift. The amount
of reads per sample mapped to host or non-microbial origin is helpful and the microbial amounts should be used for
normalization unless it is demonstrated that no significant differences are found between samples for this.

Yes, this is part of the data analysis process. Given that no extensive analyses are described in the Data Note, this is
now not included.

c. Hackflex: Seems an elegant method to try. However, I am not sure on editorial policy referencing a pre-publication
by the same author in BioRxive of over a year old with apparent no peer-reviewed follow-up? Furthermore,
pre-publications do not free authors from discussing/reflecting on comments by readers (if they make sense) to their
pre-pub like the one over a year old without a response.

That manuscript is currently under peer review, however we have been advancing it very slowly due to timing
constraints of the Australian PhD system, which demands that attention be focused first on thesis submission and
peer review, with journal publications taking 2nd priority.

d. Mock communities / control libraries; elegantly made using a cfu approach.
i. However, also dead-bacteria will contribute DNA to the library. Since no strong cfu correlations are made this might
be only of minor effect.

We agree. This is a limitation, as f requency of dead bacterial cells would not be measured by the CFU approach, yet
they could contribute to total DNA and modify relative abundances from read count.

ii. Did the authors check for the presence of plasmids?

Plasmids were not considered in the analysis and a simplifying assumption was made that each bacterial cell
contains 1 copy of the chromosome. That is, the presence of multiple partially replicated copies that are commonly
found in fast growing cells was not considered.

e. Why is a different method used for analyzing the (positive) controls using MethaPhlan2 while the rest of the
samples is analysed by other means like PhyloSift. Positive controls should be used to benchmark the flow and
analysis of the actual samples and therefore I would argue the methods should be the same.

MetaPhlAn2 has high precision in taxonomic assignment and unlike PhyloSift, it is designed to give taxonomic names
so that we could check whether the species expected in the controls were also observed, and to estimate

the
contamination rates. We did not run MetaPhlAn2 on pig samples as were not purely after a taxonomic profiling of the
pig microbiome, but the main purpose of the previously submitted manuscript was to determine whether or not an
effect on composition was detected following probiotic or antibiotic treatment, and this was obtainable using
phylogenetic diversity measures, which was done on all samples, positive controls as well as pig samples. Another
major advantage of the use of phylogenetic diversity measures is that the approach circumvents the library size
normalization, with all the issues that accompany the normalization of samples of compositional nature. However,
now that the extensive data analysis is excluded from the Data Note manuscript, this should no longer be a concern.
We also made a remark in the first paragraph of the section "Technical controls in metagenomic studies and
methodological limitations" on the biased representation of organisms in databases, and the narrowed view that can
be obtained when mapping real samples against such a database.

f. Unfortunate some contaminants were detected in the positive (and probable negative) controls. How did the
authors handle these outcomes? Were corrections applied to the data? Were taxa blacklisted or even removed?

We strongly suspect that the contamination derived from cross-well contamination. The "blacklisting" or removal
belongs to the data analysis step, which is now excluded in the Data Note format.

9) Analysis methods;
a. The KR distance metric was used which seems rather comparable to weighted unifrac. I could not quickly recover
if the KR metric returns Euclidian distances, if not revise the PCA into PCoA.

This part is no longer present in the Data Note format.

b. The weighted and unweighted PD is used. Shouldn't the unweighted PD be performed on rarefied data (at least for
alpha-diversity analysis)? See for instance "McCoy and Matsen (2013) PeerJ 1:e157; DOI 10.7717/peerj.157".

This part is no longer present in the Data Note format.

c. Correcting for bath-effects was done using COMBAT. There is a no-go for me on this. The batch-effect should be
taken as a covariate in the used statistical models and not used to correct the raw-data. This is elegantly
demonstrated by Nygaard et al 2016 Biostatistics 17:29-39.

This part is no longer present in the Data Note format.

10) The section on sortmeRNA method and results are not well integrated in the manuscript and in its current state
only deviate from the main message.

Now that the phylogenetic diversity analysis is removed from the Data Note format manuscript this should no longer
be a concern. The SortMeRNA analysis remains, with the purpose of providing a high level taxonomic overview of the
dataset.

11) Lines 641-646: I found this a very honest remark but a major flaw in processing. If samples get

contaminated
from neighboring wells…. What do the actual results tell us?

Even though there was cross-contamination, the amount of contamination was still relatively low. In fact, it did not prevent us from observing the strong (time trend) as well as the minor trends (treatment effects; small differences in age of the piglets). While the relative abundances in each sample are preserved, analyses of low abundance components are rendered much more challenging, as a result of the cross-well contamination.
Cross-well contamination has also been reported by Minich et al (2019) (see " Quantifying and understanding well-to-well contamination in microbiome research "). This is a flaw that derived from the design of the rubber sealing mats of the DNA extraction kit, described in the revised manuscript.

Figures:
- The message from figure 2 is difficult to get? Change for instance its display into line plots and only marking significant differences? Since results fulfill a tested hypothesis or not and are significant different or not. Having a numerical scale for raw and post-hoc corrected p-values seems odd. Unless a point is made in the paper why to do so.

This part is no longer present in the Data Note format.

- Figure 3; I understand the power of the chosen analysis an display is the phylogenetic linking to the axes on display.
Readability of the top figure is however zero. Should be improved. The below figure using 16S seems out-of-context in the paper (see earlier remark). Furthermore it is unreadable. Maybe improve by substituting species names to letters and provide these in a legend.

This part is no longer present in the Data Note format.

- Figure 4: From which data the heatmap is inferred from? From all the different treatments or subset of only the controls?

This part is no longer present in the Data Note format.

- Supplementary figure4; perhaps move to main part of the paper?

Given the major edits in the Data Note format, we edited this figure to reflect the workflow described in the current manuscript, and as requested, included it as a figure, rather than as a supplementary figure.

- Remaining supplementary figures are too many for me (see earlier remark). Make a choice and condense to the main messages and explain these in the text.

Many figures and supplementary figures have now been removed in the Data Note format.

Textual:
- Line 206: revise NovaSeq S4 into NovaSeq 6000 (S4) or similar. The NCBI archive has it correct.

Revised.

- Line 186: amplification step should be elaborated.

Elaborated.

- Analysis methods:
o R exact version should be given as well as its used packages.

Only a few packages are now used in the Data Note format.

Versions are provided for all the tools used.

o The used random seed in all analysis is missing.

This part is no longer present in the Data Note format.

o To increase reproducibility the github repository should provide either a version-fixed set of scripts exactly as used
for the manuscript or a version tag to the exact used scripts. Otherwise, if scripts get updated in time reproduction of
results is no longer possible.

Good suggestion, we agree. We created a version tag.

- Line 508: …trend in community composition -changes?- over time?

This part is no longer present in the Data Note format.

- Lines 624-631 seem disjoint from the paper. Remove?

This part is no longer present in the Data Note format.

- Line 639: See remark above on DNA isolation bias and the Knudsen paper.

Reported on gram-pos/neg bias, and cited the Knudsen paper.

Close
