# Supplementary material for: A large-scale metagenomic survey dataset of the post-weaning piglet gut lumen
Source: Gigascience. 2021 Jun 3;10(6):giab039. doi: 10.1093/gigascience/giab039 (PMC8173662; doi:10.1093/gigascience/giab039)
Supplement: giab039_Response_to_Reviewer_Comments_Revision_1 [file giab039_response_to_reviewer_comments_revision_1.pdf]

Author's Response To Reviewer Comments

Close

Reviewer reports:
Reviewer #2: The authors have revised their Research paper into a Data Note.
Most of the initially raised concerns on data analysis are now redundant and consequently removed from the manuscript. Please note that this will not invalidate those comments for future publications.

RE: Thank you, we will indeed continue to revise and eventually resubmit that part of the work elsewhere as a separate manuscript. Your comments have already led to great improvements and we intend to acknowledge that in the “Acknowledgements” section of the next manuscript.

Main focus of the data note is/should be such that other researchers reaching out to this dataset can find all relevant details on experimental design, experimental approach and primary data processing in this manuscript.

- The authors have removed most of the concerning analysis details.

Few comments;
1) Data description / OTUs. Are OTUs used or "ASVs". I understand sortMeRNA was used that includes qiime v1.x but current methods use a DaDa2 similar approach moving away from OTUs (as is qiime v2.x for over a long period already)?

RE: Although we are aware of the advances that ASV inference methods have led to in the analysis of 16S amplicon sequencing data, there are no rigorous performant methods, that we are aware of, to obtain ASVs from shotgun metagenomic reads. We therefore left the existing analysis unchanged.

2) Data description: to largely describe (overall picture) a simple alpha-diversity plot and beta-diversity ordination plot would be an easy/quick way that brings more meaningful insight in describing the overall samples and individual variation observed. Now all data of the various groups is averaged per piglet or mother in a bardiagram/Krona plot which does not make sense since there is a large diversity within those groups of treatments.

RE: With regards to the diversity, an analysis of diversity (included in our first submission) suggested that differences in microbial community composition (alpha and beta diversity) between treatment groups was mild, while more prominent shifts of diversity were detected between samples from distinct time points (age of the piglets). The revised manuscript now includes a PCoA to describe the beta diversity of samples. We removed the Krona plot of the mothers, and merged the Krona plot of the piglets to a panel in a combined figure with the PCoA (beta diversity). The PCoA plots highlight the strong effect of time/aging, and the (dis)similarity between the mothers and piglets at distinct time points in the trial. In the revised submission we also report alpha diversity indices.

3) Supplement Fig2; make more clear that frequency is N samples. Also add bin size in legend for both sub figures.

RE: Done.

4) Supplement Fig4; would this be a possible result of using cfu which does not take into account dead/alive ratio's? That discussion/mentioning seems missing in the current text.

RE: Yes, that is true. This has now been added to the manuscript.

Close
