# Supplementary material for: A large-scale metagenomic survey dataset of the post-weaning piglet gut lumen
Source: Gigascience. 2021 Jun 3;10(6):giab039. doi: 10.1093/gigascience/giab039 (PMC8173662; doi:10.1093/gigascience/giab039)
Supplement: giab039_Response_to_Reviewer_Comments_Revision_2 [file giab039_response_to_reviewer_comments_revision_2.pdf]

Author's Response To Reviewer Comments

Close

AU: because you have removed Supp Table 3- please delete all citations to Supp table 3 throughout the paper.
RE: Supplementary Table 3 changed to Supplementary Table 2 (barcodes)

AU: Please add a final sentence in this section and say
"Snapshots of our code, and other data further supporting this work are openly available in the GigaScience respository, GigaDB [18]."

RE: Done.

AU: please add new Reference 18 and cite the GigaDB DOI in the exact format:
18. Gaio D; DeMaere MZ; Anantanawat K; Eamens GJ; Liu M; Zingali T; Falconer L; Chapman TA; Djordjevic SP; Darling AE: Supporting data for "A large-scale metagenomic survey dataset of the post-weaning piglet gut lumen" GigaScience Database. 2021. http://doi.org/10.5524/100890.

RE: Done.

Close
