# Supplementary material for: A large-scale metagenomic survey dataset of the post-weaning piglet gut lumen
Source: Gigascience. 2021 Jun 3;10(6):giab039. doi: 10.1093/gigascience/giab039 (PMC8173662; doi:10.1093/gigascience/giab039)
Supplement: giab039_Reviewer_1_Report_Original_Submission — Weilan Wang -- 12/11/2020 Reviewed [file giab039_reviewer_1_report_original_submission.pdf]

Reviewer Report

Title: A large-scale metagenomic survey dataset of the post-weaning piglet gut lumen

Version: Original Submission **Date:** 12/11/2020

Reviewer name: Weilan Wang

Reviewer Comments to Author:

This study reported 8Tbp of shotgun metagenomic sequence data for 800 fecal microbiota collected from 126 piglets and 42 sows. The samples were collected in a longitudinal manner and the treatments were well controlled. However, your data analysis, especially shotgun sequence data, needs to be improved.
Here are my questions:
1. Why you did shotgun metagenomic sequencing rather than 16S rRNA gene sequencing as you only did compositional analysis with this 8Tbp of sequence data. Analysis of 16S rRNA gene sequence could definitely do the same thing and it's much easier and cheaper. It there is any advantages of your analysis over the common used workflow with 16s rRNA gene sequence? The main advantage of shotgun sequence is the possibility to get more functional information other than bacterial composition of our metagenomic samples. Why you didn't do any functional analysis?
2. What is the significance of this study? The strong effect of time /age on gut microbiota of post-weanling pig have been reported in many publications.

Detailed comments:
Line 160 Taxonomy of the genus Lactobacillus has been reclassified. Please see more details in this reference : "A taxonomic note on the genus Lactobacillus: Description of 23 novel genera, emended description of the genus Lactobacillus Beijerinck 1901, and union of Lactobacillaceae and Leuconostocaceae"
Line 229 How is the quality of the extracted 16S gene reads? How many reads were extracted for each sample?
Line 230 How did you assign taxonomy to the extracted 16S gene reads? Based on sequencing identity? What are your parameters for difference taxonomic levels (e.g genus/ family levels)???? Did you cluster the identical reads into OTUs before assigning taxonomy?
Line 231 Why yo only use e-value for hits filter? How about the query coverage and identity?
Line 253 There are too many results and some of them are redundant and less informative. Some of the figures could be integrated into one and the less relevant results should be deleted.

Level of Interest

Please indicate how interesting you found the manuscript: Choose an item.

Quality of Written English

Please indicate the quality of language in the manuscript: Choose an item.

Declaration of Competing Interests

Please complete a declaration of competing interests, considering the following questions:

- Have you in the past five years received reimbursements, fees, funding, or salary from an organisation that may in any way gain or lose financially from the publication of this manuscript, either now or in the future?
- Do you hold any stocks or shares in an organisation that may in any way gain or lose financially from the publication of this manuscript, either now or in the future?
- Do you hold or are you currently applying for any patents relating to the content of the manuscript?
- Have you received reimbursements, fees, funding, or salary from an organization that holds or has applied for patents relating to the content of the manuscript?
- Do you have any other financial competing interests?
- Do you have any non-financial competing interests in relation to this paper?

If you can answer no to all of the above, write 'I declare that I have no competing interests' below. If your reply is yes to any, please give details below.

I declare that I have no competing interests' below

I agree to the open peer review policy of the journal. I understand that my name will be included on my report to the authors and, if the manuscript is accepted for publication, my named report including any attachments I upload will be posted on the website along with the authors' responses. I agree for my report to be made available under an Open Access Creative Commons CC-BY license (http://creativecommons.org/licenses/by/4.0/). I understand that any comments which I do not wish to be included in my named report can be included as confidential comments to the editors, which will not be published.

Choose an item.

To further support our reviewers, we have joined with Publons, where you can gain additional credit to further highlight your hard work (see: https://publons.com/journal/530/gigascience). On publication of this paper, your review will be automatically added to Publons, you can then choose whether or not to claim your Publons credit. I understand this statement.

Yes Choose an item.
