# Supplementary material for: A large-scale metagenomic survey dataset of the post-weaning piglet gut lumen
Source: Gigascience. 2021 Jun 3;10(6):giab039. doi: 10.1093/gigascience/giab039 (PMC8173662; doi:10.1093/gigascience/giab039)
Supplement: giab039_Reviewer_2_Report_Original_Submission — Alex Bossers -- 1/6/2021 Reviewed [file giab039_reviewer_2_report_original_submission.pdf]

Reviewer Report

Title: A large-scale metagenomic survey dataset of the post-weaning piglet gut lumen

Version: Original Submission **Date:** 1/6/2021

Reviewer name: Alex Bossers

Reviewer Comments to Author:

Gaio et al have analysed the phylogenetic diversity of the fecal pig bacterial communities obtained from a large temporal piglet study in the period just after weaning. An interesting period due to all sorts of practical problems that may arise during and after the weaning period. They used shotgun-metagenomics to determine that phylogenetic diversities are influenced by litter and mainly age. Changes induced by intra muscular injection of an aminoglycoside only had subtle effect on the community compositions. The metagenomic data described in this study and shared to the public by NCBI SRA provide a great resource to standardized microbiota data in pigs in the early age just after weaning. I thank the authors for learning other ways to approach metagenomic community structures.
Generic;
Even though the conclusions, mostly based on weighted phylogenetic diversity, seem supported by the data, overall the paper confuses me and seems rather lengthy for the actual analysis performed. The same accounts for the too many supplemental figures to this paper. Furthermore, I do have questions on why this approach was chosen in the first place, since I cannot derive that directly from the paper in its current form and more simple approaches would allow the same questions to be answered at the same or even better resolution.
The used shotgun-metagenomics seems rather overdone to the actual questions answered. I assume this paper will be a predecessor of another follow-up paper describing changes in the resistome as well as functional turn-over in time linked to the treatments. But I wonder whether salami-slicing this study would be the way to go. Furthermore, a lot of the data and outcomes are presented as-is while interpretation of these outcomes in context is rather thin and confusing using for instance both phylogenetic distance (PD) and the weighted forms.
More specific thoughts.
Even though they seem many, I hope they will help to improve the next version of the manuscript as constructive as possible;

1) A good balanced depth of sequencing per sample was chosen (around 30M PE150 clusters per sample) which seems sufficient as seen in other studies that used SGS in a metagenomics approach in pigs. But why on earth were only the first 100k reads for analysis?? The rationale for this as well as for instance rarefaction-like curves to demonstrate 100k is enough, are missing.
2) Why was SG-metagenomics used in the first place? All questions could have been answered by a far cheaper meta-taxonomic approach? SG-metagenomics studies are usually warranted if additional questions to bacterial composition are answered like:
   a. Non-bacterial microorganisms like simple eukaryotes, archaea and phages.
   b. Other gene compositions like the (acquired) resistome.

c. Functional turn-over.

The analyses could probably suffice from fairly simple permanova community composition comparisons, weighted unfirac beta-diversities and negative binomial modelled differential taxa analysis?

3) I miss from the paper the rationale for using unweighted and weighted PD's instead of for instance a straightforward weighted unifrac in beta-diversity analysis. It seems overcomplicated where the results describe every time both weighted and unweighted PD's. Is this necessary? And if so why? If weighted PD would be to go, leave out the rest. Would simplify and shorten considerably improving readability to a larger (metagenomic oriented) audience.

4) The title says "…gut microbiome.". Actually the gut was only sampled using a proxy of fecal sampling. Fecal composition is know not to be the same as in other regions of the gut. Revise throughout.

5) The piglet/litter origin linked to the sows were found to be of influence on the piglet fecal community. It would be interesting to include analysis to demonstrate whether the sow is the actual driver for the piglet community composition early and later in the study.

6) The interactions/influence of piglets "at random" in one pen may be interesting to investigate since it may influence the cross-sectional outcomes instead of following the same animal over time. It is known that the order in which taxa arrive disproportionate influence later community assembly (Sprochet et al 2018).

7) A Major point in this paper is made about the potential dysbiosis due to (oral) antibiotic treatment. Therefore intra muscular injection was used to study the effects on community composition.

a. However, since the microbiome and resistome diversity prior to starting antibiotic treatment is unknown and a control group having administered antibiotics orally, seems a bit odd.

b. Furthermore, it would make the study stronger to show that antibiotic residues are actually measured in feces. This can be done on fecal samples and would strengthen this part of the study.

c. The shotgun metagenomic data is there, why wasn't the effect of antibiotics on the resistome not included? This can be fairly easy achieved using mapping strategies.

8) Normalisation, sequencing and standardisation. The overall method is elegant using MiSeq to equilibrate libraries before sequencing. Some remarks;

a. The authors find that DNA isolation efficiency is biased for groups of species using their community controls. Was this a surprise? There is an elegant paper describing DNA isolation kit issues in metagenomic shotgun applications of for instance pig feces (Knudsen et al 2016 mSystems 1(5):e00095-16). Why not adhere to described methods?

b. Input is "standardized" on total from fecal samples isolated DNA measurements. It is known that especially due to dysbiosis (i.e. diarrhea) that the ratio of microbial DNA to host or feed-derived DNA may completely shift. The amount of reads per sample mapped to host or non-microbial origin is helpful and the microbial amounts should be used for normalization unless it is demonstrated that no significant differences are found between samples for this.

c. Hackflex: Seems an elegant method to try. However, I am not sure on editorial policy referencing a pre-publication by the same author in BioRxive of over a year old with apparent no peer-reviewed follow-up? Furthermore, pre-publications do not free authors from discussing/reflecting on comments by readers (if they make sense) to their pre-pub like the one over a year old without a response.

d. Mock communities / control libraries; elegantly made using a cfu approach.

i. However, also dead-bacteria will contribute DNA to the library. Since no strong cfu correlations are made this might be only of minor effect.

ii. Did the authors check for the presence of plasmids?

e. Why is a different method used for analyzing the (positive) controls using MethaPhlan2 while the rest of the samples is analysed by other means like PhyloSift. Positive controls should be used to benchmark the flow and analysis of the actual samples and therefore I would argue the methods should be the same.

f. Unfortunate some contaminants were detected in the positive (and probable negative) controls. How did the authors handle these outcomes? Were corrections applied to the data? Were taxa blacklisted or even removed?

9) Analysis methods;

a. The KR distance metric was used which seems rather comparable to weighted unifrac. I could not quickly recover if the KR metric returns Euclidian distances, if not revise the PCA into PCoA.

b. The weighted and unweighted PD is used. Shouldn't the unweighted PD be performed on rarefied data (at least for alpha-diversity analysis)? See for instance "McCoy and Matsen (2013) PeerJ 1:e157; DOI 10.7717/peerj.157".

c. Correcting for bath-effects was done using COMBAT. There is a no-go for me on this. The batch-effect should be taken as a covariate in the used statistical models and not used to correct the raw-data. This is elegantly demonstrated by Nygaard et al 2016 Biostatistics 17:29-39.

10) The section on sortmeRNA method and results are not well integrated in the manuscript and in its current state only deviate from the main message.

11) Lines 641-646: I found this a very honest remark but a major flaw in processing. If samples get contaminated from neighboring wells…. What do the actual results tell us?

Figures:

- The message from figure 2 is difficult to get? Change for instance its display into line plots and only marking significant differences? Since results fulfill a tested hypothesis or not and are significant different or not. Having a numerical scale for raw and post-hoc corrected p-values seems odd. Unless a point is made in the paper why to do so.
- Figure 3; I understand the power of the chosen analysis an display is the phylogenetic linking to the axes on display. Readability of the top figure is however zero. Should be improved. The below figure using 16S seems out-of-context in the paper (see earlier remark). Furthermore it is unreadable. Maybe improve by substituting species names to letters and provide these in a legend.
- Figure 4: From which data the heatmap is inferred from? From all the different treatments or subset of only the controls?
- Supplementary figure4; perhaps move to main part of the paper?
- Remaining supplementary figures are too many for me (see earlier remark). Make a choice and condense to the main messages and explain these in the text.

Textual:

- Line 206: revise NovaSeq S4 into NovaSeq 6000 (S4) or similar. The NCBI archive has it correct.
- Line 186: amplification step should be elaborated.
- Analysis methods:
  - o R exact version should be given as well as its used packages.

- The used random seed in all analysis is missing.
- To increase reproducibility the github repository should provide either a version-fixed set of scripts exactly as used for the manuscript or a version tag to the exact used scripts. Otherwise, if scripts get updated in time reproduction of results is no longer possible.

- Line 508: …trend in community composition -changes?- over time?
- Lines 624-631 seem disjoint from the paper. Remove?
- Line 639: See remark above on DNA isolation bias and the Knudsen paper.

Level of Interest

Please indicate how interesting you found the manuscript: Choose an item.

Quality of Written English

Please indicate the quality of language in the manuscript: Choose an item.

Declaration of Competing Interests

Please complete a declaration of competing interests, considering the following questions:

- Have you in the past five years received reimbursements, fees, funding, or salary from an organisation that may in any way gain or lose financially from the publication of this manuscript, either now or in the future?
- Do you hold any stocks or shares in an organisation that may in any way gain or lose financially from the publication of this manuscript, either now or in the future?
- Do you hold or are you currently applying for any patents relating to the content of the manuscript?
- Have you received reimbursements, fees, funding, or salary from an organization that holds or has applied for patents relating to the content of the manuscript?
- Do you have any other financial competing interests?
- Do you have any non-financial competing interests in relation to this paper?

If you can answer no to all of the above, write 'I declare that I have no competing interests' below. If your reply is yes to any, please give details below.

I declare that I have no competing interests

I agree to the open peer review policy of the journal. I understand that my name will be included on my report to the authors and, if the manuscript is accepted for publication, my named report including any attachments I upload will be posted on the website along with the authors' responses. I agree for my report to be made available under an Open Access Creative Commons CC-BY license

(http://creativecommons.org/licenses/by/4.0/). I understand that any comments which I do not wish to be included in my named report can be included as confidential comments to the editors, which will not be published.

Choose an item.

To further support our reviewers, we have joined with Publons, where you can gain additional credit to further highlight your hard work (see: https://publons.com/journal/530/gigascience). On publication of this paper, your review will be automatically added to Publons, you can then choose whether or not to claim your Publons credit. I understand this statement.

Yes Choose an item.
