# Supplementary material for: A large-scale metagenomic survey dataset of the post-weaning piglet gut lumen
Source: Gigascience. 2021 Jun 3;10(6):giab039. doi: 10.1093/gigascience/giab039 (PMC8173662; doi:10.1093/gigascience/giab039)
Supplement: giab039_Reviewer_2_Report_Revision_1 — Alex Bossers -- 3/22/2021 Reviewed [file giab039_reviewer_2_report_revision_1.pdf]

Reviewer Report

Title: A large-scale metagenomic survey dataset of the post-weaning piglet gut lumen

Version: Revision 1 **Date:** 3/22/2021

Reviewer name: Alex Bossers

Reviewer Comments to Author:

The authors have revised their Research paper into a Data Note.
Most of the initially raised concerns on data analysis are now redundant and consequently removed from the manuscript. Please note that this will not invalidate those comments for future publications.
Main focus of the data note is/should be such that other researchers reaching out to this dataset can find all relevant details on experimental design, experimental approach and primary data processing in this manuscript.
- The authors have removed most of the concerning analysis details.
Few comments;
1) Data description / OTUs. Are OTUs used or "ASVs". I understand sortMeRNA was used that includes qiime v1.x but current methods use a DaDa2 similar approach moving away from OTUs (as is qiime v2.x for over a long period already)?
2) Data description: to largely describe (overall picture) a simple alpha-diversity plot and beta-diversity ordination plot would be an easy/quick way that brings more meaningful insight in describing the overall samples and individual variation observed. Now all data of the various groups is averaged per piglet or mother in a bardiagram/Krona plot which does not make sense since there is a large diversity within those groups of treatments.
3) Supplement Fig2; make more clear that frequency is N samples. Also add bin size in legend for both sub figures.
4) Supplement Fig4; would this be a possible result of using cfu which does not take into account dead/alive ratio's? That discussion/mentioning seems missing in the current text.

Level of Interest

Please indicate how interesting you found the manuscript: Choose an item.

Quality of Written English

Please indicate the quality of language in the manuscript: Choose an item.

Declaration of Competing Interests

Please complete a declaration of competing interests, considering the following questions:

- Have you in the past five years received reimbursements, fees, funding, or salary from an organisation that may in any way gain or lose financially from the publication of this manuscript, either now or in the future?
- Do you hold any stocks or shares in an organisation that may in any way gain or lose financially from the publication of this manuscript, either now or in the future?
- Do you hold or are you currently applying for any patents relating to the content of the manuscript?
- Have you received reimbursements, fees, funding, or salary from an organization that holds or has applied for patents relating to the content of the manuscript?
- Do you have any other financial competing interests?
- Do you have any non-financial competing interests in relation to this paper?

If you can answer no to all of the above, write 'I declare that I have no competing interests' below. If your reply is yes to any, please give details below.

I declare that I have no competing interests.

I agree to the open peer review policy of the journal. I understand that my name will be included on my report to the authors and, if the manuscript is accepted for publication, my named report including any attachments I upload will be posted on the website along with the authors' responses. I agree for my report to be made available under an Open Access Creative Commons CC-BY license (http://creativecommons.org/licenses/by/4.0/). I understand that any comments which I do not wish to be included in my named report can be included as confidential comments to the editors, which will not be published.

Choose an item.

To further support our reviewers, we have joined with Publons, where you can gain additional credit to further highlight your hard work (see: https://publons.com/journal/530/gigascience). On publication of this paper, your review will be automatically added to Publons, you can then choose whether or not to claim your Publons credit. I understand this statement.

Yes Choose an item.
